# Supplementary material for: Impact of major depression on cardiovascular outcomes for individuals with hypertension: prospective survival analysis in UK Biobank
Source: BMJ Open. 2019 Sep 30;9(9):e024433. doi: 10.1136/bmjopen-2018-024433 (PMC6797415; doi:10.1136/bmjopen-2018-024433)
Supplement: Supplementary data [file bmjopen-2018-024433supp001.pdf]

Supplementary information for Impact of major depression on cardiovascular outcomes for individuals with hypertension: prospective study in UK Biobank. Graham et al

METHODS

New-onset cardiovascular outcomes

Date and cause of death were obtained from death certificates held by the National Health Service (NHS) Information Centre for participants from England and Wales and the NHS Central Register Scotland for participants from Scotland. Date and cause of hospital admissions were identified via record linkage to Health Episode Statistics (HES) records for England, the Patient Episode Database for Wales (PEDW) and to the Scottish Morbidity Records (SMR) for Scotland. Detailed information about the record linkage procedure is available online [1 2]. At the time of analysis, mortality data were available up to 31st January 2016 for England and Wales and 11th November 2015 for Scotland. Hospital admission data were available for the Scottish, English and Welsh participants until the 31st August 2014, 31st March 2015, and 28th February 2015 respectively. Therefore, for new cardiovascular events, end of follow up was classified as the hospital admission dates unless preceded by the date of death or the date of first cardiovascular event. New onset cardiovascular events were defined as an ICD 10 code of G45, G46, I20- I25, or I6 recorded on a death certificate or hospital admission. Deaths that predated the assessment date were excluded from analysis as presumed errors as were those in which data had only recorded a death date but no cause of death or a cause of death but no death date. Participants that had hospital admissions prior to the assessment date due to the aforementioned ICD10 codes were excluded as were not first episode. In addition, ICD-9 codes 430-438, 410-414, 429 and 429.2 were also excluded. hospital records are not available for the entire lifetime of study individuals, potentially missing some early cardiovascular events, as such those with self-declared prior cardiovascular disease at baseline were also excluded.

Blood Pressure

Graham N, *et al. BMJ Open* 2019; 9:e024433. doi: 10.1136/bmjopen-2018-024433

Blood pressure was measured in a sitting position partway through the interview and at the end of the interview using a digital blood pressure monitor (Omron HEM-7015IT.). Full protocol is available online https://biobank.ctsu.ox.ac.uk/crystal/docs/Bloodpressure.pdf

Depression definition

The criteria for lifetime MDD were created via the the following questions via touchscreen questionnaire were: *"Looking back over your life, have you ever had a time when you were feeling depressed or down for at least a whole week?"* (depression)*; "Have you ever had a period of time lasting at least two days when you were so irritable that you found yourself shouting at people or starting fights or arguments?"* (irritability); *"How many weeks was the longest period when you were feeling depressed or down?"* (duration); *"Have you ever seen a general practitioner (GP) for nerves, anxiety, tension or depression?"* (consulted GP); *"Have you ever seen a psychiatrist for nerves, anxiety, tension or depression?"* (consulted psychiatrist). Participants were classified as having a history of MDD if they reported at least one episode which comprised of depression and/or irritability, with a duration of at least two weeks, plus had consulted with either a general practitioner or psychiatrist for mental ill-health.

Physical activity

Physical activity was based on self-report, utilising the short form International Physical Activity Questionnaire (IPAQ). Participants reported the frequency and duration of moderate and vigorous activity along with walking undertaken in a typical week [3]. Data were analysed in accordance with the IPAQ scoring protocol [4] and total physical activity was computed as the sum of walking, moderate and vigorous activity, measured as metabolic equivalents (MET-hours/week). Physical activity was used in analyses as a continuous variable. Participants who reported greater than 24 hours a day doing all activity were classified as missing.

Graham N, *et al. BMJ Open* 2019; 9:e024433. doi: 10.1136/bmjopen-2018-024433

Sedentary behaviour

Sedentary behaviour duration was derived from the sum of self-reported time spent driving, using computer and watching television. Those stating that they had performed "less than an hour" of sedentary activities were coded as 0.5hrs to allow use of a continuous variable. Participants who reported greater than 24 hours a day doing all activity were classified as missing.

Socio-demographic and other covariates

Self-report on taking antihypertensive medication was taken from a question specific to cardiovascular medications, where antihypertensive medication was an option to respond. Area-based socioeconomic status was derived from postcode of residence, utilising the census-derived Townsend deprivation index scored on housing, employment, social class and car availability where a negative score represents greater affluence [5 6]. Age was calculated from dates of birth and baseline assessment date. Smoking status was categorised into never, former and current smoking based on self-report, those who wished not to answer were coded as missing. Drink frequency was categorised into daily, three or four times a week, once or twice a week, one to three times a month, special occasions only, and never based on self-report. Those who wished not to answer were coded as missing. Medical history of diabetes and high cholesterol was collected from the self-completed, baseline assessment questionnaire of medical conditions. Ethnicity was categorised as Caucasian, black/mixed and Asian/mixed based on self-report. Other ethnicities coded as missing due to small numbers. Age at completing full-time education was categorised as (<16, 16, >16). Height and body weight were measured by trained nurses during the initial assessment centre visit. Body mass index (BMI) was calculated as ($weight/height^2$) and the WHO criteria[7] to classify BMI into: underweight <18.5, normal weight 18.5-24.9, overweight 25.0-29.9 and obese ≥30.0 $kg.m^{-2}$. Psychotropic medication use was defined by the presence of pharmaceuticals from British National Formulary (BNF) chapters 4.1.1 to 4.3.4 [8] on self-report medication lists at baseline. Duration of hypertension was calculated utilising age and age of hypertension diagnosis. Antihypertensive medication count

was calculated as the absolute number of ACE inhibitors, angiotensin II receptor antagonists, calcium channel blockers, beta-blockers and thiazide diuretics prescribed to an individual. Generic medication names were sought and cross-referenced with the BNF chapters 2.2.1, 2.4, 2.5.5 and 2.6.2 [8].

Statistical analysis:

A best-fit multivariable regression spline model (stata command “mvrs”) was used to find the best model to adjust for non-linear covariates. For the adverse cardiovascular outcomes, A single knot was fitted for age at age 50 and two knots were fitted for total physical activity at 1.65 and 8.062 metabolic equivalent hours. In the male subgroup analysis two knots were fitted for total physical activity at 1.7 and 8.507 metabolic equivalent hours, in the female subgroup two knots were fitted for total physical activity at 1.57 and 3.75 and two knots were fitted at systolic blood pressure 121.5 and 147.5. No bends were noted in any models for the stroke outcomes.

Model selection and covariate adjustment

All variables were tested against outcome measures (cardiovascular outcomes and stroke outcomes) using univariate analysis to assess appropriateness for inclusion in the final model. All covariates were significantly associated with the outcomes. and were Two continuous variables, age and total physical activity, expressed non-linearity within the main analysis and male subgroup analysis for cardiovascular outcomes and as such regression splines were used with two and three knots respectively. Two knots were included within the female subgroup analysis for physical activity. For stroke outcome there were no bends in the main or sex-specific models.

Within the main analysis for cardiovascular outcomes, the groups of depression only, Asian/Asian British ethnicity and BMI<18.5 covariates failed the proportionality assumption and as such, were incorporated into the model as a time varying coefficients. Within the sex specific models depression

only failed the PH test within the female only analysis and ethnicity and BMI failed within the male only analysis. For the stroke outcomes gender and BMI class failed the proportionality assumption within the main analysis, with no failures within the sex-specific analysis. Analysis was repeated with the hypertension only as the comparator group to assess for any significant difference between the co-morbid group and the hypertension only group.

Time varying covariates

Due to the finding of MDD failing the proportionality assumption in the cardiovascular outcome in the primary analysis a series of further analyses have been performed to find when the assumption was not met. A log (-log) plot (fig 3) showed the proportionality assumption was broken at 22.5 months in the fully adjusted model in the primary analysis. As such, separate models were performed prior to and after these points. Prior to 22.5months the HR for MDD shows a trend that is reduced but insignificant (HR 0.82, 95%CI 0.6 - 1.13), becoming significantly increased after the 22.5 time point. (HR 1.27, 95%CI 1.06 - 1.52) (Table 9 supplementary digital content). Both stratified models passed the proportionality assumption using Schoenfeld residuals. Similar to the major analysis, the female model showed the MDD only group failing the proportionality assumption, although this was at the 29 month time point. (tables 6 and 10 of the supplementary digital content).

Graham N, *et al. BMJ Open* 2019; 9:e024433. doi: 10.1136/bmjopen-2018-024433

References

1. Palmer LJ. UK Biobank: bank on it. *Lancet* 2007;369(9578):1980-2. doi: 10.1016/S0140-6736(07)60924-6
2. Sudlow C, Gallacher J, Allen N, et al. UK Biobank: An Open Access Resource for Identifying the Causes of a Wide Range of Complex Diseases of Middle and Old Age. *Plos Medicine* 2015;12(3) doi: 10.1371/journal.pmed.1001779
3. Guo W, Bradbury KE, Reeves GK, et al. Physical activity in relation to body size and composition in women in UK Biobank. *Annals of Epidemiology* 2015;25(6):406-13.e6. doi: http://dx.doi.org/10.1016/j.annepidem.2015.01.015
4. Craig CL, Marshall AL, Sjostrom M, et al. International physical activity questionnaire: 12-country reliability and validity. *Medicine and science in sports and exercise* 2003;35(8):1381-95. doi: 10.1249/01.mss.0000078924.61453.fb [published Online First: 2003/08/06]
5. Townsend P. Deprivation. *Journal of Social Policy* 2009;16(02):125. doi: 10.1017/s0047279400020341
6. Townsend P, Phillimore M, Beattie A. Health and Deprivation: Inequality and the North. London: Croom Helm Ltd 1988.
7. WHO. Obesity: preventing and managing the global epidemic. Report of a WHO consultation. *World Health Organ Tech Rep Ser* 2000;894:i-xii, 1-253.
8. COMMITTEE. JF. British National Formulary. 67 ed. London: BMJ Group and Pharmaceutical Press 2014.

Graham N, *et al. BMJ Open* 2019; 9:e024433. doi: 10.1136/bmjopen-2018-024433

Supplementary Tables and figures

Supplementary Table1: Descriptive analysis for adverse cardiovascular outcome – males only

	Comparator group		Hypertension only		MDD only		Hypertension plus MDD	
	N= 21570		N = 30142		N = 4169		N = 5253	
**Median age (range)***	54	(47 - 61)	61	(54 - 65)	53	(46 - 60)	59	(52 - 64)
**Ethnicity, N (%)**								
White	19562	(90.69%)	27808	(92.26%)	3923	(94.1%)	5001	(95.2%)
Asian/Asian British	863	(4.%)	969	(3.21%)	87	(2.09%)	86	(1.64%)
Black/ Black British	559	(2.59%)	780	(2.59%)	52	(1.25%)	54	(1.03%)
**Median Townsend score (range)***	-1.87	(-3.47 - 0.59)	-2.08	(-3.53 - 0.41)	-1.58	(-3.3 - 1.07)	-1.81	(-3.44 - 0.78)
**Age at leaving full-time education, N (%)**								
<16	2517	(11.67%)	6328	(20.99%)	464	(11.13%)	1005	(19.13%)
16	4473	(20.74%)	6235	(20.69%)	859	(20.6%)	1096	(20.86%)
>16	14344	(66.5%)	17257	(57.25%)	2807	(67.33%)	3118	(59.36%)
**Total physical activity in metabolic**	4.15	(1.75 - 8.51)	3.99	(1.65 - 8.51)	4.15	(1.7 - 8.36)	3.76	(1.54 - 7.97)

Graham N, *et al. BMJ Open* 2019; 9:e024433. doi: 10.1136/bmjopen-2018-024433

**Sedentary time in hours, median (range)***	4.5	(3.5 - 6)	5	(3.5 - 6.5)	5	(3.5 - 6.5)	5	(4 - 7)
**Diabetes, N (%)**	721	(3.34%)	2401	(7.97%)	159	(3.81%)	477	(9.08%)
**Hypercholesterolaemia, N (%)**	1614	(7.48%)	5585	(18.53%)	363	(8.71%)	1056	(20.1%)
**Systolic BP in mmHg, median (range)***	128	(121.5 - 133.5)	149.5	(142 - 159)	127.5	(120.5 - 133)	148	(141 - 157)
**Body Mass Index, N (%)**								
<18.5	74	(0.34%)	35	(0.12%)	22	(0.53%)	12	(0.23%)
18.5 – 25	7607	(35.27%)	5842	(19.38%)	1394	(33.44%)	890	(16.94%)
25-30	10594	(49.11%)	15114	(50.14%)	2019	(48.43%)	2532	(48.2%)
>30	3295	(15.28%)	9151	(30.36%)	734	(17.61%)	1819	(34.63%)
**Smoking status, N (%)**								
Never smoked	12038	(55.81%)	15145	(50.25%)	1999	(47.95%)	2268	(43.18%)
Previously smoked	6777	(31.42%)	12125	(40.23%)	1447	(34.71%)	2295	(43.69%)
Current smoker	2688	(12.46%)	2776	(9.21%)	716	(17.17%)	686	(13.06%)
**Alcohol frequency, N (%)**								
Daily or almost daily	4822	(22.36%)	8653	(28.71%)	969	(23.24%)	1503	(28.61%)
Three or four times a week	5718	(26.51%)	7913	(26.25%)	1022	(24.51%)	1323	(25.19%)

Graham N, *et al. BMJ Open* 2019; 9:e024433. doi: 10.1136/bmjopen-2018-024433

Once or twice a week	5932	(27.5%)	7546	(25.03%)	1063	(25.5%)	1178	(22.43%)
One to three times a month	2193	(10.17%)	2392	(7.94%)	440	(10.55%)	479	(9.12%)
Special occasions only	1554	(7.2%)	2154	(7.15%)	328	(7.87%)	423	(8.05%)
Never	1343	(6.23%)	1473	(4.89%)	345	(8.28%)	345	(6.57%)
**Psychotropic medication, N (%)**	398	(1.85%)	670	(2.22%)	678	(16.26%)	879	(16.73%)

*All data presented as N (%) and has chi-squared p-value of <0.001 except * which are median values (interquartile range) and have a Kruskal-Wallis p-value of 0.0001. Data presented as MET-hrs (hours spent doing exercise adjusted for multiples of basal metabolic rate in accordance with IPAQ).Townsend score is an area based measure based on census statistics. It is a calculation based on the number of: households without a car, overcrowded households, households not owner-occupied and unemployment.*

Graham N, *et al. BMJ Open* 2019; 9:e024433. doi: 10.1136/bmjopen-2018-024433

Supplementary Table 2: Descriptive analysis for adverse cardiovascular outcome – females only

	Comparator group		Hypertension only		MDD only		Hypertension plus MDD	
	N= 29228		N = 25893		N = 10929		N = 7676	
**Median age (range)***	54	(47 - 61)	61	(55 - 65)	53	(47 - 60)	60	(53 - 64)
**Ethnicity, N (%)**								
White	26585	(90.96%)	23441	(90.53%)	10324	(94.46%)	7271	(94.72%)
Asian/Asian British	908	(3.11%)	727	(2.81%)	174	(1.59%)	93	(1.21%)
Black/ Black British	764	(2.61%)	989	(3.82%)	167	(1.53%)	168	(2.19%)
**Median Townsend score (range)***	-1.90	(-3.44 - 0.51)	-2.06	(-3.5 - 0.38)	-1.66	(-3.3 - 0.84)	-1.87	(-3.4 - 0.74)
**Age at leaving full-time education, N (%)**								
<16	3399	(11.63%)	5757	(22.23%)	1261	(11.54%)	1602	(20.87%)
16	5792	(19.82%)	5592	(21.6%)	2319	(21.22%)	1636	(21.31%)
>16	19746	(67.56%)	14223	(54.93%)	7283	(66.64%)	4385	(57.13%)
**Total physical activity in metabolic**	3.87	(1.65 - 7.71)	3.51	(1.37 - 7.59)	3.79	(1.65 - 7.91)	3.65	(1.45 - 7.93)
**Sedentary time in hours, median (range)***	4	(3 - 5)	4	(3 - 5.5)	4	(3 - 5.5)	4.5	(3 - 6)

Graham N, *et al. BMJ Open* 2019; 9:e024433. doi: 10.1136/bmjopen-2018-024433

**Diabetes, N (%)**	547	(1.87%)	1376	(5.31%)	221	(2.02%)	452	(5.89%)
**Hypercholesterolaemia, N (%)**	1397	(4.78%)	3625	(14.%)	530	(4.85%)	1155	(15.05%)
**Systolic BP in mmHg, median (range)***	123.5	(115.5 - 130.5)	149.5	(142 - 160)	122.5	(114.5 - 130)	147.5	(140.5 - 157)
**Body Mass Index, N (%)**								
<18.5	315	(1.08%)	107	(0.41%)	81	(0.74%)	22	(0.29%)
18.5 – 25	14942	(51.12%)	7836	(30.26%)	4857	(44.44%)	1984	(25.85%)
25-30	9816	(33.58%)	10102	(39.01%)	3917	(35.84%)	2857	(37.22%)
>30	4155	(14.22%)	7848	(30.31%)	2074	(18.98%)	2813	(36.65%)
**Smoking status, N (%)**								
Never smoked	18588	(63.6%)	16358	(63.18%)	5865	(53.66%)	4186	(54.53%)
Previously smoked	8279	(28.33%)	8015	(30.95%)	3671	(33.59%)	2770	(36.09%)
Current smoker	2282	(7.81%)	1423	(5.5%)	1377	(12.6%)	695	(9.05%)
**Alcohol frequency, N (%)**								
Daily or almost daily	4628	(15.83%)	4317	(16.67%)	1767	(16.17%)	1378	(17.95%)
Three or four times a week	6457	(22.09%)	5120	(19.77%)	2231	(20.41%)	1514	(19.72%)
Once or twice a week	7712	(26.39%)	6343	(24.5%)	2817	(25.78%)	1738	(22.64%)

One to three times a month	3859	(13.2%)	3196	(12.34%)	1618	(14.8%)	1033	(13.46%)
Special occasions only	3980	(13.62%)	4176	(16.13%)	1576	(14.42%)	1306	(17.01%)
Never	2581	(8.83%)	2726	(10.53%)	917	(8.39%)	703	(9.16%)
**Psychotropic medication, N (%)**	943	(3.23%)	1125	(4.34%)	2166	(19.82%)	1643	(21.4%)

*All data presented as N (%) and has chi-squared p-value of <0.001 except * which are median values (interquartile range) and have a Kruskal-Wallis p-value of 0.0001. Data presented as MET-hrs (hours spent doing exercise adjusted for multiples of basal metabolic rate in accordance with IPAQ).Townsend score is an area based measure based on census statistics. It is a calculation based on the number of: households without a car, overcrowded households, households not owner-occupied and unemployment.*

Supplementary Table 3: Descriptive analysis for stroke outcome – males only

	Comparator group		Hypertension only		MDD only		Hypertension plus MDD	
	N = 22816		N = 32787		N = 4438		N = 5857	
**Median age (range)***	55	(47 - 62.)	61	(54 - 65)	54	(47 - 61)	60	(53 - 64)
**Ethnicity, N (%)**								
White	20699	(90.72%)	30219	(92.17%)	4173	(94.03%)	5569	(95.08%)
Asian/Asian British	932	(4.08%)	1116	(3.4%)	102	(2.3%)	105	(1.79%)
Black/ Black British	576	(2.52%)	820	(2.5%)	53	(1.19%)	59	(1.01%)
**Median Townsend score (range)***	-1.88	(-3.47 - 0.59)	-2.05	(-3.5 - 0.46)	-1.56	(-3.28 - 1.15)	-1.74	(-3.4 - 0.93)
**Age at leaving full-time education, N (%)**								
<16	2900	(12.71%)	7256	(22.13%)	558	(12.57%)	1193	(20.37%)
16	4702	(20.61%)	6704	(20.45%)	909	(20.48%)	1222	(20.86%)
>16	14960	(65.57%)	18471	(56.34%)	2930	(66.02%)	3397	(58.%)
**Total physical activity in metabolic**	4.12	(1.74 - 8.48)	3.96	(1.65 - 8.44)	4.13	(1.67 - 8.36)	3.66	(1.45 - 7.83)
**Sedentary time in hours, median (range)***	5	(3.5 - 6)	5	(4 - 7)	5	(3.5 - 6.5)	5	(4 - 7)

Graham N, *et al. BMJ Open* 2019; 9:e024433. doi: 10.1136/bmjopen-2018-024433

**Diabetes, N (%)**	873	(3.83%)	2951	(9.%)	208	(4.69%)	635	(10.84%)
**Hypercholesterolaemia, N (%)**	2045	(8.96%)	6736	(20.54%)	457	(10.3%)	1293	(22.08%)
**Systolic BP in mmHg, median (range)***	128	(121.5 - 133.5)	149	(142 - 159)	127	(120.5 - 133)	147.5	(140.5 - 156.5)
**Body Mass Index, N (%)**								
<18.5	79	(0.35%)	39	(0.12%)	22	(0.5%)	12	(0.2%)
18.5 – 25	7867	(34.48%)	6215	(18.96%)	1452	(32.72%)	960	(16.39%)
25-30	11203	(49.1%)	16341	(49.84%)	2142	(48.26%)	2780	(47.46%)
>30	3667	(16.07%)	10192	(31.09%)	822	(18.52%)	2105	(35.94%)
**Smoking status, N (%)**								
Never smoked	12502	(54.79%)	16054	(48.96%)	2094	(47.18%)	2469	(42.15%)
Previously smoked	7399	(32.43%)	13603	(41.49%)	1582	(35.65%)	2610	(44.56%)
Current smoker	2836	(12.43%)	3013	(9.19%)	754	(16.99%)	770	(13.15%)
**Alcohol frequency, N (%)**								
Daily or almost daily	5085	(22.29%)	9309	(28.39%)	1021	(23.01%)	1645	(28.09%)
Three or four times a week	6039	(26.47%)	8556	(26.1%)	1077	(24.27%)	1450	(24.76%)
Once or twice a week	6264	(27.45%)	8161	(24.89%)	1121	(25.26%)	1305	(22.28%)

One to three times a month	2307	(10.11%)	2642	(8.06%)	478	(10.77%)	538	(9.19%)
Special occasions only	1666	(7.3%)	2394	(7.3%)	355	(8.%)	503	(8.59%)
Never	1444	(6.33%)	1711	(5.22%)	383	(8.63%)	414	(7.07%)
**Psychotropic medication, N (%)**	429	(1.88%)	793	(2.42%)	735	(16.56%)	1025	(17.5%)

*All data presented as N (%) and has chi-squared p-value of <0.001 except * which are median values (interquartile range) and have a Kruskal-Wallis p-value of 0.0001. Data presented as MET-hrs (hours spent doing exercise adjusted for multiples of basal metabolic rate in accordance with IPAQ).Townsend score is an area based measure based on census statistics. It is a calculation based on the number of: households without a car, overcrowded households, households not owner-occupied and unemployment.*

Supplementary Table 4: Descriptive analysis for stroke outcome – females only

	Comparator group		Hypertension only		MDD only		Hypertension plus MDD	
	N = 29684		N = 26937		N = 11143		N = 8090	
**Median age (range)***	54	(47 - 61)	61	(56 - 65)	53	(47 - 60)	60	(54 - 64)
**Ethnicity, N (%)**								
White	26998	(90.95%)	24359	(90.43%)	10524	(94.44%)	7643	(94.47%)
Asian/Asian British	925	(3.12%)	773	(2.87%)	178	(1.6%)	104	(1.29%)
Black/ Black British	779	(2.62%)	1034	(3.84%)	170.00	(1.53%)	187	(2.31%)
**Median Townsend score (range)***	-1.90	(-3.44 - 0.52)	-2.03	(-3.48 - 0.43)	-1.66	(-3.29 - 0.86)	-1.83	(-3.38 - 0.85)
**Age at leaving full-time education, N (%)**								
<16	3546	(11.95%)	6140	(22.79%)	1326	(11.9%)	1752	(21.66%)
16	5888	(19.84%)	5803	(21.54%)	2361	(21.19%)	1731	(21.4%)
>16	19954	(67.22%)	14643	(54.36%)	7387	(66.29%)	4550	(56.24%)
**Total physical activity in metabolic**	3.85	(1.65 - 7.7)	3.49	(1.35 - 7.57)	3.79	(1.65 - 7.89)	3.61	(1.41 - 7.87)

Graham N, *et al. BMJ Open* 2019; 9:e024433. doi: 10.1136/bmjopen-2018-024433

**Sedentary time in hours, median (range)***	4.0	(3 - 5)	4.0	(3 - 5.5)	4.0	(3 - 5.5)	4.5	(3 - 6)
**Diabetes, N (%)**	581	(1.96%)	1551	(5.76%)	241	(2.16%)	528	(6.53%)
**Hypercholesterolaemia, N (%)**	1547	(5.21%)	4032	(14.97%)	592	(5.31%)	1327	(16.4%)
**Systolic BP in mmHg, median (range)***	123.5	(115.5 - 131)	149.5	(142 - 160)	122.5	(114.5 - 130)	147.0	(140.5 - 157)
**Body Mass Index, N (%)**								
<18.5	316	(1.06%)	112	(0.42%)	82	(0.74%)	26	(0.32%)
18.5 – 25	15100	(50.87%)	8027	(29.8%)	4922	(44.17%)	2057	(25.43%)
25-30	9982	(33.63%)	10476	(38.89%)	4007	(35.96%)	2989	(36.95%)
>30	4286	(14.44%)	8322	(30.89%)	2132	(19.13%)	3018	(37.31%)
**Smoking status, N (%)**								
Never smoked	18816	(63.39%)	16928	(62.84%)	5958	(53.47%)	4365	(53.96%)
Previously smoked	8452	(28.47%)	8416	(31.24%)	3758	(33.73%)	2950	(36.46%)
Current smoker	2334	(7.86%)	1488	(5.52%)	1409	(12.64%)	749	(9.26%)
**Alcohol frequency, N (%)**								

Daily or almost daily	4675	(15.75%)	4442	(16.49%)	1796	(16.12%)	1440	(17.8%)
Three or four times a week	6524	(21.98%)	5271	(19.57%)	2258	(20.26%)	1570	(19.41%)
Once or twice a week	7825	(26.36%)	6558	(24.35%)	2872	(25.77%)	1820	(22.5%)
One to three times a month	3913	(13.18%)	3329	(12.36%)	1644	(14.75%)	1089	(13.46%)
Special occasions only	4078	(13.74%)	4400	(16.33%)	1623	(14.57%)	1382	(17.08%)
Never	2658	(8.95%)	2919	(10.84%)	947	(8.5%)	785	(9.7%)
**Psychotropic medication, N (%)**	979	(3.3%)	1203	(4.47%)	2241	(20.11%)	1753	(21.67%)

*All data presented as N (%) and has chi-squared p-value of <0.001 except * which are median values (interquartile range) and have a Kruskal-Wallis p-value of 0.0001. Data presented as MET-hrs (hours spent doing exercise adjusted for multiples of basal metabolic rate in accordance with IPAQ).Townsend score is an area based measure based on census statistics. It is a calculation based on the number of: households without a car, overcrowded households, households not owner-occupied and unemployment.*

Supplementary Table 5: Risk of adverse cardiovascular event by clinical group, in males only.

	Model one (unadjusted)			Model two (partially adjusted)*			Model three (fully adjusted) †		
**Group**	HR	95% C.I.	p-value	aHR	95% C.I.	p-value	aHR	95% C.I.	p-value
No Hypertension- No MDD	1(ref)			1(ref)			1(ref)		
Hypertension only	2.21	(2.00-2.45)	2.28×10^{-53}	1.62	(1.46-1.83)	5.80×10^{-19}	1.29	(1.13-1.47)	1.35×10^{-4}
MDD only	1.17	(0.95-1.56)	0.12	1.18	(0.95-1.46)	0.12	1.12	(0.9-1.39)	0.3
Hypertension and MDD	2.46	(2.13-2.84)	3.12×10^{-34}	1.95	(1.68-2.27)	2.81×10^{-18}	1.47	(1.24-1.74)	8.71×10^{-6}

**Adjusted for sociodemographic factors (age, Townsend score, age of leaving full time education and ethnicity. †Additionally adjusted for history of diabetes, history of hypercholesterolemia, BMI, smoking history, alcohol use, systolic blood pressure, sedentary hours per day, physical activity and psychotropic medication use. MDD = Major depressive disorder, HR = Hazard ratio, aHR = Adjusted hazard ratio, C.I.= Confidence interval*

Graham N, *et al. BMJ Open* 2019; 9:e024433. doi: 10.1136/bmjopen-2018-024433

Supplementary Table 6: Risk of adverse cardiovascular event by clinical group, in females only.

	Model one (unadjusted)			Model two (partially adjusted)*			Model three (fully adjusted) †		
**Group**	HR	95% C.I.	p-value	aHR	95% C.I.	p-value	aHR	95% C.I.	p-value
No Hypertension - No MDD	1(ref)			1(ref)			1(ref)		
Hypertension only	2.75	(2.38 - 3.18)	6.16×10^{-43}	1.86	(1.6-2.17)	1.43×10^{-15}	1.64	(1.33-2.02)	4.36×10^{-6}
MDD only	0.67	(0.42-1.08)	0.10	0.72	(0.45-1.17)	0.19	0.68	(0.42-1.1)	0.12
Hypertension and MDD	3.68	(3.1-4.38)	5.62×10^{-49}	2.78	(1.58-3.29)	4.62×10^{-29}	2.18	(1.82-2.92)	4.76×10^{-11}
**Time varying Variables**									
MDD only	1.02	(1.006-1.03)	2.45×10^{-3}	1.02	(1.005-1.03)	4.00×10^{-3}	1.02	(1.004-1.03)	6.19×10^{-3}

**Adjusted for sociodemographic factors (age, Townsend score, age of leaving full time education and ethnicity. †Additionally adjusted for history of diabetes, history of hypercholesterolemia, BMI, smoking history, alcohol use, systolic blood pressure, sedentary hours per day, physical activity and psychotropic medication use. MDD = Major depressive disorder, HR = Hazard ratio, aHR = Adjusted hazard ratio, C.I.= Confidence interval*

Graham N, *et al. BMJ Open* 2019; 9:e024433. doi: 10.1136/bmjopen-2018-024433

Supplementary Table 7: Risk of stroke event by clinical group, in males only.

	Model one (unadjusted)			Model two (partially adjusted)*			Model three (fully adjusted) †		
**Group**	HR	95% C.I.	p-value	aHR	95% C.I.	p-value	aHR	95% C.I.	p-value
No Hypertension - No MDD	1(ref)			1(ref)			1(ref)		
Hypertension only	2.43	(1.95 - 3.03)	1.92×10^{-15}	1.74	(1.38 - 2.19)	2.58×10^{-6}	1.19	(0.9 - 1.58)	0.22
MDD only	1.45	(0.96 - 2.2)	0.07	1.65	(1.09 - 2.5)	0.02	1.49	(0.97 - 2.29)	0.07
Hypertension and MDD	2.39	(1.74 - 3.27)	7.34×10^{-8}	1.87	(1.35 - 2.6)	1.55×10^{-4}	1.20	(0.83 - 1.74)	0.33

**Adjusted for sociodemographic factors (age, sex, Townsend score, age of leaving full time education and ethnicity. †Additionally adjusted for history of diabetes, history of hypercholesterolemia, BMI, smoking history, alcohol use, systolic blood pressure, sedentary hours per day, physical activity and psychotropic medication use. MDD = Major depressive disorder, HR = Hazard ratio, aHR = Adjusted hazard ratio, C.I.= Confidence interval*

Supplementary Table 8: Risk of stroke event by clinical group, in females only.

	Model one (unadjusted)			Model two (partially adjusted)*			Model three (fully adjusted) †		
**Group**	HR	95% C.I.	p-value	aHR	95% C.I.	p-value	aHR	95% C.I.	p-value
No Hypertension - No MDD	1(ref)			1(ref)			1(ref)		
Hypertension only	2.38	(1.84 - 3.09)	6.50×10^{-11}	1.51	(1.14 - 1.99)	3.63×10^{-3}	1.25	(0.88 - 1.79)	0.21
MDD only	1.09	(0.73 - 1.62)	0.67	1.15	(0.76 - 1.75)	0.51	0.99	(0.64 - 1.53)	0.98
Hypertension and MDD	3.05	(2.22 - 4.21)	8.71×10^{-12}	2.22	(1.59 - 3.08)	2.27×10^{-6}	1.62	(1.08 - 2.42)	0.02

**Adjusted for sociodemographic factors (age, sex, Townsend score, age of leaving full time education and ethnicity. †Additionally adjusted for history of diabetes, history of hypercholesterolemia, BMI, smoking history, alcohol use, systolic blood pressure, sedentary hours per day, physical activity and psychotropic medication use. MDD = Major depressive disorder, HR = Hazard ratio, aHR = Adjusted hazard ratio, C.I.= Confidence interval*

Graham N, *et al. BMJ Open* 2019; 9:e024433. doi: 10.1136/bmjopen-2018-024433

Supplementary Table 9: Time stratified analysis by moment of proportional hazards failure for adverse cardiovascular outcomes (stratified at 22.5 months)

	Fully adjusted* model pre-22.5 months			Fully adjusted* model post-22.5 months		
**Group**	aHR	95% C.I.	p-value	aHR	95% C.I.	p-value
No Hypertension - No MDD	1(ref)			1(ref)		
Hypertension only	1.36	(1.12 - 1.66)	0.002	1.36	(1.19 -1.55)	$5.06x10^{-6}$
MDD only	0.82	(0.60 - 1.13)	0.22	1.27	(1.06 -1.52)	0.01
Hypertension and MDD	1.75	(1.39 - 2.21)	$2.62x10^{-6}$	1.62	(1.38 - 1.90)	$5.72x10^{-9}$

**Adjusted for sociodemographic factors (age, sex, Townsend score, age of leaving full time education and ethnicity, history of diabetes, history of hypercholesterolemia, BMI, smoking history, alcohol use, systolic blood pressure, sedentary hours per day, physical activity and psychotropic medication use.*

MDD = Major depressive disorder, aHR =adjusted hazard ratio, C.I.= Confidence interval

Graham N, *et al. BMJ Open* 2019; 9:e024433. doi: 10.1136/bmjopen-2018-024433

Supplementary Table 10: Time stratified analysis by moment of proportional hazards failure for adverse cardiovascular outcomes (females only - stratified at 29 months)

	Fully adjusted* model pre-29 months			Fully adjusted* model post-29 months		
**Group**	HR	95% C.I.	p-value	HR	95% C.I.	p-value
No Hypertension - No MDD	1(ref)			1(ref)		
Hypertension only	1.49	(1.06 - 2.08)	0.02	1.75	(1.33 - 2.30)	5.56×10^{-5}
MDD only	0.73	(0.48 - 1.10)	0.13	1.58	(1.19 - 2.09)	0.002
Hypertension and MDD	1.80	(1.24 - 2.62)	0.002	2.47	(1.83 - 3.33)	2.89×10^{-9}

**Adjusted for sociodemographic factors (age, sex, Townsend score, age of leaving full time education and ethnicity, history of diabetes, history of hypercholesterolemia, BMI, smoking history, alcohol use, systolic blood pressure, sedentary hours per day, physical activity and psychotropic medication use.*

MDD = Major depressive disorder, aHR =adjusted hazard ratio, C.I.= Confidence interval

Graham N, *et al. BMJ Open* 2019; 9:e024433. doi: 10.1136/bmjopen-2018-024433

Supplementary Table 11: Relative excess risk due to interaction results on fully adjusted* models

Analysis	RERI	95% C.I.	LR test p-value
Adverse cardiovascular outcome before 22.5 months	**0.563**	**(0.189 - 0.938)**	**0.0116**
Adverse cardiovascular outcome after 22.5 months	-0.009	(-0.293 - 0.275)	0.563
Adverse cardiovascular outcome (males only)	0.058	(-0.240 - 0.357)	0.899
Adverse cardiovascular outcome (females only)before 29 months	**0.588**	**(0.074 - 1.103)**	**0.031**
Adverse cardiovascular outcome (females only)after 29 months	0.142	(-0.447 - 0.732)	0.5173
Stroke outcome	-0.047	(-0.485 - 0.391)	0.7271
Stroke outcome (males only)	-0.480	(-1.195 - 0.234)	0.1376
Stroke outcome (females only)	0.372	(-0.216 - 0.959)	0.314

**Adjusted for sociodemographic factors (age, sex, Townsend score, age of leaving full time education and ethnicity, history of diabetes, history of hypercholesterolemia, BMI, smoking history, alcohol use, systolic blood pressure, sedentary hours per day, physical activity and psychotropic medication use.*

RERI = Relative excess risk due to interaction (additive interaction), C.I.= Confidence interval, LR test = likelihood ratio test (multiplicative interaction)

Supplementary Table 12: Comparison of additional hypertension factors (medication and diagnosis duration) across groups

	No Hypertension – No MDD		Hypertension only		MDD only		Hypertension and MDD	
**Antihypertensive medication prescription, N (%)**	1,265	(2.49)	19,045	(33.99)	476	(3.04)	5,037	(37.34)
**Number of antihypertensive medications, N (range)***	1	(1-1)	1	(1-2)	1	(1-1)	1	(1- 2)
**Reported a duration of hypertension, N (%)**	*1,376*	(2.71)	*16,709*	(29.82)	678	(4.32)	4,525	(33.55)
**Duration of hypertension in years, median (range)***	6	(2-14)	8	(4-13)	6	(3-14)	8	(4 - 14)

*****Median quantity of antihpertensive medications and median duration of hypertensive diagnosis presented for those on antihypertensive medications and supplied an age of hypertension diagnosis, respectively. MDD = Major Depressive disorder*

Graham N, *et al. BMJ Open* 2019; 9:e024433. doi: 10.1136/bmjopen-2018-024433
