# Supplementary material for: Impact of major depression on cardiovascular outcomes for individuals with hypertension: prospective survival analysis in UK Biobank
Source: BMJ Open. 2019 Sep 30;9(9):e024433. doi: 10.1136/bmjopen-2018-024433 (PMC6797415; doi:10.1136/bmjopen-2018-024433)
Supplement: Author's manuscript [file bmjopen-2018-024433.draft_revisions.pdf]

BMJ Open

BMJ Open is committed to open peer review. As part of this commitment we make the peer review history of every article we publish publicly available.

When an article is published we post the peer reviewers' comments and the authors' responses online. We also post the versions of the paper that were used during peer review. These are the versions that the peer review comments apply to.

The versions of the paper that follow are the versions that were submitted during the peer review process. They are not the versions of record or the final published versions. They should not be cited or distributed as the published version of this manuscript.

BMJ Open is an open access journal and the full, final, typeset and author-corrected version of record of the manuscript is available on our site with no access controls, subscription charges or pay-per-view fees (http://bmjopen.bmj.com).

If you have any questions on BMJ Open's open peer review process please email info.bmjopen@bmj.com

BMJ Open

Impact of major depression on cardiovascular outcomes for individuals with hypertension: prospective survival analysis in UK Biobank.

Journal:	*BMJ Open*
Manuscript ID	bmjopen-2018-024433
Article Type:	Research
Date Submitted by the Author:	25-May-2018
Complete List of Authors:	Graham, Nicholas; University of Glasgow Institute of Health and Wellbeing, Gartnavel Royal Hopsital 1055 Great Western Road Glasgow, UK G12 0XH<br>Ward, Joey; University of Glasgow Institute of Health and Wellbeing<br>Mackay, Daniel; University of Glasgow Institute of Health and Wellbeing<br>Pell, J. P.; University of Glasgow Institute of Health and Wellbeing<br>Cavanagh, Jonathan; University of Glasgow Institute of Health and Wellbeing<br>Padmanabhan, Sandosh; University of Glasgow, Institute of Cardiovascular and Medical Sciences, British Heart Foundation Glasgow Cardiovascular Research Centre<br>Smith, Daniel; University of Glasgow, Institute of Health and Wellbeing
Keywords:	EPIDEMIOLOGY, mortality, cardiovascular disease, morbidity, depression, Hypertension < CARDIOLOGY

SCHOLARONE™
Manuscripts

For peer review only

Impact of major depression on cardiovascular outcomes for individuals with hypertension: prospective survival analysis in UK Biobank.

Short title: Outcomes of Hypertension plus Depression

Nicholas A GRAHAM*[a], Clinical Research Fellow

Joey WARD[a], Research Fellow

Daniel MACKAY[b], Reader in Public Health

Jill PELL[b], Professor of Public Health

Jonathan CAVANAGH[c], Professor of Psychiatry

Sandosh PADMANABHAN[d], Professor of Cardiovascular Genomics and Therapeutics

Daniel J. SMITH[a], Professor of Psychiatry.

Number of Supplementary files: 1

Word count of Manuscript: 3,873 (exc. Tables, references, abstract summary and Author contribution statements)

Word count of Supplementary file: 1,451 (exc. tables)

Number of tables and figures: 18 tables (including 12 in supplementary digital content) and 3 figures

[a]Institute of Health and Wellbeing, University of Glasgow, Gartnavel Royal Hospital, 1055 Great Western Road, Glasgow G12 OXH. [b]Institute of Health and Wellbeing, University of Glasgow, Public Health, 1 Lilybank Gardens, Glasgow G12 8RZ. [c]Institute of Health and Wellbeing, Centre for Immunobiology, Sir Graeme Davies Building College of Medical, Veterinary and Life Sciences University of Glasgow. [d]Institute of Cardiovascular and Medical Sciences, British Heart Foundation Glasgow Cardiovascular Research centre, University of Glasgow, Glasgow G12 8TA.

For peer review only - http://bmjopen.bmj.com/site/about/guidelines.xhtml

*corresponding author: nicholas.graham@glasgow.ac.uk, phone: +44 0141 211 3918,

CONFLICTS OF INTEREST: None.

For peer review only

For peer review only - http://bmjopen.bmj.com/site/about/guidelines.xhtml

ABSTRACT

Objectives: To assess whether a history of MDD in middle-aged individuals with hypertension impacts on medium-term cardiovascular disease outcomes.

Design: Prospective cohort survival analysis using Cox proportional hazards regression with a median follow-up of 63 months (702,902 person-years). Four mutually exclusive groups were compared: hypertension only (n=56,035), MDD only (n=15,098), comorbid hypertension plus MDD (n=12,929), and an unaffected (no hypertension, no MDD) comparison group (n=50,798).

Setting: UK Biobank

Participants: UK Biobank participants without cardiovascular disease aged 37–73 who completed additional psychiatric questions at baseline interview in 2006–2010 (n=134,860).

Primary and Secondary outcome measures: First time adverse cardiovascular outcomes leading to hospital admission or death (ICD-10 codes I20-I259, I60-69 and G45- G46) adjusted in a stepwise manner for sociodemographic, health and lifestyle features. Secondary analyses were performed looking specifically at stroke outcomes (ICD-10 codes I60-69 and G45- G46)and in models separated by gender.

Results: Relative to controls, adjusted hazard ratios (HRs) for adverse cardiovascular outcomes were increased for the hypertension only group (HR=1.36, 95%CI 1.22-1.52) and were higher still for the comorbid hypertension plus MDD group (HR=1.66, 95%CI 1.45-1.9). HRs for the comorbid hypertension plus MDD group were significantly raised compared to hypertension alone (HR=1.22, 95%CI 1.1-1.35).

Conclusions: Comorbid hypertension and depression conferred greater hazard than hypertension alone for adverse cardiovascular outcomes, although evidence of an additive interaction is inconsistent. Future cardiovascular risk prediction tools may benefit from the inclusion of questions about prior history of depressive disorders.

Word count of Abstract: 239

For peer review only - http://bmjopen.bmj.com/site/about/guidelines.xhtml

Key words: epidemiology, mortality, morbidity, depression; hypertension, cardiovascular disease

Article Summary

STRENGTHS AND LIMITATIONS

- There were methodological advantages over similar studies including a very large sample size, adjustment for a more comprehensive range of confounders and inclusion of non-fatal adverse cardiovascular events from hospital admission data, along with death registry data.
- Definition of prior MDD history was based on ICD-10 diagnostic criteria as opposed to a score on a questionnaire and our composite definition of hypertension incorporated past history, current medication and objective blood pressure measurements.
- Sample was adjusted for a broad range of baseline factors such as smoking status, BMI, psychotropic medication use and diabetes status amongst others, we were unable to see how these factors changed over the course of follow-up, or assess adherence to medication.
- Although trained nurses interviewed participants to obtain medical information for group assignment, as well as medication information, the self-reported nature may limit the accuracy of information.
- UK Biobank does have some issues with selection bias, as such those with more severe depression may be less likely to attend an assessment centre

For peer review only - http://bmjopen.bmj.com/site/about/guidelines.xhtml

INTRODUCTION

By 2030 major depressive disorder (MDD) and cardiovascular disease (CVD) will be the two leading causes of disability worldwide [1]. It is established that individuals with MDD are at increased risk of developing CVD and that they experience worse long-term outcomes, with higher mortality [2]. To date, studies investigating the association between MDD and CVD have tended to focus only on ischaemic heart disease, with the possibility of a specific association between MDD and hypertension relatively under-investigated [3].

Hypertension is extremely common (affecting 1 billion people worldwide)[4] and is responsible for 50% of all stroke and 50% of all ischaemic heart disease cases[5]. It is commonly comorbid with MDD, particularly in older age-groups [6 7]. Furthermore, a biological link between hypertension and MDD is supported by genome-wide association studies which have found that variants in calcium-channel genes, important in blood pressure control and hypertension[8], also act to increase risk for mood disorders such as MDD[9 10] and bipolar disorder[11 12].

Here we make use of prospective data from the UK Biobank cohort [13] to test the hypothesis that a history of MDD in individuals with hypertension (and no previous history of CVD) impacts adversely on cardiovascular outcomes. Given the very high global prevalence of MDD and hypertension [14], this is an important question for public health which could inform future treatment approaches for both hypertension and MDD.

METHODS

Study design

This was population cohort study using data from UK Biobank. Four mutually exclusive groups (hypertension only, MDD only, hypertension plus MDD, and a comparison group) were compared for adverse CVD outcomes, as well as stroke outcomes

For peer review only - http://bmjopen.bmj.com/site/about/guidelines.xhtml

Sample description

UK Biobank is a large population cohort of 502,655 participants recruited between April 2007 and July 2010 from 21 assessment centres located across Great Britain [13]. At baseline assessment participants completed a series of detailed assessments relating to lifestyle, current and past medical history and a range of physical health measurements, including body mass index (BMI) and blood pressure. UK Biobank was approved by the North West NHS Multi-Centre Research Ethics Committee and all participants provided written informed consent to participate. This analysis is part of UK Biobank approved application number 7155.

During the last two years of recruitment, questions relating to mood disorder features were added to the baseline assessment schedule. From the 172,729 participants who were assessed in this way, 134,860 provided sufficient responses to be included in our analysis. We excluded participants from our analyses based on the following *a priori* criteria: a history of bipolar disorder (n=1,831) or schizophrenia (n=262); where there were insufficient data provided by participants to clearly rule out MDD (n= 25,520) or hypertension (n=1,080); and where there were coding errors for date and/or time of death (n=4). Participants were further excluded from the adverse cardiovascular outcome if they had a record of CVD prior to recruitment (self-reported angina, myocardial infarction (MI) or stroke, or evidence of previous hospital admission for angina, MI or stroke) (n= 9,172). For the stroke outcome this exclusion was limited to a record of only stroke prior to admission (self-report or evidence of previous hospital admission for stroke) (n=2,280).

Classification of hypertension and MDD

Participants were defined as having hypertension if either: *a)* mean blood pressure at baseline was greater than clinically-defined criteria over two measurements (systolic blood pressure greater than or equal to 140 mmHg or diastolic blood pressure greater than or equal to 90 mmHg. Where only one reading was available this was used); or *b)* self-reported 'hypertension diagnosed by a doctor'

For peer review only - http://bmjopen.bmj.com/site/about/guidelines.xhtml

plus self-report of currently taking antihypertensive medication. This composite classification was used to ensure that undiagnosed hypertensive participants were not omitted from analyses and is in line with similar epidemiological studies[15-17]. According to these criteria, n=68,964 participants (51.1% of the sample) had hypertension for the adverse cardiovascular outcomes analysis and n=73,671 participants (52% of the sample) had hypertension in the stroke outcome analysis.

A past history of MDD was defined according to the criteria for mood disorders used in several previous studies with UK Biobank data[18 19]. Participants were classified as MDD if they reported at least one episode which comprised depression and/or anhedonia, lasting at least two weeks, plus had consulted with a general practitioner or psychiatrist for mental ill-health (n=28,027 adverse cardiovascular outcomes; n =29,528 stroke outcomes)[18].

For the adverse cardiovascular outcomes, the remainder of the sample, with no history of CVD, hypertension or MDD (n=50,798) were classified as a comparator group. The three mutually exclusive diagnostic groups for this study were therefore: hypertension only (n=56,035); MDD only (n=15,098) and hypertension plus MDD (n= 12,929). For the stroke outcomes, the remainder of the sample, with no history of stroke, hypertension or MDD (n=52,502) were classified as a comparator group. The three mutually exclusive diagnostic groups for this study were therefore: hypertension only (n=59,724); MDD only (n=15,581) and hypertension plus MDD (n= 13,947)

Outcomes

The primary outcome was defined as a new-onset cardiovascular event leading to hospital admission or death, specifically angina, MI, or chronic ischaemic heart disease (ICD-10 codes I20-I259), and transient ischaemic attack (TIA) or stroke (ICD-10 codes I60-69 and G45- G46). A secondary outcome was defined as stroke leading to hospital admission or death (ICD-10 codes I60-69 and G45- G46) [20]due to the strength of relationship hypertension has with this outcome in particular [5]. Hospital admission data were obtained from Hospital Episode Statistics in England, Patient Episode Database

For peer review only - http://bmjopen.bmj.com/site/about/guidelines.xhtml

for Wales and Scottish Morbidity Records in Scotland. Mortality outcomes were obtained from the National Health Service (NHS) Information Centre for England and Wales and from the NHS Central Register for Scotland. Individuals who died from a non-cardiovascular cause/stroke were censored at the time of death but not recorded as having an event. Admission data were available for Scottish, English and Welsh participants until 31 August 2014, 31 March 2015 and 28 February 2015 respectively. End of follow up was classified as these dates unless preceded by the date of death or the date of first cardiovascular admission. In total 3,685 (2.73%) participants had a new-onset cardiovascular event during the follow-up period and 910 (0.64%) participants had a new stroke event.

Confounding variables

Information on potential confounding factors was available for age, sex, socioeconomic status (Townsend score)[21], self-reported ethnicity, age of leaving full-time education, history of diabetes, body mass index (BMI), systolic blood pressure, history of hypercholesterolemia, alcohol use, smoking history, sedentary behaviour (number of hours each day spent sitting at a computer, television or driving), physical activity levels[22] and psychotropic medication use. Specific details on these variables are provided in supplementary digital content.

Analyses

Baseline characteristics were compared between groups using Chi-squared tests for categorical variables and Kruskal Wallis for continuous variables. Confounding variables were assessed for differences in adverse cardiovascular outcomes using log rank sums. For the four groups of interest (comparator group, hypertension only group, MDD only group and hypertension plus MDD group) we assessed associations with adverse cardiovascular outcomes using Cox proportional hazard regression and the Efron method for ties[23]. Models were applied in a staged process. Our findings are reported as unadjusted (model one), partially adjusted (model two) and fully adjusted (model

For peer review only - http://bmjopen.bmj.com/site/about/guidelines.xhtml

three). Model two adjusted for sociodemographic factors (age, sex, Townsend score, age of leaving full time education and ethnicity) and model three additionally adjusted for health and lifestyle factors (history of diabetes, history of hypercholesterolemia, BMI, smoking history, alcohol use, systolic blood pressure, sedentary hours per day, physical activity and psychotropic medication use). The assumption of proportionality of hazard was assessed for the four groups and each study covariate using Schoenfeld residuals [24].

We also assessed for evidence of an interaction between hypertension and MDD. The relative excess risk due to interaction (RERI)[25] was calculated to assess for additivity in the risk at each month where the proportionality assumption for the variables of interest was not met. All analyses were performed with Stata statistical software, version 12[26] with the exception of RERI which was calculated using the Microsoft Excel method of Andersson and colleagues, which allows for comparison of adjusted outcomes[27].

Psychotropic medication use was included as a confounding variable because of reports that these medications may increase risk of mortality [28] but we also conducted a sensitivity analysis which excluded the relatively small proportion of participants who were taking psychotropic medication. Sub-group analyses looking separately at hazard rates in male and female groups only was also carried out to assess for any gender specific differences a priori.

Time-varying covariates.

In the context of Schoenfeld residuals showing non-proportionality, models with time varying covariates were used. In addition, log (-log) plots were carried out to find the time point at which the proportionality assumption fails by viewing at which time-point the variable of interest crosses the control group. Following this, the data will be stratified by time on this this time point, effectively creating two separate survival analyses pre and post the failure time point.

Patient involvement

For peer review only - http://bmjopen.bmj.com/site/about/guidelines.xhtml

189 Although patients were not directly involved with the design of the specific research questions in this study, the hypotheses tested were developed in the context of experience from clinical practice that depression and hypertension may interact to impact on cardiovascular outcomes. UK Biobank has an active and ongoing programme of participant involvement: www.ukbiobank.ac.uk/participants/. The outcome measures used were those provided by the UK Biobank data collection protocol, the design of which had input from participants. UK Biobank also has a website and social media streams to disseminate research findings and they host an annual scientific meeting which includes cohort participants.

RESULTS

The final sample for adverse cardiovascular outcome included 134,860 participants followed for a median duration of 63 months (702,901.6 person-years follow-up). Table 1 describes the baseline characteristics of the four groups. In general, the hypertension only and comorbid hypertension plus MDD groups were older, had higher BMI and were more likely to have diabetes and hypercholesterolemia. The depression only and comorbid hypertension plus MDD groups had a higher proportion of women and were more likely to be current smokers (table 1). Gender-separated descriptive tables are shown in the supplementary digital content.

The sample for stroke-specific outcomes included 141,754 participants followed for a median duration of 63 months (735247.7 person-years follow-up). Table 2 describes the baseline characteristics of the four groups which display similar characteristics to the adverse cardiovascular outcome groups.

Adverse cardiovascular outcomes

For peer review only - http://bmjopen.bmj.com/site/about/guidelines.xhtml

Within the main analysis and the female only subgroup analysis, MDD failed the proportional hazards assumption as tested by Schoenfeld residuals. Table 3 presents unadjusted and multivariate-adjusted HRs for adverse cardiovascular outcomes across the groups. In the fully adjusted model, relative to the comparator group, the HR for adverse cardiovascular outcomes was significantly raised for the hypertension only group (HR=1. 36, 95%CI 1.22-1.52) and was higher still for the comorbid hypertension plus MDD group (HR=1.66, 95%CI 1.46-1.9) (sensitivity analysis HR=1.43, 95%CI 1.27-1.62; HR=1.72, 95%CI 1.49-1.999 respectively). Table 4 presents unadjusted and multivariate-adjusted HRs for adverse cardiovascular outcomes across the groups with the hypertension only group as comparator. In the fully adjusted model, relative to the hypertension group, the HR for adverse cardiovascular outcomes was significantly raised for the comorbid hypertension plus MDD group (HR=1.22, 95%CI 1.1-1.35). These findings were also robust to sensitivity analysis (HR= 1.20, 95%CI 1.08-1.34). An adjusted survival plot is shown in fig 1 and a survival analysis stratified by time is described and included within the supplementary digital content (table 9 in the supplementary digital content).

Within the sub-analysis, the model containing only the males showed a significant increase in hazard ratio for hypertension (male HR 1.29, 95% CI 1.13-1.47) (table 5 of the supplementary digital content) and comorbid MDD and hypertension (male HR 1.47, 95%CI 1.24-1.74). However, the difference between comorbid disease and hypertension only was not statistically significant (male HR 1.14, 95%CI 0.995-1.3). The female only sub-analysis showed an increase in hazard ratio for hypertension (female HR 1.64, 95%CI 1.33-2.02) and a greater increase in comorbid MDD and hypertension (female HR 2.18, 95%CI 1.82-2.92). The difference between comorbid disease and hypertension only was also statistically significant (female HR 1.33, 95%CI 1.14- 1.56). Sensitivity analysis supported these findings.

Relative excess risk due to interaction

For peer review only - http://bmjopen.bmj.com/site/about/guidelines.xhtml

There was evidence of an additive interaction between hypertension and MDD at baseline for the overall analysis before the 22.5 month time point (RERI=0.563, 95%CI 0.189 - 0.938). However after this time point there was no evidence of interaction. Table 11 in the supplementary digital content shows the full results for this analysis.

Stroke Outcomes

None of the independent variables for stroke outcome failed the proportionality assumption. Table 5 presents unadjusted and multivariate-adjusted HRs for stroke outcomes across the groups. In the fully adjusted model, relative to the comparator group, the HR for stroke was insignificantly raised for the hypertension only group (HR=1.21, 95%CI 0.97-1.51) and the depression only group (HR=1.20, 95%CI 0.89-1.63) but significantly raised for the comorbid hypertension plus MDD group (HR=1.37, 95%CI 1.04-1.79). In the hypertension comparator group, no group was significantly different from the hypertension only group (table 6). Similar trends were shown in the gender subset analysis but mainly not reaching significance. (Tables 7-8 in supplementary digital content) An adjusted survival plot is shown in figure 3. Again, all results were supported by sensitivity analysis.

DISCUSSION

In this large population cohort of middle-aged adults without CVD (adjusted for a broad range of confounders), individuals with co-morbid hypertension and MDD were at increased risk of an adverse cardiovascular event over time when compared to those with hypertension alone, depression alone and neither condition. There was some evidence of an additive effect between hypertension and MDD at baseline, but not throughout follow-up or within subgroup analyses. Differences between co-morbid hypertension and depression were more marked in females. For stroke outcomes, comorbid depression and hypertension was the only group that showed significantly increased hazard ratios.

For peer review only - http://bmjopen.bmj.com/site/about/guidelines.xhtml

259 **Previous research**

260 Our findings expand upon previous research from UK Biobank looking at cardiovascular diseases in
261 those with bipolar disorder and MDD[19]. It was found that there were significantly increased odds of
262 having 'any cardiovascular disease' (fully adjusted OR 1.15 CI 1.12–1.19) or hypertension (fully
263 adjusted OR 1.15 CI 1.13–1.18) if depressed, with an even higher odds for stroke (fully adjusted OR
264 1.26 CI 1.13–1.40). There are distinct differences between our current paper and the previous
265 publication. Follow-up data within UK-Biobank has been released to allow meaningful prospective
266 studies be conducted. Thus, the current paper has the benefits of using hospital records and death
267 certification for outcomes, rather than self-reported data. Within our current study we were able to
268 exclude those with previous self-declared and cardiovascular disease requiring hospital admission, as
269 previous studies show depression may result from cardiovascular disease [29 30] and worsen prognosis
270 [30]. As such, we are able to make inferences about the direction of effect. In addition, we have
271 incorporated hypertension to assess for some form of interaction.

272 Other survival analyses in hypertension/MDD comorbidity have focussed primarily on mortality
273 outcomes. Using data from the National Health and Nutrition Epidemiologic Follow-up Study in the
274 United States and the Taiwanese Survey of Health and Living Status, it has been found that
275 individuals with self-reported hypertension plus depressive symptoms (compared to a reference
276 group with neither) had increased all-cause mortality (HR=1.39, 95%CI 1.14-1.69, HR=1.54, 95%CI
277 1.29-1.83, respectively) [31 32] with the former also showing increased ischaemic heart disease –
278 specific mortality (HR=1.59, 95%CI 1.08-2.34)[31]. Similarly, Hamer and colleagues have reported a
279 prospective analysis of common mental disorder on mortality outcomes in individuals with
280 hypertension versus those without hypertension in participants from the Health Survey for England
281 and the Scottish Health Survey (1994–2004), finding that risk of CVD death was highest in the group
282 with comorbid hypertension and common mental disorder[15].

283 **Strengths**

For peer review only - http://bmjopen.bmj.com/site/about/guidelines.xhtml

284 These observations are broadly consistent with our results but our study has a number of
285 methodological advantages, including a very large sample size, adjustment of analyses for a more
286 comprehensive range of confounders, and a focus on new-onset non-fatal and fatal adverse
287 cardiovascular events. We also used a definition of prior MDD history which was based on
288 diagnostic criteria within ICD-10 (rather than a threshold score on a depressive symptoms or general
289 wellbeing scale) and our composite definition of hypertension incorporated past history, baseline
290 medication and blood pressure measurements.

291 **Limitations**

292 However, some limitations are acknowledged. Recruitment criteria for UK Biobank may lead to
293 selection bias for this form of study. Specifically, age-restrictions may lead to underrepresentation of
294 early-onset hypertension and those with more severe forms of MDD may be less inclined to attend
295 for assessment. We also acknowledge limitations with our classifications of MDD and hypertension,
296 which were primarily self-report rather than formal diagnostic assessments. Although we have
297 excluded prior cardiovascular events where possible, the MDD plus hypertension sub-type may
298 capture older individuals with a degree of vascular depression, which has an established association
299 with raised blood pressure[33]. In addition, although we adjust for a host of risk factors at baseline
300 such as smoking status, BMI and psychotropic medication, we are limited by the lack of follow-up
301 data which could show change and modification of said risk factors over time. Similarly we were
302 unable to assess for medication adherence and transitions from one investigatory group to another.
303 Such modifications could explain the non-proportional nature of the depression group, which may in
304 itself be a predictor of poor medication adherence[34]. Although adherence to medication was not
305 formally assessed, the number and duration of antihypertensive medications used in the
306 hypertension plus MDD group was the same as for the hypertension only group (supplementary
307 digital content, table 12). As such, worse outcomes in the MDD plus hypertension group are
308 not explained by less intensive antihypertensive treatment at baseline. The amelioration of the

For peer review only - http://bmjopen.bmj.com/site/about/guidelines.xhtml

Hazard ratios in the adjusted models suggests other covariates contribute considerably to the risk. This is important in the context of increased rates of diabetes, hypercholesterolemia and obesity along with lower socio-economic status in the hypertension only and comorbid groups and as such it is possible we may be seeing the summation of CV risk factors. Finally, the overall recruitment rate to UK Biobank was low (at around 6%), however, the large final cohort size, the depth and diversity of phenotype data collected at baseline and the wide sociodemographic representation of participants all make our findings highly relevant to UK primary care settings. While UK Biobank participants cannot be used to provide representative disease prevalence and incidence rates, valid assessment of exposure-disease relationships are nonetheless widely generalizable and do not require participants to be representative of the UK population at large[35], although findings will not be generalizable to other countries.

Possible mechanisms

Our finding that a history of MDD, in the context of a current diagnosis of hypertension, increased the risk of new-onset CVD could be explained by shared genetic and environmental risk factors[36]. Several genetic studies have found an association between the *CACNA1c* gene and MDD [10 37-39]. *CACNA1c* codes for a calcium channel which is integral to heart contraction and important for the normal functioning of the autonomic nervous system; cortisol release and immune function[40-42], systems that are central to the pathophysiology of both hypertension and depression[43-49]. Indeed, new evidence has emerged that subgroups of depression may have increased rates of genes related to cardiovascular risk factors such as cholesterol and raised blood pressure[50]. MDD could also increase cardiovascular disease risk via several mechanisms that may interact with blood pressure control[36]. For example, significant life events are stressors linked to depression by causing elevated cortisol leading to reduced serotonin and dopamine and increased sympathetic stimulation. This can lead to cardiac ischaemia via heart rate, blood pressure and vasoconstrictive changes, plaque formation due to endothelial injury, and impaired healing and thrombus formation via platelet

For peer review only - http://bmjopen.bmj.com/site/about/guidelines.xhtml

activation and haemostatic changes[36 51 52]. Individuals with hypertension or depression already have increased sympathetic activity which may be further increased in comorbid states[51]. Furthermore, MDD is also commonly associated with unhealthy lifestyle factors such as smoking, sedentary behaviour and poor diet and increased weight [19] and cardiovascular side effects of medications have been commonly reported. Given the finding that comorbid disease and hypertension lead to increased cardiovascular events, it would be useful to assess how treatment of these conditions influences outcome.

Potential menopausal effects are tempting explanations for the variation with time in the female only and overall analysis, especially given the age range of the cohort. It is widely accepted that oestrogen has a protective impact on the heart which may be lost at menopause[53]. Furthermore, increases in blood pressure are also noted at menopause [54] and depression may have a second incidence peak around this time too. [55 56] Due to the MDD only group generally being younger and having more females than the other groups, it may capture more of the menopausal change in cardiovascular hazard during follow up than other groups leading to disproportionate hazards. However, such findings are found in the female only analysis where age is similar between the comparator group and the MDD only group.

The disproportional hazards in the MDD only group leads to a trend of lowered hazard ratios at the start of follow up and a significantly increased hazard at the end of follow up. This lowered trend initially leads to a significant RERI finding which is not maintained. Of note, MDD correlates highly with neuroticism which has been shown to be inconsistent in regards to whether it is a risk factor or protective, including in UK Biobank. [57] It is thought that conscientiousness and poor self-reported health interact with neuroticism for better outcomes, it may be that premenopausal or perimenopausal states may influence survival positively in women[58]. Of further investigatory interest may be to assess if those with depression are more likely to develop hypertension during

For peer review only - http://bmjopen.bmj.com/site/about/guidelines.xhtml

For peer review only

menopause or indeed whether neuroticism and/or depression interacts with menopause to influence survival.

CONCLUSIONS

Overall, our findings may have important implications for routine clinical practice, particularly within primary care settings. Although evidence of an additive interaction is inconsistent, we found that comorbid hypertension and depression conferred greater hazard than hypertension alone for adverse cardiovascular outcomes. This significant finding remained after adjustment for factors such as BMI, smoking status and diabetes and was robust to sensitivity analysis excluding those on psychotropic medication. One possible implication is that clinicians should be more aware of the negative long-term impact on CVD outcomes caused by a history of MDD in the context of hypertension, particularly patients with no previous history of CVD. Although this work awaits replication and testing in other cohorts and settings, it may be that future iterations of CVD risk prediction tools, such as ASSIGN[59], would benefit from the addition of a question on whether individuals have a past history of MDD, so that they can be offered more intensive support to prevent CVD[60].

For peer review only

ACKNOWLEDGEMENTS

We are grateful to all participants of the UK Biobank cohort. UK Biobank was established by the Wellcome Trust, the Medical Research Council, Department of Health, Scottish Government and the Northwest Regional Development Agency. It has also had funding from the Welsh Assembly Government and the British Heart Foundation. UK Biobank is hosted by the University of Manchester and supported by the National Health Service (NHS). NG is supported by the Aitchison Family Clinical Research Fellowship at the University of Glasgow and DJS is supported by a Lister Institute Prize Fellowship. JC is supported by the Sackler Trust and the Wellcome Trust

Footnotes

Authors Statement: Contributors NG, JW, JP, JC, DS, SP and DM, contributed to study design and writing of the manuscript. JP and DM contributed to data acquisition. NG conducted data processing and statistical analyses.

Funding: Authors declare no support from any organisation for the submitted work;

Competing interests: All authors have completed the ICMJE uniform disclosure form at http://www.icmje.org/coi_disclosure.pdf and no financial relationships with any organisations that might have an interest in the submitted work in the previous three years; no other relationships or activities that could appear to have influenced the submitted work.

Ethics approval: This study has been conducted using UK Biobank data. UK Biobank has received ethics approval from the UK Biobank Research Ethics Committee (ref. 11/NW/0382).

Data sharing statement: The data used in this study are available via a direct application to UK Biobank.

For peer review only

Transparency statement: The lead author affirms that this manuscript is an honest, accurate and transparent account of the study being reported; that no important aspects of the study have been omitted; and that any discrepancies from the study as planned (and, if relevant, registered) have been explained.

COMPETING INTERESTS STATEMENTS

All authors have completed the ICMJE uniform disclosure form at http://www.icmje.org/coi_disclosure.pdf and no financial relationships with any organisations that might have an interest in the submitted work in the previous three years; no other relationships or activities that could appear to have influenced the submitted work.

For peer review only - http://bmjopen.bmj.com/site/about/guidelines.xhtml

References

1. Organization. WH. The global burden of disease: 2004 update. Geneva, Switzerland.: WHO press. 2008.

2. Hare DL, Toukhsati SR, Johansson P, et al. Depression and cardiovascular disease: a clinical review. *Eur Heart J* 2014;35(21):1365-72. doi: 10.1093/eurheartj/eht462

3. Cohen BE, Edmondson D, Kronish IM. State of the Art Review: Depression, Stress, Anxiety, and Cardiovascular Disease. *Am J Hypertens* 2015;28(11):1295-302. doi: 10.1093/ajh/hpv047

4. Kearney PM, Whelton M, Reynolds K, et al. Global burden of hypertension: analysis of worldwide data. *Lancet* 2005;365(9455):217-23. doi: 10.1016/S0140-6736(05)17741-1

5. Lawes CM, Vander Hoorn S, Rodgers A, et al. Global burden of blood-pressure-related disease, 2001. *Lancet* 2008;371(9623):1513-8. doi: 10.1016/S0140-6736(08)60655-8

6. Meng L, Chen D, Yang Y, et al. Depression increases the risk of hypertension incidence: a meta-analysis of prospective cohort studies. *J Hypertens* 2012;30:842 - 51.

7. Wu EL, Chien IC, Lin CH, et al. Increased risk of hypertension in patients with major depressive disorder: a population-based study. *J Psychosom Res* 2012;73(3):169-74. doi: 10.1016/j.jpsychores.2012.07.002

8. Johnson AD, Newton-Cheh C, Chasman DI, et al. Association of hypertension drug target genes with blood pressure and hypertension in 86,588 individuals. *Hypertension* 2011;57(5):903-10. doi: 10.1161/HYPERTENSIONAHA.110.158667

9. Casamassima F HJ, Fava M, Sachs GS, Smoller JW, Cassano GB, Lattanzi L, Fagerness J, Stange JP, Perlis RH. Phenotypic effects of a bipolar liability gene among individuals with major depressive disorder. . *Am J Med Genet B Neuropsychiatr Genet* 2010;153B:303-09.

10. Green EK, Grozeva D, Jones I, et al. The bipolar disorder risk allele at CACNA1C also confers risk of recurrent major depression and of schizophrenia. *Molecular Psychiatry* 2010;15(10):1016-22. doi: 10.1038/mp.2009.49

11. Ferreira MA, O'Donovan MC, Meng YA, et al. Collaborative genome-wide association analysis supports a role for ANK3 and CACNA1C in bipolar disorder. *Nat Genet* 2008;40(9):1056-8. doi: 10.1038/ng.209

12. Consortium WTCC. Identification of risk loci with shared effects on five major psychiatric disorders: a genome-wide analysis. *The Lancet* 2013;381(9875):1371-79.

13. Sudlow C, Gallacher J, Allen N, et al. UK biobank: an open access resource for identifying the causes of a wide range of complex diseases of middle and old age. *PLoS Med* 2015;12(3):e1001779. doi: 10.1371/journal.pmed.1001779

14. Ferrari AJ, Charlson FJ, Norman RE, et al. Burden of depressive disorders by country, sex, age, and year: findings from the global burden of disease study 2010. *PLoS Med* 2013;10(11):e1001547. doi: 10.1371/journal.pmed.1001547

15. Hamer M, Batty GD, Stamatakis E, et al. The combined influence of hypertension and common mental disorder on all-cause and cardiovascular disease mortality. *J Hypertens* 2010;28(12):2401-6. doi: 10.1097/HJH.0b013e32833e9d7c

16. Ong KL, Cheung BM, Man YB, et al. Prevalence, awareness, treatment, and control of hypertension among United States adults 1999-2004. *Hypertension* 2007;49(1):69-75. doi: 10.1161/01.hyp.0000252676.46043.18 [published Online First: 2006/12/13]

17. Egan BM, Zhao Y, Axon RN. US trends in prevalence, awareness, treatment, and control of hypertension, 1988-2008. *Jama* 2010;303(20):2043-50. doi: 10.1001/jama.2010.650 [published Online First: 2010/05/27]

18. Smith DJ, Nicholl BI, Cullen B, et al. Prevalence and characteristics of probable major depression and bipolar disorder within UK biobank: cross-sectional study of 172,751 participants. *PLoS One* 2013;8(11):e75362. doi: 10.1371/journal.pone.0075362

For peer review only

For peer review only - http://bmjopen.bmj.com/site/about/guidelines.xhtml

19. Martin DJ, Ul-Haq Z, Nicholl BI, et al. Cardiometabolic disease and features of depression and bipolar disorder: population-based, cross-sectional study. *Br J Psychiatry* 2016;208(4):343-51. doi: 10.1192/bjp.bp.114.157784
20. Woodfield R, Grant I, Group UKBSO, et al. Accuracy of Electronic Health Record Data for Identifying Stroke Cases in Large-Scale Epidemiological Studies: A Systematic Review from the UK Biobank Stroke Outcomes Group. *PLOS ONE* 2015;10(10):e0140533. doi: 10.1371/journal.pone.0140533
21. Townsend P. Deprivation. *Journal of Social Policy* 2009;16(02):125. doi: 10.1017/s0047279400020341
22. Guo W, Bradbury KE, Reeves GK, et al. Physical activity in relation to body size and composition in women in UK Biobank. *Annals of Epidemiology* 2015;25(6):406-13.e6. doi: http://dx.doi.org/10.1016/j.annepidem.2015.01.015
23. Efron B. The Efficiency of Cox's Likelihood Function for Censored Data. *Journal of the American Statistical Association,* 1977;72(359):557-65.
24. Schoenfeld D. Partial Residuals for the Proportional Hazards Regression-Model. *Biometrika* 1982;69(1):239-41. doi: Doi 10.2307/2335876
25. Hosmer DW, Lemeshow S. Confidence interval estimation of interaction. *Epidemiology (Cambridge, Mass)* 1992;3(5):452-6. [published Online First: 1992/09/01]
26. Stata Statistical Software, version 12 [program]. College station, Texas.
27. Andersson T, Alfredsson L, Kallberg H, et al. Calculating measures of biological interaction. *European journal of epidemiology* 2005;20(7):575-9. [published Online First: 2005/08/27]
28. Weich S, Pearce HL, Croft P, et al. Effect of anxiolytic and hypnotic drug prescriptions on mortality hazards: retrospective cohort study. *BMJ* 2014;348:g1996. doi: 10.1136/bmj.g1996
29. Kang HJ, Stewart R, Bae KY, et al. Predictive value of homocysteine for depression after acute coronary syndrome. *Oncotarget* 2016;7(42):69032-40. doi: 10.18632/oncotarget.11966
30. Lichtman JH, Froelicher ES, Blumenthal JA, et al. Depression as a risk factor for poor prognosis among patients with acute coronary syndrome: systematic review and recommendations: a scientific statement from the American Heart Association. *Circulation* 2014;129(12):1350-69. doi: 10.1161/CIR.0000000000000019
31. Axon RN, Zhao Y, Egede LE. Association of depressive symptoms with all-cause and ischemic heart disease mortality in adults with self-reported hypertension. *Am J Hypertens* 2010;23(1):30-7. doi: 10.1038/ajh.2009.199
32. Kuo PL, Pu C. The contribution of depression to mortality among elderly with self-reported hypertension: analysis using a national representative longitudinal survey. *J Hypertens* 2011;29(11):2084-90. doi: 10.1097/HJH.0b013e32834b59ad [published Online First: 2011/09/22]
33. Aizenstein HJ, Baskys A, Boldrini M, et al. Vascular depression consensus report – a critical update. *BMC Medicine* 2016;14(1):161. doi: 10.1186/s12916-016-0720-5
34. Goldstein CM, Gathright EC, Garcia S. Relationship between depression and medication adherence in cardiovascular disease: the perfect challenge for the integrated care team. *Patient preference and adherence* 2017;11:547-59. doi: 10.2147/PPA.S127277
35. Fry A, Littlejohns TJ, Sudlow C, et al. Comparison of Sociodemographic and Health-Related Characteristics of UK Biobank Participants With Those of the General Population. *American journal of epidemiology* 2017;186(9):1026-34. doi: 10.1093/aje/kwx246 [published Online First: 2017/06/24]
36. Nemeroff CB, Goldschmidt-Clermont PJ. Heartache and heartbreak - the link between depression and cardiovascular disease. *Nat Rev Cardiol* 2012;9(9):526-39.
37. He K, An Z, Wang Q, et al. CACNA1C, schizophrenia and major depressive disorder in the Han Chinese population. *British Journal of Psychiatry* 2014;204(1):36-39. doi: 10.1192/bjp.bp.113.126979

For peer review only - http://bmjopen.bmj.com/site/about/guidelines.xhtml

38. Liu Y, Blackwood DH, Caesar S, et al. Meta-Analysis of Genome-Wide Association Data of Bipolar Disorder and Major Depressive Disorder. *Molecular psychiatry* 2011;16(1):10.1038/mp.2009.107. doi: 10.1038/mp.2009.107
39. Sullivan PF, De Geus EJC, Willemsen G, et al. Genome-wide association for major depressive disorder: A possible role for the presynaptic protein piccolo. *Molecular Psychiatry* 2009;14(4):359-75. doi: 10.1038/mp.2008.125
40. Robert V, Triffaux E, Savignac M, et al. Singularities of calcium signaling in effector T-lymphocytes. *Biochimica et Biophysica Acta (BBA) - Molecular Cell Research* 2013;1833(7):1595-602. doi: http://dx.doi.org/10.1016/j.bbamcr.2012.12.001
41. Nohara LL, Stanwood SR, Omilusik KD, et al. Tweeters, Woofers and Horns: The Complex Orchestration of Calcium Currents in T Lymphocytes. *Frontiers in immunology* 2015;6:234. doi: 10.3389/fimmu.2015.00234 [published Online First: 2015/06/09]
42. Loechner KJ, Salmon WC, Fu J, et al. Cell Cycle-Dependent Localization of Voltage-Dependent Calcium Channels and the Mitotic Apparatus in a Neuroendocrine Cell Line(AtT-20). *Int J Cell Biol* 2009;2009:487959. doi: 10.1155/2009/487959
43. Dantzer R, O'Connor JC, Freund GG, et al. From inflammation to sickness and depression: when the immune system subjugates the brain. *Nat Rev Neurosci* 2008;9(1):46-56. doi: 10.1038/nrn2297
44. Abboud FM, Harwani SC, Chapleau MW. Autonomic neural regulation of the immune system: implications for hypertension and cardiovascular disease. *Hypertension* 2012;59(4):755-62. doi: 10.1161/HYPERTENSIONAHA.111.186833
45. Schiffrin EL. The immune system: role in hypertension. *The Canadian journal of cardiology* 2013;29(5):543-8. doi: 10.1016/j.cjca.2012.06.009 [published Online First: 2012/08/21]
46. Mancia G, Grassi G. The autonomic nervous system and hypertension. *Circ Res* 2014;114(11):1804-14. doi: 10.1161/CIRCRESAHA.114.302524
47. Carney RM, Freedland KE, Veith RC. Depression, the autonomic nervous system, and coronary heart disease. *Psychosomatic medicine* 2005;67 Suppl 1:S29-33. doi: 10.1097/01.psy.0000162254.61556.d5 [published Online First: 2005/06/15]
48. Baune BT, Stuart M, Gilmour A, et al. The relationship between subtypes of depression and cardiovascular disease: a systematic review of biological models. *Transl Psychiatry* 2012;2(3):e92. doi: 10.1038/tp.2012.18
49. Pariante CM, Lightman SL. The HPA axis in major depression: classical theories and new developments. *Trends Neurosci* 2008;31(9):464-8. doi: 10.1016/j.tins.2008.06.006
50. Howard DM, Clarke T-K, Adams MJ, et al. The Stratification Of Major Depressive Disorder Into Genetic Subgroups. *bioRxiv* 2017 doi: 10.1101/134601
51. Barton DA, Dawood T, Lambert EA, et al. Sympathetic activity in major depressive disorder: identifying those at increased cardiac risk? *J Hypertens* 2007;25(10):2117-24. doi: 10.1097/HJH.0b013e32829baae7 [published Online First: 2007/09/22]
52. Gouin J-P, Kiecolt-Glaser JK. The Impact of Psychological Stress on Wound Healing: Methods and Mechanisms. *Immunology and allergy clinics of North America* 2011;31(1):81-93. doi: 10.1016/j.iac.2010.09.010
53. Rosano GM, Panina G. Oestrogens and the heart. *Therapie* 1999;54(3):381-5. [published Online First: 1999/09/29]
54. Zanchetti A, Facchetti R, Cesana GC, et al. Menopause-related blood pressure increase and its relationship to age and body mass index: the SIMONA epidemiological study. *J Hypertens* 2005;23(12):2269-76. [published Online First: 2005/11/05]
55. Eaton WW, Anthony JC, Gallo J, et al. Natural history of diagnostic interview schedule/ dsm-iv major depression: The baltimore epidemiologic catchment area follow-up. *Archives of General Psychiatry* 1997;54(11):993-99. doi: 10.1001/archpsyc.1997.01830230023003

For peer review only - http://bmjopen.bmj.com/site/about/guidelines.xhtml

56. Blazer DG, Kessler RC, McGonagle KA, et al. The prevalence and distribution of major depression in a national community sample: the National Comorbidity Survey. *Am J Psychiatry* 1994;151(7):979-86. doi: 10.1176/ajp.151.7.979 [published Online First: 1994/07/01]
57. Gale CR, Čukić I, Batty GD, et al. When Is Higher Neuroticism Protective Against Death? Findings From UK Biobank. *Psychological Science* 2017;28(9):1345-57. doi: 10.1177/0956797617709813
58. Ploubidis GB, Grundy E. Personality and all cause mortality: Evidence for indirect links. *Personality and Individual Differences* 2009;47(3):203-08. doi: https://doi.org/10.1016/j.paid.2009.02.022
59. Woodward M, Brindle P, Tunstall-Pedoe H. Adding social deprivation and family history to cardiovascular risk assessment: the ASSIGN score from the Scottish Heart Health Extended Cohort (SHHEC). *Heart* 2007;93(2):172-76. doi: 10.1136/hrt.2006.108167
60. Hippisley-Cox J, Coupland C, Vinogradova Y, et al. Predicting cardiovascular risk in England and Wales: prospective derivation and validation of QRISK2. *BMJ* 2008;336(7659):1475-82. doi: 10.1136/bmj.39609.449676.25

For peer review only

For peer review only - http://bmjopen.bmj.com/site/about/guidelines.xhtml

570 **Table 1. Baseline characteristics for adverse cardiovascular outcomes**

	Comparator group		Hypertension only		MDD only		Hypertension plus MDD	
	N = 50798		N = 56035		N = 15098		N = 12929	
**Median age (range)***	54	(47 - 61)	61	(55 - 65)	53	(46 - 60)	60	(53 - 64)
**Females, N (%)**	29228	(57.54%)	25893	(46.21%)	10929	(72.39%)	7676	(59.37%)
**Ethnicity, N (%)**								
White	46147	(90.84%)	51249	(91.46%)	14247	(94.36%)	12272	(94.92%)
Asian/Asian British	1771	(3.49%)	1696	(3.03%)	261	(1.73%)	179	(1.38%)
Black/ Black British	1323	(2.6%)	1769	(3.16%)	219	(1.45%)	222	(1.72%)
**Median Townsend score (range)***	-1.89	(-3.45 - 0.54)	-2.07	(-3.51 - 0.39)	-1.64	(-3.3 - 0.93)	-1.84	(-3.42 - 0.76)
**Age at leaving full-time education, N (%)**								
<16	5916	(11.65%)	12085	(21.57%)	1725	(11.43%)	2607	(20.16%)
16	10265	(20.21%)	11827	(21.11%)	3178	(21.05%)	2732	(21.13%)
>16	34090	(67.11%)	31480	(56.18%)	10090	(66.83%)	7503	(58.03%)
**Total physical activity in metabolic**	3.97	(1.68 - 8.03)	3.79	(1.51 - 8.03)	3.89	(1.66 - 8)	3.68	(1.49 - 7.95)
**Sedentary time in hours, median (range)***	4	(3 - 6)	4.5	(3.5 - 6)	4.5	(3 - 6)	5	(3.5 - 6)

For peer review only - http://bmjopen.bmj.com/site/about/guidelines.xhtml

**Diabetes, N (%)**	1268	(2.5%)	3777	(6.74%)	380	(2.52%)	929	(7.19%)
**Hypercholesterolaemia, N (%)**	3011	(5.93%)	9210	(16.44%)	893	(5.91%)	2211	(17.1%)
**Systolic BP in mmHg, median (range)***	125.5	(118 - 132)	149.5	(142 - 159.5)	124	(116 - 131)	147.5	(140.5 - 157.)
**Body Mass Index, N (%)**								
<18.5	389	(0.77%)	142	(0.25%)	103	(0.68%)	34	(0.26%)
18.5 – 25	22549	(44.39%)	13678	(24.41%)	6251	(41.4%)	2874	(22.23%)
25-30	20410	(40.18%)	25216	(45 %)	5936	(39.32%)	5389	(41.68%)
>30	7450	(14.67%)	16999	(30.34%)	2808	(18.6%)	4632	(35.83%)
**Smoking status, N (%)**								
Never smoked	30626	(60.29%)	31503	(56.22%)	7864	(52.09%)	6454	(49.92%)
Previously smoked	15056	(29.64%)	20140	(35.94%)	5118	(33.9%)	5065	(39.18%)
Current smoker	4970	(9.78%)	4199	(7.49%)	2093	(13.86%)	1381	(10.68%)
**Alcohol frequency, N (%)**								
Daily or almost daily	9450	(18.6%)	12970	(23.15%)	2736	(18.12%)	2881	(22.28%)
Three or four times a week	12175	(23.97%)	13033	(23.26%)	3253	(21.55%)	2837	(21.94%)
Once or twice a week	13644	(26.86%)	13889	(24.79%)	3880	(25.7%)	2916	(22.55%)

For peer review only - http://bmjopen.bmj.com/site/about/guidelines.xhtml

One to three times a month	6052	(11.91%)	5588	(9.97%)	2058	(13.63%)	1512	(11.69%)
Special occasions only	5534	(10.89%)	6330	(11.3%)	1904	(12.61%)	1729	(13.37%)
Never	3924	(7.72%)	4199	(7.49%)	1262	(8.36%)	1048	(8.11%)
**Psychotropic medication, N (%)**	1341	(2.64%)	1795	(3.2%)	2844	(18.84%)	2522	(19.51%)

*Data presented as N (%) except * which are median values (interquartile range). Data presented as MET-hrs (hours spent doing exercise adjusted for multiples of basal metabolic rate in accordance with IPAQ).*

For peer review only

Table 2 Baseline characteristics for stroke outcomes

	Comparator group		Hypertension only		MDD only		Hypertension plus MDD	
	N = 52502		N = 59724		N = 15581		N = 13947	
**Median age (range)***	54	(47 - 61)	61	(55 - 65)	54	(47 - 61)	60	(53 - 64)
**Females, N (%)**	29684	(56.54%)	26937	(45.1%)	11143	(71.52%)	8090	(58.01%)
**Ethnicity, N (%)**								
White	47697	(90.85%)	54578	(91.38%)	14697	(94.33%)	13212	(94.73%)
Asian/Asian British	1857	(3.54%)	1889	(3.16%)	280	(1.8%)	209	(1.5%)
Black/ Black British	1355	(2.58%)	1854	(3.1%)	223	(1.43%)	246	(1.76%)
**Median Townsend score (range)***	-1.89	(-3.45 - 0.55)	-2.04	(-3.49 - 0.44)	-1.56	(-3.28 - 1.15)	-1.74	(-3.4 - 0.93)
**Age at leaving full-time education, N (%)**								
<16	6446	(12.28%)	13396	(22.43%)	1884	(12.09%)	2945	(21.12%)
16	10590	(20.17%)	12507	(20.94%)	3270	(20.99%)	2953	(21.17%)
>16	34914	(66.5%)	33114	(55.45%)	10317	(66.22%)	7947	(56.98%)
**Total physical activity in metabolic**	3.96	(1.67 - 8.02)	3.75	(1.5 - 8)	4.13	(1.67 - 8.36)	3.66	(1.45 - 7.83)
**Sedentary time in hours, median (range)***	4	(3 - 6)	5	(3.5 - 6)	5	(3.5 - 6.5)	5	(4 - 7)

For peer review only - http://bmjopen.bmj.com/site/about/guidelines.xhtml

**Diabetes, N (%)**	1454	(2.77%)	4502	(7.54%)	449	(2.88%)	1163	(8.34%)
**Hypercholesterolaemia, N (%)**	3592	(6.84%)	10768	(18.03%)	1049	(6.73%)	2620	(18.79%)
**Systolic BP in mmHg, median (range)***	125.5	(118 - 132)	149.5	(142 - 159.5)	127	(120.5 - 133)	147.5	(140.5 - 156.5)
**Body Mass Index, N (%)**								
<18.5	395	(0.75%)	151	(0.25%)	104	(0.67%)	38	(0.27%)
18.5 – 25	22967	(43.75%)	14242	(23.85%)	6374	(40.91%)	3017	(21.63%)
25-30	21185	(40.35%)	26817	(44.9%)	6149	(39.46%)	5769	(41.36%)
>30	7953	(15.15%)	18514	(31.%)	2954	(18.96%)	5123	(36.73%)
**Smoking status, N (%)**								
Never smoked	31318	(59.65%)	32982	(55.22%)	8052	(51.68%)	6834	(49%)
Previously smoked	15851	(30.19%)	22019	(36.87%)	5340	(34.27%)	5560	(39.87%)
Current smoker	5170	(9.85%)	4501	(7.54%)	2163	(13.88%)	1519	(10.89%)
**Alcohol frequency, N (%)**								
Daily or almost daily	9760	(18.59%)	13751	(23.02%)	2817	(18.08%)	3085	(22.12%)
Three or four times a week	12563	(23.93%)	13827	(23.15%)	3335	(21.4%)	3020	(21.65%)
Once or twice a week	14089	(26.84%)	14719	(24.65%)	3993	(25.63%)	3125	(22.41%)

For peer review only - http://bmjopen.bmj.com/site/about/guidelines.xhtml

One to three times a month	6220	(11.85%)	5971	(10%)	2122	(13.62%)	1627	(11.67%)
Special occasions only	5744	(10.94%)	6794	(11.38%)	1978	(12.69%)	1885	(13.52%)
Never	4102	(7.81%)	4630	(7.75%)	1330	(8.54%)	1199	(8.6%)
**Psychotropic medication, N (%)**	1408	(2.68%)	1996	(3.34%)	2976	(19.1%)	2778	(19.92%)

575 *Data presented as N (%) except * which are median values (interquartile range). Data presented as MET-hrs (hours spent doing exercise adjusted for*
576 *multiples of basal metabolic rate in accordance with IPAQ).*

For peer review only - http://bmjopen.bmj.com/site/about/guidelines.xhtml

Table 3: Risk of adverse cardiovascular event by clinical group: unadjusted, partially adjusted and fully adjusted models.

	Unadjusted				Model 1 - Sociodemographic				Model 2 - Model 1 + Health/ Lifestyle			
**Group**	H.R.	95% C.I.		p-value	H.R.	95% C.I.		p-value	H.R.	95% C.I.		p-value
No Hypertension - No MDD	1(ref)				1(ref)				1(ref)			
Hypertension only	2.60	(2.39	- 2.82)	$3.31x10^{-113}$	1.72	(1.57	- 1.88)	$1.99x10^{-33}$	1.36	(1.22	- 1.52)	$2.92x10^{-8}$
MDD only	0.69	(0.51	- 0.94)	0.02	0.82	(0.6	- 1.13)	0.23	0.75	(0.54	- 1.04)	0.08
Hypertension and MDD	2.84	(2.55	- 3.17)	$6.31x10^{-77}$	2.27	(2.02	- 2.55)	$2.75x10^{-44}$	1.66	(1.45	- 1.9)	$7.48x10^{-14}$
**Time varying Variables**												
MDD only	1.01	(1.004	- 1.02)	$2.38x10^{-3}$	1.01	(1.004	- 1.02)	$3.19x10^{-3}$	1.01	(1.004	- 1.02)	$3.03x10^{-3}$

**Adjusted for sociodemographic factors (age, sex, Townsend score, age of leaving full time education and ethnicity. †Additionally adjusted for history of diabetes, history of hypercholesterolemia, BMI, smoking history, alcohol use, systolic blood pressure, sedentary hours per day, physical activity and psychotropic medication use. MDD = Major depressive disorder, HR = Hazard ratio, C.I. = Confidence interval.*

For peer review only

For peer review only - http://bmjopen.bmj.com/site/about/guidelines.xhtml

Table 4: Risk of adverse cardiovascular event by clinical group: unadjusted, partially adjusted and fully adjusted models with hypertension as the comparator

	Unadjusted			Model 1 - Sociodemographic			Model 2 - Model 1 + Health/ Lifestyle		
**Group**	H.R.	95% C.I.	p-value	H.R.	95% C.I.	p-value	H.R.	95% C.I.	p-value
Hypertension only	1(ref)			1(ref)			1(ref)		
No Hypertension - No MDD	0.38	(0.35 - 0.42)	3.31x10^{-113}	0.58	(0.53 - 0.63)	1.99x10^{-33}	0.73	(0.66 - 0.82)	2.92x10^{-8}
MDD only	0.27	(0.2 - 0.36)	1.14x10^{-17}	0.48	(0.35 - 0.66)	4.91x10^{-6}	0.55	(0.4 - 0.76)	3.23x10^{-4}
Hypertension and MDD	1.09	(0.996 - 1.2)	0.06	1.32	(1.2 - 1.46)	3.07x10^{-8}	1.22	(1.1 - 1.35)	1.30x10^{-4}
**Time varying Variables**									
MDD only	1.01	(1.004 - 1.02)	0.002	1.01	(1.004 - 1.02)	3.19x10^{-3}	1.01	(1.004 - 1.02)	3.03x10^{-3}

**Adjusted for sociodemographic factors (age, sex, Townsend score, age of leaving full time education and ethnicity. †Additionally adjusted for history of diabetes, history of hypercholesterolemia, BMI, smoking history, alcohol use, systolic blood pressure, sedentary hours per day, physical activity and psychotropic medication use. MDD = Major depressive disorder, HR = Hazard ratio, C.I. = Confidence interval.*

For peer review only - http://bmjopen.bmj.com/site/about/guidelines.xhtml

589 Table 5: Risk of stroke event by clinical group: unadjusted, partially adjusted and fully adjusted models.

	Unadjusted			Model 1 - Sociodemographic			Model 2 - Model 1 + Health/ Lifestyle		
**Group**	H.R.	95% C.I.	p-value	H.R.	95% C.I.	p-value	H.R.	95% C.I.	p-value
No Hypertension- No MDD	1(ref)			1(ref)			1(ref)		
Hypertension only	2.55	(2.16 - 3.02)	3.84×10^{-28}	1.64	(1.38 - 1.96)	3.35×10^{-8}	1.21	(0.97 - 1.51)	0.09
MDD only	1.14	(0.86 - 1.52)	0.37	1.37	(1.02 - 1.84)	0.037	1.20	(0.89 - 1.63)	0.24
Hypertension and MDD	2.67	(2.13 - 3.34)	9.79×10^{-18}	2.05	(1.63 - 2.58)	1.08×10^{-9}	1.37	(1.04 - 1.79)	0.02

590 **Adjusted for sociodemographic factors (age, sex, Townsend score, age of leaving full time education and ethnicity.* [†]*Additionally adjusted for history of*
591 *diabetes, history of hypercholesterolemia, BMI, smoking history, alcohol use, systolic blood pressure, sedentary hours per day, physical activity and*
592 *psychotropic medication use. MDD = Major depressive disorder, HR = Hazard ratio, C.I. = Confidence interval.*

593

594

For peer review only - http://bmjopen.bmj.com/site/about/guidelines.xhtml

Table 6: Risk of stroke event by clinical group: unadjusted, partially adjusted and fully adjusted models with hypertension as the comparator

	Unadjusted			Model 1 - Sociodemographic			Model 2 - Model 1 + Health/ Lifestyle		
**Group**	H.R.	95% C.I.	p-value	H.R.	95% C.I.	p-value	H.R.	95% C.I.	p-value
Hypertension only	1(ref)			1(ref)			1(ref)		
No Hypertension - No MDD	0.39	(0.33 - 0.46)	3.84×10^{-28}	0.61	(0.51 - 0.73)	3.35×10^{-8}	0.82	(0.66 - 1.03)	0.09
MDD only	0.45	(0.34 - 0.58)	1.43×10^{-9}	0.83	(0.63 - 1.1)	0.19	0.99	(0.73 - 1.35)	0.95
Hypertension and MDD	1.05	(0.86 - 1.27)	0.64	1.25	(1.03 - 1.52)	0.03	1.13	(0.92 - 1.39)	0.26

**Adjusted for sociodemographic factors (age, sex, Townsend score, age of leaving full time education and ethnicity. †Additionally adjusted for history of diabetes, history of hypercholesterolemia, BMI, smoking history, alcohol use, systolic blood pressure, sedentary hours per day, physical activity and psychotropic medication use. MDD = Major depressive disorder, HR = Hazard ratio, C.I. = Confidence interval.*

For peer review only - http://bmjopen.bmj.com/site/about/guidelines.xhtml

Fig1. Adjusted survival analysis graph for adverse cardiovascular outcome.

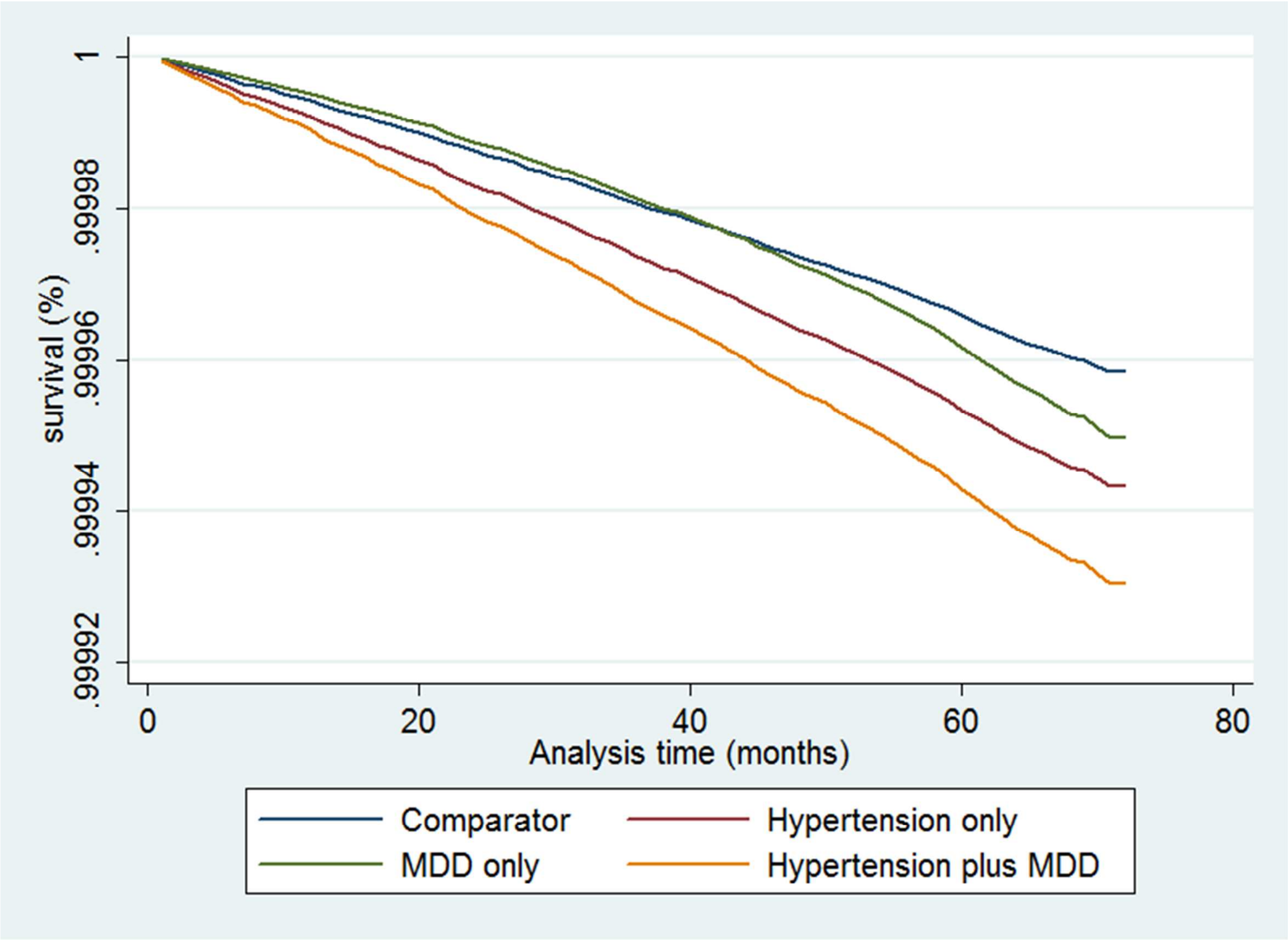

Fig 2. Log (-log) plot showing non-proportionality of MDD only survival over time. Paths between the comparator group and the MDD group appear to cross at the 22.5 month mark.

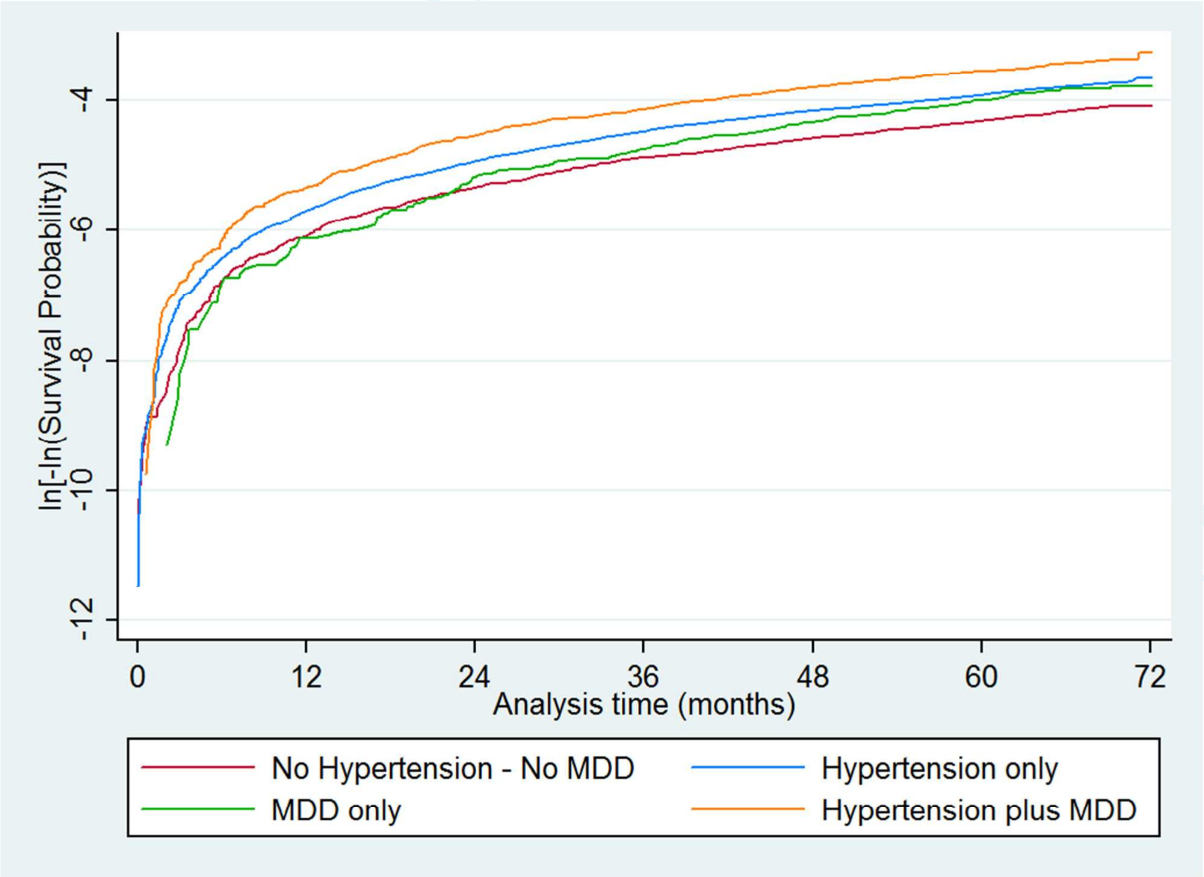

For peer review only - http://bmjopen.bmj.com/site/about/guidelines.xhtml

Fig 3. Adjusted survival analysis graph for stroke outcomes.

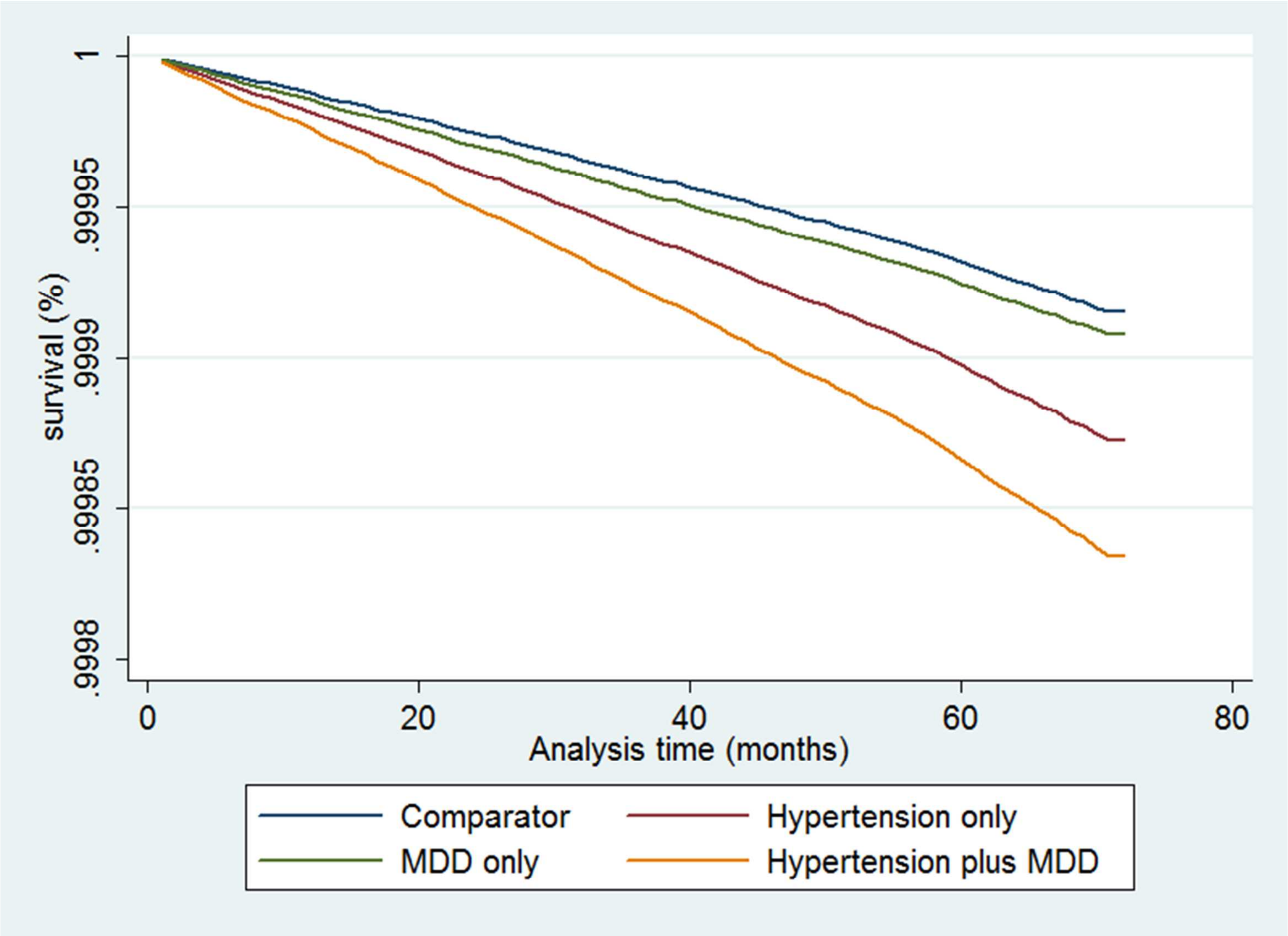

Supplemental Digital Content 1.doc

For peer review only

Supplementary information for Impact of major depression on cardiovascular outcomes for individuals with hypertension: prospective study in UK Biobank. Graham et al

METHODS

New-onset cardiovascular outcomes

Date and cause of death were obtained from death certificates held by the National Health Service (NHS) Information Centre for participants from England and Wales and the NHS Central Register Scotland for participants from Scotland. Date and cause of hospital admissions were identified via record linkage to Health Episode Statistics (HES) records for England, the Patient Episode Database for Wales (PEDW) and to the Scottish Morbidity Records (SMR) for Scotland. Detailed information about the record linkage procedure is available online [1 2]. At the time of analysis, mortality data were available up to 31st January 2016 for England and Wales and 11th November 2015 for Scotland. Hospital admission data were available for the Scottish, English and Welsh participants until the 31st August 2014, 31st March 2015, and 28th February 2015 respectively. Therefore, for new cardiovascular events, end of follow up was classified as the hospital admission dates unless preceded by the date of death or the date of first cardiovascular event. New onset cardiovascular events were defined as an ICD 10 code of G45, G46, I20- I25, or I6 recorded on a death certificate or hospital admission. Deaths that predated the assessment date were excluded from analysis as presumed errors as were those in which data had only recorded a death date but no cause of death or a cause of death but no death date. Participants that had hospital admissions prior to the assessment date due to the aforementioned ICD10 codes were excluded as were not first episode. In addition, ICD-9 codes 430-438, 410-414, 429 and

For peer review only - http://bmjopen.bmj.com/site/about/guidelines.xhtml

429.2 were also excluded. hospital records are not available for the entire lifetime of study individuals, potentially missing some early cardiovascular events, as such those with self-declared prior cardiovascular disease at baseline were also excluded.

Blood Pressure

Blood pressure was measured in a sitting position partway through the interview and at the end of the interview using a digital blood pressure monitor (Omron HEM-7015IT.). Full protocol is available online https://biobank.ctsu.ox.ac.uk/crystal/docs/Bloodpressure.pdf

Physical activity

Physical activity was based on self-report, utilising the short form International Physical Activity Questionnaire (IPAQ). Participants reported the frequency and duration of moderate and vigorous activity along with walking undertaken in a typical week [3]. Data were analysed in accordance with the IPAQ scoring protocol [4] and total physical activity was computed as the sum of walking, moderate and vigorous activity, measured as metabolic equivalents (MET-hours/week). Physical activity was used in analyses as a continuous variable. Participants who reported greater than 24 hours a day doing all activity were classified as missing.

Sedentary behaviour

Sedentary behaviour duration was derived from the sum of self-reported time spent driving, using computer and watching television. Those stating that they had performed "less than an hour" of sedentary activities were coded as 0.5hrs to allow use of a continuous variable.

For peer review only - http://bmjopen.bmj.com/site/about/guidelines.xhtml

Participants who reported greater than 24 hours a day doing all activity were classified as missing.

Socio-demographic and other covariates

Self-report on taking antihypertensive medication was taken from a question specific to cardiovascular medications, where antihypertensive medication was an option to respond. Area-based socioeconomic status was derived from postcode of residence, utilising the census-derived Townsend deprivation index scored on housing, employment, social class and car availability where a negative score represents greater affluence [5 6]. Age was calculated from dates of birth and baseline assessment date. Smoking status was categorised into never, former and current smoking based on self-report, those who wished not to answer were coded as missing. Drink frequency was categorised into daily, three or four times a week, once or twice a week, one to three times a month, special occasions only, and never based on self-report. Those who wished not to answer were coded as missing. Medical history of diabetes and high cholesterol was collected from the self-completed, baseline assessment questionnaire of medical conditions. Ethnicity was categorised as Caucasian, black/mixed and Asian/mixed based on self-report. Other ethnicities coded as missing due to small numbers. Age at completing full-time education was categorised as (<16, 16, >16). Height and body weight were measured by trained nurses during the initial assessment centre visit. Body mass index (BMI) was calculated as (weight/height^2) and the WHO criteria[7] to classify BMI into: underweight <18.5, normal weight 18.5-24.9, overweight 25.0-29.9 and obese ≥30.0 kg.m^{-2}. Psychotropic medication use was defined by the presence of pharmaceuticals from British National Formulary (BNF) chapters 4.1.1 to 4.3.4 [8] on self-report medication lists at baseline. Duration of hypertension

For peer review only - http://bmjopen.bmj.com/site/about/guidelines.xhtml

was calculated utilising age and age of hypertension diagnosis. Antihypertensive medication count was calculated as the absolute number of ACE inhibitors, angiotensin II receptor antagonists, calcium channel blockers, beta-blockers and thiazide diuretics prescribed to an individual. Generic medication names were sought and cross-referenced with the BNF chapters 2.2.1, 2.4, 2.5.5 and 2.6.2 [8].

Statistical analysis:

A best-fit multivariable regression spline model (stata command “mvrs”) was used to find the best model to adjust for non-linear covariates. For the adverse cardiovascular outcomes, A single knot was fitted for age at age 50 and two knots were fitted for total physical activity at 1.65 and 8.062 metabolic equivalent hours. In the male subgroup analysis two knots were fitted for total physical activity at 1.7 and 8.507 metabolic equivalent hours, in the female subgroup two knots were fitted for total physical activity at 1.57 and 3.75 and two knots were fitted at systolic blood pressure 121.5 and 147.5. No bends were noted in any models for the stroke outcomes.

Model selection and covariate adjustment

Two continuous variables, age and total physical activity, expressed non-linearity within the main analysis and male subgroup analysis for cardiovascular outcomes and as such regression splines were used with two and three knots respectively. Two knots were included within the female subgroup analysis for physical activity. For stroke outcome there were no bends in the main or sex-specific models. Further detail on this is provided in the supplementary digital content.

For peer review only - http://bmjopen.bmj.com/site/about/guidelines.xhtml

Within the main analysis for cardiovascular outcomes, the groups of depression only, Asian/Asian British ethnicity and BMI<18.5 covariates failed the proportionality assumption and as such, were incorporated into the model as a time varying coefficients. Within the sex specific models depression only failed the PH test within the female only analysis and ethnicity and BMI failed within the male only analysis. For the stroke outcomes gender and BMI class failed the proportionality assumption within the main analysis, with no failures within the sex-specific analysis. Analysis was repeated with the hypertension only as the comparator group to assess for any significant difference between the co-morbid group and the hypertension only group.

Time varying covariates

Due to the finding of MDD failing the proportionality assumption in the cardiovascular outcome in the primary analysis a series of further analyses have been performed to find when the assumption was not met. A log (-log) plot (fig 2) showed the proportionality assumption was broken at 22.5 months in the fully adjusted model in the primary analysis. As such, separate models were performed prior to and after these points. Prior to 22.5months the HR for MDD shows a trend that is reduced but insignificant (HR 0.82, 95%CI 0.6 - 1.13), becoming significantly increased after the 22.5 time point. (HR 1.27, 95%CI 1.06 - 1.52) (Table 9 supplementary digital content). Both stratified models passed the proportionality assumption using Schoenfeld residuals. Similar to the major analysis, the female model showed the MDD only group failing the proportionality assumption, although this was at the 29 month time point. (tables 6 and 10 of the supplementary digital content).

For peer review only - http://bmjopen.bmj.com/site/about/guidelines.xhtml

For peer review only

For peer review only - http://bmjopen.bmj.com/site/about/guidelines.xhtml

References

1. Palmer LJ. UK Biobank: bank on it. *Lancet* 2007;369(9578):1980-2. doi: 10.1016/S0140-6736(07)60924-6
2. Sudlow C, Gallacher J, Allen N, et al. UK Biobank: An Open Access Resource for Identifying the Causes of a Wide Range of Complex Diseases of Middle and Old Age. *Plos Medicine* 2015;12(3) doi: 10.1371/journal.pmed.1001779
3. Guo W, Bradbury KE, Reeves GK, et al. Physical activity in relation to body size and composition in women in UK Biobank. *Annals of Epidemiology* 2015;25(6):406-13.e6. doi: http://dx.doi.org/10.1016/j.annepidem.2015.01.015
4. Craig CL, Marshall AL, Sjostrom M, et al. International physical activity questionnaire: 12-country reliability and validity. *Medicine and science in sports and exercise* 2003;35(8):1381-95. doi: 10.1249/01.mss.0000078924.61453.fb [published Online First: 2003/08/06]
5. Townsend P. Deprivation. *Journal of Social Policy* 2009;16(02):125. doi: 10.1017/s0047279400020341
6. Townsend P, Phillimore M, Beattie A. Health and Deprivation: Inequality and the North. London: Croom Helm Ltd 1988.
7. WHO. Obesity: preventing and managing the global epidemic. Report of a WHO consultation. *World Health Organ Tech Rep Ser* 2000;894:i-xii, 1-253.
8. COMMITTEE. JF. British National Formulary. 67 ed. London: BMJ Group and Pharmaceutical Press 2014.

For peer review only - http://bmjopen.bmj.com/site/about/guidelines.xhtml

For peer review only

For peer review only - http://bmjopen.bmj.com/site/about/guidelines.xhtml

Supplementary Tables and figures

Supplementary Table1: Descriptive analysis for adverse cardiovascular outcome – males only

	Comparator group		Hypertension only		MDD only		Hypertension plus MDD	
	N= 21570		N = 30142		N = 4169		N = 5253	
**Median age (range)***	54	(47 - 61)	61	(54 - 65)	53	(46 - 60)	59	(52 - 64)
**Ethnicity, N (%)**								
White	19562	(90.69%)	27808	(92.26%)	3923	(94.1%)	5001	(95.2%)
Asian/Asian British	863	(4.%)	969	(3.21%)	87	(2.09%)	86	(1.64%)
Black/ Black British	559	(2.59%)	780	(2.59%)	52	(1.25%)	54	(1.03%)
**Median Townsend score (range)***	-1.87	(-3.47 - 0.59)	-2.08	(-3.53 - 0.41)	-1.58	(-3.3 - 1.07)	-1.81	(-3.44 - 0.78)
**Age at leaving full-time education, N (%)**								
<16	2517	(11.67%)	6328	(20.99%)	464	(11.13%)	1005	(19.13%)
16	4473	(20.74%)	6235	(20.69%)	859	(20.6%)	1096	(20.86%)
>16	14344	(66.5%)	17257	(57.25%)	2807	(67.33%)	3118	(59.36%)
**Total physical activity in metabolic**	4.15	(1.75 - 8.51)	3.99	(1.65 - 8.51)	4.15	(1.7 - 8.36)	3.76	(1.54 - 7.97)

For peer review only - http://bmjopen.bmj.com/site/about/guidelines.xhtml

**Sedentary time in hours, median (range)***	4.5	(3.5 - 6)	5	(3.5 - 6.5)	5	(3.5 - 6.5)	5	(4 - 7)
**Diabetes, N (%)**	721	(3.34%)	2401	(7.97%)	159	(3.81%)	477	(9.08%)
**Hypercholesterolaemia, N (%)**	1614	(7.48%)	5585	(18.53%)	363	(8.71%)	1056	(20.1%)
**Systolic BP in mmHg, median (range)***	128	(121.5 - 133.5)	149.5	(142 - 159)	127.5	(120.5 - 133)	148	(141 - 157)
**Body Mass Index, N (%)**								
<18.5	74	(0.34%)	35	(0.12%)	22	(0.53%)	12	(0.23%)
18.5 – 25	7607	(35.27%)	5842	(19.38%)	1394	(33.44%)	890	(16.94%)
25-30	10594	(49.11%)	15114	(50.14%)	2019	(48.43%)	2532	(48.2%)
>30	3295	(15.28%)	9151	(30.36%)	734	(17.61%)	1819	(34.63%)
**Smoking status, N (%)**								
Never smoked	12038	(55.81%)	15145	(50.25%)	1999	(47.95%)	2268	(43.18%)
Previously smoked	6777	(31.42%)	12125	(40.23%)	1447	(34.71%)	2295	(43.69%)
Current smoker	2688	(12.46%)	2776	(9.21%)	716	(17.17%)	686	(13.06%)
**Alcohol frequency, N (%)**								
Daily or almost daily	4822	(22.36%)	8653	(28.71%)	969	(23.24%)	1503	(28.61%)
Three or four times a week	5718	(26.51%)	7913	(26.25%)	1022	(24.51%)	1323	(25.19%)

For peer review only - http://bmjopen.bmj.com/site/about/guidelines.xhtml

Once or twice a week	5932	(27.5%)	7546	(25.03%)	1063	(25.5%)	1178	(22.43%)
One to three times a month	2193	(10.17%)	2392	(7.94%)	440	(10.55%)	479	(9.12%)
Special occasions only	1554	(7.2%)	2154	(7.15%)	328	(7.87%)	423	(8.05%)
Never	1343	(6.23%)	1473	(4.89%)	345	(8.28%)	345	(6.57%)
**Psychotropic medication, N (%)**	398	(1.85%)	670	(2.22%)	678	(16.26%)	879	(16.73%)

*Data presented as N (%) except * which are median values (interquartile range). Data presented as MET-hrs (hours spent doing exercise adjusted for multiples of basal metabolic rate in accordance with IPAQ).*

For peer review only - http://bmjopen.bmj.com/site/about/guidelines.xhtml

Supplementary Table 2: Descriptive analysis for adverse cardiovascular outcome – females only

	Comparator group		Hypertension only		MDD only		Hypertension plus MDD	
	N= 29228		N = 25893		N = 10929		N = 7676	
**Median age (range)***	54	(47 - 61)	61	(55 - 65)	53	(47 - 60)	60	(53 - 64)
**Ethnicity, N (%)**								
White	26585	(90.96%)	23441	(90.53%)	10324	(94.46%)	7271	(94.72%)
Asian/Asian British	908	(3.11%)	727	(2.81%)	174	(1.59%)	93	(1.21%)
Black/ Black British	764	(2.61%)	989	(3.82%)	167	(1.53%)	168	(2.19%)
**Median Townsend score (range)***	-1.90	(-3.44 - 0.51)	-2.06	(-3.5 - 0.38)	-1.66	(-3.3 - 0.84)	-1.87	(-3.4 - 0.74)
**Age at leaving full-time education, N (%)**								
<16	3399	(11.63%)	5757	(22.23%)	1261	(11.54%)	1602	(20.87%)
16	5792	(19.82%)	5592	(21.6%)	2319	(21.22%)	1636	(21.31%)
>16	19746	(67.56%)	14223	(54.93%)	7283	(66.64%)	4385	(57.13%)
**Total physical activity in metabolic**	3.87	(1.65 - 7.71)	3.51	(1.37 - 7.59)	3.79	(1.65 - 7.91)	3.65	(1.45 - 7.93)
**Sedentary time in hours, median (range)***	4	(3 - 5)	4	(3 - 5.5)	4	(3 - 5.5)	4.5	(3 - 6)

For peer review only - http://bmjopen.bmj.com/site/about/guidelines.xhtml

**Diabetes, N (%)**	547	(1.87%)	1376	(5.31%)	221	(2.02%)	452	(5.89%)
**Hypercholesterolaemia, N (%)**	1397	(4.78%)	3625	(14.%)	530	(4.85%)	1155	(15.05%)
**Systolic BP in mmHg, median (range)***	123.5	(115.5 - 130.5)	149.5	(142 - 160)	122.5	(114.5 - 130)	147.5	(140.5 - 157)
**Body Mass Index, N (%)**								
<18.5	315	(1.08%)	107	(0.41%)	81	(0.74%)	22	(0.29%)
18.5 – 25	14942	(51.12%)	7836	(30.26%)	4857	(44.44%)	1984	(25.85%)
25-30	9816	(33.58%)	10102	(39.01%)	3917	(35.84%)	2857	(37.22%)
>30	4155	(14.22%)	7848	(30.31%)	2074	(18.98%)	2813	(36.65%)
**Smoking status, N (%)**								
Never smoked	18588	(63.6%)	16358	(63.18%)	5865	(53.66%)	4186	(54.53%)
Previously smoked	8279	(28.33%)	8015	(30.95%)	3671	(33.59%)	2770	(36.09%)
Current smoker	2282	(7.81%)	1423	(5.5%)	1377	(12.6%)	695	(9.05%)
**Alcohol frequency, N (%)**								
Daily or almost daily	4628	(15.83%)	4317	(16.67%)	1767	(16.17%)	1378	(17.95%)
Three or four times a week	6457	(22.09%)	5120	(19.77%)	2231	(20.41%)	1514	(19.72%)
Once or twice a week	7712	(26.39%)	6343	(24.5%)	2817	(25.78%)	1738	(22.64%)

For peer review only - http://bmjopen.bmj.com/site/about/guidelines.xhtml

One to three times a month	3859	(13.2%)	3196	(12.34%)	1618	(14.8%)	1033	(13.46%)
Special occasions only	3980	(13.62%)	4176	(16.13%)	1576	(14.42%)	1306	(17.01%)
Never	2581	(8.83%)	2726	(10.53%)	917	(8.39%)	703	(9.16%)
**Psychotropic medication, N (%)**	943	(3.23%)	1125	(4.34%)	2166	(19.82%)	1643	(21.4%)

*Data presented as N (%) except * which are median values (interquartile range). Data presented as MET-hrs (hours spent doing exercise adjusted for multiples of basal metabolic rate in accordance with IPAQ).*

For peer review only - http://bmjopen.bmj.com/site/about/guidelines.xhtml

Supplementary Table 3: Descriptive analysis for stroke outcome – males only

	Comparator group		Hypertension only		MDD only		Hypertension plus MDD	
	N = 22816		N = 32787		N = 4438		N = 5857	
**Median age (range)***	55	(47 - 62.)	61	(54 - 65)	54	(47 - 61)	60	(53 - 64)
**Ethnicity, N (%)**								
White	20699	(90.72%)	30219	(92.17%)	4173	(94.03%)	5569	(95.08%)
Asian/Asian British	932	(4.08%)	1116	(3.4%)	102	(2.3%)	105	(1.79%)
Black/ Black British	576	(2.52%)	820	(2.5%)	53	(1.19%)	59	(1.01%)
**Median Townsend score (range)***	-1.88	(-3.47 - 0.59)	-2.05	(-3.5 - 0.46)	-1.56	(-3.28 - 1.15)	-1.74	(-3.4 - 0.93)
**Age at leaving full-time education, N (%)**								
<16	2900	(12.71%)	7256	(22.13%)	558	(12.57%)	1193	(20.37%)
16	4702	(20.61%)	6704	(20.45%)	909	(20.48%)	1222	(20.86%)
>16	14960	(65.57%)	18471	(56.34%)	2930	(66.02%)	3397	(58.%)
**Total physical activity in metabolic**	4.12	(1.74 - 8.48)	3.96	(1.65 - 8.44)	4.13	(1.67 - 8.36)	3.66	(1.45 - 7.83)
**Sedentary time in hours, median (range)***	5	(3.5 - 6)	5	(4 - 7)	5	(3.5 - 6.5)	5	(4 - 7)

For peer review only - http://bmjopen.bmj.com/site/about/guidelines.xhtml

**Diabetes, N (%)**	873	(3.83%)	2951	(9.%)	208	(4.69%)	635	(10.84%)
**Hypercholesterolaemia, N (%)**	2045	(8.96%)	6736	(20.54%)	457	(10.3%)	1293	(22.08%)
**Systolic BP in mmHg, median (range)***	128	(121.5 - 133.5)	149	(142 - 159)	127	(120.5 - 133)	147.5	(140.5 - 156.5)
**Body Mass Index, N (%)**								
<18.5	79	(0.35%)	39	(0.12%)	22	(0.5%)	12	(0.2%)
18.5 – 25	7867	(34.48%)	6215	(18.96%)	1452	(32.72%)	960	(16.39%)
25-30	11203	(49.1%)	16341	(49.84%)	2142	(48.26%)	2780	(47.46%)
>30	3667	(16.07%)	10192	(31.09%)	822	(18.52%)	2105	(35.94%)
**Smoking status, N (%)**								
Never smoked	12502	(54.79%)	16054	(48.96%)	2094	(47.18%)	2469	(42.15%)
Previously smoked	7399	(32.43%)	13603	(41.49%)	1582	(35.65%)	2610	(44.56%)
Current smoker	2836	(12.43%)	3013	(9.19%)	754	(16.99%)	770	(13.15%)
**Alcohol frequency, N (%)**								
Daily or almost daily	5085	(22.29%)	9309	(28.39%)	1021	(23.01%)	1645	(28.09%)
Three or four times a week	6039	(26.47%)	8556	(26.1%)	1077	(24.27%)	1450	(24.76%)
Once or twice a week	6264	(27.45%)	8161	(24.89%)	1121	(25.26%)	1305	(22.28%)

For peer review only - http://bmjopen.bmj.com/site/about/guidelines.xhtml

One to three times a month	2307	(10.11%)	2642	(8.06%)	478	(10.77%)	538	(9.19%)
Special occasions only	1666	(7.3%)	2394	(7.3%)	355	(8.%)	503	(8.59%)
Never	1444	(6.33%)	1711	(5.22%)	383	(8.63%)	414	(7.07%)
**Psychotropic medication, N (%)**	429	(1.88%)	793	(2.42%)	735	(16.56%)	1025	(17.5%)

*Data presented as N (%) except * which are median values (interquartile range). Data presented as MET-hrs (hours spent doing exercise adjusted for multiples of basal metabolic rate in accordance with IPAQ).*

For peer review only

For peer review only - http://bmjopen.bmj.com/site/about/guidelines.xhtml

Supplementary Table 4: Descriptive analysis for stroke outcome – females only

	Comparator group		Hypertension only		MDD only		Hypertension plus MDD	
	N = 29684		N = 26937		N = 11143		N = 8090	
**Median age (range)***	54	(47 - 61)	61	(56 - 65)	53	(47 - 60)	60	(54 - 64)
**Ethnicity, N (%)**								
White	26998	(90.95%)	24359	(90.43%)	10524	(94.44%)	7643	(94.47%)
Asian/Asian British	925	(3.12%)	773	(2.87%)	178	(1.6%)	104	(1.29%)
Black/ Black British	779	(2.62%)	1034	(3.84%)	170.00	(1.53%)	187	(2.31%)
**Median Townsend score (range)***	-1.90	(-3.44 - 0.52)	-2.03	(-3.48 - 0.43)	-1.66	(-3.29 - 0.86)	-1.83	(-3.38 - 0.85)
**Age at leaving full-time education, N (%)**								
<16	3546	(11.95%)	6140	(22.79%)	1326	(11.9%)	1752	(21.66%)
16	5888	(19.84%)	5803	(21.54%)	2361	(21.19%)	1731	(21.4%)
>16	19954	(67.22%)	14643	(54.36%)	7387	(66.29%)	4550	(56.24%)
**Total physical activity in metabolic**	3.85	(1.65 - 7.7)	3.49	(1.35 - 7.57)	3.79	(1.65 - 7.89)	3.61	(1.41 - 7.87)

For peer review only - http://bmjopen.bmj.com/site/about/guidelines.xhtml

**Sedentary time in hours, median (range)***	4.0	(3 - 5)	4.0	(3 - 5.5)	4.0	(3 - 5.5)	4.5	(3 - 6)
**Diabetes, N (%)**	581	(1.96%)	1551	(5.76%)	241	(2.16%)	528	(6.53%)
**Hypercholesterolaemia, N (%)**	1547	(5.21%)	4032	(14.97%)	592	(5.31%)	1327	(16.4%)
**Systolic BP in mmHg, median (range)***	123.5	(115.5 - 131)	149.5	(142 - 160)	122.5	(114.5 - 130)	147.0	(140.5 - 157)
**Body Mass Index, N (%)**								
<18.5	316	(1.06%)	112	(0.42%)	82	(0.74%)	26	(0.32%)
18.5 – 25	15100	(50.87%)	8027	(29.8%)	4922	(44.17%)	2057	(25.43%)
25-30	9982	(33.63%)	10476	(38.89%)	4007	(35.96%)	2989	(36.95%)
>30	4286	(14.44%)	8322	(30.89%)	2132	(19.13%)	3018	(37.31%)
**Smoking status, N (%)**								
Never smoked	18816	(63.39%)	16928	(62.84%)	5958	(53.47%)	4365	(53.96%)
Previously smoked	8452	(28.47%)	8416	(31.24%)	3758	(33.73%)	2950	(36.46%)
Current smoker	2334	(7.86%)	1488	(5.52%)	1409	(12.64%)	749	(9.26%)
**Alcohol frequency, N (%)**								

For peer review only - http://bmjopen.bmj.com/site/about/guidelines.xhtml

Daily or almost daily	4675	(15.75%)	4442	(16.49%)	1796	(16.12%)	1440	(17.8%)
Three or four times a week	6524	(21.98%)	5271	(19.57%)	2258	(20.26%)	1570	(19.41%)
Once or twice a week	7825	(26.36%)	6558	(24.35%)	2872	(25.77%)	1820	(22.5%)
One to three times a month	3913	(13.18%)	3329	(12.36%)	1644	(14.75%)	1089	(13.46%)
Special occasions only	4078	(13.74%)	4400	(16.33%)	1623	(14.57%)	1382	(17.08%)
Never	2658	(8.95%)	2919	(10.84%)	947	(8.5%)	785	(9.7%)
**Psychotropic medication, N (%)**	979	(3.3%)	1203	(4.47%)	2241	(20.11%)	1753	(21.67%)

*Data presented as N (%) except * which are median values (interquartile range). Data presented as MET-hrs (hours spent doing exercise adjusted for multiples of basal metabolic rate in accordance with IPAQ).*

For peer review only - http://bmjopen.bmj.com/site/about/guidelines.xhtml

Supplementary Table 5: Risk of adverse cardiovascular event by clinical group, in males only.

	Model one (unadjusted)			Model two (partially adjusted)*			Model three (fully adjusted) †		
**Group**	H.R.	95% C.I.	p-value	H.R.	95% C.I.	p-value	H.R.	95% C.I.	p-value
No Hypertension- No MDD	1(ref)			1(ref)			1(ref)		
Hypertension only	2.21	(2.00-2.45)	**$2.28x10^{-53}$**	1.62	(1.46-1.83)	**$5.80x10^{-19}$**	1.29	(1.13-1.47)	**$1.35x10^{-4}$**
MDD only	1.17	(0.95-1.56)	0.12	1.18	(0.95-1.46)	0.12	1.12	(0.9-1.39)	0.3
Hypertension and MDD	2.46	(2.13-2.84)	**$3.12x10^{-34}$**	1.95	(1.68-2.27)	**$2.81x10^{-18}$**	1.47	(1.24-1.74)	**$8.71x10^{-6}$**

**Adjusted for sociodemographic factors (age, Townsend score, age of leaving full time education and ethnicity. †Additionally adjusted for history of diabetes, history of hypercholesterolemia, BMI, smoking history, alcohol use, systolic blood pressure, sedentary hours per day, physical activity and psychotropic medication use. MDD = Major depressive disorder, H.R. =Hazard ratio, C.I.= Confidence interval*

For peer review only

Supplementary Table 6: Risk of adverse cardiovascular event by clinical group, in females only.

	Model one (unadjusted)			Model two (partially adjusted)*			Model three (fully adjusted) †		
**Group**	H.R.	95% C.I.	p-value	H.R.	95% C.I.	p-value	H.R.	95% C.I.	p-value
No Hypertension - No MDD	1(ref)			1(ref)			1(ref)		
Hypertension only	2.75	(2.38 - 3.18)	**6.16×10^{-43}**	1.86	(1.6-2.17)	**1.43×10^{-15}**	1.64	(1.33-2.02)	**4.36×10^{-6}**
MDD only	0.67	(0.42-1.08)	0.10	0.72	(0.45-1.17)	0.19	0.68	(0.42-1.1)	0.12
Hypertension and MDD	3.68	(3.1-4.38)	**5.62×10^{-49}**	2.78	(1.58-3.29)	**4.62×10^{-29}**	2.18	(1.82-2.92)	**4.76×10^{-11}**
**Time varying Variables**									
MDD only	1.02	(1.006-1.03)	**2.45×10^{-3}**	1.02	(1.005-1.03)	**4.00×10^{-3}**	1.02	(1.004-1.03)	**6.19×10^{-3}**

**Adjusted for sociodemographic factors (age, Townsend score, age of leaving full time education and ethnicity. †Additionally adjusted for history of diabetes, history of hypercholesterolemia, BMI, smoking history, alcohol use, systolic blood pressure, sedentary hours per day, physical activity and psychotropic medication use. MDD = Major depressive disorder, H.R. =Hazard ratio, C.I.= Confidence interval*

For peer review only

Supplementary Table 7: Risk of stroke event by clinical group, in males only.

	Model one (unadjusted)			Model two (partially adjusted)*			Model three (fully adjusted) †		
**Group**	H.R.	95% C.I.	p-value	H.R.	95% C.I.	p-value	H.R.	95% C.I.	p-value
No Hypertension - No MDD	1(ref)			1(ref)			1(ref)		
Hypertension only	2.43	(1.95 - 3.03)	**1.92×10^{-15}**	1.74	(1.38 - 2.19)	**2.58×10^{-6}**	1.19	(0.9 - 1.58)	0.22
MDD only	1.45	(0.96 - 2.2)	0.07	1.65	(1.09 - 2.5)	0.02	1.49	(0.97 - 2.29)	0.07
Hypertension and MDD	2.39	(1.74 - 3.27)	**7.34×10^{-8}**	1.87	(1.35 - 2.6)	**1.55×10^{-4}**	1.20	(0.83 - 1.74)	0.33

**Adjusted for sociodemographic factors (age, sex, Townsend score, age of leaving full time education and ethnicity. †Additionally adjusted for history of diabetes, history of hypercholesterolemia, BMI, smoking history, alcohol use, systolic blood pressure, sedentary hours per day, physical activity and psychotropic medication use. MDD = Major depressive disorder, H.R. =Hazard ratio, C.I.= Confidence interval*

For peer review only - http://bmjopen.bmj.com/site/about/guidelines.xhtml

Supplementary Table 8: Risk of stroke event by clinical group, in females only.

	Model one (unadjusted)			Model two (partially adjusted)*			Model three (fully adjusted) †		
**Group**	H.R.	95% C.I.	p-value	H.R.	95% C.I.	p-value	H.R.	95% C.I.	p-value
No Hypertension - No MDD	1(ref)			1(ref)			1(ref)		
Hypertension only	2.38	(1.84 - 3.09)	**$6.50x10^{-11}$**	1.51	(1.14 - 1.99)	**$3.63x10^{-3}$**	1.25	(0.88 - 1.79)	0.21
MDD only	1.09	(0.73 - 1.62)	0.67	1.15	(0.76 - 1.75)	0.51	0.99	(0.64 - 1.53)	0.98
Hypertension and MDD	3.05	(2.22 - 4.21)	**$8.71x10^{-12}$**	2.22	(1.59 - 3.08)	**$2.27x10^{-6}$**	1.62	(1.08 - 2.42)	0.02

**Adjusted for sociodemographic factors (age, sex, Townsend score, age of leaving full time education and ethnicity. †Additionally adjusted for history of diabetes, history of hypercholesterolemia, BMI, smoking history, alcohol use, systolic blood pressure, sedentary hours per day, physical activity and psychotropic medication use. MDD = Major depressive disorder, H.R. =Hazard ratio, C.I.= Confidence interval*

For peer review only - http://bmjopen.bmj.com/site/about/guidelines.xhtml

Supplementary Table 9: Time stratified analysis by moment of proportional hazards failure for adverse cardiovascular outcomes (stratified at 22.5 months)

	Fully adjusted* model pre-22.5 months			Fully adjusted* model post-22.5 months		
**Group**	H.R.	95% C.I.	p-value	H.R.	95% C.I.	p-value
No Hypertension - No MDD	1(ref)			1(ref)		
Hypertension only	1.36	(1.12 - 1.66)	**0.002**	1.36	(1.19 -1.55)	**$5.06x10^{-6}$**
MDD only	0.82	(0.60 - 1.13)	0.22	1.27	(1.06 -1.52)	**0.01**
Hypertension and MDD	1.75	(1.39 - 2.21)	**$2.62x10^{-6}$**	1.62	(1.38 - 1.90)	**$5.72x10^{-9}$**

**Adjusted for sociodemographic factors (age, sex, Townsend score, age of leaving full time education and ethnicity, history of diabetes, history of hypercholesterolemia, BMI, smoking history, alcohol use, systolic blood pressure, sedentary hours per day, physical activity and psychotropic medication use.*

MDD = Major depressive disorder, H.R. =Hazard ratio, C.I.= Confidence interval

For peer review only - http://bmjopen.bmj.com/site/about/guidelines.xhtml

Supplementary Table 10: Time stratified analysis by moment of proportional hazards failure for adverse cardiovascular outcomes (females only - stratified at 29 months)

	Fully adjusted* model pre-29 months			Fully adjusted* model post-29 months		
**Group**	H.R.	95% C.I.	p-value	H.R.	95% C.I.	p-value
No Hypertension - No MDD	1(ref)			1(ref)		
Hypertension only	1.49	(1.06 - 2.08)	**0.02**	1.75	(1.33 - 2.30)	**$5.56x10^{-5}$**
MDD only	0.73	(0.48 - 1.10)	0.13	1.58	(1.19 - 2.09)	**0.002**
Hypertension and MDD	1.80	(1.24 - 2.62)	**0.002**	2.47	(1.83 - 3.33)	**$2.89x10^{-9}$**

**Adjusted for sociodemographic factors (age, sex, Townsend score, age of leaving full time education and ethnicity, history of diabetes, history of hypercholesterolemia, BMI, smoking history, alcohol use, systolic blood pressure, sedentary hours per day, physical activity and psychotropic medication use.*

MDD = Major depressive disorder, H.R. =Hazard ratio, C.I.= Confidence interval

For peer review only - http://bmjopen.bmj.com/site/about/guidelines.xhtml

Supplementary Table 11: Relative excess risk due to interaction results on fully adjusted* models

Analysis	RERI	95% C.I.
Adverse cardiovascular outcome before 22.5 months	**0.563**	**(0.189 - 0.938)**
Adverse cardiovascular outcome after 22.5 months	-0.009	(-0.293 - 0.275)
Adverse cardiovascular outcome (males only)	0.058	(-0.240 - 0.357)
Adverse cardiovascular outcome (females only)before 29 months	**0.588**	**(0.074 - 1.103)**
Adverse cardiovascular outcome (females only)after 29 months	0.142	(-0.447 - 0.732)
Stroke outcome	-0.047	(-0.485 - 0.391)
Stroke outcome (males only)	-0.480	(-1.195 - 0.234)
Stroke outcome (females only)	0.372	(-0.216 - 0.959)

**Adjusted for sociodemographic factors (age, sex, Townsend score, age of leaving full time education and ethnicity, history of diabetes, history of hypercholesterolemia, BMI, smoking history, alcohol use, systolic blood pressure, sedentary hours per day, physical activity and psychotropic medication use.*

RERI = Relative excess risk due to interaction, C.I.= Confidence interval

For peer review only - http://bmjopen.bmj.com/site/about/guidelines.xhtml

Supplementary Table 12: Comparison of additional hypertension factors (medication and diagnosis duration) across groups

	No Hypertension – No MDD		Hypertension only		MDD only		Hypertension and MDD	
**Antihypertensive medication prescription, N (%)**	1,265	(2.49)	19,045	(33.99)	476	(3.04)	5,037	(37.34)
**Number of antihypertensive medications, N (range)***	1	(1-1)	1	(1-2)	1	(1-1)	1	(1- 2)
**Reported a duration of hypertension, N (%)**	*1,376*	(2.71)	*16,709*	(29.82)	678	(4.32)	4,525	(33.55)
**Duration of hypertension in years, median (range)***	6	(2-14)	8	(4-13)	6	(3-14)	8	(4 - 14)

*****Median quantity of antihpertensive medications and median duration of hypertensive diagnosis presented for those on antihypertensive medications and supplied an age of hypertension diagnosis, respectively.MDD = Major Depressive disorder*

For peer review only - http://bmjopen.bmj.com/site/about/guidelines.xhtml

Reporting checklist for cohort study.

Based on the STROBE cohort guidelines.

Instructions to authors

Complete this checklist by entering the page numbers from your manuscript where readers will find each of the items listed below.

Your article may not currently address all the items on the checklist. Please modify your text to include the missing information. If you are certain that an item does not apply, please write "n/a" and provide a short explanation.

Upload your completed checklist as an extra file when you submit to a journal.

In your methods section, say that you used the STROBE cohort reporting guidelines, and cite them as:

von Elm E, Altman DG, Egger M, Pocock SJ, Gotzsche PC, Vandenbroucke JP. The Strengthening the Reporting of Observational Studies in Epidemiology (STROBE) Statement: guidelines for reporting observational studies.

		Reporting Item	Page Number
Title	#1a	Indicate the study’s design with a commonly used term in the title or the abstract	1
Abstract	#1b	Provide in the abstract an informative and balanced summary of what was done and what was found	3
Background / rationale	#2	Explain the scientific background and rationale for the investigation being reported	5
Objectives	#3	State specific objectives, including any prespecified hypotheses	5
Study design	#4	Present key elements of study design early in the paper	5
Setting	#5	Describe the setting, locations, and relevant dates, including periods of recruitment, exposure, follow-up, and data collection	6
Eligibility criteria	#6a	Give the eligibility criteria, and the sources and methods of selection of participants. Describe methods of follow-up.	6-7

For peer review only

	#6b	For matched studies, give matching criteria and number of exposed and unexposed	n/a
Variables	#7	Clearly define all outcomes, exposures, predictors, potential confounders, and effect modifiers. Give diagnostic criteria, if applicable	7-8
Data sources / measurement	#8	For each variable of interest give sources of data and details of methods of assessment (measurement). Describe comparability of assessment methods if there is more than one group. Give information separately for for exposed and unexposed groups if applicable.	6-8
Bias	#9	Describe any efforts to address potential sources of bias	6
Study size	#10	Explain how the study size was arrived at	6
Quantitative variables	#11	Explain how quantitative variables were handled in the analyses. If applicable, describe which groupings were chosen, and why	See note 1
Statistical methods	#12a	Describe all statistical methods, including those used to control for confounding	8-9
	#12b	Describe any methods used to examine subgroups and interactions	See note 2
	#12c	Explain how missing data were addressed	6-7
	#12d	If applicable, explain how loss to follow-up was addressed	1
	#12e	Describe any sensitivity analyses	9
Participants	#13a	Report numbers of individuals at each stage of study—eg numbers potentially eligible, examined for eligibility, confirmed eligible, included in the study, completing follow-up, and analysed. Give information separately for for exposed and unexposed groups if applicable.	10
	#13b	Give reasons for non-participation at each stage	6,7
	#13c	Consider use of a flow diagram	n/a
Descriptive data	#14a	Give characteristics of study participants (eg demographic, clinical, social) and information on exposures and potential	10

For peer review only

		confounders. Give information separately for exposed and unexposed groups if applicable.	
	#14b	Indicate number of participants with missing data for each variable of interest	See note 3
	#14c	Summarise follow-up time (eg, average and total amount)	10
Outcome data	#15	Report numbers of outcome events or summary measures over time. Give information separately for exposed and unexposed groups if applicable.	6
Main results	#16a	Give unadjusted estimates and, if applicable, confounder-adjusted estimates and their precision (eg, 95% confidence interval). Make clear which confounders were adjusted for and why they were included	11-12
	#16b	Report category boundaries when continuous variables were categorized	n/a
	#16c	If relevant, consider translating estimates of relative risk into absolute risk for a meaningful time period	n/a
Other analyses	#17	Report other analyses done—e.g., analyses of subgroups and interactions, and sensitivity analyses	See note 4
Key results	#18	Summarise key results with reference to study objectives	12
Limitations	#19	Discuss limitations of the study, taking into account sources of potential bias or imprecision. Discuss both direction and magnitude of any potential bias.	14
Interpretation	#20	Give a cautious overall interpretation considering objectives, limitations, multiplicity of analyses, results from similar studies, and other relevant evidence.	13-17
Generalisability	#21	Discuss the generalisability (external validity) of the study results	15
Funding	#22	Give the source of funding and the role of the funders for the present study and, if applicable, for the original study on which the present article is based	18

Author notes

1. 6,7,8,9, supplementary

For peer review only

2. 8-9, supplementary

3. n/a (supplementary)

4. 11-12, supplemental

The STROBE checklist is distributed under the terms of the Creative Commons Attribution License CC-BY. This checklist was completed on 25. May 2018 using http://www.goodreports.org/, a tool made by the EQUATOR Network in collaboration with Penelope.ai

For peer review only

Impact of major depression on cardiovascular outcomes for individuals with hypertension: prospective survival analysis in UK Biobank.

Journal:	*BMJ Open*
Manuscript ID	bmjopen-2018-024433.R1
Article Type:	Original research
Date Submitted by the Author:	11-Dec-2018
Complete List of Authors:	Graham, Nicholas; University of Glasgow Institute of Health and Wellbeing, Gartnavel Royal Hopsital 1055 Great Western Road Glasgow, UK G12 0XH<br>Ward, Joey; University of Glasgow Institute of Health and Wellbeing<br>Mackay, Daniel; University of Glasgow Institute of Health and Wellbeing<br>Pell, J. P.; University of Glasgow Institute of Health and Wellbeing<br>Cavanagh, Jonathan; University of Glasgow Institute of Health and Wellbeing<br>Padmanabhan, Sandosh; University of Glasgow, Institute of Cardiovascular and Medical Sciences, British Heart Foundation Glasgow Cardiovascular Research Centre<br>Smith, Daniel; University of Glasgow, Institute of Health and Wellbeing
**Primary Subject Heading**:	Epidemiology
Secondary Subject Heading:	Mental health, Cardiovascular medicine
Keywords:	EPIDEMIOLOGY, mortality, cardiovascular disease, morbidity, depression, Hypertension < CARDIOLOGY

SCHOLARONE™ Manuscripts

Impact of major depression on cardiovascular outcomes for individuals with hypertension: prospective survival analysis in UK Biobank.

Short title: Outcomes of Hypertension plus Depression

Nicholas A GRAHAM*[a], Clinical Research Fellow

Joey WARD[a], Research Fellow

Daniel MACKAY[b], Reader in Public Health

Jill PELL[b], Professor of Public Health

Jonathan CAVANAGH[c], Professor of Psychiatry

Sandosh PADMANABHAN[d], Professor of Cardiovascular Genomics and Therapeutics

Daniel J. SMITH[a], Professor of Psychiatry.

Number of Supplementary files: 1

Word count of Manuscript: 3,836 (exc. Tables, references, abstract, summary and Author contribution statements)

Word count of Supplementary file: 1,455 (exc. tables)

Number of tables and figures: 18 tables (including 12 in supplementary digital content) and 3 figures

[a]Institute of Health and Wellbeing, University of Glasgow, Gartnavel Royal Hospital, 1055 Great Western Road, Glasgow G12 OXH. [b]Institute of Health and Wellbeing, University of Glasgow, Public Health, 1 Lilybank Gardens, Glasgow G12 8RZ. [c]Institute of Health and Wellbeing, Centre for Immunobiology, Sir Graeme Davies Building College of Medical, Veterinary and Life Sciences University of Glasgow. [d]Institute of Cardiovascular and Medical Sciences, British Heart Foundation Glasgow Cardiovascular Research centre, University of Glasgow, Glasgow G12 8TA.

For peer review only - http://bmjopen.bmj.com/site/about/guidelines.xhtml

For peer review only

*corresponding author: nicholas.graham@glasgow.ac.uk, phone: +44 0141 211 3918,

CONFLICTS OF INTEREST: None.

For peer review only

For peer review only - http://bmjopen.bmj.com/site/about/guidelines.xhtml

ABSTRACT

Objectives: To assess whether a history of major depressive disorder (MDD) in middle-aged individuals with hypertension influences first-onset cardiovascular disease outcomes.

Design: Prospective cohort survival analysis using Cox proportional hazards regression with a median follow-up of 63 months (702,902 person-years). Four mutually exclusive groups were compared: hypertension only (n=56,035), MDD only (n=15,098), comorbid hypertension plus MDD (n=12,929), and an unaffected (no hypertension, no MDD) comparison group (n=50,798).

Setting: UK Biobank

Participants: UK Biobank participants without cardiovascular disease aged 37–73 who completed psychiatric questions relating ICD-10 diagnostic criteria on a touchscreen questionnaire at baseline interview in 2006–2010 (n=134,860).

Primary and Secondary outcome measures: First-onset adverse cardiovascular outcomes leading to hospital admission or death (ICD-10 codes I20-I259, I60-69 and G45- G46), adjusted in a stepwise manner for sociodemographic, health and lifestyle features. Secondary analyses were performed looking specifically at stroke outcomes (ICD-10 codes I60-69 and G45- G46) and in models separated by gender.

Results: Relative to controls, adjusted hazard ratios (HRs) for adverse cardiovascular outcomes were increased for the hypertension only group (HR=1.36, 95%CI 1.22-1.52) and were higher still for the comorbid hypertension plus MDD group (HR=1.66, 95%CI 1.45-1.9). HRs for the comorbid hypertension plus MDD group were significantly raised compared to hypertension alone (HR=1.22, 95%CI 1.1-1.35). An additive interaction measured using relative excess risk due to interaction (RERI) was identified at baseline (RERI=0.563, 95%CI 0.189 - 0.938) but not maintained during follow-up.

Limitations: Possible selection bias in UK Biobank and inability to assess for levels of medication adherence.

For peer review only - http://bmjopen.bmj.com/site/about/guidelines.xhtml

Conclusions: Comorbid hypertension and MDD conferred greater hazard than hypertension alone for adverse cardiovascular outcomes, although evidence of an additive interaction between hypertension and MDD was inconsistent over time. Future cardiovascular risk prediction tools may benefit from the inclusion of questions about prior history of depressive disorders.

Word count of Abstract: 297

Key words: epidemiology, mortality, morbidity, depression; hypertension, cardiovascular disease

Article Summary

STRENGTHS AND LIMITATIONS

- Methodological advantages over previous studies, including a very large sample size, adjustment for a more comprehensive range of confounders, and the inclusion of non-fatal adverse cardiovascular events from hospital admission data and death registry data.
- Definition of prior MDD history was based on ICD-10 diagnostic criteria (rather than a score on a symptoms questionnaire) and our composite definition of hypertension incorporated past history, current medication and objective blood pressure measurements.
- Although analyses were adjusted for a broad range of baseline factors (such as smoking status, BMI, psychotropic medication use and diabetes), we were unable to account for how these factors may have changed over the course of follow-up, or assess adherence to cardiovascular medications.
- Trained nurses interviewed UK Biobank participants, but the self-report nature of some of these data may represent a limitation.
- UK Biobank may have issues with respect to selection biases. For example, individuals with more severe MDD may have been less likely to volunteer.

For peer review only

INTRODUCTION

By 2030 major depressive disorder (MDD) and cardiovascular disease (CVD) will be the two leading causes of disability worldwide[1]. It is established that individuals with MDD are at increased risk of developing CVD and that they experience worse long-term outcomes[2]. To date, studies looking at the interaction between hypertension and MDD have focussed on all-cause death[3-5] cardiovascular death[5] or incorporated individuals with previous CVD[3-6], and suggested a possible additive interaction between hypertension and MDD on survival[5 6]. MDD is well known to worsen post-cardiovascular event survival[6 7]. The risk to first onset cardiovascular is not known. Within this study we look specifically at first onset events, irrespective of whether they lead to death or not.

Hypertension is extremely common (affecting 1 billion people worldwide)[8] and is responsible for 50% of all cardiovascular disease[9]. It is commonly comorbid with MDD[10 11], with recent meta-analysis showing 27% of individuals with hypertension having MDD[12] and population-based studies showing a hypertension prevalence of 21% in those with MDD[11]. A biological link has been found by genome-wide association studies, showing calcium-channel genes, important in blood pressure (BP) control and hypertension[13], also act to increase risk for MDD[14 15] and bipolar disorder[16 17]. The sympathetic nervous system (SNS), Renin-angiotensin system, the immune system and the cortisol stress response system are all also implicated in both conditions[18]. Medication management of both conditions are also thought to impact one another with side effects of psychotropic medications including raised BP and vice versa[19-21], although there is contrary evidence suggesting either medication or MDD may in actual fact be protective of hypertension[20 22].

Here we make use of prospective data from the UK Biobank cohort[23] to test the hypothesis that a lifetime history of MDD in individuals with hypertension (but no previous history of CVD) impacts adversely on first-episode cardiovascular events. We also assess whether MDD exacerbates the effects of hypertension as a risk factor for cardiovascular events. Given the high global prevalence of

For peer review only - http://bmjopen.bmj.com/site/about/guidelines.xhtml

MDD and hypertension[24], this is an important question for public health, which could inform future treatment approaches for both conditions.

METHODS

Study design

This was population cohort study using data from UK Biobank. Four mutually exclusive groups (hypertension only, MDD only, hypertension plus MDD, and a comparison group) were compared for adverse CVD outcomes, as well as stroke outcomes

Sample description

UK Biobank is a large population cohort of 502,655 participants recruited between April 2007 and July 2010 from 21 assessment centres located across Great Britain[23]. Participants aged 40-69 were invited to take participate if registered with the NHS and lived within a reasonable distance of an assessment centre. At baseline assessment participants completed a series of detailed assessments relating to lifestyle and medical history on touchscreen questionnaire and have a range of physical health measurements, including body mass index (BMI) and BP taken by a nurse. UK Biobank was approved by the North West NHS Multi-Centre Research Ethics Committee and all participants provided written informed consent to participate. This analysis is part of UK Biobank approved application number 7155.

During the last two years of recruitment, questions relating to mood disorder features were added to the baseline assessment schedule questionnaire. From the 172,729 participants asked these questions, 134,860 provided sufficient responses to be included in our analysis. We excluded participants from our analyses based on the following *a priori* criteria: a history of bipolar disorder (n=1,831) or schizophrenia (n=262); where there were insufficient data provided by participants to clearly rule out MDD (n= 25,520) or hypertension (n=1,080); and where there were coding errors for date and/or time of death (n=4). These exclusions were based on self-report and criteria from Smith

For peer review only - http://bmjopen.bmj.com/site/about/guidelines.xhtml

et al. for the psychiatric outcomes where available, or where they responded “don’t know” or “prefer not to answer” to questions or data was missing that would limit our ability to exclude the presence of hypertension or MDD. Participants were further excluded from the adverse CVD outcome if they had a record of CVD prior to recruitment (self-reported angina, myocardial infarction (MI) or stroke, or previous hospital admission for angina, MI or stroke) (n= 9,172). For the stroke outcome this exclusion was limited to a record of stroke prior to baseline assessment (self-report or previous hospital admission for stroke) (n=2,280).

Classification of hypertension and MDD

Participants were defined as having hypertension if either: *a)* mean BP at baseline was greater than clinically-defined criteria over two measurements (systolic BP greater than or equal to 140 mmHg or diastolic BP greater than or equal to 90 mmHg. Where only one reading was available this was used (n=1,571)); or *b)* self-reported ‘hypertension diagnosed by a doctor’ plus self-report of currently taking antihypertensive medication. This composite classification was used to ensure that undiagnosed hypertensive participants were not misclassified and is in line with similar epidemiological studies[5 25 26]. The requirement for antihypertensive use in the context of a history of hypertension was incorporated to limit those on beta-blockers for anxiety. According to these criteria, n=68,964 participants (51.1% of the sample) had hypertension for the adverse cardiovascular outcomes analysis and n=73,671 participants (52% of the sample) had hypertension in the stroke outcome analysis.

A history of lifetime MDD was defined according to the criteria for mood disorders developed by Smith et al[27 28] and has been used in further papers[28-32]. (n=28,027 adverse cardiovascular outcomes; n =29,528 stroke outcomes). This is described in more detail within the supplementary content.

For the adverse cardiovascular outcomes, the remainder of the sample, with no history of hypertension or MDD (n=50,798) were classified as a comparator group. The three mutually

For peer review only - http://bmjopen.bmj.com/site/about/guidelines.xhtml

exclusive diagnostic groups for this study were therefore: hypertension only (n=56,035); MDD only (n=15,098) and hypertension plus MDD (n= 12,929). For the stroke outcomes, the mutually exclusive groups were hypertension only (n=59,724); MDD only (n=15,581) and hypertension plus MDD (n= 13,947) and no hypertension – no MDD (n=52,502).

Outcomes

The primary outcome was defined as a first-episode cardiovascular event leading to hospital admission or death, specifically angina, MI, or chronic ischaemic heart disease (ICD-10 codes I20-I259), and transient ischaemic attack (TIA) or stroke (ICD-10 codes I60-69 and G45- G46). A secondary outcome was defined as stroke leading to hospital admission or death (ICD-10 codes I60-69 and G45- G46)[33] due to the strength of relationship hypertension has with this outcome in particular[9]. Admission data were obtained from Hospital Episode Statistics in England, Patient Episode Database for Wales and Scottish Morbidity Records in Scotland. Mortality outcomes were obtained from the National Health Service (NHS) Information Centre for England and Wales and from the NHS Central Register for Scotland. Individuals who died from a non-cardiovascular cause/stroke were censored at the time of death but not recorded as having an event. Admission data were available for Scottish, English and Welsh participants until 31 August 2014, 31 March 2015 and 28 February 2015 respectively. End of follow-up was classified as these dates unless preceded by date of death or the date of first cardiovascular admission. In total 3,685 (2.73%) participants had a first-episode cardiovascular event during the follow-up period (total number of all deaths plus non-fatal cardiovascular events = 5,788) and 910 (0.64%) participants had a first-episode stroke event (total number of all deaths plus non-fatal stroke events = 7,317).

Confounding variables

Information on potential confounding factors was available for age, sex, socioeconomic status (Townsend score)[34], self-reported ethnicity, age of leaving full-time education, diabetes, body mass

For peer review only

For peer review only - http://bmjopen.bmj.com/site/about/guidelines.xhtml

index (BMI), systolic BP, hypercholesterolemia, alcohol use, smoking history, sedentary behaviour (number of hours each day spent sitting at a computer, television or driving), physical activity levels[35] and psychotropic medication use. Specific details on these variables are provided in supplementary content.

Analyses

Baseline characteristics were compared between groups using Chi-squared tests for categorical variables and Kruskal Wallis for continuous variables. Confounding variables were assessed for differences in adverse cardiovascular outcomes using log rank sums. For the four groups of interest we assessed associations with adverse cardiovascular outcomes using Cox proportional hazard regression and the Efron method for ties[36]. Models were applied in a staged process in line with previous studies[3-5] and reported as unadjusted (model one), partially adjusted (model two) and fully adjusted (model three). Model two adjusted for sociodemographic factors (age, sex, Townsend score, age of leaving full time education and ethnicity) and model three additionally adjusted for health and lifestyle factors (diabetes, hypercholesterolemia, BMI, smoking history, alcohol use, systolic BP, sedentary hours per day, physical activity and psychotropic medication use). The proportionality of hazard assumption was assessed using Schoenfeld residuals[37]. We compared our fully adjusted models with results from competing risk analyses using the Fine and Grey approach[38], incorporating non-cardiovascular deaths as a competing event for cardiovascular events, and non-stroke deaths for stroke events. The relative excess risk due to interaction (RERI)[39] was calculated to assess for additivity in the risk. This was done at each month where the proportionality assumption for the variables of interest was not met. All analyses were performed with Stata statistical software, version 12[40] with the exception of RERI which was calculated using the Microsoft Excel method of Andersson and colleagues, which allows for comparison of adjusted outcomes[41].

Psychotropic medication use was included as a confounding variable because of reports that they may increase risk of mortality[42] but we also conducted a sensitivity analysis which excluded

For peer review only - http://bmjopen.bmj.com/site/about/guidelines.xhtml

participants who were taking psychotropic medication. Sub-group analyses looking separately at hazard ratios (HR) in male and female groups only was also carried out to assess for any gender specific differences in light of differing rates of depression and adverse cardiovascular events in each gender[43 27].

Time-varying coefficients.

In the context of Schoenfeld residuals showing non-proportionality, models with time varying coefficients were used. In addition, log(-log) plots were carried out to find the time point at which the proportionality assumption failed. Following this, the data will be stratified by time at this time point, effectively creating two separate survival analyses pre and post the failure time point.

Patient involvement

Although patients were not directly involved with the design of the specific research questions in this study, the hypotheses tested were developed in the context of clinical experience that depression and hypertension may interact to impact on CVD. UK Biobank has an active and ongoing programme of participant involvement: www.ukbiobank.ac.uk/participants/. The outcome measures used were those provided by the UK Biobank data collection protocol, the design of which had input from participants. UK Biobank also has a website and social media streams to disseminate research findings and hosts an annual scientific meeting, which includes cohort participants.

RESULTS

The final sample for adverse cardiovascular outcome included 134,860 participants followed for a median duration of 63 months (702,901.6 person-years follow-up, mean 62.5 months). Table 1 describes the baseline characteristics of the four groups. In general, the hypertension only and comorbid hypertension plus MDD groups were older, had higher BMI and were more likely to have diabetes and hypercholesterolemia. The MDD only and comorbid hypertension plus MDD groups

For peer review only - http://bmjopen.bmj.com/site/about/guidelines.xhtml

had a higher proportion of women and were more likely to be current smokers (table 1). Gender-separated descriptive tables are shown in the supplementary content (Supplementary tables 1 and 2).

The sample for stroke-specific outcomes included 141,754 participants followed for a median duration of 63 months (735247.7 person-years follow-up, mean 62.2 months). Table 2 describes the baseline characteristics of the four groups which display similar characteristics to the adverse CVD outcome groups. Gender-separated descriptive tables are shown in the supplementary content (Supplementary tables 3 and 4).

Adverse cardiovascular outcomes

Within the main analysis and the female only subgroup analysis, MDD failed the proportional hazards assumption. Table 3 presents unadjusted and multivariate-adjusted Hazard ratios (aHR) for adverse cardiovascular outcomes. In the fully adjusted model, relative to the comparator group, the aHR for adverse cardiovascular outcomes was significantly raised for hypertension only (aHR=1. 36, 95%CI 1.22-1.52) and higher still for comorbid hypertension plus MDD (aHR=1.66, 95%CI 1.46-1.9) but reduced for MDD only (aHR=0.55, 95%CI 0.46-0.76). Although this was noted to increase over time as a time-varying coefficient. With the exception of MDD, these findings were robust to sensitivity-analysis excluding those on psychotropic medication (sensitivity analysis aHR=1.43, 95%CI 1.27-1.62; aHR=1.72, 95%CI 1.49-1.999, aHR=0.74, 95%CI 0.52-1.06 respectively). Table 4 presents HRs and aHRs for adverse

cardiovascular outcomes using the hypertension only group as comparator. In the fully adjusted model, relative to hypertension, the aHR for adverse cardiovascular outcomes was significantly raised for comorbid hypertension plus MDD (aHR=1.22, 95%CI 1.1-1.35, sensitivity-analysis aHR= 1.20, 95%CI 1.08-1.34). An adjusted survival plot is shown in figure 1.

For peer review only

For peer review only - http://bmjopen.bmj.com/site/about/guidelines.xhtml

Within the sub-analysis, the male-only model showed a significant increase in hazard ratio for hypertension (male aHR 1.29, 95% CI 1.13-1.47) (table 5 of the supplementary digital content) and comorbid MDD and hypertension (male aHR 1.47, 95%CI 1.24-1.74). However, the difference between comorbid disease and hypertension only was not statistically significant (aHR 1.14, 95%CI 0.995-1.3). The female only sub-analysis showed an increase in hazard ratio for hypertension (aHR 1.64, 95%CI 1.33-2.02) and a greater increase in comorbid MDD and hypertension (aHR 2.18, 95%CI 1.82-2.92) (table 6 of the supplementary content). The difference between comorbid disease and hypertension only was also statistically significant (aHR 1.33, 95%CI 1.14- 1.56). Sensitivity analysis supported these findings.

Stroke Outcomes

None of the independent variables for stroke outcome failed the proportionality assumption. Table 5 presents HRs and aHRs for stroke outcomes. In the fully adjusted model, the aHR for stroke was insignificantly raised for hypertension only (aHR=1.21, 95%CI 0.97-1.51) and depression only (aHR=1.20, 95%CI 0.89-1.63) but significantly raised for comorbid hypertension plus MDD (aHR=1.37, 95%CI 1.04-1.79). In the hypertension comparator group, no group was significantly different from hypertension only (table 6). Similar trends were shown in the gender subset analysis but not reaching significance (Tables 7-8 in supplementary digital content). An adjusted survival plot is shown in figure 2. Again, all results were supported by sensitivity analysis excluding those on psychotropic medication.

Relative excess risk due to interaction, time stratified analysis and competing risk analysis

Survival analysis stratified by time is described and included within the supplementary content (supplementary table 9 and 10 and figure 3). There was evidence of an additive interaction between hypertension and MDD at baseline for the overall analysis before the 22.5 month time point (RERI=0.563, 95%CI 0.189 - 0.938). However, after this time point there was no evidence of

For peer review only - http://bmjopen.bmj.com/site/about/guidelines.xhtml

265 interaction. Table 11 in the supplementary digital content shows the full results for this analysis.
266 Competing risk analysis showed no significant difference from the main analyses for cardiovascular
267 outcomes or stroke outcomes (tables 7-8)

268

269 DISCUSSION

270 In this large population cohort of middle-aged adults without CVD (adjusted for a broad range of
271 confounders), individuals with co-morbid hypertension and MDD were at increased risk of adverse
272 cardiovascular events when compared to those with hypertension alone, MDD alone and neither
273 condition. There was some evidence of an additive effect between hypertension and MDD at
274 baseline, but not throughout follow-up or within subgroup analyses. Differences between co-
275 morbid disease and either disease alone or no disease were more marked in females. For stroke
276 outcomes, comorbid depression and hypertension was the only group that showed significantly
277 increased hazard ratios.

278 Previous research

279 Our findings expand upon previous research from UK Biobank looking at cardiovascular diseases in
280 those with bipolar disorder and MDD[28]. It was found that there were significantly increased odds of
281 having 'any CVD' (fully adjusted OR 1.15 CI 1.12–1.19) or hypertension (fully adjusted OR 1.15 CI
282 1.13–1.18) if depressed, with even higher odds for stroke (fully adjusted OR 1.26 CI 1.13–1.40).
283 There are distinct differences between our current paper and the previous publication. Follow-up
284 data within UK-Biobank has been released to allow meaningful prospective studies be conducted.
285 Thus, the current paper has the benefits of using hospital records and death certification for
286 outcomes, rather than self-reported data. We are also able to make inferences about the direction
287 of effect regarding MDD and CVD and assess the influence of hypertension and MDD over time, both

For peer review only - http://bmjopen.bmj.com/site/about/guidelines.xhtml

in isolation and when comorbid, and assess for statistical interaction to inform on whether there may be a biological interaction.

Other survival analyses in hypertension/MDD comorbidity have focussed primarily on mortality outcomes. In the National Health and Nutrition Epidemiologic Follow-up Study in the United States[31] and the Taiwanese Survey of Health and Living Status[32], individuals with self-reported hypertension plus depressive symptoms (compared to a reference group with neither) had increased all-cause mortality (aHR=1.39, 95%CI 1.14-1.69, aHR=1.54, 95%CI 1.29-1.83, respectively) [3 4] with the former also showing increased CVD specific mortality (aHR=1.59, 95%CI 1.08-2.34)[4]. Similarly, Hamer and colleagues[5] reported a prospective analysis of common mental disorder on mortality outcomes in individuals with hypertension versus those without hypertension in participants from the Health Survey for England and the Scottish Health Survey (1994–2004), finding that risk of CVD death was highest in the group with comorbid disease.

Strengths

These observations are broadly consistent with our results but our study has a number of methodological advantages, including a very large sample size, adjustment of analyses for a more comprehensive range of confounders, and a focus on first-episode non-fatal and fatal adverse cardiovascular events. We also used a definition of prior MDD history which was based on diagnostic criteria within ICD-10 (rather than a threshold score on a depressive symptoms or general wellbeing scale) and our composite definition of hypertension incorporated past history, baseline medication and BP measurements. We believe our lifetime definition to be better suited as it offers a view depression and depressive symptoms over the course of a lifespan as opposed the past week. Also, within our current study we were able to exclude those with previous self-declared or hospital admission CVD, as previous studies show depression may result from cardiovascular disease[44 45] and worsen prognosis[45]

For peer review only - http://bmjopen.bmj.com/site/about/guidelines.xhtml

Limitations

However, some limitations are acknowledged. Recruitment criteria for UK Biobank may lead to selection bias. Specifically, age-restrictions may lead to underrepresentation of early-onset hypertension and those with more severe MDD may be less inclined to attend for assessment. We also acknowledge limitations with our classifications of MDD and hypertension, which were primarily self-report rather than formal diagnostic assessments. Although we have excluded prior cardiovascular events where possible, the MDD plus hypertension sub-type may capture older individuals with a degree of vascular depression, which has an established association with raised BP[46]. In addition, although we adjust for a host of risk factors at baseline such as smoking status, BMI and psychotropic medication, we are limited by the lack of follow-up data, which could show change and modification of said risk factors over time. Similarly, we were unable to assess for medication adherence and transitions from one investigatory group to another. Such modifications could explain the non-proportional nature of the depression group, which may in itself be a predictor of poor medication adherence[47]. Although adherence to medication was not formally assessed, the number and duration of antihypertensive medications used in the hypertension plus MDD group was the same as for the hypertension only group (supplementary content, table 12). As such, worse outcomes in the MDD plus hypertension group are not explained by less intensive antihypertensive treatment at baseline. The end-points used for stroke and cardiovascular events also require to be further validated, however are in line with previous epidemiological studies[5] and have been suggested in previous papers in UK Biobank[33]. Cardiovascular endpoints have not, to our knowledge, been validated within UKbiobank, however we do not feel that this will bias the results towards any particular group. The amelioration of the aHR suggests other covariates contribute considerably to the risk. This is important in the context of increased rates of diabetes, hypercholesterolemia and obesity along with lower socio-economic status in the hypertension only and comorbid groups and as such it is possible we may be seeing the summation of CV risk factors. Finally, the overall recruitment rate to UK Biobank was low (at around 6%); however, the large final cohort size, the

For peer review only - http://bmjopen.bmj.com/site/about/guidelines.xhtml

depth and diversity of phenotype data collected at baseline, and the wide sociodemographic representation of participants all make our findings highly relevant to UK primary care settings. While UK Biobank participants cannot be used to provide representative disease prevalence and incidence rates, valid assessment of exposure-disease relationships are nonetheless widely generalizable and do not require participants to be representative of the UK population at large[48], although findings will not be generalizable to other countries.

Possible mechanisms

Our finding that a history of MDD, in the context of a current diagnosis of hypertension increased the risk of first-episode CVD is complicated by the time varying risk that MDD conveys to CVD. Sub-sample analysis show this time-varying aspect is gender-specific to females. Within our sample, the MDD group has a slightly reduced BP compared to comparators. Previously, reduced BP has been put forth as being causative of MDD and therefore reducing CVD risk[20], but findings from longitudinal studies are inconsistent with regards to direction of effect[49 50]. Potential menopausal effects are also tempting explanations. Common factors for BP and mood such as neuropeptide Y[51 52] may also influence cardiovascular outcomes. Neuropeptide Y has a complex relationship with oestrogen[53] and both have dampening effect on the SNS[54].

Personality factors may also play a role. MDD correlates highly with neuroticism which, although inconsistent, may be protective of cardiovascular disease[55]. Conscientiousness traits may lead to better outcomes[56] and it is possible that this trait has been selected for within UK Biobank. Despite this early reduced risk, due to the time varying nature of MDD, MDD has increased risk in the latter aspects of the time-stratified analyses for the full and female only analyses (supplementary table 9 and 10). The findings from our study in this context suggest MDDs role as a risk factor for cardiovascular disease and its relationship with blood pressure may be much more complex than initially thought, in particular within female populations.

For peer review only - http://bmjopen.bmj.com/site/about/guidelines.xhtml

We can see in the hypertension only baseline models that comorbid hypertension and depression convey a significantly greater risk than hypertension alone. Individuals with either hypertension or depression may have increased sympathetic stimulation that is increased further in comorbid states leading to worse outcomes[57].

CONCLUSIONS

Overall, our findings may have important implications for routine clinical practice, particularly within primary care settings and further demonstrate the complex relationship between depression and hypertension. Although evidence of an additive interaction is inconsistent, we found that comorbid hypertension and depression conferred greater hazard than hypertension alone for adverse cardiovascular outcomes. This significant finding remained after adjustment for factors such as BMI, smoking status and diabetes and was robust to sensitivity analysis excluding those on psychotropic medication. One possible implication is that clinicians should be more aware of the negative long-term impact on CVD outcomes caused by a history of MDD in the context of hypertension, even in those with no previous history of CVD. Although this work awaits replication and testing in other cohorts and settings, further work in this field may suggest that future iterations of CVD risk prediction tools, such as ASSIGN[58], would benefit from the addition of a question on whether individuals have a past history of MDD, so that they can be offered more intensive support to prevent CVD[59].

For peer review only - http://bmjopen.bmj.com/site/about/guidelines.xhtml

ACKNOWLEDGEMENTS

We are grateful to all participants of the UK Biobank cohort. UK Biobank was established by the Wellcome Trust, the Medical Research Council, Department of Health, Scottish Government and the Northwest Regional Development Agency. It has also had funding from the Welsh Assembly Government and the British Heart Foundation. UK Biobank is hosted by the University of Manchester and supported by the National Health Service (NHS). NG is supported by the Aitchison Family Clinical Research Fellowship at the University of Glasgow and DJS is supported by a Lister Institute Prize Fellowship. JC is supported by the Sackler Trust and the Wellcome Trust. Funding also acknowledged from MRC Mental Health Data Pathfinder Award to DJS (MC_PC_17217).

Footnotes

Authors Statement: Contributors NG, JW, JP, JC, DS, SP and DM, contributed to study design and writing of the manuscript. JP and DM contributed to data acquisition. NG conducted data processing and statistical analyses.

Funding: Authors declare no support from any organisation for the submitted work;

Competing interests: All authors have completed the ICMJE uniform disclosure form at http://www.icmje.org/coi_disclosure.pdf and no financial relationships with any organisations that might have an interest in the submitted work in the previous three years; no other relationships or activities that could appear to have influenced the submitted work.

Ethics approval: This study has been conducted using UK Biobank data. UK Biobank has received ethics approval from the UK Biobank Research Ethics Committee (ref. 11/NW/0382).

Data sharing statement: The data used in this study are available via a direct application to UK Biobank.

For peer review only

Transparency statement: The lead author affirms that this manuscript is an honest, accurate and transparent account of the study being reported; that no important aspects of the study have been omitted; and that any discrepancies from the study as planned (and, if relevant, registered) have been explained.

COMPETING INTERESTS STATEMENTS

All authors have completed the ICMJE uniform disclosure form at http://www.icmje.org/coi_disclosure.pdf and no financial relationships with any organisations that might have an interest in the submitted work in the previous three years; no other relationships or activities that could appear to have influenced the submitted work.

For peer review only

References

1. Organization. WH. The global burden of disease: 2004 update. Geneva, Switzerland.: WHO press. 2008.
2. Hare DL, Toukhsati SR, Johansson P, et al. Depression and cardiovascular disease: a clinical review. *Eur Heart J* 2014;35(21):1365-72. doi: 10.1093/eurheartj/eht462
3. Kuo PL, Pu C. The contribution of depression to mortality among elderly with self-reported hypertension: analysis using a national representative longitudinal survey. *J Hypertens* 2011;29(11):2084-90. doi: 10.1097/HJH.0b013e32834b59ad [published Online First: 2011/09/22]
4. Axon RN, Zhao Y, Egede LE. Association of depressive symptoms with all-cause and ischemic heart disease mortality in adults with self-reported hypertension. *Am J Hypertens* 2010;23(1):30-7. doi: 10.1038/ajh.2009.199
5. Hamer M, Batty GD, Stamatakis E, et al. The combined influence of hypertension and common mental disorder on all-cause and cardiovascular disease mortality. *J Hypertens* 2010;28(12):2401-6. doi: 10.1097/HJH.0b013e32833e9d7c
6. Jani BD, Cavanagh J, Barry SJ, et al. Relationship Between Blood Pressure Values, Depressive Symptoms, and Cardiovascular Outcomes in Patients With Cardiometabolic Disease. *Journal of clinical hypertension (Greenwich, Conn)* 2016;18(10):1027-35. doi: 10.1111/jch.12813 [published Online First: 2016/04/05]
7. Taylor CB, Youngblood ME, Catellier D, et al. Effects of antidepressant medication on morbidity and mortality in depressed patients after myocardial infarction. *Arch Gen Psychiatry* 2005;62(7):792-8. doi: 10.1001/archpsyc.62.7.792 [published Online First: 2005/07/06]
8. Kearney PM, Whelton M, Reynolds K, et al. Global burden of hypertension: analysis of worldwide data. *Lancet* 2005;365(9455):217-23. doi: 10.1016/S0140-6736(05)17741-1
9. Lawes CM, Vander Hoorn S, Rodgers A, et al. Global burden of blood-pressure-related disease, 2001. *Lancet* 2008;371(9623):1513-8. doi: 10.1016/S0140-6736(08)60655-8
10. Meng L, Chen D, Yang Y, et al. Depression increases the risk of hypertension incidence: a meta-analysis of prospective cohort studies. *J Hypertens* 2012;30:842 - 51.
11. Wu EL, Chien IC, Lin CH, et al. Increased risk of hypertension in patients with major depressive disorder: a population-based study. *J Psychosom Res* 2012;73(3):169-74. doi: 10.1016/j.jpsychores.2012.07.002
12. Li Z, Li Y, Chen L, et al. Prevalence of Depression in Patients With Hypertension: A Systematic Review and Meta-Analysis. *Medicine (Baltimore)* 2015;94(31):e1317. doi: 10.1097/md.0000000000001317 [published Online First: 2015/08/08]
13. Johnson AD, Newton-Cheh C, Chasman DI, et al. Association of hypertension drug target genes with blood pressure and hypertension in 86,588 individuals. *Hypertension* 2011;57(5):903-10. doi: 10.1161/HYPERTENSIONAHA.110.158667
14. Casamassima F HJ, Fava M, Sachs GS, Smoller JW, Cassano GB, Lattanzi L, Fagerness J, Stange JP, Perlis RH. Phenotypic effects of a bipolar liability gene among individuals with major depressive disorder. . *Am J Med Genet B Neuropsychiatr Genet* 2010;153B:303-09.
15. Green EK, Grozeva D, Jones I, et al. The bipolar disorder risk allele at CACNA1C also confers risk of recurrent major depression and of schizophrenia. *Molecular Psychiatry* 2010;15(10):1016-22. doi: 10.1038/mp.2009.49
16. Ferreira MA, O'Donovan MC, Meng YA, et al. Collaborative genome-wide association analysis supports a role for ANK3 and CACNA1C in bipolar disorder. *Nat Genet* 2008;40(9):1056-8. doi: 10.1038/ng.209
17. Consortium WTCC. Identification of risk loci with shared effects on five major psychiatric disorders: a genome-wide analysis. *The Lancet* 2013;381(9875):1371-79.

For peer review only - http://bmjopen.bmj.com/site/about/guidelines.xhtml

18. Scalco AZ, Scalco MZ, Azul JB, et al. Hypertension and depression. *Clinics (Sao Paulo)* 2005;60(3):241-50. doi: /S1807-59322005000300010
19. Boal AH, Smith DJ, McCallum L, et al. Monotherapy With Major Antihypertensive Drug Classes and Risk of Hospital Admissions for Mood Disorders. *Hypertension* 2016;68(5):1132-38. doi: 10.1161/hypertensionaha.116.08188 [published Online First: 2016/10/14]
20. Licht CM, de Geus EJ, Seldenrijk A, et al. Depression is associated with decreased blood pressure, but antidepressant use increases the risk for hypertension. *Hypertension* 2009;53(4):631-8. doi: 10.1161/HYPERTENSIONAHA.108.126698
21. Crookes DM, Demmer RT, Keyes KM, et al. Depressive Symptoms, Antidepressant Use, and Hypertension in Young Adulthood. *Epidemiology (Cambridge, Mass)* 2018;29(4):547-55. doi: 10.1097/ede.0000000000000840 [published Online First: 2018/04/10]
22. Diminic-Lisica I, Popovic B, Rebic J, et al. Outcome of treatment with antidepressants in patients with hypertension and undetected depression. *Int J Psychiatry Med* 2014;47(2):115-29. doi: 10.2190/PM.47.2.c [published Online First: 2014/08/03]
23. Sudlow C, Gallacher J, Allen N, et al. UK biobank: an open access resource for identifying the causes of a wide range of complex diseases of middle and old age. *PLoS Med* 2015;12(3):e1001779. doi: 10.1371/journal.pmed.1001779
24. Ferrari AJ, Charlson FJ, Norman RE, et al. Burden of depressive disorders by country, sex, age, and year: findings from the global burden of disease study 2010. *PLoS Med* 2013;10(11):e1001547. doi: 10.1371/journal.pmed.1001547
25. Ong KL, Cheung BM, Man YB, et al. Prevalence, awareness, treatment, and control of hypertension among United States adults 1999-2004. *Hypertension* 2007;49(1):69-75. doi: 10.1161/01.hyp.0000252676.46043.18 [published Online First: 2006/12/13]
26. Egan BM, Zhao Y, Axon RN. US trends in prevalence, awareness, treatment, and control of hypertension, 1988-2008. *Jama* 2010;303(20):2043-50. doi: 10.1001/jama.2010.650 [published Online First: 2010/05/27]
27. Smith DJ, Nicholl BI, Cullen B, et al. Prevalence and characteristics of probable major depression and bipolar disorder within UK biobank: cross-sectional study of 172,751 participants. *PLoS One* 2013;8(11):e75362. doi: 10.1371/journal.pone.0075362
28. Martin DJ, Ul-Haq Z, Nicholl BI, et al. Cardiometabolic disease and features of depression and bipolar disorder: population-based, cross-sectional study. *Br J Psychiatry* 2016;208(4):343-51. doi: 10.1192/bjp.bp.114.157784
29. Ul-Haq Z, Smith DJ, Nicholl BI, et al. Gender differences in the association between adiposity and probable major depression: a cross-sectional study of 140,564 UK Biobank participants. *BMC psychiatry* 2014;14:153-53. doi: 10.1186/1471-244X-14-153
30. Sarkar C, Webster C, Gallacher J. Residential greenness and prevalence of major depressive disorders: a cross-sectional, observational, associational study of 94 879 adult UK Biobank participants. *The Lancet Planetary Health* 2018;2(4):e162-e73. doi: https://doi.org/10.1016/S2542-5196(18)30051-2
31. Hall LS, Adams MJ, Arnau-Soler A, et al. Genome-wide meta-analyses of stratified depression in Generation Scotland and UK Biobank. *Translational psychiatry* 2018;8(1):9-9. doi: 10.1038/s41398-017-0034-1
32. Howard DM, Adams MJ, Shirali M, et al. Genome-wide association study of depression phenotypes in UK Biobank identifies variants in excitatory synaptic pathways. *Nature Communications* 2018;9(1):1470. doi: 10.1038/s41467-018-03819-3
33. Woodfield R, Grant I, Group UKBSO, et al. Accuracy of Electronic Health Record Data for Identifying Stroke Cases in Large-Scale Epidemiological Studies: A Systematic Review from the UK Biobank Stroke Outcomes Group. *PLOS ONE* 2015;10(10):e0140533. doi: 10.1371/journal.pone.0140533
34. Townsend P. Deprivation. *Journal of Social Policy* 2009;16(02):125. doi: 10.1017/s0047279400020341

For peer review only - http://bmjopen.bmj.com/site/about/guidelines.xhtml

35. Guo W, Bradbury KE, Reeves GK, et al. Physical activity in relation to body size and composition in women in UK Biobank. *Annals of Epidemiology* 2015;25(6):406-13.e6. doi: http://dx.doi.org/10.1016/j.annepidem.2015.01.015
36. Efron B. The Efficiency of Cox's Likelihood Function for Censored Data. *Journal of the American Statistical Association,* 1977;72(359):557-65.
37. Schoenfeld D. Partial Residuals for the Proportional Hazards Regression-Model. *Biometrika* 1982;69(1):239-41. doi: Doi 10.2307/2335876
38. Fine JP, Gray RJ. A Proportional Hazards Model for the Subdistribution of a Competing Risk. *Journal of the American Statistical Association* 1999;94(446):496-509. doi: 10.2307/2670170
39. Hosmer DW, Lemeshow S. Confidence interval estimation of interaction. *Epidemiology (Cambridge, Mass)* 1992;3(5):452-6. [published Online First: 1992/09/01]
40. Stata Statistical Software, version 12 [program]. College station, Texas.
41. Andersson T, Alfredsson L, Kallberg H, et al. Calculating measures of biological interaction. *European journal of epidemiology* 2005;20(7):575-9. [published Online First: 2005/08/27]
42. Weich S, Pearce HL, Croft P, et al. Effect of anxiolytic and hypnotic drug prescriptions on mortality hazards: retrospective cohort study. *BMJ* 2014;348:g1996. doi: 10.1136/bmj.g1996
43. Nichols M, Townsend N, Scarborough P, et al. Cardiovascular disease in Europe 2014: epidemiological update. *European Heart Journal* 2014;35(42):2950-59. doi: 10.1093/eurheartj/ehu299
44. Kang HJ, Stewart R, Bae KY, et al. Predictive value of homocysteine for depression after acute coronary syndrome. *Oncotarget* 2016;7(42):69032-40. doi: 10.18632/oncotarget.11966
45. Lichtman JH, Froelicher ES, Blumenthal JA, et al. Depression as a risk factor for poor prognosis among patients with acute coronary syndrome: systematic review and recommendations: a scientific statement from the American Heart Association. *Circulation* 2014;129(12):1350-69. doi: 10.1161/CIR.0000000000000019
46. Aizenstein HJ, Baskys A, Boldrini M, et al. Vascular depression consensus report – a critical update. *BMC Medicine* 2016;14(1):161. doi: 10.1186/s12916-016-0720-5
47. Goldstein CM, Gathright EC, Garcia S. Relationship between depression and medication adherence in cardiovascular disease: the perfect challenge for the integrated care team. *Patient preference and adherence* 2017;11:547-59. doi: 10.2147/PPA.S127277
48. Fry A, Littlejohns TJ, Sudlow C, et al. Comparison of Sociodemographic and Health-Related Characteristics of UK Biobank Participants With Those of the General Population. *American journal of epidemiology* 2017;186(9):1026-34. doi: 10.1093/aje/kwx246 [published Online First: 2017/06/24]
49. Hildrum B, Mykletun A, Holmen J, et al. Effect of anxiety and depression on blood pressure: 11-year longitudinal population study. *British Journal of Psychiatry* 2018;193(2):108-13. doi: 10.1192/bjp.bp.107.045013 [published Online First: 01/02]
50. Paterniti S. Low blood pressure and risk of depression in the elderly: A prospective community-based study. *The British Journal of Psychiatry* 2000;176(5):464-67. doi: 10.1192/bjp.176.5.464
51. Zhang P, Qi Y-X, Yao Q-P, et al. Neuropeptide Y Stimulates Proliferation and Migration of Vascular Smooth Muscle Cells from Pregnancy Hypertensive Rats via Y1 and Y5 Receptors. *PLOS ONE* 2015;10(7):e0131124. doi: 10.1371/journal.pone.0131124
52. Morales-Medina JC, Dumont Y, Quirion R. A possible role of neuropeptide Y in depression and stress. *Brain research* 2010;1314:194-205. doi: https://doi.org/10.1016/j.brainres.2009.09.077
53. Pelletier G, Li S, Luu-The V, et al. Oestrogenic Regulation of Pro-Opiomelanocortin, Neuropeptide Y and Corticotrophin-Releasing Hormone mRNAs in Mouse Hypothalamus. *Journal of Neuroendocrinology* 2007;19(6):426-31. doi: 10.1111/j.1365-2826.2007.01548.x
54. Rosano GM, Panina G. Oestrogens and the heart. *Therapie* 1999;54(3):381-5. [published Online First: 1999/09/29]

For peer review only

For peer review only - http://bmjopen.bmj.com/site/about/guidelines.xhtml

55. Gale CR, Čukić I, Batty GD, et al. When Is Higher Neuroticism Protective Against Death? Findings From UK Biobank. *Psychological Science* 2017;28(9):1345-57. doi: 10.1177/0956797617709813
56. Cheng H, Montgomery S, Treglown L, et al. Emotional stability, conscientiousness, and self-reported hypertension in adulthood. *Personality and Individual Differences* 2017;115:159-63. doi: https://doi.org/10.1016/j.paid.2016.02.034
57. Barton DA, Dawood T, Lambert EA, et al. Sympathetic activity in major depressive disorder: identifying those at increased cardiac risk? *J Hypertens* 2007;25(10):2117-24. doi: 10.1097/HJH.0b013e32829baae7 [published Online First: 2007/09/22]
58. Woodward M, Brindle P, Tunstall-Pedoe H. Adding social deprivation and family history to cardiovascular risk assessment: the ASSIGN score from the Scottish Heart Health Extended Cohort (SHHEC). *Heart* 2007;93(2):172-76. doi: 10.1136/hrt.2006.108167
59. Hippisley-Cox J, Coupland C, Vinogradova Y, et al. Predicting cardiovascular risk in England and Wales: prospective derivation and validation of QRISK2. *BMJ* 2008;336(7659):1475-82. doi: 10.1136/bmj.39609.449676.25

For peer review only

For peer review only - http://bmjopen.bmj.com/site/about/guidelines.xhtml

Table 1. Baseline characteristics for adverse cardiovascular outcomes

	Comparator group		Hypertension only		MDD only		Hypertension plus MDD	
	N = 50798		N = 56035		N = 15098		N = 12929	
**Median age (range)***	54	(47 - 61)	61	(55 - 65)	53	(46 - 60)	60	(53 - 64)
**Females, N (%)**	29228	(57.54%)	25893	(46.21%)	10929	(72.39%)	7676	(59.37%)
**Ethnicity, N (%)**								
White	46147	(90.84%)	51249	(91.46%)	14247	(94.36%)	12272	(94.92%)
Asian/Asian British	1771	(3.49%)	1696	(3.03%)	261	(1.73%)	179	(1.38%)
Black/ Black British	1323	(2.6%)	1769	(3.16%)	219	(1.45%)	222	(1.72%)
**Median Townsend score (range)***	-1.89	(-3.45 - 0.54)	-2.07	(-3.51 - 0.39)	-1.64	(-3.3 - 0.93)	-1.84	(-3.42 - 0.76)
**Age at leaving full-time education, N (%)**								
<16	5916	(11.65%)	12085	(21.57%)	1725	(11.43%)	2607	(20.16%)
16	10265	(20.21%)	11827	(21.11%)	3178	(21.05%)	2732	(21.13%)
>16	34090	(67.11%)	31480	(56.18%)	10090	(66.83%)	7503	(58.03%)
**Total physical activity in metabolic**	3.97	(1.68 - 8.03)	3.79	(1.51 - 8.03)	3.89	(1.66 - 8)	3.68	(1.49 - 7.95)
**Sedentary time in hours, median (range)***	4	(3 - 6)	4.5	(3.5 - 6)	4.5	(3 - 6)	5	(3.5 - 6)

For peer review only - http://bmjopen.bmj.com/site/about/guidelines.xhtml

**Diabetes, N (%)**	1268	(2.5%)	3777	(6.74%)	380	(2.52%)	929	(7.19%)
**Hypercholesterolaemia, N (%)**	3011	(5.93%)	9210	(16.44%)	893	(5.91%)	2211	(17.1%)
**Systolic BP in mmHg, median (range)***	125.5	(118 - 132)	149.5	(142 - 159.5)	124	(116 - 131)	147.5	(140.5 - 157.)
**Body Mass Index, N (%)**								
<18.5	389	(0.77%)	142	(0.25%)	103	(0.68%)	34	(0.26%)
18.5 – 25	22549	(44.39%)	13678	(24.41%)	6251	(41.4%)	2874	(22.23%)
25-30	20410	(40.18%)	25216	(45 %)	5936	(39.32%)	5389	(41.68%)
>30	7450	(14.67%)	16999	(30.34%)	2808	(18.6%)	4632	(35.83%)
**Smoking status, N (%)**								
Never smoked	30626	(60.29%)	31503	(56.22%)	7864	(52.09%)	6454	(49.92%)
Previously smoked	15056	(29.64%)	20140	(35.94%)	5118	(33.9%)	5065	(39.18%)
Current smoker	4970	(9.78%)	4199	(7.49%)	2093	(13.86%)	1381	(10.68%)
**Alcohol frequency, N (%)**								
Daily or almost daily	9450	(18.6%)	12970	(23.15%)	2736	(18.12%)	2881	(22.28%)
Three or four times a week	12175	(23.97%)	13033	(23.26%)	3253	(21.55%)	2837	(21.94%)
Once or twice a week	13644	(26.86%)	13889	(24.79%)	3880	(25.7%)	2916	(22.55%)

For peer review only - http://bmjopen.bmj.com/site/about/guidelines.xhtml

One to three times a month	6052	(11.91%)	5588	(9.97%)	2058	(13.63%)	1512	(11.69%)
Special occasions only	5534	(10.89%)	6330	(11.3%)	1904	(12.61%)	1729	(13.37%)
Never	3924	(7.72%)	4199	(7.49%)	1262	(8.36%)	1048	(8.11%)
**Psychotropic medication, N (%)**	1341	(2.64%)	1795	(3.2%)	2844	(18.84%)	2522	(19.51%)

*All data presented as N (%) and has chi-squared p-value of <0.001 except * which are median values (interquartile range) and have a Kruskal-Wallis p-value of 0.0001. Data presented as MET-hrs (hours spent doing exercise adjusted for multiples of basal metabolic rate in accordance with IPAQ).Townsend score is an area based measure based on census statistics. It is a calculation based on the number of: households without a car, overcrowded households, households not owner-occupied and unemployment.*

For peer review only

Table 2 Baseline characteristics for stroke outcomes

	Comparator group		Hypertension only		MDD only		Hypertension plus MDD	
	N = 52502		N = 59724		N = 15581		N = 13947	
**Median age (range)***	54	(47 - 61)	61	(55 - 65)	54	(47 - 61)	60	(53 - 64)
**Females, N (%)**	29684	(56.54%)	26937	(45.1%)	11143	(71.52%)	8090	(58.01%)
**Ethnicity, N (%)**								
White	47697	(90.85%)	54578	(91.38%)	14697	(94.33%)	13212	(94.73%)
Asian/Asian British	1857	(3.54%)	1889	(3.16%)	280	(1.8%)	209	(1.5%)
Black/ Black British	1355	(2.58%)	1854	(3.1%)	223	(1.43%)	246	(1.76%)
**Median Townsend score (range)***	-1.89	(-3.45 - 0.55)	-2.04	(-3.49 - 0.44)	-1.56	(-3.28 - 1.15)	-1.74	(-3.4 - 0.93)
**Age at leaving full-time education, N (%)**								
<16	6446	(12.28%)	13396	(22.43%)	1884	(12.09%)	2945	(21.12%)
16	10590	(20.17%)	12507	(20.94%)	3270	(20.99%)	2953	(21.17%)
>16	34914	(66.5%)	33114	(55.45%)	10317	(66.22%)	7947	(56.98%)
**Total physical activity in metabolic**	3.96	(1.67 - 8.02)	3.75	(1.5 - 8)	4.13	(1.67 - 8.36)	3.66	(1.45 - 7.83)
**Sedentary time in hours, median (range)***	4	(3 - 6)	5	(3.5 - 6)	5	(3.5 - 6.5)	5	(4 - 7)

For peer review only - http://bmjopen.bmj.com/site/about/guidelines.xhtml

**Diabetes, N (%)**	1454	(2.77%)	4502	(7.54%)	449	(2.88%)	1163	(8.34%)
**Hypercholesterolaemia, N (%)**	3592	(6.84%)	10768	(18.03%)	1049	(6.73%)	2620	(18.79%)
**Systolic BP in mmHg, median (range)***	125.5	(118 - 132)	149.5	(142 - 159.5)	127	(120.5 - 133)	147.5	(140.5 - 156.5)
**Body Mass Index, N (%)**								
<18.5	395	(0.75%)	151	(0.25%)	104	(0.67%)	38	(0.27%)
18.5 – 25	22967	(43.75%)	14242	(23.85%)	6374	(40.91%)	3017	(21.63%)
25-30	21185	(40.35%)	26817	(44.9%)	6149	(39.46%)	5769	(41.36%)
>30	7953	(15.15%)	18514	(31.%)	2954	(18.96%)	5123	(36.73%)
**Smoking status, N (%)**								
Never smoked	31318	(59.65%)	32982	(55.22%)	8052	(51.68%)	6834	(49%)
Previously smoked	15851	(30.19%)	22019	(36.87%)	5340	(34.27%)	5560	(39.87%)
Current smoker	5170	(9.85%)	4501	(7.54%)	2163	(13.88%)	1519	(10.89%)
**Alcohol frequency, N (%)**								
Daily or almost daily	9760	(18.59%)	13751	(23.02%)	2817	(18.08%)	3085	(22.12%)
Three or four times a week	12563	(23.93%)	13827	(23.15%)	3335	(21.4%)	3020	(21.65%)
Once or twice a week	14089	(26.84%)	14719	(24.65%)	3993	(25.63%)	3125	(22.41%)

For peer review only - http://bmjopen.bmj.com/site/about/guidelines.xhtml

One to three times a month	6220	(11.85%)	5971	(10%)	2122	(13.62%)	1627	(11.67%)
Special occasions only	5744	(10.94%)	6794	(11.38%)	1978	(12.69%)	1885	(13.52%)
Never	4102	(7.81%)	4630	(7.75%)	1330	(8.54%)	1199	(8.6%)
**Psychotropic medication, N (%)**	1408	(2.68%)	1996	(3.34%)	2976	(19.1%)	2778	(19.92%)

*All data presented as N (%) and has chi-squared p-value of <0.001 except * which are median values (interquartile range) and have a Kruskal-Wallis p-value of 0.0001. Data presented as MET-hrs (hours spent doing exercise adjusted for multiples of basal metabolic rate in accordance with IPAQ).Townsend score is an area based measure based on census statistics. It is a calculation based on the number of: households without a car, overcrowded households, households not owner-occupied and unemployment.*

For peer review only

Table 3: Risk of adverse cardiovascular event by clinical group: unadjusted, partially adjusted and fully adjusted models.

	Unadjusted			Model 1 - Sociodemographic			Model 2 - Model 1 + Health/ Lifestyle		
**Group**	HR	95% C.I.	p-value	aHR	95% C.I.	p-value	aHR	95% C.I.	p-value
No Hypertension - No MDD	1(ref)			1(ref)			1(ref)		
Hypertension only	2.60	(2.39 - 2.82)	$3.31x10^{-113}$	1.72	(1.57 - 1.88)	$1.99x10^{-33}$	1.36	(1.22 - 1.52)	$2.92x10^{-8}$
MDD only	0.69	(0.51 - 0.94)	0.02	0.82	(0.6 - 1.13)	0.23	0.75	(0.54 - 1.04)	0.08
Hypertension and MDD	2.84	(2.55 - 3.17)	$6.31x10^{-77}$	2.27	(2.02 - 2.55)	$2.75x10^{-44}$	1.66	(1.45 - 1.9)	$7.48x10^{-14}$
**Time varying Variables**									
MDD only	1.01	(1.004 - 1.02)	$2.38x10^{-3}$	1.01	(1.004 - 1.02)	$3.19x10^{-3}$	1.01	(1.004 - 1.02)	$3.03x10^{-3}$

**Adjusted for sociodemographic factors (age, sex, Townsend score, age of leaving full time education and ethnicity. †Additionally adjusted for history of diabetes, history of hypercholesterolemia, BMI, smoking history, alcohol use, systolic blood pressure, sedentary hours per day, physical activity and psychotropic medication use. MDD = Major depressive disorder, HR = Hazard ratio, aHR = Adjusted hazard ratio, C.I. = Confidence interval.*

For peer review only

Table 4: Risk of adverse cardiovascular event by clinical group: unadjusted, partially adjusted and fully adjusted models with hypertension as the comparator

	Unadjusted			Model 1 - Sociodemographic			Model 2 - Model 1 + Health/ Lifestyle		
**Group**	HR	95% C.I.	p-value	aHR	95% C.I.	p-value	aHR	95% C.I.	p-value
Hypertension only	1(ref)			1(ref)			1(ref)		
No Hypertension - No MDD	0.38	(0.35 - 0.42)	3.31×10^{-113}	0.58	(0.53 - 0.63)	1.99×10^{-33}	0.73	(0.66 - 0.82)	2.92×10^{-8}
MDD only	0.27	(0.2 - 0.36)	1.14×10^{-17}	0.48	(0.35 - 0.66)	4.91×10^{-6}	0.55	(0.4 - 0.76)	3.23×10^{-4}
Hypertension and MDD	1.09	(0.996 - 1.2)	0.06	1.32	(1.2 - 1.46)	3.07×10^{-8}	1.22	(1.1 - 1.35)	1.30×10^{-4}
**Time varying Variables**									
MDD only	1.01	(1.004 - 1.02)	0.002	1.01	(1.004 - 1.02)	3.19×10^{-3}	1.01	(1.004 - 1.02)	3.03×10^{-3}

**Adjusted for sociodemographic factors (age, sex, Townsend score, age of leaving full time education and ethnicity. †Additionally adjusted for history of diabetes, history of hypercholesterolemia, BMI, smoking history, alcohol use, systolic blood pressure, sedentary hours per day, physical activity and psychotropic medication use. MDD = Major depressive disorder, HR = Hazard ratio, aHR = Adjusted hazard ratio, C.I. = Confidence interval.*

For peer review only

For peer review only - http://bmjopen.bmj.com/site/about/guidelines.xhtml

Table 5: Risk of stroke event by clinical group: unadjusted, partially adjusted and fully adjusted models.

	Unadjusted			Model 1 - Sociodemographic			Model 2 - Model 1 + Health/ Lifestyle		
**Group**	HR	95% C.I.	p-value	aHR	95% C.I.	p-value	aHR	95% C.I.	p-value
No Hypertension- No MDD	1(ref)			1(ref)			1(ref)		
Hypertension only	2.55	(2.16 - 3.02)	$3.84x10^{-28}$	1.64	(1.38 - 1.96)	$3.35x10^{-8}$	1.21	(0.97 - 1.51)	0.09
MDD only	1.14	(0.86 - 1.52)	0.37	1.37	(1.02 - 1.84)	0.037	1.20	(0.89 - 1.63)	0.24
Hypertension and MDD	2.67	(2.13 - 3.34)	$9.79x10^{-18}$	2.05	(1.63 - 2.58)	$1.08x10^{-9}$	1.37	(1.04 - 1.79)	0.02

**Adjusted for sociodemographic factors (age, sex, Townsend score, age of leaving full time education and ethnicity. †Additionally adjusted for history of diabetes, history of hypercholesterolemia, BMI, smoking history, alcohol use, systolic blood pressure, sedentary hours per day, physical activity and psychotropic medication use. MDD = Major depressive disorder, HR = Hazard ratio, aHR = Adjusted hazard ratio, C.I. = Confidence interval.*

For peer review only - http://bmjopen.bmj.com/site/about/guidelines.xhtml

Table 6: Risk of stroke event by clinical group: unadjusted, partially adjusted and fully adjusted models with hypertension as the comparator

	Unadjusted			Model 1 - Sociodemographic			Model 2 - Model 1 + Health/ Lifestyle		
**Group**	HR	95% C.I.	p-value	aHR	95% C.I.	p-value	aHR	95% C.I.	p-value
Hypertension only	1(ref)			1(ref)			1(ref)		
No Hypertension - No MDD	0.39	(0.33 - 0.46)	$3.84\text{x}10^{-28}$	0.61	(0.51 - 0.73)	$3.35\text{x}10^{-8}$	0.82	(0.66 - 1.03)	0.09
MDD only	0.45	(0.34 - 0.58)	$1.43\text{x}10^{-9}$	0.83	(0.63 - 1.1)	0.19	0.99	(0.73 - 1.35)	0.95
Hypertension and MDD	1.05	(0.86 - 1.27)	0.64	1.25	(1.03 - 1.52)	0.03	1.13	(0.92 - 1.39)	0.26

**Adjusted for sociodemographic factors (age, sex, Townsend score, age of leaving full time education and ethnicity. †Additionally adjusted for history of diabetes, history of hypercholesterolemia, BMI, smoking history, alcohol use, systolic blood pressure, sedentary hours per day, physical activity and psychotropic medication use. MDD = Major depressive disorder, HR = Hazard ratio, aHR = Adjusted hazard ratio, C.I. = Confidence interval.*

For peer review only

Table 7: Fully adjusted HR compared with results from competing risks analysis for cardiovascular endpoints

	Fully adjusted non-competing risks analysis			Fully adjusted competing risks model		
**Group**	aHR	95% C.I.	p-value	aHR	95% C.I.	p-value
No Hypertension - No MDD	1(ref)			1(ref)		
Hypertension only	1.36	(1.22- 1.52)	2.92×10^{-8}	1.37	(1.22-1.53)	4×10^{-8}
MDD only	0.75	(0.54- 1.04)	0.08	0.76	(0.55-1.03)	0.08
Hypertension and MDD	1.66	(1.45- 1.9)	7.48×10^{-14}	1.67	(1.45-1.91)	2.2×10^{-13}
tvc						
MDD only	1.01	(1.004- 1.02)	3.03×10^{-3}	1.01	(1.004-1.02)	0.003

Adjusted for age, sex, Townsend score, age of leaving full time education and ethnicity, history of diabetes, history of hypercholesterolemia, BMI, smoking history, alcohol use, systolic blood pressure, sedentary hours per day, physical activity and psychotropic medication use. MDD = Major depressive disorder, aHR = Adjusted hazard ratio, C.I. = Confidence interval.

For peer review only - http://bmjopen.bmj.com/site/about/guidelines.xhtml

Table 8: Fully adjusted HR compared with results from competing risks analysis for stroke endpoints

	**Fully adjusted non-competing risks analysis**			**Fully adjusted competing risks model**		
**Group**	aHR	95% C.I.	p-value	aHR	95% C.I.	p-value
No Hypertension - No MDD	1(ref)			1(ref)		
Hypertension only	1.21	(0.97- 1.51)	0.09	1.21	(0.96- 1.52)	0.1
MDD only	1.20	(0.89- 1.63)	0.24	1.20	(0.88- 1.64)	0.25
Hypertension and MDD	1.37	(1.04- 1.79)	0.02	1.36	(1.03- 1.8)	0.031

Adjusted for age, sex, Townsend score, age of leaving full time education and ethnicity, history of diabetes, history of hypercholesterolemia, BMI, smoking history, alcohol use, systolic blood pressure, sedentary hours per day, physical activity and psychotropic medication use. MDD = Major depressive disorder, aHR = Adjusted hazard ratio, C.I. = Confidence interval.

Figure 1: Adjusted survival analysis graph for adverse cardiovascular outcome showing greatest hazard for the comorbid group. MDD appears protective compared to the comparator group initially, however, shows increased hazard after 41 months. Analysis adjusted for age, sex, Townsend score, age of leaving full time education and ethnicity, history of diabetes, history of hypercholesterolemia, BMI, smoking history, alcohol use, systolic blood pressure, sedentary hours per day, physical activity and psychotropic medication use (MDD = Major Depressive disorder)

Figure 2: Adjusted survival analysis graph for stroke outcomes showing significantly increased hazard for comorbid Hypertension and MDD, with similar insignificant increased hazard trends for hypertension only and MDD only. Analysis adjusted for age, sex, Townsend score, age of leaving full time education and ethnicity, history of diabetes, history of hypercholesterolemia, BMI, smoking history, alcohol use, systolic blood pressure, sedentary hours per day, physical activity and psychotropic medication use. (MDD = Major Depressive disorder)

For peer review only

Figure 3: Log (-log) plot showing non-proportionality of MDD only survival over time. Paths between the comparator group and the MDD group cross at the 22.5 month mark. Analysis adjusted for age, sex, Townsend score, age of leaving full time education and ethnicity, history of diabetes, history of hypercholesterolemia, BMI, smoking history, alcohol use, systolic blood pressure, sedentary hours per day, physical activity and psychotropic medication use. (MDD = Major Depressive disorder)

For peer review only

For peer review only - http://bmjopen.bmj.com/site/about/guidelines.xhtml

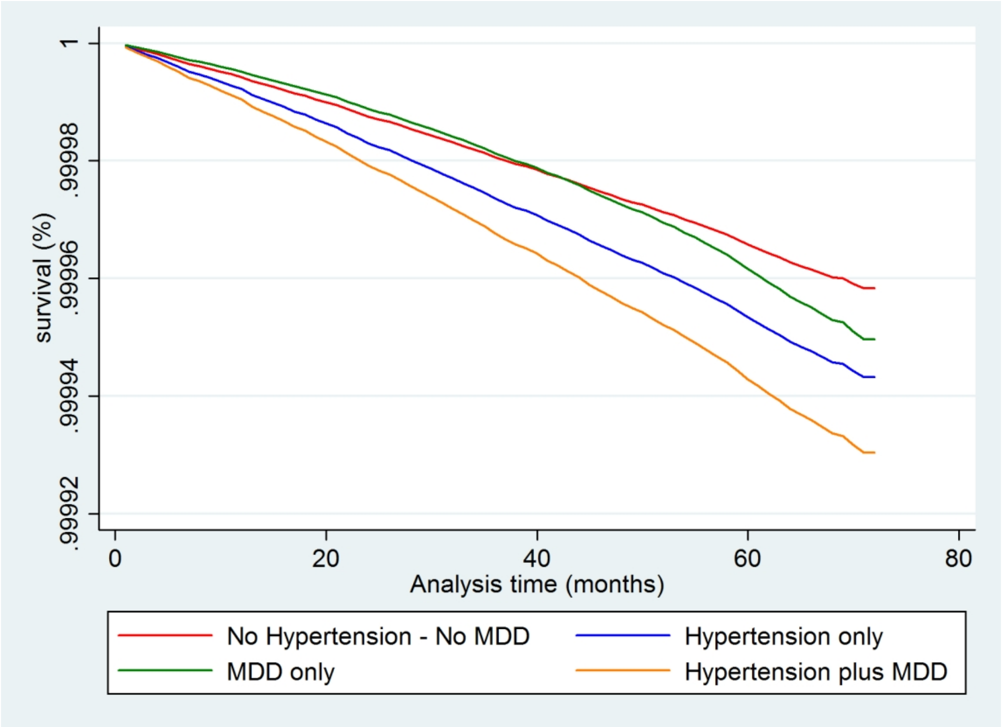

Adjusted survival analysis graph for adverse cardiovascular outcome showing greatest hazard for the comorbid group. MDD appears protective compared to the comparator group initially, however, shows increased hazard after 41 months. Analysis adjusted for age, sex, Townsend score, age of leaving full time education and ethnicity, history of diabetes, history of hypercholesterolemia, BMI, smoking history, alcohol use, systolic blood pressure, sedentary hours per day, physical activity and psychotropic medication use (MDD = Major Depressive disorder)

152x110mm (300 x 300 DPI)

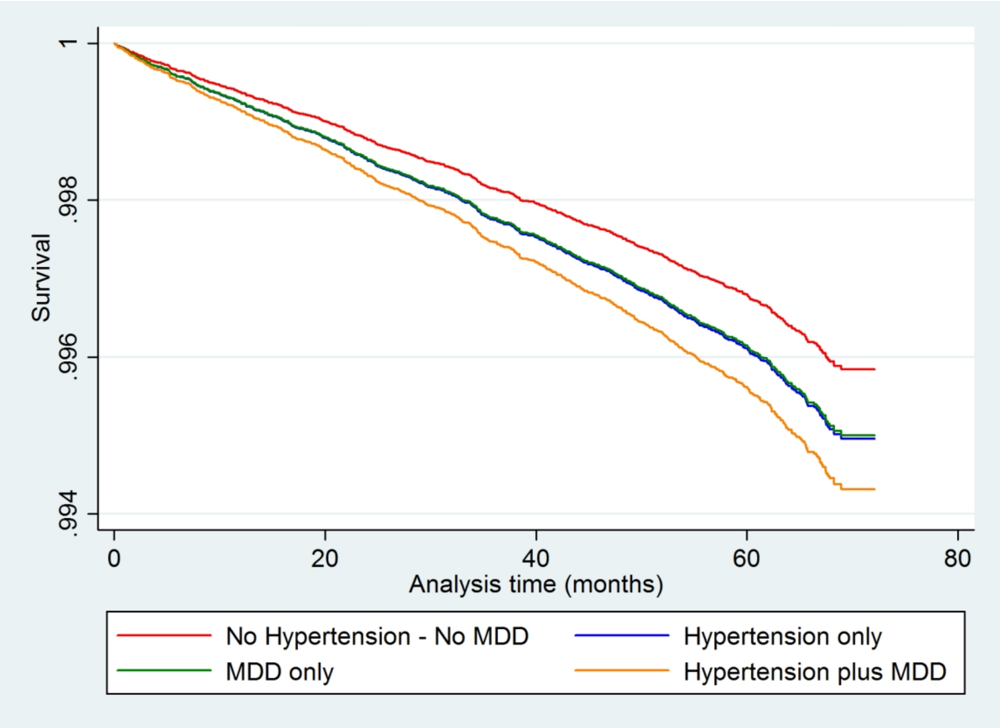

Adjusted survival analysis graph for stroke outcomes showing significantly increased hazard for comorbid Hypertension and MDD, with similar insignificant increased hazard trends for hypertension only and MDD only. Analysis adjusted for age, sex, Townsend score, age of leaving full time education and ethnicity, history of diabetes, history of hypercholesterolemia, BMI, smoking history, alcohol use, systolic blood pressure, sedentary hours per day, physical activity and psychotropic medication use. (MDD = Major Depressive disorder)

152x110mm (300 x 300 DPI)

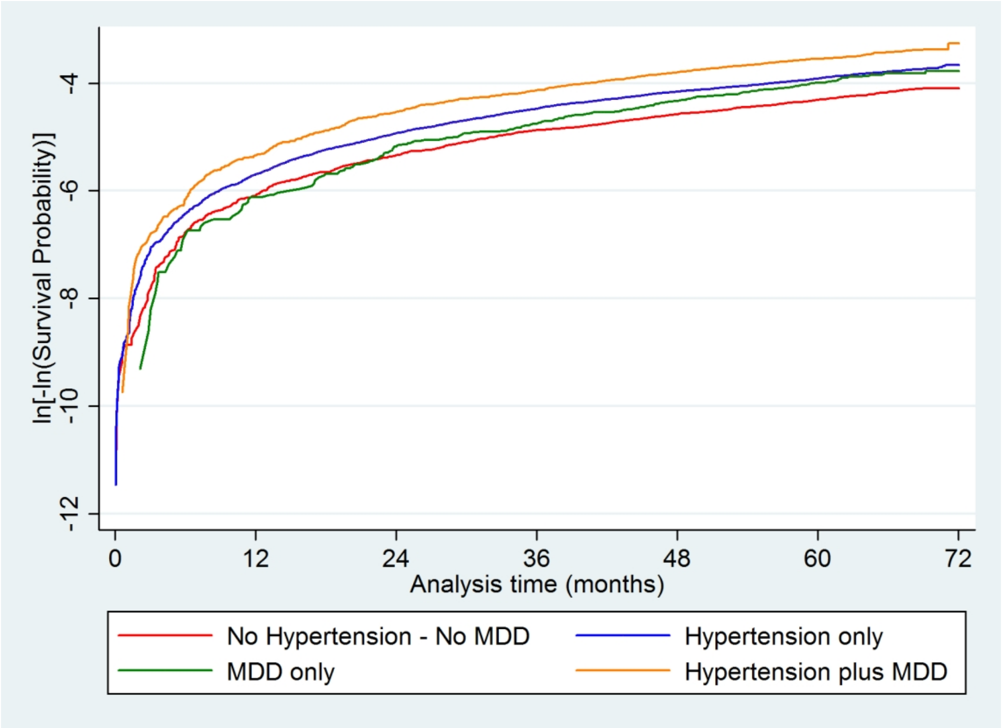

Log (-log) plot showing non-proportionality of MDD only survival over time. Paths between the comparator group and the MDD group cross at the 22.5 month mark. Analysis adjusted for age, sex, Townsend score, age of leaving full time education and ethnicity, history of diabetes, history of hypercholesterolemia, BMI, smoking history, alcohol use, systolic blood pressure, sedentary hours per day, physical activity and psychotropic medication use. (MDD = Major Depressive disorder)

152x110mm (300 x 300 DPI)

Supplementary information for Impact of major depression on cardiovascular outcomes for individuals with hypertension: prospective study in UK Biobank. Graham et al

METHODS

New-onset cardiovascular outcomes

Date and cause of death were obtained from death certificates held by the National Health Service (NHS) Information Centre for participants from England and Wales and the NHS Central Register Scotland for participants from Scotland. Date and cause of hospital admissions were identified via record linkage to Health Episode Statistics (HES) records for England, the Patient Episode Database for Wales (PEDW) and to the Scottish Morbidity Records (SMR) for Scotland. Detailed information about the record linkage procedure is available online [1 2]. At the time of analysis, mortality data were available up to 31st January 2016 for England and Wales and 11th November 2015 for Scotland. Hospital admission data were available for the Scottish, English and Welsh participants until the 31st August 2014, 31st March 2015, and 28th February 2015 respectively. Therefore, for new cardiovascular events, end of follow up was classified as the hospital admission dates unless preceded by the date of death or the date of first cardiovascular event. New onset cardiovascular events were defined as an ICD 10 code of G45, G46, I20- I25, or I6 recorded on a death certificate or hospital admission. Deaths that predated the assessment date were excluded from analysis as presumed errors as were those in which data had only recorded a death date but no cause of death or a cause of death but no death date. Participants that had hospital admissions prior to the assessment date due to the aforementioned ICD10 codes were excluded as were not first episode. In addition, ICD-9 codes 430-438, 410-414, 429 and

For peer review only - http://bmjopen.bmj.com/site/about/guidelines.xhtml

429.2 were also excluded.   hospital records are not available for the entire lifetime of study individuals, potentially missing some early cardiovascular events, as such those with self-declared prior cardiovascular disease at baseline were also excluded.

Blood Pressure

Blood pressure was measured in a sitting position partway through the interview and at the end of the interview using a digital blood pressure monitor (Omron HEM-7015IT.). Full protocol is available online https://biobank.ctsu.ox.ac.uk/crystal/docs/Bloodpressure.pdf

Depression definition

The criteria for lifetime MDD were created via the the following questions via touchscreen questionnaire were: *"Looking back over your life, have you ever had a time when you were feeling depressed or down for at least a whole week?"* (depression)*; "Have you ever had a period of time lasting at least two days when you were so irritable that you found yourself shouting at people or starting fights or arguments?"* (irritability); *"How many weeks was the longest period when you were feeling depressed or down?"* (duration); *"Have you ever seen a general practitioner (GP) for nerves, anxiety, tension or depression?"* (consulted GP); *"Have you ever seen a psychiatrist for nerves, anxiety, tension or depression?"* (consulted psychiatrist). Participants were classified as having a history of MDD if they reported at least one episode which comprised of depression and/or irritability, with a duration of at least two weeks, plus had consulted with either a general practitioner or psychiatrist for mental ill-health.

Physical activity

Physical activity was based on self-report, utilising the short form International Physical Activity Questionnaire (IPAQ). Participants reported the frequency and duration of

For peer review only - http://bmjopen.bmj.com/site/about/guidelines.xhtml

moderate and vigorous activity along with walking undertaken in a typical week [3]. Data were analysed in accordance with the IPAQ scoring protocol [4] and total physical activity was computed as the sum of walking, moderate and vigorous activity, measured as metabolic equivalents (MET-hours/week). Physical activity was used in analyses as a continuous variable. Participants who reported greater than 24 hours a day doing all activity were classified as missing.

Sedentary behaviour

Sedentary behaviour duration was derived from the sum of self-reported time spent driving, using computer and watching television. Those stating that they had performed "less than an hour" of sedentary activities were coded as 0.5hrs to allow use of a continuous variable. Participants who reported greater than 24 hours a day doing all activity were classified as missing.

Socio-demographic and other covariates

Self-report on taking antihypertensive medication was taken from a question specific to cardiovascular medications, where antihypertensive medication was an option to respond. Area-based socioeconomic status was derived from postcode of residence, utilising the census-derived Townsend deprivation index scored on housing, employment, social class and car availability where a negative score represents greater affluence [5 6]. Age was calculated from dates of birth and baseline assessment date. Smoking status was categorised into never, former and current smoking based on self-report, those who wished not to answer were coded as missing. Drink frequency was categorised into daily, three or four times a week, once or twice a week, one to three times a month, special occasions only, and never based on self-report. Those who wished not to answer were coded as

For peer review only - http://bmjopen.bmj.com/site/about/guidelines.xhtml

missing. Medical history of diabetes and high cholesterol was collected from the self-completed, baseline assessment questionnaire of medical conditions. Ethnicity was categorised as Caucasian, black/mixed and Asian/mixed based on self-report. Other ethnicities coded as missing due to small numbers. Age at completing full-time education was categorised as (<16, 16, >16). Height and body weight were measured by trained nurses during the initial assessment centre visit. Body mass index (BMI) was calculated as (weight/height^2) and the WHO criteria[7] to classify BMI into: underweight <18.5, normal weight 18.5-24.9, overweight 25.0-29.9 and obese ≥30.0 kg.m^{-2}. Psychotropic medication use was defined by the presence of pharmaceuticals from British National Formulary (BNF) chapters 4.1.1 to 4.3.4 [8] on self-report medication lists at baseline. Duration of hypertension was calculated utilising age and age of hypertension diagnosis. Antihypertensive medication count was calculated as the absolute number of ACE inhibitors, angiotensin II receptor antagonists, calcium channel blockers, beta-blockers and thiazide diuretics prescribed to an individual. Generic medication names were sought and cross-referenced with the BNF chapters 2.2.1, 2.4, 2.5.5 and 2.6.2 [8].

Statistical analysis:

A best-fit multivariable regression spline model (stata command “mvrs”) was used to find the best model to adjust for non-linear covariates. For the adverse cardiovascular outcomes, A single knot was fitted for age at age 50 and two knots were fitted for total physical activity at 1.65 and 8.062 metabolic equivalent hours. In the male subgroup analysis two knots were fitted for total physical activity at 1.7 and 8.507 metabolic equivalent hours, in the female subgroup two knots were fitted for total physical activity at 1.57 and 3.75 and two knots

For peer review only - http://bmjopen.bmj.com/site/about/guidelines.xhtml

were fitted at systolic blood pressure 121.5 and 147.5. No bends were noted in any models for the stroke outcomes.

Model selection and covariate adjustment

All variables were tested against outcome measures (cardiovascular outcomes and stroke outcomes) using univariate analysis to assess appropriateness for inclusion in the final model. All covariates were significantly associated with the outcomes. and were Two continuous variables, age and total physical activity, expressed non-linearity within the main analysis and male subgroup analysis for cardiovascular outcomes and as such regression splines were used with two and three knots respectively. Two knots were included within the female subgroup analysis for physical activity. For stroke outcome there were no bends in the main or sex-specific models.

Within the main analysis for cardiovascular outcomes, the groups of depression only, Asian/Asian British ethnicity and BMI<18.5 covariates failed the proportionality assumption and as such, were incorporated into the model as a time varying coefficients. Within the sex specific models depression only failed the PH test within the female only analysis and ethnicity and BMI failed within the male only analysis. For the stroke outcomes gender and BMI class failed the proportionality assumption within the main analysis, with no failures within the sex-specific analysis. Analysis was repeated with the hypertension only as the comparator group to assess for any significant difference between the co-morbid group and the hypertension only group.

Time varying covariates

Due to the finding of MDD failing the proportionality assumption in the cardiovascular outcome in the primary analysis a series of further analyses have been performed to find when the assumption was not met. A log (-log) plot (fig 3) showed the proportionality assumption was broken at 22.5 months in the fully adjusted model in the primary analysis. As such, separate models were

For peer review only - http://bmjopen.bmj.com/site/about/guidelines.xhtml

performed prior to and after these points. Prior to 22.5months the HR for MDD shows a trend that is reduced but insignificant (HR 0.82, 95%CI 0.6 - 1.13), becoming significantly increased after the 22.5 time point. (HR 1.27, 95%CI 1.06 - 1.52) (Table 9 supplementary digital content). Both stratified models passed the proportionality assumption using Schoenfeld residuals. Similar to the major analysis, the female model showed the MDD only group failing the proportionality assumption, although this was at the 29 month time point. (tables 6 and 10 of the supplementary digital content).

For peer review only

For peer review only - http://bmjopen.bmj.com/site/about/guidelines.xhtml

References

1. Palmer LJ. UK Biobank: bank on it. *Lancet* 2007;369(9578):1980-2. doi: 10.1016/S0140-6736(07)60924-6
2. Sudlow C, Gallacher J, Allen N, et al. UK Biobank: An Open Access Resource for Identifying the Causes of a Wide Range of Complex Diseases of Middle and Old Age. *Plos Medicine* 2015;12(3) doi: 10.1371/journal.pmed.1001779
3. Guo W, Bradbury KE, Reeves GK, et al. Physical activity in relation to body size and composition in women in UK Biobank. *Annals of Epidemiology* 2015;25(6):406-13.e6. doi: http://dx.doi.org/10.1016/j.annepidem.2015.01.015
4. Craig CL, Marshall AL, Sjostrom M, et al. International physical activity questionnaire: 12-country reliability and validity. *Medicine and science in sports and exercise* 2003;35(8):1381-95. doi: 10.1249/01.mss.0000078924.61453.fb [published Online First: 2003/08/06]
5. Townsend P. Deprivation. *Journal of Social Policy* 2009;16(02):125. doi: 10.1017/s0047279400020341
6. Townsend P, Phillimore M, Beattie A. Health and Deprivation: Inequality and the North. London: Croom Helm Ltd 1988.
7. WHO. Obesity: preventing and managing the global epidemic. Report of a WHO consultation. *World Health Organ Tech Rep Ser* 2000;894:i-xii, 1-253.
8. COMMITTEE. JF. British National Formulary. 67 ed. London: BMJ Group and Pharmaceutical Press 2014.

For peer review only

For peer review only - http://bmjopen.bmj.com/site/about/guidelines.xhtml

For peer review only

For peer review only - http://bmjopen.bmj.com/site/about/guidelines.xhtml

Supplementary Tables and figures

Supplementary Table1: Descriptive analysis for adverse cardiovascular outcome – males only

	Comparator group		Hypertension only		MDD only		Hypertension plus MDD	
	N= 21570		N = 30142		N = 4169		N = 5253	
**Median age (range)***	54	(47 - 61)	61	(54 - 65)	53	(46 - 60)	59	(52 - 64)
**Ethnicity, N (%)**								
White	19562	(90.69%)	27808	(92.26%)	3923	(94.1%)	5001	(95.2%)
Asian/Asian British	863	(4.%)	969	(3.21%)	87	(2.09%)	86	(1.64%)
Black/ Black British	559	(2.59%)	780	(2.59%)	52	(1.25%)	54	(1.03%)
**Median Townsend score (range)***	-1.87	(-3.47 - 0.59)	-2.08	(-3.53 - 0.41)	-1.58	(-3.3 - 1.07)	-1.81	(-3.44 - 0.78)
**Age at leaving full-time education, N (%)**								
<16	2517	(11.67%)	6328	(20.99%)	464	(11.13%)	1005	(19.13%)
16	4473	(20.74%)	6235	(20.69%)	859	(20.6%)	1096	(20.86%)
>16	14344	(66.5%)	17257	(57.25%)	2807	(67.33%)	3118	(59.36%)
**Total physical activity in metabolic**	4.15	(1.75 - 8.51)	3.99	(1.65 - 8.51)	4.15	(1.7 - 8.36)	3.76	(1.54 - 7.97)

For peer review only - http://bmjopen.bmj.com/site/about/guidelines.xhtml

**Sedentary time in hours, median (range)***	4.5	(3.5 - 6)	5	(3.5 - 6.5)	5	(3.5 - 6.5)	5	(4 - 7)
**Diabetes, N (%)**	721	(3.34%)	2401	(7.97%)	159	(3.81%)	477	(9.08%)
**Hypercholesterolaemia, N (%)**	1614	(7.48%)	5585	(18.53%)	363	(8.71%)	1056	(20.1%)
**Systolic BP in mmHg, median (range)***	128	(121.5 - 133.5)	149.5	(142 - 159)	127.5	(120.5 - 133)	148	(141 - 157)
**Body Mass Index, N (%)**								
<18.5	74	(0.34%)	35	(0.12%)	22	(0.53%)	12	(0.23%)
18.5 – 25	7607	(35.27%)	5842	(19.38%)	1394	(33.44%)	890	(16.94%)
25-30	10594	(49.11%)	15114	(50.14%)	2019	(48.43%)	2532	(48.2%)
>30	3295	(15.28%)	9151	(30.36%)	734	(17.61%)	1819	(34.63%)
**Smoking status, N (%)**								
Never smoked	12038	(55.81%)	15145	(50.25%)	1999	(47.95%)	2268	(43.18%)
Previously smoked	6777	(31.42%)	12125	(40.23%)	1447	(34.71%)	2295	(43.69%)
Current smoker	2688	(12.46%)	2776	(9.21%)	716	(17.17%)	686	(13.06%)
**Alcohol frequency, N (%)**								
Daily or almost daily	4822	(22.36%)	8653	(28.71%)	969	(23.24%)	1503	(28.61%)
Three or four times a week	5718	(26.51%)	7913	(26.25%)	1022	(24.51%)	1323	(25.19%)

For peer review only - http://bmjopen.bmj.com/site/about/guidelines.xhtml

Once or twice a week	5932	(27.5%)	7546	(25.03%)	1063	(25.5%)	1178	(22.43%)
One to three times a month	2193	(10.17%)	2392	(7.94%)	440	(10.55%)	479	(9.12%)
Special occasions only	1554	(7.2%)	2154	(7.15%)	328	(7.87%)	423	(8.05%)
Never	1343	(6.23%)	1473	(4.89%)	345	(8.28%)	345	(6.57%)
**Psychotropic medication, N (%)**	398	(1.85%)	670	(2.22%)	678	(16.26%)	879	(16.73%)

*All data presented as N (%) and has chi-squared p-value of <0.001 except * which are median values (interquartile range) and have a Kruskal-Wallis p-value of 0.0001. Data presented as MET-hrs (hours spent doing exercise adjusted for multiples of basal metabolic rate in accordance with IPAQ).Townsend score is an area based measure based on census statistics. It is a calculation based on the number of: households without a car, overcrowded households, households not owner-occupied and unemployment.*

For peer review only - http://bmjopen.bmj.com/site/about/guidelines.xhtml

Supplementary Table 2: Descriptive analysis for adverse cardiovascular outcome – females only

	Comparator group		Hypertension only		MDD only		Hypertension plus MDD	
	N= 29228		N = 25893		N = 10929		N = 7676	
**Median age (range)***	54	(47 - 61)	61	(55 - 65)	53	(47 - 60)	60	(53 - 64)
**Ethnicity, N (%)**								
White	26585	(90.96%)	23441	(90.53%)	10324	(94.46%)	7271	(94.72%)
Asian/Asian British	908	(3.11%)	727	(2.81%)	174	(1.59%)	93	(1.21%)
Black/ Black British	764	(2.61%)	989	(3.82%)	167	(1.53%)	168	(2.19%)
**Median Townsend score (range)***	-1.90	(-3.44 - 0.51)	-2.06	(-3.5 - 0.38)	-1.66	(-3.3 - 0.84)	-1.87	(-3.4 - 0.74)
**Age at leaving full-time education, N (%)**								
<16	3399	(11.63%)	5757	(22.23%)	1261	(11.54%)	1602	(20.87%)
16	5792	(19.82%)	5592	(21.6%)	2319	(21.22%)	1636	(21.31%)
>16	19746	(67.56%)	14223	(54.93%)	7283	(66.64%)	4385	(57.13%)
**Total physical activity in metabolic**	3.87	(1.65 - 7.71)	3.51	(1.37 - 7.59)	3.79	(1.65 - 7.91)	3.65	(1.45 - 7.93)
**Sedentary time in hours, median (range)***	4	(3 - 5)	4	(3 - 5.5)	4	(3 - 5.5)	4.5	(3 - 6)

For peer review only - http://bmjopen.bmj.com/site/about/guidelines.xhtml

**Diabetes, N (%)**	547	(1.87%)	1376	(5.31%)	221	(2.02%)	452	(5.89%)
**Hypercholesterolaemia, N (%)**	1397	(4.78%)	3625	(14.%)	530	(4.85%)	1155	(15.05%)
**Systolic BP in mmHg, median (range)***	123.5	(115.5 - 130.5)	149.5	(142 - 160)	122.5	(114.5 - 130)	147.5	(140.5 - 157)
**Body Mass Index, N (%)**								
<18.5	315	(1.08%)	107	(0.41%)	81	(0.74%)	22	(0.29%)
18.5 – 25	14942	(51.12%)	7836	(30.26%)	4857	(44.44%)	1984	(25.85%)
25-30	9816	(33.58%)	10102	(39.01%)	3917	(35.84%)	2857	(37.22%)
>30	4155	(14.22%)	7848	(30.31%)	2074	(18.98%)	2813	(36.65%)
**Smoking status, N (%)**								
Never smoked	18588	(63.6%)	16358	(63.18%)	5865	(53.66%)	4186	(54.53%)
Previously smoked	8279	(28.33%)	8015	(30.95%)	3671	(33.59%)	2770	(36.09%)
Current smoker	2282	(7.81%)	1423	(5.5%)	1377	(12.6%)	695	(9.05%)
**Alcohol frequency, N (%)**								
Daily or almost daily	4628	(15.83%)	4317	(16.67%)	1767	(16.17%)	1378	(17.95%)
Three or four times a week	6457	(22.09%)	5120	(19.77%)	2231	(20.41%)	1514	(19.72%)
Once or twice a week	7712	(26.39%)	6343	(24.5%)	2817	(25.78%)	1738	(22.64%)

For peer review only - http://bmjopen.bmj.com/site/about/guidelines.xhtml

One to three times a month	3859	(13.2%)	3196	(12.34%)	1618	(14.8%)	1033	(13.46%)
Special occasions only	3980	(13.62%)	4176	(16.13%)	1576	(14.42%)	1306	(17.01%)
Never	2581	(8.83%)	2726	(10.53%)	917	(8.39%)	703	(9.16%)
**Psychotropic medication, N (%)**	943	(3.23%)	1125	(4.34%)	2166	(19.82%)	1643	(21.4%)

*All data presented as N (%) and has chi-squared p-value of <0.001 except * which are median values (interquartile range) and have a Kruskal-Wallis p-value of 0.0001. Data presented as MET-hrs (hours spent doing exercise adjusted for multiples of basal metabolic rate in accordance with IPAQ).Townsend score is an area based measure based on census statistics. It is a calculation based on the number of: households without a car, overcrowded households, households not owner-occupied and unemployment.*

For peer review only

Supplementary Table 3: Descriptive analysis for stroke outcome – males only

	Comparator group		Hypertension only		MDD only		Hypertension plus MDD	
	N = 22816		N = 32787		N = 4438		N = 5857	
**Median age (range)***	55	(47 - 62.)	61	(54 - 65)	54	(47 - 61)	60	(53 - 64)
**Ethnicity, N (%)**								
White	20699	(90.72%)	30219	(92.17%)	4173	(94.03%)	5569	(95.08%)
Asian/Asian British	932	(4.08%)	1116	(3.4%)	102	(2.3%)	105	(1.79%)
Black/ Black British	576	(2.52%)	820	(2.5%)	53	(1.19%)	59	(1.01%)
**Median Townsend score (range)***	-1.88	(-3.47 - 0.59)	-2.05	(-3.5 - 0.46)	-1.56	(-3.28 - 1.15)	-1.74	(-3.4 - 0.93)
**Age at leaving full-time education, N (%)**								
<16	2900	(12.71%)	7256	(22.13%)	558	(12.57%)	1193	(20.37%)
16	4702	(20.61%)	6704	(20.45%)	909	(20.48%)	1222	(20.86%)
>16	14960	(65.57%)	18471	(56.34%)	2930	(66.02%)	3397	(58.%)
**Total physical activity in metabolic**	4.12	(1.74 - 8.48)	3.96	(1.65 - 8.44)	4.13	(1.67 - 8.36)	3.66	(1.45 - 7.83)
**Sedentary time in hours, median (range)***	5	(3.5 - 6)	5	(4 - 7)	5	(3.5 - 6.5)	5	(4 - 7)

15

**Diabetes, N (%)**	873	(3.83%)	2951	(9.%)	208	(4.69%)	635	(10.84%)
**Hypercholesterolaemia, N (%)**	2045	(8.96%)	6736	(20.54%)	457	(10.3%)	1293	(22.08%)
**Systolic BP in mmHg, median (range)***	128	(121.5 - 133.5)	149	(142 - 159)	127	(120.5 - 133)	147.5	(140.5 - 156.5)
**Body Mass Index, N (%)**								
<18.5	79	(0.35%)	39	(0.12%)	22	(0.5%)	12	(0.2%)
18.5 – 25	7867	(34.48%)	6215	(18.96%)	1452	(32.72%)	960	(16.39%)
25-30	11203	(49.1%)	16341	(49.84%)	2142	(48.26%)	2780	(47.46%)
>30	3667	(16.07%)	10192	(31.09%)	822	(18.52%)	2105	(35.94%)
**Smoking status, N (%)**								
Never smoked	12502	(54.79%)	16054	(48.96%)	2094	(47.18%)	2469	(42.15%)
Previously smoked	7399	(32.43%)	13603	(41.49%)	1582	(35.65%)	2610	(44.56%)
Current smoker	2836	(12.43%)	3013	(9.19%)	754	(16.99%)	770	(13.15%)
**Alcohol frequency, N (%)**								
Daily or almost daily	5085	(22.29%)	9309	(28.39%)	1021	(23.01%)	1645	(28.09%)
Three or four times a week	6039	(26.47%)	8556	(26.1%)	1077	(24.27%)	1450	(24.76%)
Once or twice a week	6264	(27.45%)	8161	(24.89%)	1121	(25.26%)	1305	(22.28%)

For peer review only - http://bmjopen.bmj.com/site/about/guidelines.xhtml

One to three times a month	2307	(10.11%)	2642	(8.06%)	478	(10.77%)	538	(9.19%)
Special occasions only	1666	(7.3%)	2394	(7.3%)	355	(8.%)	503	(8.59%)
Never	1444	(6.33%)	1711	(5.22%)	383	(8.63%)	414	(7.07%)
**Psychotropic medication, N (%)**	429	(1.88%)	793	(2.42%)	735	(16.56%)	1025	(17.5%)

*All data presented as N (%) and has chi-squared p-value of <0.001 except * which are median values (interquartile range) and have a Kruskal-Wallis p-value of 0.0001. Data presented as MET-hrs (hours spent doing exercise adjusted for multiples of basal metabolic rate in accordance with IPAQ).Townsend score is an area based measure based on census statistics. It is a calculation based on the number of: households without a car, overcrowded households, households not owner-occupied and unemployment.*

For peer review only - http://bmjopen.bmj.com/site/about/guidelines.xhtml

Supplementary Table 4: Descriptive analysis for stroke outcome – females only

	Comparator group		Hypertension only		MDD only		Hypertension plus MDD	
	N = 29684		N = 26937		N = 11143		N = 8090	
**Median age (range)***	54	(47 - 61)	61	(56 - 65)	53	(47 - 60)	60	(54 - 64)
**Ethnicity, N (%)**								
White	26998	(90.95%)	24359	(90.43%)	10524	(94.44%)	7643	(94.47%)
Asian/Asian British	925	(3.12%)	773	(2.87%)	178	(1.6%)	104	(1.29%)
Black/ Black British	779	(2.62%)	1034	(3.84%)	170.00	(1.53%)	187	(2.31%)
**Median Townsend score (range)***	-1.90	(-3.44 - 0.52)	-2.03	(-3.48 - 0.43)	-1.66	(-3.29 - 0.86)	-1.83	(-3.38 - 0.85)
**Age at leaving full-time education, N (%)**								
<16	3546	(11.95%)	6140	(22.79%)	1326	(11.9%)	1752	(21.66%)
16	5888	(19.84%)	5803	(21.54%)	2361	(21.19%)	1731	(21.4%)
>16	19954	(67.22%)	14643	(54.36%)	7387	(66.29%)	4550	(56.24%)
**Total physical activity in metabolic**	3.85	(1.65 - 7.7)	3.49	(1.35 - 7.57)	3.79	(1.65 - 7.89)	3.61	(1.41 - 7.87)

For peer review only - http://bmjopen.bmj.com/site/about/guidelines.xhtml

**Sedentary time in hours, median (range)***	4.0	(3 - 5)	4.0	(3 - 5.5)	4.0	(3 - 5.5)	4.5	(3 - 6)
**Diabetes, N (%)**	581	(1.96%)	1551	(5.76%)	241	(2.16%)	528	(6.53%)
**Hypercholesterolaemia, N (%)**	1547	(5.21%)	4032	(14.97%)	592	(5.31%)	1327	(16.4%)
**Systolic BP in mmHg, median (range)***	123.5	(115.5 - 131)	149.5	(142 - 160)	122.5	(114.5 - 130)	147.0	(140.5 - 157)
**Body Mass Index, N (%)**								
<18.5	316	(1.06%)	112	(0.42%)	82	(0.74%)	26	(0.32%)
18.5 – 25	15100	(50.87%)	8027	(29.8%)	4922	(44.17%)	2057	(25.43%)
25-30	9982	(33.63%)	10476	(38.89%)	4007	(35.96%)	2989	(36.95%)
>30	4286	(14.44%)	8322	(30.89%)	2132	(19.13%)	3018	(37.31%)
**Smoking status, N (%)**								
Never smoked	18816	(63.39%)	16928	(62.84%)	5958	(53.47%)	4365	(53.96%)
Previously smoked	8452	(28.47%)	8416	(31.24%)	3758	(33.73%)	2950	(36.46%)
Current smoker	2334	(7.86%)	1488	(5.52%)	1409	(12.64%)	749	(9.26%)
**Alcohol frequency, N (%)**								

For peer review only - http://bmjopen.bmj.com/site/about/guidelines.xhtml

Daily or almost daily	4675	(15.75%)	4442	(16.49%)	1796	(16.12%)	1440	(17.8%)
Three or four times a week	6524	(21.98%)	5271	(19.57%)	2258	(20.26%)	1570	(19.41%)
Once or twice a week	7825	(26.36%)	6558	(24.35%)	2872	(25.77%)	1820	(22.5%)
One to three times a month	3913	(13.18%)	3329	(12.36%)	1644	(14.75%)	1089	(13.46%)
Special occasions only	4078	(13.74%)	4400	(16.33%)	1623	(14.57%)	1382	(17.08%)
Never	2658	(8.95%)	2919	(10.84%)	947	(8.5%)	785	(9.7%)
**Psychotropic medication, N (%)**	979	(3.3%)	1203	(4.47%)	2241	(20.11%)	1753	(21.67%)

*All data presented as N (%) and has chi-squared p-value of <0.001 except * which are median values (interquartile range) and have a Kruskal-Wallis p-value of 0.0001. Data presented as MET-hrs (hours spent doing exercise adjusted for multiples of basal metabolic rate in accordance with IPAQ).Townsend score is an area based measure based on census statistics. It is a calculation based on the number of: households without a car, overcrowded households, households not owner-occupied and unemployment.*

For peer review only - http://bmjopen.bmj.com/site/about/guidelines.xhtml

Supplementary Table 5: Risk of adverse cardiovascular event by clinical group, in males only.

	Model one (unadjusted)			Model two (partially adjusted)*			Model three (fully adjusted) †		
**Group**	HR	95% C.I.	p-value	aHR	95% C.I.	p-value	aHR	95% C.I.	p-value
No Hypertension- No MDD	1(ref)			1(ref)			1(ref)		
Hypertension only	2.21	(2.00-2.45)	2.28×10^{-53}	1.62	(1.46-1.83)	5.80×10^{-19}	1.29	(1.13-1.47)	1.35×10^{-4}
MDD only	1.17	(0.95-1.56)	0.12	1.18	(0.95-1.46)	0.12	1.12	(0.9-1.39)	0.3
Hypertension and MDD	2.46	(2.13-2.84)	3.12×10^{-34}	1.95	(1.68-2.27)	2.81×10^{-18}	1.47	(1.24-1.74)	8.71×10^{-6}

**Adjusted for sociodemographic factors (age, Townsend score, age of leaving full time education and ethnicity. †Additionally adjusted for history of diabetes, history of hypercholesterolemia, BMI, smoking history, alcohol use, systolic blood pressure, sedentary hours per day, physical activity and psychotropic medication use. MDD = Major depressive disorder, HR = Hazard ratio, aHR = Adjusted hazard ratio, C.I.= Confidence interval*

For peer review only - http://bmjopen.bmj.com/site/about/guidelines.xhtml

Supplementary Table 6: Risk of adverse cardiovascular event by clinical group, in females only.

	Model one (unadjusted)			Model two (partially adjusted)*			Model three (fully adjusted) †		
**Group**	HR	95% C.I.	p-value	aHR	95% C.I.	p-value	aHR	95% C.I.	p-value
No Hypertension - No MDD	1(ref)			1(ref)			1(ref)		
Hypertension only	2.75	(2.38 - 3.18)	$6.16x10^{-43}$	1.86	(1.6-2.17)	$1.43x10^{-15}$	1.64	(1.33-2.02)	$4.36x10^{-6}$
MDD only	0.67	(0.42-1.08)	0.10	0.72	(0.45-1.17)	0.19	0.68	(0.42-1.1)	0.12
Hypertension and MDD	3.68	(3.1-4.38)	$5.62x10^{-49}$	2.78	(1.58-3.29)	$4.62x10^{-29}$	2.18	(1.82-2.92)	$4.76x10^{-11}$
**Time varying Variables**									
MDD only	1.02	(1.006-1.03)	$2.45x10^{-3}$	1.02	(1.005-1.03)	$4.00x10^{-3}$	1.02	(1.004-1.03)	$6.19x10^{-3}$

**Adjusted for sociodemographic factors (age, Townsend score, age of leaving full time education and ethnicity. †Additionally adjusted for history of diabetes, history of hypercholesterolemia, BMI, smoking history, alcohol use, systolic blood pressure, sedentary hours per day, physical activity and psychotropic medication use. MDD = Major depressive disorder, HR = Hazard ratio, aHR = Adjusted hazard ratio, C.I.= Confidence interval*

For peer review only - http://bmjopen.bmj.com/site/about/guidelines.xhtml

Supplementary Table 7: Risk of stroke event by clinical group, in males only.

	Model one (unadjusted)			Model two (partially adjusted)*			Model three (fully adjusted) †		
**Group**	HR	95% C.I.	p-value	aHR	95% C.I.	p-value	aHR	95% C.I.	p-value
No Hypertension - No MDD	1(ref)			1(ref)			1(ref)		
Hypertension only	2.43	(1.95 - 3.03)	1.92×10^{-15}	1.74	(1.38 - 2.19)	2.58×10^{-6}	1.19	(0.9 - 1.58)	0.22
MDD only	1.45	(0.96 - 2.2)	0.07	1.65	(1.09 - 2.5)	0.02	1.49	(0.97 - 2.29)	0.07
Hypertension and MDD	2.39	(1.74 - 3.27)	7.34×10^{-8}	1.87	(1.35 - 2.6)	1.55×10^{-4}	1.20	(0.83 - 1.74)	0.33

**Adjusted for sociodemographic factors (age, sex, Townsend score, age of leaving full time education and ethnicity. †Additionally adjusted for history of diabetes, history of hypercholesterolemia, BMI, smoking history, alcohol use, systolic blood pressure, sedentary hours per day, physical activity and psychotropic medication use. MDD = Major depressive disorder, HR = Hazard ratio, aHR = Adjusted hazard ratio, C.I.= Confidence interval*

For peer review only - http://bmjopen.bmj.com/site/about/guidelines.xhtml

Supplementary Table 8: Risk of stroke event by clinical group, in females only.

	Model one (unadjusted)			Model two (partially adjusted)*			Model three (fully adjusted) †		
**Group**	HR	95% C.I.	p-value	aHR	95% C.I.	p-value	aHR	95% C.I.	p-value
No Hypertension - No MDD	1(ref)			1(ref)			1(ref)		
Hypertension only	2.38	(1.84 - 3.09)	$6.50x10^{-11}$	1.51	(1.14 - 1.99)	$3.63x10^{-3}$	1.25	(0.88 - 1.79)	0.21
MDD only	1.09	(0.73 - 1.62)	0.67	1.15	(0.76 - 1.75)	0.51	0.99	(0.64 - 1.53)	0.98
Hypertension and MDD	3.05	(2.22 - 4.21)	$8.71x10^{-12}$	2.22	(1.59 - 3.08)	$2.27x10^{-6}$	1.62	(1.08 - 2.42)	0.02

**Adjusted for sociodemographic factors (age, sex, Townsend score, age of leaving full time education and ethnicity. †Additionally adjusted for history of diabetes, history of hypercholesterolemia, BMI, smoking history, alcohol use, systolic blood pressure, sedentary hours per day, physical activity and psychotropic medication use. MDD = Major depressive disorder, HR = Hazard ratio, aHR = Adjusted hazard ratio, C.I.= Confidence interval*

For peer review only - http://bmjopen.bmj.com/site/about/guidelines.xhtml

Supplementary Table 9: Time stratified analysis by moment of proportional hazards failure for adverse cardiovascular outcomes (stratified at 22.5 months)

	Fully adjusted* model pre-22.5 months			Fully adjusted* model post-22.5 months		
**Group**	aHR	95% C.I.	p-value	aHR	95% C.I.	p-value
No Hypertension - No MDD	1(ref)			1(ref)		
Hypertension only	1.36	(1.12 - 1.66)	0.002	1.36	(1.19 -1.55)	$5.06x10^{-6}$
MDD only	0.82	(0.60 - 1.13)	0.22	1.27	(1.06 -1.52)	0.01
Hypertension and MDD	1.75	(1.39 - 2.21)	$2.62x10^{-6}$	1.62	(1.38 - 1.90)	$5.72x10^{-9}$

**Adjusted for sociodemographic factors (age, sex, Townsend score, age of leaving full time education and ethnicity, history of diabetes, history of hypercholesterolemia, BMI, smoking history, alcohol use, systolic blood pressure, sedentary hours per day, physical activity and psychotropic medication use.*

MDD = Major depressive disorder, aHR =adjusted hazard ratio, C.I.= Confidence interval

For peer review only - http://bmjopen.bmj.com/site/about/guidelines.xhtml

Supplementary Table 10: Time stratified analysis by moment of proportional hazards failure for adverse cardiovascular outcomes (females only - stratified at 29 months)

	Fully adjusted* model pre-29 months			Fully adjusted* model post-29 months		
**Group**	HR	95% C.I.	p-value	HR	95% C.I.	p-value
No Hypertension - No MDD	1(ref)			1(ref)		
Hypertension only	1.49	(1.06 - 2.08)	0.02	1.75	(1.33 - 2.30)	5.56×10^{-5}
MDD only	0.73	(0.48 - 1.10)	0.13	1.58	(1.19 - 2.09)	0.002
Hypertension and MDD	1.80	(1.24 - 2.62)	0.002	2.47	(1.83 - 3.33)	2.89×10^{-9}

**Adjusted for sociodemographic factors (age, sex, Townsend score, age of leaving full time education and ethnicity, history of diabetes, history of hypercholesterolemia, BMI, smoking history, alcohol use, systolic blood pressure, sedentary hours per day, physical activity and psychotropic medication use.*

MDD = Major depressive disorder, aHR =adjusted hazard ratio, C.I.= Confidence interval

For peer review only - http://bmjopen.bmj.com/site/about/guidelines.xhtml

Supplementary Table 11: Relative excess risk due to interaction results on fully adjusted* models

Analysis	RERI	95% C.I.
Adverse cardiovascular outcome before 22.5 months	**0.563**	**(0.189 - 0.938)**
Adverse cardiovascular outcome after 22.5 months	-0.009	(-0.293 - 0.275)
Adverse cardiovascular outcome (males only)	0.058	(-0.240 - 0.357)
Adverse cardiovascular outcome (females only)before 29 months	**0.588**	**(0.074 - 1.103)**
Adverse cardiovascular outcome (females only)after 29 months	0.142	(-0.447 - 0.732)
Stroke outcome	-0.047	(-0.485 - 0.391)
Stroke outcome (males only)	-0.480	(-1.195 - 0.234)
Stroke outcome (females only)	0.372	(-0.216 - 0.959)

**Adjusted for sociodemographic factors (age, sex, Townsend score, age of leaving full time education and ethnicity, history of diabetes, history of hypercholesterolemia, BMI, smoking history, alcohol use, systolic blood pressure, sedentary hours per day, physical activity and psychotropic medication use.*

RERI = Relative excess risk due to interaction, C.I.= Confidence interval

For peer review only - http://bmjopen.bmj.com/site/about/guidelines.xhtml

Supplementary Table 12: Comparison of additional hypertension factors (medication and diagnosis duration) across groups

	No Hypertension – No MDD		Hypertension only		MDD only		Hypertension and MDD	
**Antihypertensive medication prescription, N (%)**	1,265	(2.49)	19,045	(33.99)	476	(3.04)	5,037	(37.34)
**Number of antihypertensive medications, N (range)***	1	(1-1)	1	(1-2)	1	(1-1)	1	(1- 2)
**Reported a duration of hypertension, N (%)**	*1,376*	(2.71)	*16,709*	(29.82)	678	(4.32)	4,525	(33.55)
**Duration of hypertension in years, median (range)***	6	(2-14)	8	(4-13)	6	(3-14)	8	(4 - 14)

*****_Median quantity of antihpertensive medications and median duration of hypertensive diagnosis presented for those on antihypertensive medications and supplied an age of hypertension diagnosis, respectively. MDD = Major Depressive disorder_

For peer review only - http://bmjopen.bmj.com/site/about/guidelines.xhtml

Reporting checklist for cohort study.

Based on the STROBE cohort guidelines.

Instructions to authors

Complete this checklist by entering the page numbers from your manuscript where readers will find each of the items listed below.

Your article may not currently address all the items on the checklist. Please modify your text to include the missing information. If you are certain that an item does not apply, please write "n/a" and provide a short explanation.

Upload your completed checklist as an extra file when you submit to a journal.

In your methods section, say that you used the STROBE cohort reporting guidelines, and cite them as:

von Elm E, Altman DG, Egger M, Pocock SJ, Gotzsche PC, Vandenbroucke JP. The Strengthening the Reporting of Observational Studies in Epidemiology (STROBE) Statement: guidelines for reporting observational studies.

		Reporting Item	Page Number
Title	#1a	Indicate the study’s design with a commonly used term in the title or the abstract	1
Abstract	#1b	Provide in the abstract an informative and balanced summary of what was done and what was found	3
Background / rationale	#2	Explain the scientific background and rationale for the investigation being reported	5
Objectives	#3	State specific objectives, including any prespecified hypotheses	5
Study design	#4	Present key elements of study design early in the paper	5
Setting	#5	Describe the setting, locations, and relevant dates, including periods of recruitment, exposure, follow-up, and data collection	6
Eligibility criteria	#6a	Give the eligibility criteria, and the sources and methods of selection of participants. Describe methods of follow-up.	6-7

For peer review only

	#6b	For matched studies, give matching criteria and number of exposed and unexposed	n/a
Variables	#7	Clearly define all outcomes, exposures, predictors, potential confounders, and effect modifiers. Give diagnostic criteria, if applicable	7-8
Data sources / measurement	#8	For each variable of interest give sources of data and details of methods of assessment (measurement). Describe comparability of assessment methods if there is more than one group. Give information separately for for exposed and unexposed groups if applicable.	6-8
Bias	#9	Describe any efforts to address potential sources of bias	6
Study size	#10	Explain how the study size was arrived at	6
Quantitative variables	#11	Explain how quantitative variables were handled in the analyses. If applicable, describe which groupings were chosen, and why	See note 1
Statistical methods	#12a	Describe all statistical methods, including those used to control for confounding	8-9
	#12b	Describe any methods used to examine subgroups and interactions	See note 2
	#12c	Explain how missing data were addressed	6-7
	#12d	If applicable, explain how loss to follow-up was addressed	1
	#12e	Describe any sensitivity analyses	9
Participants	#13a	Report numbers of individuals at each stage of study—eg numbers potentially eligible, examined for eligibility, confirmed eligible, included in the study, completing follow-up, and analysed. Give information separately for for exposed and unexposed groups if applicable.	10
	#13b	Give reasons for non-participation at each stage	6,7
	#13c	Consider use of a flow diagram	n/a
Descriptive data	#14a	Give characteristics of study participants (eg demographic, clinical, social) and information on exposures and potential	10

For peer review only

		confounders. Give information separately for exposed and unexposed groups if applicable.	
	#14b	Indicate number of participants with missing data for each variable of interest	See note 3
	#14c	Summarise follow-up time (eg, average and total amount)	10
Outcome data	#15	Report numbers of outcome events or summary measures over time. Give information separately for exposed and unexposed groups if applicable.	6
Main results	#16a	Give unadjusted estimates and, if applicable, confounder-adjusted estimates and their precision (eg, 95% confidence interval). Make clear which confounders were adjusted for and why they were included	11-12
	#16b	Report category boundaries when continuous variables were categorized	n/a
	#16c	If relevant, consider translating estimates of relative risk into absolute risk for a meaningful time period	n/a
Other analyses	#17	Report other analyses done—e.g., analyses of subgroups and interactions, and sensitivity analyses	See note 4
Key results	#18	Summarise key results with reference to study objectives	12
Limitations	#19	Discuss limitations of the study, taking into account sources of potential bias or imprecision. Discuss both direction and magnitude of any potential bias.	14
Interpretation	#20	Give a cautious overall interpretation considering objectives, limitations, multiplicity of analyses, results from similar studies, and other relevant evidence.	13-17
Generalisability	#21	Discuss the generalisability (external validity) of the study results	15
Funding	#22	Give the source of funding and the role of the funders for the present study and, if applicable, for the original study on which the present article is based	18

Author notes

1. 6,7,8,9, supplementary

For peer review only

2. 8-9, supplementary

3. n/a (supplementary)

4. 11-12, supplemental

The STROBE checklist is distributed under the terms of the Creative Commons Attribution License CC-BY. This checklist was completed on 25. May 2018 using http://www.goodreports.org/, a tool made by the EQUATOR Network in collaboration with Penelope.ai

For peer review only

BMJ Open

Impact of major depression on cardiovascular outcomes for individuals with hypertension: prospective survival analysis in UK Biobank.

Journal:	*BMJ Open*
Manuscript ID	bmjopen-2018-024433.R2
Article Type:	Original research
Date Submitted by the Author:	20-Mar-2019
Complete List of Authors:	Graham, Nicholas; University of Glasgow Institute of Health and Wellbeing, Gartnavel Royal Hopsital 1055 Great Western Road Glasgow, UK G12 0XH<br>Ward, Joey; University of Glasgow Institute of Health and Wellbeing<br>Mackay, Daniel; University of Glasgow Institute of Health and Wellbeing<br>Pell, J. P.; University of Glasgow Institute of Health and Wellbeing<br>Cavanagh, Jonathan; University of Glasgow Institute of Health and Wellbeing<br>Padmanabhan, Sandosh; University of Glasgow, Institute of Cardiovascular and Medical Sciences, British Heart Foundation Glasgow Cardiovascular Research Centre<br>Smith, Daniel; University of Glasgow, Institute of Health and Wellbeing
**Primary Subject Heading**:	Epidemiology
Secondary Subject Heading:	Mental health, Cardiovascular medicine
Keywords:	EPIDEMIOLOGY, mortality, cardiovascular disease, morbidity, depression, Hypertension < CARDIOLOGY

SCHOLARONE™
Manuscripts

Impact of major depression on cardiovascular outcomes for individuals with hypertension: prospective survival analysis in UK Biobank.

Short title: Outcomes of Hypertension plus Depression

Nicholas A GRAHAM*[a], Clinical Research Fellow

Joey WARD[a], Research Fellow

Daniel MACKAY[b], Reader in Public Health

Jill PELL[b], Professor of Public Health

Jonathan CAVANAGH[c], Professor of Psychiatry

Sandosh PADMANABHAN[d], Professor of Cardiovascular Genomics and Therapeutics

Daniel J. SMITH[a], Professor of Psychiatry.

Number of Supplementary files: 1

Word count of Manuscript: 3,969 (exc. Tables, references, abstract, summary and Author contribution statements)

Word count of Supplementary file: 1,455 (exc. tables)

Number of tables and figures: 18 tables (including 12 in supplementary digital content) and 3 figures

[a]Institute of Health and Wellbeing, University of Glasgow, Gartnavel Royal Hospital, 1055 Great Western Road, Glasgow G12 OXH. [b]Institute of Health and Wellbeing, University of Glasgow, Public Health, 1 Lilybank Gardens, Glasgow G12 8RZ. [c]Institute of Health and Wellbeing, Centre for Immunobiology, Sir Graeme Davies Building College of Medical, Veterinary and Life Sciences University of Glasgow. [d]Institute of Cardiovascular and Medical Sciences, British Heart Foundation Glasgow Cardiovascular Research centre, University of Glasgow, Glasgow G12 8TA.

For peer review only

For peer review only - http://bmjopen.bmj.com/site/about/guidelines.xhtml

*corresponding author: nicholas.graham@glasgow.ac.uk, phone: +44 0141 211 3918,

CONFLICTS OF INTEREST: None.

For peer review only

For peer review only - http://bmjopen.bmj.com/site/about/guidelines.xhtml

ABSTRACT

Objectives: To assess whether a history of major depressive disorder (MDD) in middle-aged individuals with hypertension influences first-onset cardiovascular disease outcomes.

Design: Prospective cohort survival analysis using Cox proportional hazards regression with a median follow-up of 63 months (702,902 person-years). Four mutually exclusive groups were compared: hypertension only (n=56,035), MDD only (n=15,098), comorbid hypertension plus MDD (n=12,929), and an unaffected (no hypertension, no MDD) comparison group (n=50,798).

Setting: UK Biobank

Participants: UK Biobank participants without cardiovascular disease aged 39–70 who completed psychiatric questions relating ICD-10 diagnostic criteria on a touchscreen questionnaire at baseline interview in 2006–2010 (n=134,860).

Primary and Secondary outcome measures: First-onset adverse cardiovascular outcomes leading to hospital admission or death (ICD-10 codes I20-I259, I60-69 and G45- G46), adjusted in a stepwise manner for sociodemographic, health and lifestyle features. Secondary analyses were performed looking specifically at stroke outcomes (ICD-10 codes I60-69 and G45- G46) and in gender-separated models.

Results: Relative to controls, adjusted hazard ratios (HRs) for adverse cardiovascular outcomes were increased for the hypertension only group (HR=1.36, 95%CI 1.22-1.52) and were higher still for the comorbid hypertension plus MDD group (HR=1.66, 95%CI 1.45-1.9). HRs for the comorbid hypertension plus MDD group were significantly raised compared to hypertension alone (HR=1.22, 95%CI 1.1-1.35). Interaction measured using relative excess risk due to interaction (RERI) and likelihood ratios (LR) were identified at baseline (RERI=0.563, 95%CI 0.189 - 0.938; LR chi 6.38, p= 0.0116) but not maintained during follow-up.

Limitations: Possible selection bias in UK Biobank and inability to assess for levels of medication adherence.

For peer review only - http://bmjopen.bmj.com/site/about/guidelines.xhtml

Conclusions: Comorbid hypertension and MDD conferred greater hazard than hypertension alone for adverse cardiovascular outcomes, although evidence of interaction between hypertension and MDD was inconsistent over time. Future cardiovascular risk prediction tools may benefit from the inclusion of questions about prior history of depressive disorders.

Word count of Abstract: 299

Key words: epidemiology, mortality, morbidity, depression; hypertension, cardiovascular disease

Article Summary

STRENGTHS AND LIMITATIONS

- Methodological advantages over previous studies, including a very large sample size, adjustment for a more comprehensive range of confounders, and the inclusion of non-fatal adverse cardiovascular events from hospital admission data and death registry data.
- Definition of prior MDD history was based on ICD-10 diagnostic criteria (rather than a score on a symptoms questionnaire) and our composite definition of hypertension incorporated past history, current medication and objective blood pressure measurements.
- Although analyses were adjusted for a broad range of baseline factors (such as smoking status, BMI, psychotropic medication use and diabetes), we were unable to account for how these factors may have changed over the course of follow-up, or assess adherence to cardiovascular medications.
- Trained nurses interviewed UK Biobank participants, but the self-report nature of some of these data may represent a limitation.
- UK Biobank may have issues with respect to selection biases. For example, individuals with more severe MDD may have been less likely to volunteer.

For peer review only

INTRODUCTION

By 2030 major depressive disorder (MDD) and cardiovascular disease (CVD) will be the two leading causes of disability worldwide[1]. MDD is associated with CVD and worse long-term outcomes[2]. To date, survival analysis in comorbid hypertension and MDD have focussed on all-cause death[3-5] cardiovascular death[5] or incorporated individuals with previous CVD[3-6], and have suggested a possible additive interaction between hypertension and MDD on survival[5 6]. MDD is well known to worsen post-cardiovascular event survival[6 7]. The contribution on survival to first-onset CVD is less clear when MDD is stratified by hypertension and no prior study has assessed comorbid MDD and hypertension on first episode CVD. Within this study we look specifically at first-onset events, irrespective of whether they lead to death or not.

Hypertension is extremely common (affecting 1 billion people worldwide)[8] and is responsible for 50% of all CVD[9]. It is commonly comorbid with MDD[10 11], with recent meta-analysis showing 27% of individuals with hypertension having MDD[12] and population-based studies showing a hypertension prevalence of 21% in those with MDD[11]. A biological link has been found by genome-wide association studies, showing calcium-channel genes, important in blood pressure (BP) control and hypertension[13], also act to increase risk for MDD[14 15] and bipolar disorder (BD)[16 17]. The sympathetic nervous system (SNS), Renin-angiotensin system, the immune system and the cortisol stress response system are all also implicated in both conditions[18]. Medication management of both conditions are also thought to impact one another with side effects of psychotropic medications including raised BP and vice versa[19-21], although there is contrary evidence suggesting either medication or MDD may in actual fact be protective of hypertension[20 22].

Here we make use of prospective data from the UK Biobank cohort[23] to test the hypothesis that a lifetime history of MDD in individuals with hypertension impacts adversely on first-episode cardiovascular events. We also assess whether MDD exacerbates the effects of hypertension as a risk factor for CVD.

For peer review only - http://bmjopen.bmj.com/site/about/guidelines.xhtml

METHODS

Study design

This was a population cohort study using data from UK Biobank. Four mutually exclusive groups (hypertension only, MDD only, hypertension plus MDD, and a comparison group) were compared for adverse CVD and stroke outcomes.

Sample description

UK Biobank is a large cohort of 502,655 participants recruited between April 2007 and July 2010 from 21 assessment centres located across Great Britain[23]. Participants aged 39-70 were invited to take participate if registered with the NHS and lived within a reasonable distance of an assessment centre. At baseline assessment participants completed a series of detailed assessments relating to lifestyle and medical history on touchscreen questionnaire and have a range of physical health measurements, including body mass index (BMI) and BP taken by a nurse. UK Biobank was approved by the North West NHS Multi-Centre Research Ethics Committee and all participants provided written informed consent to participate. This analysis is part of UK Biobank approved application number 7155.

During the last two years of recruitment, questions relating to mood disorder features were added to the baseline assessment schedule questionnaire. From the 172,729 participants asked these questions, 134,860 provided sufficient responses to be included in our analysis. Participants were excluded based on the following *a priori* criteria: a history of BD (n=1,831) or schizophrenia (n=262); where there were insufficient data provided by participants to clearly rule out MDD (n= 25,520) or hypertension (n=1,080); and where there were coding errors for date and/or time of death (n=4). These exclusions were based on self-report (individuals who listed schizophrenia or BD from a list of pre-existing medical conditions), or criteria for BD as per Smith et al,[24] or where they responded “don’t know” or “prefer not to answer” to questions or data was missing that would limit our ability

For peer review only - http://bmjopen.bmj.com/site/about/guidelines.xhtml

to exclude the presence of hypertension or MDD. Participants were further excluded from the adverse CVD outcome if they had a record of CVD prior to recruitment (self-reported angina, myocardial infarction (MI) or stroke based on specific questions, or previous hospital admission for angina, MI or stroke) (n= 9,172). For the stroke outcome this exclusion was limited to a record of stroke prior to baseline assessment (self-report or previous hospital admission for stroke) (n=2,280).

Classification of hypertension and MDD

Participants were defined as having hypertension if either: *a)* mean BP at baseline was greater than clinically-defined criteria over two measurements (systolic BP greater than or equal to 140 mmHg or diastolic BP greater than or equal to 90 mmHg. Where only one reading was available this was used (n=1,571)); or *b)* self-reported 'hypertension diagnosed by a doctor' plus self-report of currently taking antihypertensive medication. This composite classification was used to ensure that undiagnosed hypertensive participants were not misclassified and is in line with similar epidemiological studies[5 25 26]. The requirement for antihypertensive use in the context of a history of hypertension was incorporated to limit those on beta-blockers for anxiety. According to these criteria, n=68,964 participants (51.1% of the sample) had hypertension for the adverse cardiovascular outcomes analysis and n=73,671 participants (52% of the sample) had hypertension in the stroke outcome analysis.

A history of lifetime MDD was defined according to the criteria for mood disorders developed by Smith et al[24 27] and has been used in further papers[27-31]. (n=28,027 adverse cardiovascular outcomes; n=29,528 stroke outcomes). Participants were classified as having a history of MDD if they reported at least one episode, which comprised of depression and/or irritability, with a duration of at least two weeks, plus had consulted with either a general practitioner or psychiatrist for mental ill-health. This classification followed the structured diagnostic approach within the International Classification of Diseases[24] and is described in more detail within the supplementary content.

For peer review only - http://bmjopen.bmj.com/site/about/guidelines.xhtml

For the adverse cardiovascular outcomes, the remainder of the sample, with no history of hypertension or MDD (n=50,798) were classified as a comparator group. The three mutually exclusive diagnostic groups for this study were therefore: hypertension only (n=56,035); MDD only (n=15,098) and hypertension plus MDD (n= 12,929). For the stroke outcomes, the mutually exclusive groups were hypertension only (n=59,724); MDD only (n=15,581) and hypertension plus MDD (n= 13,947) and no hypertension – no MDD (n=52,502).

Outcomes

The primary outcome was defined as a first-episode cardiovascular event leading to hospital admission or death, specifically angina, MI, or chronic ischaemic heart disease (ICD-10 codes I20-I259), and transient ischaemic attack (TIA) or stroke (ICD-10 codes I60-69 and G45- G46). A secondary outcome was defined as stroke leading to hospital admission or death (ICD-10 codes I60-69 and G45- G46)[32] due to the strength of relationship hypertension has with this outcome in particular[9]. Admission data were obtained from Hospital Episode Statistics in England, Patient Episode Database for Wales and Scottish Morbidity Records in Scotland. Mortality outcomes were obtained from the National Health Service (NHS) Information Centre for England and Wales and from the NHS Central Register for Scotland. Individuals who died from a non-cardiovascular cause/stroke were censored at the time of death but not recorded as having an event. Admission data were available for Scottish, English and Welsh participants until 31 August 2014, 31 March 2015 and 28 February 2015 respectively. End of follow-up was classified as these dates unless preceded by date of death or the date of first cardiovascular admission.

Confounding variables

Information on potential confounding factors was available for age, sex, socioeconomic status (Townsend score)[33], self-reported ethnicity, age of leaving full-time education, diabetes, body mass index (BMI), systolic BP, hypercholesterolemia, alcohol use, smoking history, sedentary behaviour

For peer review only - http://bmjopen.bmj.com/site/about/guidelines.xhtml

171 (number of hours each day spent sitting at a computer, television or driving), physical activity levels[34]
172 and psychotropic medication use. Specific details on these variables are provided in supplementary
173 content.

174 **Analyses**

175 Baseline characteristics were compared between groups using Chi-squared tests for categorical
176 variables and Kruskal Wallis for continuous variables. Confounding variables were assessed for
177 differences in adverse cardiovascular outcomes using log rank sums. For the four groups of interest
178 we assessed associations with adverse cardiovascular outcomes using Cox proportional hazard
179 regression and the Efron method for ties[35]. Models were applied in a staged process in line with
180 previous studies[3-5] and reported as unadjusted (model one), partially adjusted (model two) and fully
181 adjusted (model three). Model two adjusted for sociodemographic factors (age, sex, Townsend
182 score, age of leaving full time education and ethnicity) and model three additionally adjusted for
183 health and lifestyle factors (diabetes, hypercholesterolemia, BMI, smoking history, alcohol use,
184 systolic BP, sedentary hours per day, physical activity and psychotropic medication use). The
185 proportionality of hazard assumption was assessed using Schoenfeld residuals[36]. We compared our
186 fully adjusted models with results from competing risk analyses using the Fine and Grey approach[37],
187 incorporating non-cardiovascular deaths as a competing event for cardiovascular events, and non-
188 stroke deaths for stroke events. The relative excess risk due to interaction (RERI)[38] was calculated to
189 assess for additivity in the risk. This was done at each month where the proportionality assumption
190 for the variables of interest was not met. All analyses were performed with Stata statistical software,
191 version 12[39] with the exception of RERI which was calculated using the Microsoft Excel method of
192 Andersson and colleagues, which allows for comparison of adjusted outcomes[40]. Presence of
193 multiplicative interaction was calculated using the likelihood ratio test.[41]

194 Psychotropic medication use was included as a confounding variable because of reports that they
195 may increase risk of mortality[42] but we also conducted a sensitivity analysis which excluded

For peer review only - http://bmjopen.bmj.com/site/about/guidelines.xhtml

participants who were taking psychotropic medication. Sub-group analyses looking separately at hazard ratios (HR) in male and female groups only was also carried out to assess for any gender specific differences in light of differing rates of depression and adverse cardiovascular events in each gender[43, 24].

Time-varying coefficients.

In the context of Schoenfeld residuals showing non-proportionality, models with time varying coefficients were used. In addition, log(-log) plots were carried out to find the time point at which the proportionality assumption failed. Following this, the data will be stratified by time at this time point, effectively creating two separate survival analyses pre and post the failure time point.

Patient involvement

Although patients were not directly involved with the design of the specific research questions in this study, the hypotheses tested were developed in the context of clinical experience that depression and hypertension may interact to impact on CVD. UK Biobank has an active and ongoing programme of participant involvement: www.ukbiobank.ac.uk/participants/. The outcome measures used were those provided by the UK Biobank data collection protocol, the design of which had input from participants. UK Biobank also has a website and social media streams to disseminate research findings and hosts an annual scientific meeting, which includes cohort participants.

For peer review only

RESULTS

The final sample for adverse cardiovascular outcome included 134,860 participants followed for a median duration of 63 months (702,901.6 person-years follow-up, mean 62.5 months). In total 3,685 (2.73%) participants had a first-episode cardiovascular event during the follow-up period (total number of all deaths plus non-fatal cardiovascular events = 5,788) and 910 (0.64%) participants had a first-episode stroke event (total number of all deaths plus non-fatal stroke events = 7,317).

For peer review only - http://bmjopen.bmj.com/site/about/guidelines.xhtml

Table 1 describes the baseline characteristics of the four groups. In general, the hypertension only and comorbid hypertension plus MDD groups were older, had higher BMI and were more likely to have diabetes and hypercholesterolemia. The MDD only and comorbid hypertension plus MDD groups had a higher proportion of women and were more likely to be current smokers (table 1). Gender-separated descriptive tables are shown in the supplementary content (Supplementary tables 1 and 2).

The sample for stroke-specific outcomes included 141,754 participants followed for a median duration of 63 months (735247.7 person-years follow-up, mean 62.2 months). Table 2 describes the baseline characteristics of the four groups which display similar characteristics to the adverse CVD outcome groups. Gender-separated descriptive tables are shown in the supplementary content (Supplementary tables 3 and 4).

Adverse cardiovascular outcomes

Within the main analysis and the female only subgroup analysis, MDD failed the proportional hazards assumption. Table 3 presents unadjusted and multivariate-adjusted Hazard ratios (aHR) for adverse cardiovascular outcomes. In the fully adjusted model, relative to the comparator group, the aHR for adverse cardiovascular outcomes was significantly raised for hypertension only (aHR=1. 36, 95%CI 1.22-1.52) and higher still for comorbid hypertension plus MDD (aHR=1.66, 95%CI 1.46-1.9) but reduced for MDD only (aHR=0.55, 95%CI 0.46-0.76). Although the MDD only HR was noted to increase over time as a time-varying coefficient. With the exception of MDD, these findings were robust to sensitivity-analysis excluding those on psychotropic medication (sensitivity analysis aHR=1.43, 95%CI 1.27-1.62; aHR=1.72, 95%CI 1.49-1.999, aHR=0.74, 95%CI 0.52-1.06 respectively). Table 4 presents HRs and aHRs for adverse cardiovascular outcomes using the hypertension only group as comparator. In the fully adjusted model, relative to hypertension, the aHR for adverse cardiovascular outcomes was significantly raised for comorbid hypertension plus MDD (aHR=1.22,

For peer review only

For peer review only - http://bmjopen.bmj.com/site/about/guidelines.xhtml

95%CI 1.1-1.35, sensitivity-analysis aHR= 1.20, 95%CI 1.08-1.34). An adjusted survival plot is shown in figure 1.

Within the sub-analysis, the male-only model showed a significant increase in HR for hypertension (male aHR 1.29, 95% CI 1.13-1.47) (supplementary table 5) and comorbid MDD and hypertension (male aHR 1.47, 95%CI 1.24-1.74). However, the difference between comorbid disease and hypertension only was not statistically significant (aHR 1.14, 95%CI 0.995-1.3). The female only sub-analysis showed an increase in HR for hypertension (aHR 1.64, 95%CI 1.33-2.02) and a greater increase in comorbid MDD and hypertension (aHR 2.18, 95%CI 1.82-2.92) (table 6 of the supplementary content). The difference between comorbid disease and hypertension only was also statistically significant (aHR 1.33, 95%CI 1.14- 1.56). Sensitivity analysis supported these findings.

Stroke Outcomes

None of the independent variables for stroke outcome failed the proportionality assumption. Table 5 presents HRs and aHRs for stroke outcomes. In the fully adjusted model, the aHR for stroke was insignificantly raised for hypertension only (aHR=1.21, 95%CI 0.97-1.51) and depression only (aHR=1.20, 95%CI 0.89-1.63) but significantly raised for comorbid hypertension plus MDD (aHR=1.37, 95%CI 1.04-1.79). In the hypertension comparator group, no group was significantly different from hypertension only (table 6). Similar trends were shown in the gender subset analysis but not reaching significance (supplementary Tables 7-8). An adjusted survival plot is shown in figure 2. Again, all results were supported by sensitivity analysis excluding those on psychotropic medication.

Interaction, time stratified analysis and competing risk analysis

Survival analysis stratified by time is described and included within the supplementary content (supplementary tables 9, 10 and figure 3). There was evidence of interaction between hypertension and MDD at baseline for the overall cardiovascular outcome analysis before the 22.5 month time point (RERI=0.563, 95%CI 0.189 - 0.938. Likelihood ratio p-value 0.0116) and the female only

For peer review only - http://bmjopen.bmj.com/site/about/guidelines.xhtml

cardiovascular endpoint analysis before the 29 month time point (RERI=0.588, 95%CI 0.074 - 1.103. Likelihood ratio p-value 0.031). However, after these time points there was no evidence of interaction. Supplementary table 11 shows the full results for this analysis. Competing risk analysis showed no significant difference from the main analyses for cardiovascular outcomes or stroke outcomes (tables 7-8)

DISCUSSION

In this large population cohort of middle-aged adults without CVD (adjusted for a broad range of confounders), individuals with co-morbid hypertension and MDD were at increased risk of CVD when compared to those with hypertension alone, MDD alone and neither condition. There was some evidence of interaction between hypertension and MDD at baseline, but not throughout follow-up and only within both subgroups. Differences between co-morbid disease and either disease alone or no disease were more marked in females. For stroke outcomes, comorbid depression and hypertension was the only group that showed significantly increased HRs.

Previous research

Our findings expand upon previous research from UK Biobank looking at CVD in those with BD and MDD[27]. It was found that there were significantly increased odds of having 'any CVD' (fully adjusted OR 1.15 CI 1.12–1.19) or hypertension (fully adjusted OR 1.15 CI 1.13–1.18) if depressed, with even higher odds for stroke (fully adjusted OR 1.26 CI 1.13–1.40). There are distinct differences between our current paper and the previous publication. Follow-up data within UK-Biobank has been released to allow meaningful prospective studies be conducted. Thus, the current paper has the benefits of using hospital records and death certification for outcomes, rather than self-reported data. We are also able to make inferences about the direction of effect regarding MDD and CVD and assess the

For peer review only - http://bmjopen.bmj.com/site/about/guidelines.xhtml

influence of hypertension and MDD over time, both in isolation and when comorbid, and assess for statistical interaction to inform on whether there may be a biological interaction.

Other survival analyses in hypertension/MDD comorbidity have focussed primarily on mortality outcomes. In the National Health and Nutrition Epidemiologic Follow-up Study in the United States[31] and the Taiwanese Survey of Health and Living Status[32], individuals with self-reported hypertension plus depressive symptoms (compared to a reference group with neither) had increased all-cause mortality (aHR=1.39, 95%CI 1.14-1.69, aHR=1.54, 95%CI 1.29-1.83, respectively) [3 4] with the former also showing increased CVD specific mortality (aHR=1.59, 95%CI 1.08-2.34)[4]. Similarly, Hamer and colleagues[5] reported a prospective analysis of common mental disorder on mortality outcomes in individuals with hypertension versus those without hypertension in participants from the Health Survey for England and the Scottish Health Survey (1994–2004), finding that risk of CVD death was highest in the group with comorbid disease.

Strengths

These observations are broadly consistent with our results but our study has a number of methodological advantages, including a very large sample size, adjustment of analyses for a more comprehensive range of confounders, and a focus on first-episode non-fatal and fatal adverse cardiovascular events. We also used a definition of prior MDD history which was based on diagnostic criteria within ICD-10 (rather than a threshold score on a depressive symptoms or general wellbeing scale) and our composite definition of hypertension incorporated past history, baseline medication and BP measurements. Lifetime MDD is thought to be under-reported in the literature. However, using current symptom scores may reduce power and precision because a smaller number of respondents would be identified as having an episode of MDD.[44] Given that we are assessing outcomes for which risk accumulates over a lifetime, we felt that a primary focus on lifetime episodes was appropriate. We believe our lifetime definition to be better suited as it offers a view depression and depressive symptoms over the course of a lifespan as opposed the past week. Also,

For peer review only - http://bmjopen.bmj.com/site/about/guidelines.xhtml

within our current study we were able to exclude those with previous self-declared or hospital admission CVD, as previous studies show depression may result from CVD[45 46] and worsen prognosis[46]

Limitations

However, some limitations are acknowledged. Recruitment criteria for UK Biobank may lead to selection bias. Specifically, age-restrictions may lead to underrepresentation of early-onset hypertension and those with more severe MDD may be less inclined to attend for assessment. We also acknowledge limitations with our classifications of MDD and hypertension, which were primarily self-report rather than formal diagnostic assessments. Although we have excluded prior cardiovascular events where possible, the MDD plus hypertension sub-type may capture older individuals with a degree of vascular depression, which has an established association with raised BP[47]. In addition, although we adjust for a host of risk factors at baseline such as smoking status, BMI and psychotropic medication, we are limited by the lack of follow-up data, which could show change and modification of said risk factors over time. Similarly, we were unable to assess for medication adherence and transitions from one investigatory group to another. Participants who are aware of or had sought treatment for MDD may also have complicated our findings, however, our sensitivity analysis excluded those using pharmaceutical treatments and was in keeping with our main findings. Such modifications could explain the non-proportional nature of the depression group, which may in itself be a predictor of poor medication adherence[48]. Although adherence to medication was not formally assessed, the number and duration of antihypertensive medications used in the hypertension plus MDD group was the same as for the hypertension only group (supplementary content, table 12). As such, worse outcomes in the MDD plus hypertension group are not explained by less intensive antihypertensive treatment at baseline. The end-points used for stroke and cardiovascular events also require to be further validated, however are in line with previous epidemiological studies[5] and have been suggested in previous papers in UK Biobank[32].

For peer review only - http://bmjopen.bmj.com/site/about/guidelines.xhtml

Cardiovascular endpoints have not, to our knowledge, been validated within UKbiobank, however we do not feel that this will bias the results towards any particular group. The amelioration of the aHR suggests other covariates contribute considerably to the risk. This is important in the context of increased rates of diabetes, hypercholesterolemia and obesity along with lower socio-economic status in the hypertension only and comorbid groups and as such we may be seeing the summation of CV risk factors. Finally, the overall recruitment rate to UK Biobank was low (at around 6%); however, the large final cohort size, the depth and diversity of phenotype data collected at baseline, and the wide sociodemographic representation of participants all make our findings highly relevant to UK primary care settings. While UK Biobank participants cannot be used to provide representative disease prevalence and incidence rates, valid assessment of exposure-disease relationships are nonetheless widely generalizable and do not require participants to be representative of the UK population at large[49], although findings will not be generalizable to other countries.

Possible mechanisms

Our finding that a history of MDD, in the context of a current diagnosis of hypertension increased the risk of first-episode CVD is complicated by the time varying risk that MDD conveys to CVD. Sub-sample analysis show this time-varying aspect is gender-specific to females. Within our sample, the MDD group has a slightly reduced BP compared to comparators. Previously, reduced BP has been put forth as being causative of MDD and therefore reducing CVD risk[20], but findings from longitudinal studies are inconsistent with regards to direction of effect[50 51]. Potential menopausal effects are tempting explanations. Common factors for BP and mood such as neuropeptide Y[52 53] may also influence cardiovascular outcomes. Neuropeptide Y has a complex relationship with oestrogen[54] and both have dampening effect on the SNS[55].

Personality factors may also play a role. MDD correlates highly with neuroticism which, although inconsistent, may be protective of CVD[56]. Conscientiousness traits may lead to better outcomes[57]

For peer review only - http://bmjopen.bmj.com/site/about/guidelines.xhtml

and it is possible that this trait has been selected for within UK Biobank. Despite this early reduced risk, due to the time varying nature of MDD, MDD has increased risk in the latter aspects of the time-stratified analyses for the full and female only analyses (supplementary table 9 and 10). The findings from our study in this context suggest MDDs role as a risk factor for CVD and its relationship with BP may be much more complex than initially thought, in particular within female populations however further investigation is clearly needed.

We can see in the hypertension only baseline models that comorbid hypertension and depression convey a significantly greater risk than hypertension alone. Individuals with either hypertension or depression may have increased sympathetic stimulation that is increased further in comorbid states leading to worse outcomes[58].

CONCLUSIONS

Overall, our findings may have important implications for routine clinical practice, particularly within primary care settings and further demonstrate the complex relationship between depression and hypertension. Although evidence of an interaction is inconsistent, we found that comorbid hypertension and depression conferred greater hazard than hypertension alone for adverse cardiovascular outcomes. This significant finding remained after adjustment for factors such as BMI, smoking status and diabetes and was robust to sensitivity analysis excluding those on psychotropic medication. One implication is that clinicians should be more aware of the negative long-term impact on CVD outcomes caused by a history of MDD in the context of hypertension. Although this work awaits replication and testing in other cohorts and settings, further work in this field may suggest that future iterations of CVD risk prediction tools, such as ASSIGN[59], would benefit from the addition of a question on whether individuals have a past history of MDD, to facilitate more intensive support to prevent CVD[60].

For peer review only - http://bmjopen.bmj.com/site/about/guidelines.xhtml

ACKNOWLEDGEMENTS

We are grateful to all participants of the UK Biobank cohort. UK Biobank was established by the Wellcome Trust, the Medical Research Council, Department of Health, Scottish Government and the Northwest Regional Development Agency. It has also had funding from the Welsh Assembly Government and the British Heart Foundation. UK Biobank is hosted by the University of Manchester and supported by the National Health Service (NHS). NG is supported by the Aitchison Family Clinical Research Fellowship at the University of Glasgow and DJS is supported by a Lister Institute Prize Fellowship. JC is supported by the Sackler Trust and the Wellcome Trust. Funding also acknowledged from MRC Mental Health Data Pathfinder Award to DJS (MC_PC_17217).

Footnotes

Authors Statement: Contributors NG, JW, JP, JC, DS, SP and DM, contributed to study design and writing of the manuscript. JP and DM contributed to data acquisition. NG conducted data processing and statistical analyses.

Funding: Authors declare no support from any organisation for the submitted work;

Competing interests: All authors have completed the ICMJE uniform disclosure form at http://www.icmje.org/coi_disclosure.pdf and no financial relationships with any organisations that might have an interest in the submitted work in the previous three years; no other relationships or activities that could appear to have influenced the submitted work.

Ethics approval: This study has been conducted using UK Biobank data. UK Biobank has received ethics approval from the UK Biobank Research Ethics Committee (ref. 11/NW/0382).

Data sharing statement: The data used in this study are available via a direct application to UK Biobank.

For peer review only

Transparency statement: The lead author affirms that this manuscript is an honest, accurate and transparent account of the study being reported; that no important aspects of the study have been omitted; and that any discrepancies from the study as planned (and, if relevant, registered) have been explained.

COMPETING INTERESTS STATEMENTS

All authors have completed the ICMJE uniform disclosure form at http://www.icmje.org/coi_disclosure.pdf and no financial relationships with any organisations that might have an interest in the submitted work in the previous three years; no other relationships or activities that could appear to have influenced the submitted work.

For peer review only

For peer review only - http://bmjopen.bmj.com/site/about/guidelines.xhtml

References

1. Organization. WH. The global burden of disease: 2004 update. Geneva, Switzerland.: WHO press. 2008.
2. Hare DL, Toukhsati SR, Johansson P, et al. Depression and cardiovascular disease: a clinical review. *Eur Heart J* 2014;35(21):1365-72. doi: 10.1093/eurheartj/eht462
3. Kuo PL, Pu C. The contribution of depression to mortality among elderly with self-reported hypertension: analysis using a national representative longitudinal survey. *J Hypertens* 2011;29(11):2084-90. doi: 10.1097/HJH.0b013e32834b59ad [published Online First: 2011/09/22]
4. Axon RN, Zhao Y, Egede LE. Association of depressive symptoms with all-cause and ischemic heart disease mortality in adults with self-reported hypertension. *Am J Hypertens* 2010;23(1):30-7. doi: 10.1038/ajh.2009.199
5. Hamer M, Batty GD, Stamatakis E, et al. The combined influence of hypertension and common mental disorder on all-cause and cardiovascular disease mortality. *J Hypertens* 2010;28(12):2401-6. doi: 10.1097/HJH.0b013e32833e9d7c
6. Jani BD, Cavanagh J, Barry SJ, et al. Relationship Between Blood Pressure Values, Depressive Symptoms, and Cardiovascular Outcomes in Patients With Cardiometabolic Disease. *Journal of clinical hypertension (Greenwich, Conn)* 2016;18(10):1027-35. doi: 10.1111/jch.12813 [published Online First: 2016/04/05]
7. Taylor CB, Youngblood ME, Catellier D, et al. Effects of antidepressant medication on morbidity and mortality in depressed patients after myocardial infarction. *Arch Gen Psychiatry* 2005;62(7):792-8. doi: 10.1001/archpsyc.62.7.792 [published Online First: 2005/07/06]
8. Kearney PM, Whelton M, Reynolds K, et al. Global burden of hypertension: analysis of worldwide data. *Lancet* 2005;365(9455):217-23. doi: 10.1016/S0140-6736(05)17741-1
9. Lawes CM, Vander Hoorn S, Rodgers A, et al. Global burden of blood-pressure-related disease, 2001. *Lancet* 2008;371(9623):1513-8. doi: 10.1016/S0140-6736(08)60655-8
10. Meng L, Chen D, Yang Y, et al. Depression increases the risk of hypertension incidence: a meta-analysis of prospective cohort studies. *J Hypertens* 2012;30:842 - 51.
11. Wu EL, Chien IC, Lin CH, et al. Increased risk of hypertension in patients with major depressive disorder: a population-based study. *J Psychosom Res* 2012;73(3):169-74. doi: 10.1016/j.jpsychores.2012.07.002
12. Li Z, Li Y, Chen L, et al. Prevalence of Depression in Patients With Hypertension: A Systematic Review and Meta-Analysis. *Medicine (Baltimore)* 2015;94(31):e1317. doi: 10.1097/md.0000000000001317 [published Online First: 2015/08/08]
13. Johnson AD, Newton-Cheh C, Chasman DI, et al. Association of hypertension drug target genes with blood pressure and hypertension in 86,588 individuals. *Hypertension* 2011;57(5):903-10. doi: 10.1161/HYPERTENSIONAHA.110.158667
14. Casamassima F HJ, Fava M, Sachs GS, Smoller JW, Cassano GB, Lattanzi L, Fagerness J, Stange JP, Perlis RH. Phenotypic effects of a bipolar liability gene among individuals with major depressive disorder. . *Am J Med Genet B Neuropsychiatr Genet* 2010;153B:303-09.
15. Green EK, Grozeva D, Jones I, et al. The bipolar disorder risk allele at CACNA1C also confers risk of recurrent major depression and of schizophrenia. *Molecular Psychiatry* 2010;15(10):1016-22. doi: 10.1038/mp.2009.49
16. Ferreira MA, O'Donovan MC, Meng YA, et al. Collaborative genome-wide association analysis supports a role for ANK3 and CACNA1C in bipolar disorder. *Nat Genet* 2008;40(9):1056-8. doi: 10.1038/ng.209
17. Consortium WTCC. Identification of risk loci with shared effects on five major psychiatric disorders: a genome-wide analysis. *The Lancet* 2013;381(9875):1371-79.

For peer review only - http://bmjopen.bmj.com/site/about/guidelines.xhtml

18. Scalco AZ, Scalco MZ, Azul JB, et al. Hypertension and depression. *Clinics (Sao Paulo)* 2005;60(3):241-50. doi: /S1807-59322005000300010

19. Boal AH, Smith DJ, McCallum L, et al. Monotherapy With Major Antihypertensive Drug Classes and Risk of Hospital Admissions for Mood Disorders. *Hypertension* 2016;68(5):1132-38. doi: 10.1161/hypertensionaha.116.08188 [published Online First: 2016/10/14]

20. Licht CM, de Geus EJ, Seldenrijk A, et al. Depression is associated with decreased blood pressure, but antidepressant use increases the risk for hypertension. *Hypertension* 2009;53(4):631-8. doi: 10.1161/HYPERTENSIONAHA.108.126698

21. Crookes DM, Demmer RT, Keyes KM, et al. Depressive Symptoms, Antidepressant Use, and Hypertension in Young Adulthood. *Epidemiology (Cambridge, Mass)* 2018;29(4):547-55. doi: 10.1097/ede.0000000000000840 [published Online First: 2018/04/10]

22. Diminic-Lisica I, Popovic B, Rebic J, et al. Outcome of treatment with antidepressants in patients with hypertension and undetected depression. *Int J Psychiatry Med* 2014;47(2):115-29. doi: 10.2190/PM.47.2.c [published Online First: 2014/08/03]

23. Sudlow C, Gallacher J, Allen N, et al. UK biobank: an open access resource for identifying the causes of a wide range of complex diseases of middle and old age. *PLoS Med* 2015;12(3):e1001779. doi: 10.1371/journal.pmed.1001779

24. Smith DJ, Nicholl BI, Cullen B, et al. Prevalence and characteristics of probable major depression and bipolar disorder within UK biobank: cross-sectional study of 172,751 participants. *PLoS One* 2013;8(11):e75362. doi: 10.1371/journal.pone.0075362

25. Ong KL, Cheung BM, Man YB, et al. Prevalence, awareness, treatment, and control of hypertension among United States adults 1999-2004. *Hypertension* 2007;49(1):69-75. doi: 10.1161/01.hyp.0000252676.46043.18 [published Online First: 2006/12/13]

26. Egan BM, Zhao Y, Axon RN. US trends in prevalence, awareness, treatment, and control of hypertension, 1988-2008. *Jama* 2010;303(20):2043-50. doi: 10.1001/jama.2010.650 [published Online First: 2010/05/27]

27. Martin DJ, Ul-Haq Z, Nicholl BI, et al. Cardiometabolic disease and features of depression and bipolar disorder: population-based, cross-sectional study. *Br J Psychiatry* 2016;208(4):343-51. doi: 10.1192/bjp.bp.114.157784

28. Ul-Haq Z, Smith DJ, Nicholl BI, et al. Gender differences in the association between adiposity and probable major depression: a cross-sectional study of 140,564 UK Biobank participants. *BMC psychiatry* 2014;14:153-53. doi: 10.1186/1471-244X-14-153

29. Sarkar C, Webster C, Gallacher J. Residential greenness and prevalence of major depressive disorders: a cross-sectional, observational, associational study of 94 879 adult UK Biobank participants. *The Lancet Planetary Health* 2018;2(4):e162-e73. doi: https://doi.org/10.1016/S2542-5196(18)30051-2

30. Hall LS, Adams MJ, Arnau-Soler A, et al. Genome-wide meta-analyses of stratified depression in Generation Scotland and UK Biobank. *Translational psychiatry* 2018;8(1):9-9. doi: 10.1038/s41398-017-0034-1

31. Howard DM, Adams MJ, Shirali M, et al. Genome-wide association study of depression phenotypes in UK Biobank identifies variants in excitatory synaptic pathways. *Nature Communications* 2018;9(1):1470. doi: 10.1038/s41467-018-03819-3

32. Woodfield R, Grant I, Group UKBSO, et al. Accuracy of Electronic Health Record Data for Identifying Stroke Cases in Large-Scale Epidemiological Studies: A Systematic Review from the UK Biobank Stroke Outcomes Group. *PLOS ONE* 2015;10(10):e0140533. doi: 10.1371/journal.pone.0140533

33. Townsend P. Deprivation. *Journal of Social Policy* 2009;16(02):125. doi: 10.1017/s0047279400020341

34. Guo W, Bradbury KE, Reeves GK, et al. Physical activity in relation to body size and composition in women in UK Biobank. *Annals of Epidemiology* 2015;25(6):406-13.e6. doi: http://dx.doi.org/10.1016/j.annepidem.2015.01.015

For peer review only - http://bmjopen.bmj.com/site/about/guidelines.xhtml

35. Efron B. The Efficiency of Cox's Likelihood Function for Censored Data. *Journal of the American Statistical Association,* 1977;72(359):557-65.
36. Schoenfeld D. Partial Residuals for the Proportional Hazards Regression-Model. *Biometrika* 1982;69(1):239-41. doi: Doi 10.2307/2335876
37. Fine JP, Gray RJ. A Proportional Hazards Model for the Subdistribution of a Competing Risk. *Journal of the American Statistical Association* 1999;94(446):496-509. doi: 10.2307/2670170
38. Hosmer DW, Lemeshow S. Confidence interval estimation of interaction. *Epidemiology (Cambridge, Mass)* 1992;3(5):452-6. [published Online First: 1992/09/01]
39. Stata Statistical Software, version 12 [program]. College station, Texas.
40. Andersson T, Alfredsson L, Kallberg H, et al. Calculating measures of biological interaction. *European journal of epidemiology* 2005;20(7):575-9. [published Online First: 2005/08/27]
41. Marshall SW. Power for tests of interaction: effect of raising the Type I error rate. *Epidemiologic Perspectives & Innovations* 2007;4(1):4. doi: 10.1186/1742-5573-4-4
42. Weich S, Pearce HL, Croft P, et al. Effect of anxiolytic and hypnotic drug prescriptions on mortality hazards: retrospective cohort study. *BMJ* 2014;348:g1996. doi: 10.1136/bmj.g1996
43. Nichols M, Townsend N, Scarborough P, et al. Cardiovascular disease in Europe 2014: epidemiological update. *European Heart Journal* 2014;35(42):2950-59. doi: 10.1093/eurheartj/ehu299
44. Patten SB. Accumulation of major depressive episodes over time in a prospective study indicates that retrospectively assessed lifetime prevalence estimates are too low. *BMC psychiatry* 2009;9:19-19. doi: 10.1186/1471-244X-9-19
45. Kang HJ, Stewart R, Bae KY, et al. Predictive value of homocysteine for depression after acute coronary syndrome. *Oncotarget* 2016;7(42):69032-40. doi: 10.18632/oncotarget.11966
46. Lichtman JH, Froelicher ES, Blumenthal JA, et al. Depression as a risk factor for poor prognosis among patients with acute coronary syndrome: systematic review and recommendations: a scientific statement from the American Heart Association. *Circulation* 2014;129(12):1350-69. doi: 10.1161/CIR.0000000000000019
47. Aizenstein HJ, Baskys A, Boldrini M, et al. Vascular depression consensus report – a critical update. *BMC Medicine* 2016;14(1):161. doi: 10.1186/s12916-016-0720-5
48. Goldstein CM, Gathright EC, Garcia S. Relationship between depression and medication adherence in cardiovascular disease: the perfect challenge for the integrated care team. *Patient preference and adherence* 2017;11:547-59. doi: 10.2147/PPA.S127277
49. Fry A, Littlejohns TJ, Sudlow C, et al. Comparison of Sociodemographic and Health-Related Characteristics of UK Biobank Participants With Those of the General Population. *American journal of epidemiology* 2017;186(9):1026-34. doi: 10.1093/aje/kwx246 [published Online First: 2017/06/24]
50. Hildrum B, Mykletun A, Holmen J, et al. Effect of anxiety and depression on blood pressure: 11-year longitudinal population study. *British Journal of Psychiatry* 2018;193(2):108-13. doi: 10.1192/bjp.bp.107.045013 [published Online First: 01/02]
51. Paterniti S. Low blood pressure and risk of depression in the elderly: A prospective community-based study. *The British Journal of Psychiatry* 2000;176(5):464-67. doi: 10.1192/bjp.176.5.464
52. Zhang P, Qi Y-X, Yao Q-P, et al. Neuropeptide Y Stimulates Proliferation and Migration of Vascular Smooth Muscle Cells from Pregnancy Hypertensive Rats via Y1 and Y5 Receptors. *PLOS ONE* 2015;10(7):e0131124. doi: 10.1371/journal.pone.0131124
53. Morales-Medina JC, Dumont Y, Quirion R. A possible role of neuropeptide Y in depression and stress. *Brain research* 2010;1314:194-205. doi: https://doi.org/10.1016/j.brainres.2009.09.077
54. Pelletier G, Li S, Luu-The V, et al. Oestrogenic Regulation of Pro-Opiomelanocortin, Neuropeptide Y and Corticotrophin-Releasing Hormone mRNAs in Mouse Hypothalamus. *Journal of Neuroendocrinology* 2007;19(6):426-31. doi: 10.1111/j.1365-2826.2007.01548.x

For peer review only - http://bmjopen.bmj.com/site/about/guidelines.xhtml

55. Rosano GM, Panina G. Oestrogens and the heart. *Therapie* 1999;54(3):381-5. [published Online First: 1999/09/29]
56. Gale CR, Čukić I, Batty GD, et al. When Is Higher Neuroticism Protective Against Death? Findings From UK Biobank. *Psychological Science* 2017;28(9):1345-57. doi: 10.1177/0956797617709813
57. Cheng H, Montgomery S, Treglown L, et al. Emotional stability, conscientiousness, and self-reported hypertension in adulthood. *Personality and Individual Differences* 2017;115:159-63. doi: https://doi.org/10.1016/j.paid.2016.02.034
58. Barton DA, Dawood T, Lambert EA, et al. Sympathetic activity in major depressive disorder: identifying those at increased cardiac risk? *J Hypertens* 2007;25(10):2117-24. doi: 10.1097/HJH.0b013e32829baae7 [published Online First: 2007/09/22]
59. Woodward M, Brindle P, Tunstall-Pedoe H. Adding social deprivation and family history to cardiovascular risk assessment: the ASSIGN score from the Scottish Heart Health Extended Cohort (SHHEC). *Heart* 2007;93(2):172-76. doi: 10.1136/hrt.2006.108167
60. Hippisley-Cox J, Coupland C, Vinogradova Y, et al. Predicting cardiovascular risk in England and Wales: prospective derivation and validation of QRISK2. *BMJ* 2008;336(7659):1475-82. doi: 10.1136/bmj.39609.449676.25

For peer review only - http://bmjopen.bmj.com/site/about/guidelines.xhtml

Table 1. Baseline characteristics for adverse cardiovascular outcomes

	Comparator group	Hypertension only	MDD only	Hypertension plus MDD
	N = 50798	N = 56035	N = 15098	N = 12929
**Median age (range)***	54 (47 - 61)	61 (55 - 65)	53 (46 - 60)	60 (53 - 64)
**Females, N (%)**	29228 (57.54%)	25893 (46.21%)	10929 (72.39%)	7676 (59.37%)
**Ethnicity, N (%)**				
White	46147 (90.84%)	51249 (91.46%)	14247 (94.36%)	12272 (94.92%)
Asian/Asian British	1771 (3.49%)	1696 (3.03%)	261 (1.73%)	179 (1.38%)
Black/ Black British	1323 (2.6%)	1769 (3.16%)	219 (1.45%)	222 (1.72%)
**Median Townsend score (range)***	-1.89 (-3.45 - 0.54)	-2.07 (-3.51 - 0.39)	-1.64 (-3.3 - 0.93)	-1.84 (-3.42 - 0.76)
**Age at leaving full-time education, N (%)**				
<16	5916 (11.65%)	12085 (21.57%)	1725 (11.43%)	2607 (20.16%)
16	10265 (20.21%)	11827 (21.11%)	3178 (21.05%)	2732 (21.13%)
>16	34090 (67.11%)	31480 (56.18%)	10090 (66.83%)	7503 (58.03%)
**Total physical activity in metabolic**	3.97 (1.68 - 8.03)	3.79 (1.51 - 8.03)	3.89 (1.66 - 8)	3.68 (1.49 - 7.95)
**Sedentary time in hours, median (range)***	4 (3 - 6)	4.5 (3.5 - 6)	4.5 (3 - 6)	5 (3.5 - 6)

For peer review only - http://bmjopen.bmj.com/site/about/guidelines.xhtml

**Diabetes, N (%)**	1268	(2.5%)	3777	(6.74%)	380	(2.52%)	929	(7.19%)
**Hypercholesterolaemia, N (%)**	3011	(5.93%)	9210	(16.44%)	893	(5.91%)	2211	(17.1%)
**Systolic BP in mmHg, median (range)***	125.5	(118 - 132)	149.5	(142 - 159.5)	124	(116 - 131)	147.5	(140.5 - 157.)
**Body Mass Index, N (%)**								
<18.5	389	(0.77%)	142	(0.25%)	103	(0.68%)	34	(0.26%)
18.5 – 25	22549	(44.39%)	13678	(24.41%)	6251	(41.4%)	2874	(22.23%)
25-30	20410	(40.18%)	25216	(45 %)	5936	(39.32%)	5389	(41.68%)
>30	7450	(14.67%)	16999	(30.34%)	2808	(18.6%)	4632	(35.83%)
**Smoking status, N (%)**								
Never smoked	30626	(60.29%)	31503	(56.22%)	7864	(52.09%)	6454	(49.92%)
Previously smoked	15056	(29.64%)	20140	(35.94%)	5118	(33.9%)	5065	(39.18%)
Current smoker	4970	(9.78%)	4199	(7.49%)	2093	(13.86%)	1381	(10.68%)
**Alcohol frequency, N (%)**								
Daily or almost daily	9450	(18.6%)	12970	(23.15%)	2736	(18.12%)	2881	(22.28%)
Three or four times a week	12175	(23.97%)	13033	(23.26%)	3253	(21.55%)	2837	(21.94%)
Once or twice a week	13644	(26.86%)	13889	(24.79%)	3880	(25.7%)	2916	(22.55%)

For peer review only - http://bmjopen.bmj.com/site/about/guidelines.xhtml

One to three times a month	6052	(11.91%)	5588	(9.97%)	2058	(13.63%)	1512	(11.69%)
Special occasions only	5534	(10.89%)	6330	(11.3%)	1904	(12.61%)	1729	(13.37%)
Never	3924	(7.72%)	4199	(7.49%)	1262	(8.36%)	1048	(8.11%)
**Psychotropic medication, N (%)**	1341	(2.64%)	1795	(3.2%)	2844	(18.84%)	2522	(19.51%)

*All data presented as N (%) and has chi-squared p-value of <0.001 except * which are median values (interquartile range) and have a Kruskal-Wallis p-value of 0.0001. Data presented as MET-hrs (hours spent doing exercise adjusted for multiples of basal metabolic rate in accordance with IPAQ).Townsend score is an area based measure based on census statistics. It is a calculation based on the number of: households without a car, overcrowded households, households not owner-occupied and unemployment.*

For peer review only - http://bmjopen.bmj.com/site/about/guidelines.xhtml

Table 2 Baseline characteristics for stroke outcomes

	Comparator group	Hypertension only	MDD only	Hypertension plus MDD
	N = 52502	N = 59724	N = 15581	N = 13947
**Median age (range)***	54 (47 - 61)	61 (55 - 65)	54 (47 - 61)	60 (53 - 64)
**Females, N (%)**	29684 (56.54%)	26937 (45.1%)	11143 (71.52%)	8090 (58.01%)
**Ethnicity, N (%)**				
White	47697 (90.85%)	54578 (91.38%)	14697 (94.33%)	13212 (94.73%)
Asian/Asian British	1857 (3.54%)	1889 (3.16%)	280 (1.8%)	209 (1.5%)
Black/ Black British	1355 (2.58%)	1854 (3.1%)	223 (1.43%)	246 (1.76%)
**Median Townsend score (range)***	-1.89 (-3.45 - 0.55)	-2.04 (-3.49 - 0.44)	-1.56 (-3.28 - 1.15)	-1.74 (-3.4 - 0.93)
**Age at leaving full-time education, N (%)**				
<16	6446 (12.28%)	13396 (22.43%)	1884 (12.09%)	2945 (21.12%)
16	10590 (20.17%)	12507 (20.94%)	3270 (20.99%)	2953 (21.17%)
>16	34914 (66.5%)	33114 (55.45%)	10317 (66.22%)	7947 (56.98%)
**Total physical activity in metabolic**	3.96 (1.67 - 8.02)	3.75 (1.5 - 8)	4.13 (1.67 - 8.36)	3.66 (1.45 - 7.83)
**Sedentary time in hours, median (range)***	4 (3 - 6)	5 (3.5 - 6)	5 (3.5 - 6.5)	5 (4 - 7)

For peer review only - http://bmjopen.bmj.com/site/about/guidelines.xhtml

**Diabetes, N (%)**	1454	(2.77%)	4502	(7.54%)	449	(2.88%)	1163	(8.34%)
**Hypercholesterolaemia, N (%)**	3592	(6.84%)	10768	(18.03%)	1049	(6.73%)	2620	(18.79%)
**Systolic BP in mmHg, median (range)***	125.5	(118 - 132)	149.5	(142 - 159.5)	127	(120.5 - 133)	147.5	(140.5 - 156.5)
**Body Mass Index, N (%)**								
<18.5	395	(0.75%)	151	(0.25%)	104	(0.67%)	38	(0.27%)
18.5 – 25	22967	(43.75%)	14242	(23.85%)	6374	(40.91%)	3017	(21.63%)
25-30	21185	(40.35%)	26817	(44.9%)	6149	(39.46%)	5769	(41.36%)
>30	7953	(15.15%)	18514	(31.%)	2954	(18.96%)	5123	(36.73%)
**Smoking status, N (%)**								
Never smoked	31318	(59.65%)	32982	(55.22%)	8052	(51.68%)	6834	(49%)
Previously smoked	15851	(30.19%)	22019	(36.87%)	5340	(34.27%)	5560	(39.87%)
Current smoker	5170	(9.85%)	4501	(7.54%)	2163	(13.88%)	1519	(10.89%)
**Alcohol frequency, N (%)**								
Daily or almost daily	9760	(18.59%)	13751	(23.02%)	2817	(18.08%)	3085	(22.12%)
Three or four times a week	12563	(23.93%)	13827	(23.15%)	3335	(21.4%)	3020	(21.65%)
Once or twice a week	14089	(26.84%)	14719	(24.65%)	3993	(25.63%)	3125	(22.41%)

For peer review only - http://bmjopen.bmj.com/site/about/guidelines.xhtml

One to three times a month	6220	(11.85%)	5971	(10%)	2122	(13.62%)	1627	(11.67%)
Special occasions only	5744	(10.94%)	6794	(11.38%)	1978	(12.69%)	1885	(13.52%)
Never	4102	(7.81%)	4630	(7.75%)	1330	(8.54%)	1199	(8.6%)
**Psychotropic medication, N (%)**	1408	(2.68%)	1996	(3.34%)	2976	(19.1%)	2778	(19.92%)

*All data presented as N (%) and has chi-squared p-value of <0.001 except * which are median values (interquartile range) and have a Kruskal-Wallis p-value of 0.0001. Data presented as MET-hrs (hours spent doing exercise adjusted for multiples of basal metabolic rate in accordance with IPAQ).Townsend score is an area based measure based on census statistics. It is a calculation based on the number of: households without a car, overcrowded households, households not owner-occupied and unemployment.*

For peer review only

Table 3: Risk of adverse cardiovascular event by clinical group: unadjusted, partially adjusted and fully adjusted models.

	Unadjusted			Model 1 - Sociodemographic			Model 2 - Model 1 + Health/ Lifestyle		
**Group**	HR	95% C.I.	p-value	aHR	95% C.I.	p-value	aHR	95% C.I.	p-value
No Hypertension - No MDD	1(ref)			1(ref)			1(ref)		
Hypertension only	2.60	(2.39 - 2.82)	3.31×10^{-113}	1.72	(1.57 - 1.88)	1.99×10^{-33}	1.36	(1.22 - 1.52)	2.92×10^{-8}
MDD only	0.69	(0.51 - 0.94)	0.02	0.82	(0.6 - 1.13)	0.23	0.75	(0.54 - 1.04)	0.08
Hypertension and MDD	2.84	(2.55 - 3.17)	6.31×10^{-77}	2.27	(2.02 - 2.55)	2.75×10^{-44}	1.66	(1.45 - 1.9)	7.48×10^{-14}
**Time varying Variables**									
MDD only	1.01	(1.004 - 1.02)	2.38×10^{-3}	1.01	(1.004 - 1.02)	3.19×10^{-3}	1.01	(1.004 - 1.02)	3.03×10^{-3}

**Adjusted for sociodemographic factors (age, sex, Townsend score, age of leaving full time education and ethnicity. †Additionally adjusted for history of diabetes, history of hypercholesterolemia, BMI, smoking history, alcohol use, systolic blood pressure, sedentary hours per day, physical activity and psychotropic medication use. MDD = Major depressive disorder, HR = Hazard ratio, aHR = Adjusted hazard ratio, C.I. = Confidence interval.*

For peer review only

Table 4: Risk of adverse cardiovascular event by clinical group: unadjusted, partially adjusted and fully adjusted models with hypertension as the comparator

	Unadjusted			Model 1 - Sociodemographic			Model 2 - Model 1 + Health/ Lifestyle		
**Group**	HR	95% C.I.	p-value	aHR	95% C.I.	p-value	aHR	95% C.I.	p-value
Hypertension only	1(ref)			1(ref)			1(ref)		
No Hypertension - No MDD	0.38	(0.35 - 0.42)	3.31×10^{-113}	0.58	(0.53 - 0.63)	1.99×10^{-33}	0.73	(0.66 - 0.82)	2.92×10^{-8}
MDD only	0.27	(0.2 - 0.36)	1.14×10^{-17}	0.48	(0.35 - 0.66)	4.91×10^{-6}	0.55	(0.4 - 0.76)	3.23×10^{-4}
Hypertension and MDD	1.09	(0.996 - 1.2)	0.06	1.32	(1.2 - 1.46)	3.07×10^{-8}	1.22	(1.1 - 1.35)	1.30×10^{-4}
**Time varying Variables**									
MDD only	1.01	(1.004 - 1.02)	0.002	1.01	(1.004 - 1.02)	3.19×10^{-3}	1.01	(1.004 - 1.02)	3.03×10^{-3}

**Adjusted for sociodemographic factors (age, sex, Townsend score, age of leaving full time education and ethnicity. †Additionally adjusted for history of diabetes, history of hypercholesterolemia, BMI, smoking history, alcohol use, systolic blood pressure, sedentary hours per day, physical activity and psychotropic medication use. MDD = Major depressive disorder, HR = Hazard ratio, aHR = Adjusted hazard ratio, C.I. = Confidence interval.*

For peer review only

For peer review only - http://bmjopen.bmj.com/site/about/guidelines.xhtml

Table 5: Risk of stroke event by clinical group: unadjusted, partially adjusted and fully adjusted models.

	Unadjusted			Model 1 - Sociodemographic			Model 2 - Model 1 + Health/ Lifestyle		
**Group**	HR	95% C.I.	p-value	aHR	95% C.I.	p-value	aHR	95% C.I.	p-value
No Hypertension- No MDD	1(ref)			1(ref)			1(ref)		
Hypertension only	2.55	(2.16 - 3.02)	$3.84x10^{-28}$	1.64	(1.38 - 1.96)	$3.35x10^{-8}$	1.21	(0.97 - 1.51)	0.09
MDD only	1.14	(0.86 - 1.52)	0.37	1.37	(1.02 - 1.84)	0.037	1.20	(0.89 - 1.63)	0.24
Hypertension and MDD	2.67	(2.13 - 3.34)	$9.79x10^{-18}$	2.05	(1.63 - 2.58)	$1.08x10^{-9}$	1.37	(1.04 - 1.79)	0.02

**Adjusted for sociodemographic factors (age, sex, Townsend score, age of leaving full time education and ethnicity. †Additionally adjusted for history of diabetes, history of hypercholesterolemia, BMI, smoking history, alcohol use, systolic blood pressure, sedentary hours per day, physical activity and psychotropic medication use. MDD = Major depressive disorder, HR = Hazard ratio, aHR = Adjusted hazard ratio, C.I. = Confidence interval.*

For peer review only

Table 6: Risk of stroke event by clinical group: unadjusted, partially adjusted and fully adjusted models with hypertension as the comparator

	Unadjusted			Model 1 - Sociodemographic			Model 2 - Model 1 + Health/ Lifestyle		
**Group**	HR	95% C.I.	p-value	aHR	95% C.I.	p-value	aHR	95% C.I.	p-value
Hypertension only	1(ref)			1(ref)			1(ref)		
No Hypertension - No MDD	0.39	(0.33 - 0.46)	$3.84x10^{-28}$	0.61	(0.51 - 0.73)	$3.35x10^{-8}$	0.82	(0.66 - 1.03)	0.09
MDD only	0.45	(0.34 - 0.58)	$1.43x10^{-9}$	0.83	(0.63 - 1.1)	0.19	0.99	(0.73 - 1.35)	0.95
Hypertension and MDD	1.05	(0.86 - 1.27)	0.64	1.25	(1.03 - 1.52)	0.03	1.13	(0.92 - 1.39)	0.26

**Adjusted for sociodemographic factors (age, sex, Townsend score, age of leaving full time education and ethnicity. †Additionally adjusted for history of diabetes, history of hypercholesterolemia, BMI, smoking history, alcohol use, systolic blood pressure, sedentary hours per day, physical activity and psychotropic medication use. MDD = Major depressive disorder, HR = Hazard ratio, aHR = Adjusted hazard ratio, C.I. = Confidence interval.*

For peer review only

627 Table 7: Fully adjusted HR compared with results from competing risks analysis for cardiovascular endpoints

Group	Fully adjusted non-competing risks analysis			Fully adjusted competing risks model		
	aHR	95% C.I.	p-value	aHR	95% C.I.	p-value
No Hypertension - No MDD	1(ref)			1(ref)		
Hypertension only	1.36	(1.22- 1.52)	$2.92x10^{-8}$	1.37	(1.22-1.53)	$4 x10^{-8}$
MDD only	0.75	(0.54- 1.04)	0.08	0.76	(0.55-1.03)	0.08
Hypertension and MDD	1.66	(1.45- 1.9)	$7.48x10^{-14}$	1.67	(1.45-1.91)	$2.2 x10^{-13}$
tvc						
MDD only	1.01	(1.004- 1.02)	$3.03x10^{-3}$	1.01	(1.004-1.02)	0.003

628 *Adjusted for age, sex, Townsend score, age of leaving full time education and ethnicity, history of diabetes, history of hypercholesterolemia, BMI, smoking*
629 *history, alcohol use, systolic blood pressure, sedentary hours per day, physical activity and psychotropic medication use. MDD = Major depressive disorder,*
630 *aHR = Adjusted hazard ratio, C.I. = Confidence interval.*

For peer review only - http://bmjopen.bmj.com/site/about/guidelines.xhtml

Table 8: Fully adjusted HR compared with results from competing risks analysis for stroke endpoints

	**Fully adjusted non-competing risks analysis**			**Fully adjusted competing risks model**		
**Group**	aHR	95% C.I.	p-value	aHR	95% C.I.	p-value
No Hypertension - No MDD	1(ref)			1(ref)		
Hypertension only	1.21	(0.97- 1.51)	0.09	1.21	(0.96- 1.52)	0.1
MDD only	1.20	(0.89- 1.63)	0.24	1.20	(0.88- 1.64)	0.25
Hypertension and MDD	1.37	(1.04- 1.79)	0.02	1.36	(1.03- 1.8)	0.031

Adjusted for age, sex, Townsend score, age of leaving full time education and ethnicity, history of diabetes, history of hypercholesterolemia, BMI, smoking history, alcohol use, systolic blood pressure, sedentary hours per day, physical activity and psychotropic medication use. MDD = Major depressive disorder, aHR = Adjusted hazard ratio, C.I. = Confidence interval.

Figure 1: Adjusted survival analysis graph for adverse cardiovascular outcome showing greatest hazard for the comorbid group. MDD appears protective compared to the comparator group initially, however, shows increased hazard after 41 months. Analysis adjusted for age, sex, Townsend score, age of leaving full time education and ethnicity, history of diabetes, history of hypercholesterolemia, BMI, smoking history, alcohol use, systolic blood pressure, sedentary hours per day, physical activity and psychotropic medication use (MDD = Major Depressive disorder)

Figure 2: Adjusted survival analysis graph for stroke outcomes showing significantly increased hazard for comorbid Hypertension and MDD, with similar insignificant increased hazard trends for hypertension only and MDD only. Analysis adjusted for age, sex, Townsend score, age of leaving full time education and ethnicity, history of diabetes, history of hypercholesterolemia, BMI, smoking history, alcohol use, systolic blood pressure, sedentary hours per day, physical activity and psychotropic medication use. (MDD = Major Depressive disorder)

For peer review only

For peer review only - http://bmjopen.bmj.com/site/about/guidelines.xhtml

Figure 3: Log (-log) plot showing non-proportionality of MDD only survival over time. Paths between the comparator group and the MDD group cross at the 22.5 month mark. Analysis adjusted for age, sex, Townsend score, age of leaving full time education and ethnicity, history of diabetes, history of hypercholesterolemia, BMI, smoking history, alcohol use, systolic blood pressure, sedentary hours per day, physical activity and psychotropic medication use. (MDD = Major Depressive disorder)

For peer review only

For peer review only - http://bmjopen.bmj.com/site/about/guidelines.xhtml

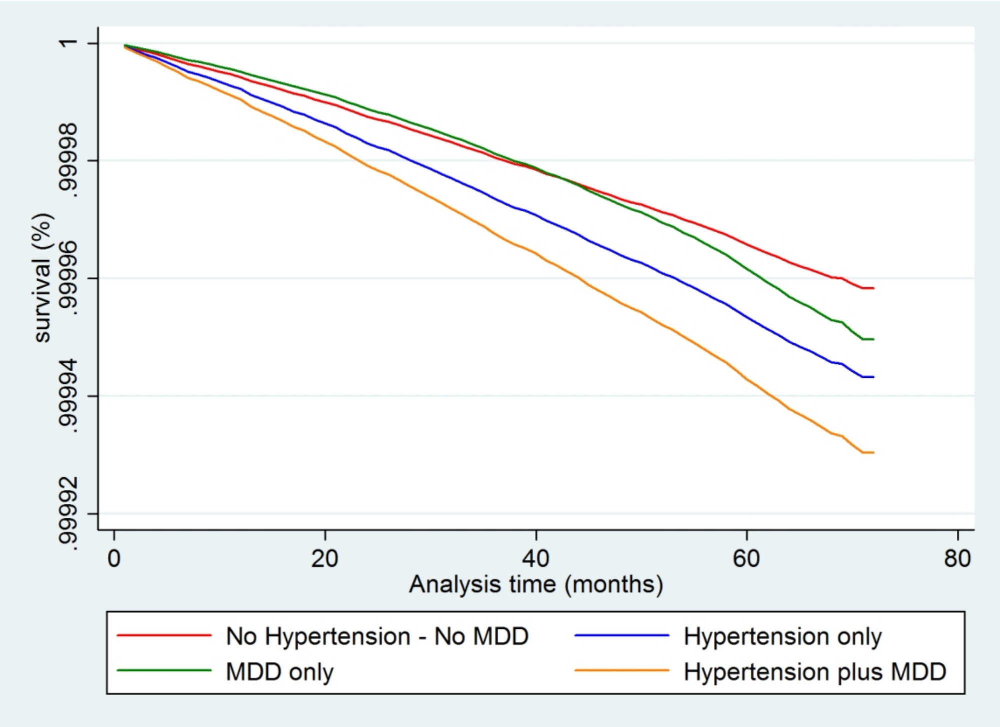

Adjusted survival analysis graph for adverse cardiovascular outcome showing greatest hazard for the comorbid group. MDD appears protective compared to the comparator group initially, however, shows increased hazard after 41 months. Analysis adjusted for age, sex, Townsend score, age of leaving full time education and ethnicity, history of diabetes, history of hypercholesterolemia, BMI, smoking history, alcohol use, systolic blood pressure, sedentary hours per day, physical activity and psychotropic medication use (MDD = Major Depressive disorder)

152x110mm (300 x 300 DPI)

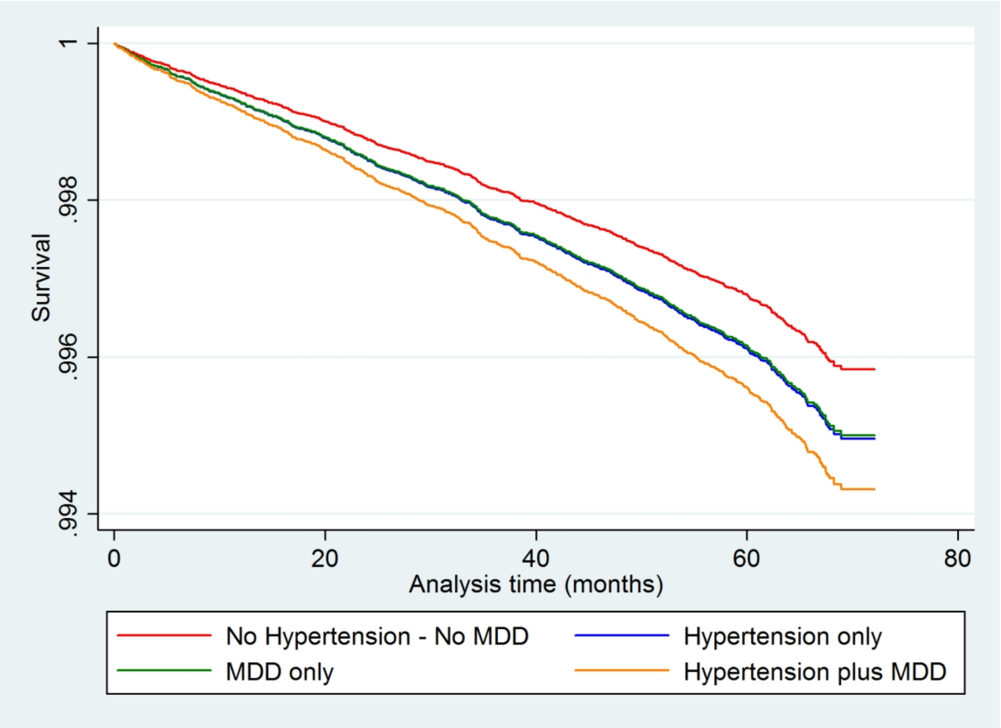

Adjusted survival analysis graph for stroke outcomes showing significantly increased hazard for comorbid Hypertension and MDD, with similar insignificant increased hazard trends for hypertension only and MDD only. Analysis adjusted for age, sex, Townsend score, age of leaving full time education and ethnicity, history of diabetes, history of hypercholesterolemia, BMI, smoking history, alcohol use, systolic blood pressure, sedentary hours per day, physical activity and psychotropic medication use. (MDD = Major Depressive disorder)

152x110mm (300 x 300 DPI)

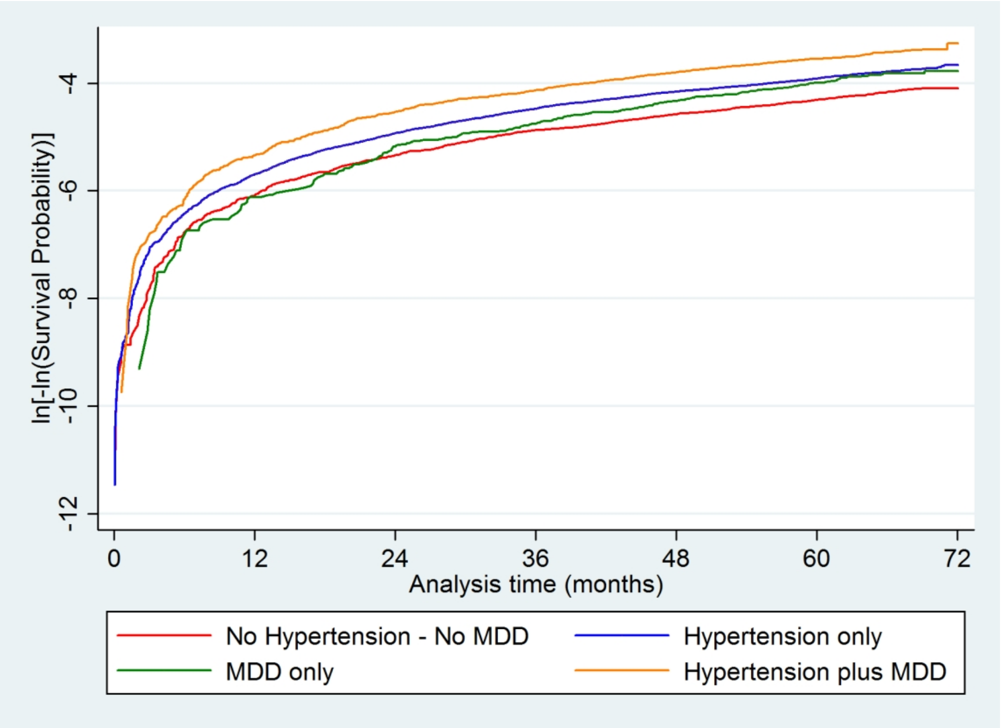

Log (-log) plot showing non-proportionality of MDD only survival over time. Paths between the comparator group and the MDD group cross at the 22.5 month mark. Analysis adjusted for age, sex, Townsend score, age of leaving full time education and ethnicity, history of diabetes, history of hypercholesterolemia, BMI, smoking history, alcohol use, systolic blood pressure, sedentary hours per day, physical activity and psychotropic medication use. (MDD = Major Depressive disorder)

152x110mm (300 x 300 DPI)

Supplementary information for Impact of major depression on cardiovascular outcomes for individuals with hypertension: prospective study in UK Biobank. Graham et al

METHODS

New-onset cardiovascular outcomes

Date and cause of death were obtained from death certificates held by the National Health Service (NHS) Information Centre for participants from England and Wales and the NHS Central Register Scotland for participants from Scotland. Date and cause of hospital admissions were identified via record linkage to Health Episode Statistics (HES) records for England, the Patient Episode Database for Wales (PEDW) and to the Scottish Morbidity Records (SMR) for Scotland. Detailed information about the record linkage procedure is available online [1 2]. At the time of analysis, mortality data were available up to 31st January 2016 for England and Wales and 11th November 2015 for Scotland. Hospital admission data were available for the Scottish, English and Welsh participants until the 31st August 2014, 31st March 2015, and 28th February 2015 respectively. Therefore, for new cardiovascular events, end of follow up was classified as the hospital admission dates unless preceded by the date of death or the date of first cardiovascular event. New onset cardiovascular events were defined as an ICD 10 code of G45, G46, I20- I25, or I6 recorded on a death certificate or hospital admission. Deaths that predated the assessment date were excluded from analysis as presumed errors as were those in which data had only recorded a death date but no cause of death or a cause of death but no death date. Participants that had hospital admissions prior to the assessment date due to the aforementioned ICD10 codes were excluded as were not first episode. In addition, ICD-9 codes 430-438, 410-414, 429 and

For peer review only - http://bmjopen.bmj.com/site/about/guidelines.xhtml

429.2 were also excluded. hospital records are not available for the entire lifetime of study individuals, potentially missing some early cardiovascular events, as such those with self-declared prior cardiovascular disease at baseline were also excluded.

Blood Pressure

Blood pressure was measured in a sitting position partway through the interview and at the end of the interview using a digital blood pressure monitor (Omron HEM-7015IT.). Full protocol is available online https://biobank.ctsu.ox.ac.uk/crystal/docs/Bloodpressure.pdf

Depression definition

The criteria for lifetime MDD were created via the the following questions via touchscreen questionnaire were: *"Looking back over your life, have you ever had a time when you were feeling depressed or down for at least a whole week?"* (depression)*; "Have you ever had a period of time lasting at least two days when you were so irritable that you found yourself shouting at people or starting fights or arguments?"* (irritability); *"How many weeks was the longest period when you were feeling depressed or down?"* (duration); *"Have you ever seen a general practitioner (GP) for nerves, anxiety, tension or depression?"* (consulted GP); *"Have you ever seen a psychiatrist for nerves, anxiety, tension or depression?"* (consulted psychiatrist). Participants were classified as having a history of MDD if they reported at least one episode which comprised of depression and/or irritability, with a duration of at least two weeks, plus had consulted with either a general practitioner or psychiatrist for mental ill-health.

Physical activity

Physical activity was based on self-report, utilising the short form International Physical Activity Questionnaire (IPAQ). Participants reported the frequency and duration of

For peer review only - http://bmjopen.bmj.com/site/about/guidelines.xhtml

moderate and vigorous activity along with walking undertaken in a typical week [3]. Data were analysed in accordance with the IPAQ scoring protocol [4] and total physical activity was computed as the sum of walking, moderate and vigorous activity, measured as metabolic equivalents (MET-hours/week). Physical activity was used in analyses as a continuous variable. Participants who reported greater than 24 hours a day doing all activity were classified as missing.

Sedentary behaviour

Sedentary behaviour duration was derived from the sum of self-reported time spent driving, using computer and watching television. Those stating that they had performed “less than an hour” of sedentary activities were coded as 0.5hrs to allow use of a continuous variable. Participants who reported greater than 24 hours a day doing all activity were classified as missing.

Socio-demographic and other covariates

Self-report on taking antihypertensive medication was taken from a question specific to cardiovascular medications, where antihypertensive medication was an option to respond. Area-based socioeconomic status was derived from postcode of residence, utilising the census-derived Townsend deprivation index scored on housing, employment, social class and car availability where a negative score represents greater affluence [5 6]. Age was calculated from dates of birth and baseline assessment date. Smoking status was categorised into never, former and current smoking based on self-report, those who wished not to answer were coded as missing. Drink frequency was categorised into daily, three or four times a week, once or twice a week, one to three times a month, special occasions only, and never based on self-report. Those who wished not to answer were coded as

For peer review only - http://bmjopen.bmj.com/site/about/guidelines.xhtml

missing. Medical history of diabetes and high cholesterol was collected from the self-completed, baseline assessment questionnaire of medical conditions. Ethnicity was categorised as Caucasian, black/mixed and Asian/mixed based on self-report. Other ethnicities coded as missing due to small numbers. Age at completing full-time education was categorised as (<16, 16, >16). Height and body weight were measured by trained nurses during the initial assessment centre visit. Body mass index (BMI) was calculated as (weight/height^2) and the WHO criteria[7] to classify BMI into: underweight <18.5, normal weight 18.5-24.9, overweight 25.0-29.9 and obese ≥30.0 kg.m^{-2}. Psychotropic medication use was defined by the presence of pharmaceuticals from British National Formulary (BNF) chapters 4.1.1 to 4.3.4 [8] on self-report medication lists at baseline. Duration of hypertension was calculated utilising age and age of hypertension diagnosis. Antihypertensive medication count was calculated as the absolute number of ACE inhibitors, angiotensin II receptor antagonists, calcium channel blockers, beta-blockers and thiazide diuretics prescribed to an individual. Generic medication names were sought and cross-referenced with the BNF chapters 2.2.1, 2.4, 2.5.5 and 2.6.2 [8].

Statistical analysis:

A best-fit multivariable regression spline model (stata command "mvrs") was used to find the best model to adjust for non-linear covariates. For the adverse cardiovascular outcomes, A single knot was fitted for age at age 50 and two knots were fitted for total physical activity at 1.65 and 8.062 metabolic equivalent hours. In the male subgroup analysis two knots were fitted for total physical activity at 1.7 and 8.507 metabolic equivalent hours, in the female subgroup two knots were fitted for total physical activity at 1.57 and 3.75 and two knots

For peer review only - http://bmjopen.bmj.com/site/about/guidelines.xhtml

were fitted at systolic blood pressure 121.5 and 147.5. No bends were noted in any models for the stroke outcomes.

Model selection and covariate adjustment

All variables were tested against outcome measures (cardiovascular outcomes and stroke outcomes) using univariate analysis to assess appropriateness for inclusion in the final model. All covariates were significantly associated with the outcomes. and were Two continuous variables, age and total physical activity, expressed non-linearity within the main analysis and male subgroup analysis for cardiovascular outcomes and as such regression splines were used with two and three knots respectively. Two knots were included within the female subgroup analysis for physical activity. For stroke outcome there were no bends in the main or sex-specific models.

Within the main analysis for cardiovascular outcomes, the groups of depression only, Asian/Asian British ethnicity and BMI<18.5 covariates failed the proportionality assumption and as such, were incorporated into the model as a time varying coefficients. Within the sex specific models depression only failed the PH test within the female only analysis and ethnicity and BMI failed within the male only analysis. For the stroke outcomes gender and BMI class failed the proportionality assumption within the main analysis, with no failures within the sex-specific analysis. Analysis was repeated with the hypertension only as the comparator group to assess for any significant difference between the co-morbid group and the hypertension only group.

Time varying covariates

Due to the finding of MDD failing the proportionality assumption in the cardiovascular outcome in the primary analysis a series of further analyses have been performed to find when the assumption was not met. A log (-log) plot (fig 3) showed the proportionality assumption was broken at 22.5 months in the fully adjusted model in the primary analysis. As such, separate models were

For peer review only - http://bmjopen.bmj.com/site/about/guidelines.xhtml

performed prior to and after these points. Prior to 22.5months the HR for MDD shows a trend that is reduced but insignificant (HR 0.82, 95%CI 0.6 - 1.13), becoming significantly increased after the 22.5 time point. (HR 1.27, 95%CI 1.06 - 1.52) (Table 9 supplementary digital content). Both stratified models passed the proportionality assumption using Schoenfeld residuals. Similar to the major analysis, the female model showed the MDD only group failing the proportionality assumption, although this was at the 29 month time point. (tables 6 and 10 of the supplementary digital content).

For peer review only

For peer review only - http://bmjopen.bmj.com/site/about/guidelines.xhtml

References

1. Palmer LJ. UK Biobank: bank on it. *Lancet* 2007;369(9578):1980-2. doi: 10.1016/S0140-6736(07)60924-6
2. Sudlow C, Gallacher J, Allen N, et al. UK Biobank: An Open Access Resource for Identifying the Causes of a Wide Range of Complex Diseases of Middle and Old Age. *Plos Medicine* 2015;12(3) doi: 10.1371/journal.pmed.1001779
3. Guo W, Bradbury KE, Reeves GK, et al. Physical activity in relation to body size and composition in women in UK Biobank. *Annals of Epidemiology* 2015;25(6):406-13.e6. doi: http://dx.doi.org/10.1016/j.annepidem.2015.01.015
4. Craig CL, Marshall AL, Sjostrom M, et al. International physical activity questionnaire: 12-country reliability and validity. *Medicine and science in sports and exercise* 2003;35(8):1381-95. doi: 10.1249/01.mss.0000078924.61453.fb [published Online First: 2003/08/06]
5. Townsend P. Deprivation. *Journal of Social Policy* 2009;16(02):125. doi: 10.1017/s0047279400020341
6. Townsend P, Phillimore M, Beattie A. Health and Deprivation: Inequality and the North. London: Croom Helm Ltd 1988.
7. WHO. Obesity: preventing and managing the global epidemic. Report of a WHO consultation. *World Health Organ Tech Rep Ser* 2000;894:i-xii, 1-253.
8. COMMITTEE. JF. British National Formulary. 67 ed. London: BMJ Group and Pharmaceutical Press 2014.

For peer review only - http://bmjopen.bmj.com/site/about/guidelines.xhtml

For peer review only

For peer review only - http://bmjopen.bmj.com/site/about/guidelines.xhtml

Supplementary Tables and figures

Supplementary Table1: Descriptive analysis for adverse cardiovascular outcome – males only

	Comparator group		Hypertension only		MDD only		Hypertension plus MDD	
	N= 21570		N = 30142		N = 4169		N = 5253	
**Median age (range)***	54	(47 - 61)	61	(54 - 65)	53	(46 - 60)	59	(52 - 64)
**Ethnicity, N (%)**								
White	19562	(90.69%)	27808	(92.26%)	3923	(94.1%)	5001	(95.2%)
Asian/Asian British	863	(4.%)	969	(3.21%)	87	(2.09%)	86	(1.64%)
Black/ Black British	559	(2.59%)	780	(2.59%)	52	(1.25%)	54	(1.03%)
**Median Townsend score (range)***	-1.87	(-3.47 - 0.59)	-2.08	(-3.53 - 0.41)	-1.58	(-3.3 - 1.07)	-1.81	(-3.44 - 0.78)
**Age at leaving full-time education, N (%)**								
<16	2517	(11.67%)	6328	(20.99%)	464	(11.13%)	1005	(19.13%)
16	4473	(20.74%)	6235	(20.69%)	859	(20.6%)	1096	(20.86%)
>16	14344	(66.5%)	17257	(57.25%)	2807	(67.33%)	3118	(59.36%)
**Total physical activity in metabolic**	4.15	(1.75 - 8.51)	3.99	(1.65 - 8.51)	4.15	(1.7 - 8.36)	3.76	(1.54 - 7.97)

For peer review only - http://bmjopen.bmj.com/site/about/guidelines.xhtml

**Sedentary time in hours, median (range)***	4.5	(3.5 - 6)	5	(3.5 - 6.5)	5	(3.5 - 6.5)	5	(4 - 7)
**Diabetes, N (%)**	721	(3.34%)	2401	(7.97%)	159	(3.81%)	477	(9.08%)
**Hypercholesterolaemia, N (%)**	1614	(7.48%)	5585	(18.53%)	363	(8.71%)	1056	(20.1%)
**Systolic BP in mmHg, median (range)***	128	(121.5 - 133.5)	149.5	(142 - 159)	127.5	(120.5 - 133)	148	(141 - 157)
**Body Mass Index, N (%)**								
<18.5	74	(0.34%)	35	(0.12%)	22	(0.53%)	12	(0.23%)
18.5 – 25	7607	(35.27%)	5842	(19.38%)	1394	(33.44%)	890	(16.94%)
25-30	10594	(49.11%)	15114	(50.14%)	2019	(48.43%)	2532	(48.2%)
>30	3295	(15.28%)	9151	(30.36%)	734	(17.61%)	1819	(34.63%)
**Smoking status, N (%)**								
Never smoked	12038	(55.81%)	15145	(50.25%)	1999	(47.95%)	2268	(43.18%)
Previously smoked	6777	(31.42%)	12125	(40.23%)	1447	(34.71%)	2295	(43.69%)
Current smoker	2688	(12.46%)	2776	(9.21%)	716	(17.17%)	686	(13.06%)
**Alcohol frequency, N (%)**								
Daily or almost daily	4822	(22.36%)	8653	(28.71%)	969	(23.24%)	1503	(28.61%)
Three or four times a week	5718	(26.51%)	7913	(26.25%)	1022	(24.51%)	1323	(25.19%)

For peer review only - http://bmjopen.bmj.com/site/about/guidelines.xhtml

Once or twice a week	5932	(27.5%)	7546	(25.03%)	1063	(25.5%)	1178	(22.43%)
One to three times a month	2193	(10.17%)	2392	(7.94%)	440	(10.55%)	479	(9.12%)
Special occasions only	1554	(7.2%)	2154	(7.15%)	328	(7.87%)	423	(8.05%)
Never	1343	(6.23%)	1473	(4.89%)	345	(8.28%)	345	(6.57%)
**Psychotropic medication, N (%)**	398	(1.85%)	670	(2.22%)	678	(16.26%)	879	(16.73%)

*All data presented as N (%) and has chi-squared p-value of <0.001 except * which are median values (interquartile range) and have a Kruskal-Wallis p-value of 0.0001. Data presented as MET-hrs (hours spent doing exercise adjusted for multiples of basal metabolic rate in accordance with IPAQ).Townsend score is an area based measure based on census statistics. It is a calculation based on the number of: households without a car, overcrowded households, households not owner-occupied and unemployment.*

For peer review only - http://bmjopen.bmj.com/site/about/guidelines.xhtml

Supplementary Table 2: Descriptive analysis for adverse cardiovascular outcome – females only

	Comparator group		Hypertension only		MDD only		Hypertension plus MDD	
	N= 29228		N = 25893		N = 10929		N = 7676	
**Median age (range)***	54	(47 - 61)	61	(55 - 65)	53	(47 - 60)	60	(53 - 64)
**Ethnicity, N (%)**								
White	26585	(90.96%)	23441	(90.53%)	10324	(94.46%)	7271	(94.72%)
Asian/Asian British	908	(3.11%)	727	(2.81%)	174	(1.59%)	93	(1.21%)
Black/ Black British	764	(2.61%)	989	(3.82%)	167	(1.53%)	168	(2.19%)
**Median Townsend score (range)***	-1.90	(-3.44 - 0.51)	-2.06	(-3.5 - 0.38)	-1.66	(-3.3 - 0.84)	-1.87	(-3.4 - 0.74)
**Age at leaving full-time education, N (%)**								
<16	3399	(11.63%)	5757	(22.23%)	1261	(11.54%)	1602	(20.87%)
16	5792	(19.82%)	5592	(21.6%)	2319	(21.22%)	1636	(21.31%)
>16	19746	(67.56%)	14223	(54.93%)	7283	(66.64%)	4385	(57.13%)
**Total physical activity in metabolic**	3.87	(1.65 - 7.71)	3.51	(1.37 - 7.59)	3.79	(1.65 - 7.91)	3.65	(1.45 - 7.93)
**Sedentary time in hours, median (range)***	4	(3 - 5)	4	(3 - 5.5)	4	(3 - 5.5)	4.5	(3 - 6)

For peer review only - http://bmjopen.bmj.com/site/about/guidelines.xhtml

**Diabetes, N (%)**	547	(1.87%)	1376	(5.31%)	221	(2.02%)	452	(5.89%)
**Hypercholesterolaemia, N (%)**	1397	(4.78%)	3625	(14.%)	530	(4.85%)	1155	(15.05%)
**Systolic BP in mmHg, median (range)***	123.5	(115.5 - 130.5)	149.5	(142 - 160)	122.5	(114.5 - 130)	147.5	(140.5 - 157)
**Body Mass Index, N (%)**								
<18.5	315	(1.08%)	107	(0.41%)	81	(0.74%)	22	(0.29%)
18.5 – 25	14942	(51.12%)	7836	(30.26%)	4857	(44.44%)	1984	(25.85%)
25-30	9816	(33.58%)	10102	(39.01%)	3917	(35.84%)	2857	(37.22%)
>30	4155	(14.22%)	7848	(30.31%)	2074	(18.98%)	2813	(36.65%)
**Smoking status, N (%)**								
Never smoked	18588	(63.6%)	16358	(63.18%)	5865	(53.66%)	4186	(54.53%)
Previously smoked	8279	(28.33%)	8015	(30.95%)	3671	(33.59%)	2770	(36.09%)
Current smoker	2282	(7.81%)	1423	(5.5%)	1377	(12.6%)	695	(9.05%)
**Alcohol frequency, N (%)**								
Daily or almost daily	4628	(15.83%)	4317	(16.67%)	1767	(16.17%)	1378	(17.95%)
Three or four times a week	6457	(22.09%)	5120	(19.77%)	2231	(20.41%)	1514	(19.72%)
Once or twice a week	7712	(26.39%)	6343	(24.5%)	2817	(25.78%)	1738	(22.64%)

For peer review only - http://bmjopen.bmj.com/site/about/guidelines.xhtml

One to three times a month	3859	(13.2%)	3196	(12.34%)	1618	(14.8%)	1033	(13.46%)
Special occasions only	3980	(13.62%)	4176	(16.13%)	1576	(14.42%)	1306	(17.01%)
Never	2581	(8.83%)	2726	(10.53%)	917	(8.39%)	703	(9.16%)
**Psychotropic medication, N (%)**	943	(3.23%)	1125	(4.34%)	2166	(19.82%)	1643	(21.4%)

*All data presented as N (%) and has chi-squared p-value of <0.001 except * which are median values (interquartile range) and have a Kruskal-Wallis p-value of 0.0001. Data presented as MET-hrs (hours spent doing exercise adjusted for multiples of basal metabolic rate in accordance with IPAQ).Townsend score is an area based measure based on census statistics. It is a calculation based on the number of: households without a car, overcrowded households, households not owner-occupied and unemployment.*

For peer review only - http://bmjopen.bmj.com/site/about/guidelines.xhtml

Supplementary Table 3: Descriptive analysis for stroke outcome – males only

	Comparator group		Hypertension only		MDD only		Hypertension plus MDD	
	N = 22816		N = 32787		N = 4438		N = 5857	
**Median age (range)***	55	(47 - 62.)	61	(54 - 65)	54	(47 - 61)	60	(53 - 64)
**Ethnicity, N (%)**								
White	20699	(90.72%)	30219	(92.17%)	4173	(94.03%)	5569	(95.08%)
Asian/Asian British	932	(4.08%)	1116	(3.4%)	102	(2.3%)	105	(1.79%)
Black/ Black British	576	(2.52%)	820	(2.5%)	53	(1.19%)	59	(1.01%)
**Median Townsend score (range)***	-1.88	(-3.47 - 0.59)	-2.05	(-3.5 - 0.46)	-1.56	(-3.28 - 1.15)	-1.74	(-3.4 - 0.93)
**Age at leaving full-time education, N (%)**								
<16	2900	(12.71%)	7256	(22.13%)	558	(12.57%)	1193	(20.37%)
16	4702	(20.61%)	6704	(20.45%)	909	(20.48%)	1222	(20.86%)
>16	14960	(65.57%)	18471	(56.34%)	2930	(66.02%)	3397	(58.%)
**Total physical activity in metabolic**	4.12	(1.74 - 8.48)	3.96	(1.65 - 8.44)	4.13	(1.67 - 8.36)	3.66	(1.45 - 7.83)
**Sedentary time in hours, median (range)***	5	(3.5 - 6)	5	(4 - 7)	5	(3.5 - 6.5)	5	(4 - 7)

For peer review only - http://bmjopen.bmj.com/site/about/guidelines.xhtml

**Diabetes, N (%)**	873	(3.83%)	2951	(9.%)	208	(4.69%)	635	(10.84%)
**Hypercholesterolaemia, N (%)**	2045	(8.96%)	6736	(20.54%)	457	(10.3%)	1293	(22.08%)
**Systolic BP in mmHg, median (range)***	128	(121.5 - 133.5)	149	(142 - 159)	127	(120.5 - 133)	147.5	(140.5 - 156.5)
**Body Mass Index, N (%)**								
<18.5	79	(0.35%)	39	(0.12%)	22	(0.5%)	12	(0.2%)
18.5 – 25	7867	(34.48%)	6215	(18.96%)	1452	(32.72%)	960	(16.39%)
25-30	11203	(49.1%)	16341	(49.84%)	2142	(48.26%)	2780	(47.46%)
>30	3667	(16.07%)	10192	(31.09%)	822	(18.52%)	2105	(35.94%)
**Smoking status, N (%)**								
Never smoked	12502	(54.79%)	16054	(48.96%)	2094	(47.18%)	2469	(42.15%)
Previously smoked	7399	(32.43%)	13603	(41.49%)	1582	(35.65%)	2610	(44.56%)
Current smoker	2836	(12.43%)	3013	(9.19%)	754	(16.99%)	770	(13.15%)
**Alcohol frequency, N (%)**								
Daily or almost daily	5085	(22.29%)	9309	(28.39%)	1021	(23.01%)	1645	(28.09%)
Three or four times a week	6039	(26.47%)	8556	(26.1%)	1077	(24.27%)	1450	(24.76%)
Once or twice a week	6264	(27.45%)	8161	(24.89%)	1121	(25.26%)	1305	(22.28%)

One to three times a month	2307	(10.11%)	2642	(8.06%)	478	(10.77%)	538	(9.19%)
Special occasions only	1666	(7.3%)	2394	(7.3%)	355	(8.%)	503	(8.59%)
Never	1444	(6.33%)	1711	(5.22%)	383	(8.63%)	414	(7.07%)
**Psychotropic medication, N (%)**	429	(1.88%)	793	(2.42%)	735	(16.56%)	1025	(17.5%)

*All data presented as N (%) and has chi-squared p-value of <0.001 except * which are median values (interquartile range) and have a Kruskal-Wallis p-value of 0.0001. Data presented as MET-hrs (hours spent doing exercise adjusted for multiples of basal metabolic rate in accordance with IPAQ).Townsend score is an area based measure based on census statistics. It is a calculation based on the number of: households without a car, overcrowded households, households not owner-occupied and unemployment.*

For peer review only

Supplementary Table 4: Descriptive analysis for stroke outcome – females only

	Comparator group		Hypertension only		MDD only		Hypertension plus MDD	
	N = 29684		N = 26937		N = 11143		N = 8090	
**Median age (range)***	54	(47 - 61)	61	(56 - 65)	53	(47 - 60)	60	(54 - 64)
**Ethnicity, N (%)**								
White	26998	(90.95%)	24359	(90.43%)	10524	(94.44%)	7643	(94.47%)
Asian/Asian British	925	(3.12%)	773	(2.87%)	178	(1.6%)	104	(1.29%)
Black/ Black British	779	(2.62%)	1034	(3.84%)	170.00	(1.53%)	187	(2.31%)
**Median Townsend score (range)***	-1.90	(-3.44 - 0.52)	-2.03	(-3.48 - 0.43)	-1.66	(-3.29 - 0.86)	-1.83	(-3.38 - 0.85)
**Age at leaving full-time education, N (%)**								
<16	3546	(11.95%)	6140	(22.79%)	1326	(11.9%)	1752	(21.66%)
16	5888	(19.84%)	5803	(21.54%)	2361	(21.19%)	1731	(21.4%)
>16	19954	(67.22%)	14643	(54.36%)	7387	(66.29%)	4550	(56.24%)
**Total physical activity in metabolic**	3.85	(1.65 - 7.7)	3.49	(1.35 - 7.57)	3.79	(1.65 - 7.89)	3.61	(1.41 - 7.87)

For peer review only - http://bmjopen.bmj.com/site/about/guidelines.xhtml

**Sedentary time in hours, median (range)***	4.0	(3 - 5)	4.0	(3 - 5.5)	4.0	(3 - 5.5)	4.5	(3 - 6)
**Diabetes, N (%)**	581	(1.96%)	1551	(5.76%)	241	(2.16%)	528	(6.53%)
**Hypercholesterolaemia, N (%)**	1547	(5.21%)	4032	(14.97%)	592	(5.31%)	1327	(16.4%)
**Systolic BP in mmHg, median (range)***	123.5	(115.5 - 131)	149.5	(142 - 160)	122.5	(114.5 - 130)	147.0	(140.5 - 157)
**Body Mass Index, N (%)**								
<18.5	316	(1.06%)	112	(0.42%)	82	(0.74%)	26	(0.32%)
18.5 – 25	15100	(50.87%)	8027	(29.8%)	4922	(44.17%)	2057	(25.43%)
25-30	9982	(33.63%)	10476	(38.89%)	4007	(35.96%)	2989	(36.95%)
>30	4286	(14.44%)	8322	(30.89%)	2132	(19.13%)	3018	(37.31%)
**Smoking status, N (%)**								
Never smoked	18816	(63.39%)	16928	(62.84%)	5958	(53.47%)	4365	(53.96%)
Previously smoked	8452	(28.47%)	8416	(31.24%)	3758	(33.73%)	2950	(36.46%)
Current smoker	2334	(7.86%)	1488	(5.52%)	1409	(12.64%)	749	(9.26%)
**Alcohol frequency, N (%)**								

Daily or almost daily	4675	(15.75%)	4442	(16.49%)	1796	(16.12%)	1440	(17.8%)
Three or four times a week	6524	(21.98%)	5271	(19.57%)	2258	(20.26%)	1570	(19.41%)
Once or twice a week	7825	(26.36%)	6558	(24.35%)	2872	(25.77%)	1820	(22.5%)
One to three times a month	3913	(13.18%)	3329	(12.36%)	1644	(14.75%)	1089	(13.46%)
Special occasions only	4078	(13.74%)	4400	(16.33%)	1623	(14.57%)	1382	(17.08%)
Never	2658	(8.95%)	2919	(10.84%)	947	(8.5%)	785	(9.7%)
**Psychotropic medication, N (%)**	979	(3.3%)	1203	(4.47%)	2241	(20.11%)	1753	(21.67%)

*All data presented as N (%) and has chi-squared p-value of <0.001 except * which are median values (interquartile range) and have a Kruskal-Wallis p-value of 0.0001. Data presented as MET-hrs (hours spent doing exercise adjusted for multiples of basal metabolic rate in accordance with IPAQ).Townsend score is an area based measure based on census statistics. It is a calculation based on the number of: households without a car, overcrowded households, households not owner-occupied and unemployment.*

For peer review only - http://bmjopen.bmj.com/site/about/guidelines.xhtml

Supplementary Table 5: Risk of adverse cardiovascular event by clinical group, in males only.

	Model one (unadjusted)			Model two (partially adjusted)*			Model three (fully adjusted) †		
**Group**	HR	95% C.I.	p-value	aHR	95% C.I.	p-value	aHR	95% C.I.	p-value
No Hypertension- No MDD	1(ref)			1(ref)			1(ref)		
Hypertension only	2.21	(2.00-2.45)	$2.28x10^{-53}$	1.62	(1.46-1.83)	$5.80x10^{-19}$	1.29	(1.13-1.47)	$1.35x10^{-4}$
MDD only	1.17	(0.95-1.56)	0.12	1.18	(0.95-1.46)	0.12	1.12	(0.9-1.39)	0.3
Hypertension and MDD	2.46	(2.13-2.84)	**3**$.12x10^{-34}$	1.95	(1.68-2.27)	$2.81x10^{-18}$	1.47	(1.24-1.74)	$8.71x10^{-6}$

**Adjusted for sociodemographic factors (age, Townsend score, age of leaving full time education and ethnicity. †Additionally adjusted for history of diabetes, history of hypercholesterolemia, BMI, smoking history, alcohol use, systolic blood pressure, sedentary hours per day, physical activity and psychotropic medication use. MDD = Major depressive disorder, HR = Hazard ratio, aHR = Adjusted hazard ratio, C.I.= Confidence interval*

For peer review only

For peer review only - http://bmjopen.bmj.com/site/about/guidelines.xhtml

Supplementary Table 6: Risk of adverse cardiovascular event by clinical group, in females only.

	Model one (unadjusted)			Model two (partially adjusted)*			Model three (fully adjusted) †		
**Group**	HR	95% C.I.	p-value	aHR	95% C.I.	p-value	aHR	95% C.I.	p-value
No Hypertension - No MDD	1(ref)			1(ref)			1(ref)		
Hypertension only	2.75	(2.38 - 3.18)	6.16×10^{-43}	1.86	(1.6-2.17)	1.43×10^{-15}	1.64	(1.33-2.02)	4.36×10^{-6}
MDD only	0.67	(0.42-1.08)	0.10	0.72	(0.45-1.17)	0.19	0.68	(0.42-1.1)	0.12
Hypertension and MDD	3.68	(3.1-4.38)	5.62×10^{-49}	2.78	(1.58-3.29)	4.62×10^{-29}	2.18	(1.82-2.92)	4.76×10^{-11}
**Time varying Variables**									
MDD only	1.02	(1.006-1.03)	2.45×10^{-3}	1.02	(1.005-1.03)	4.00×10^{-3}	1.02	(1.004-1.03)	6.19×10^{-3}

**Adjusted for sociodemographic factors (age, Townsend score, age of leaving full time education and ethnicity. †Additionally adjusted for history of diabetes, history of hypercholesterolemia, BMI, smoking history, alcohol use, systolic blood pressure, sedentary hours per day, physical activity and psychotropic medication use. MDD = Major depressive disorder, HR = Hazard ratio, aHR = Adjusted hazard ratio, C.I.= Confidence interval*

For peer review only - http://bmjopen.bmj.com/site/about/guidelines.xhtml

Supplementary Table 7: Risk of stroke event by clinical group, in males only.

	Model one (unadjusted)			Model two (partially adjusted)*			Model three (fully adjusted) †		
**Group**	HR	95% C.I.	p-value	aHR	95% C.I.	p-value	aHR	95% C.I.	p-value
No Hypertension - No MDD	1(ref)			1(ref)			1(ref)		
Hypertension only	2.43	(1.95 - 3.03)	1.92×10^{-15}	1.74	(1.38 - 2.19)	2.58×10^{-6}	1.19	(0.9 - 1.58)	0.22
MDD only	1.45	(0.96 - 2.2)	0.07	1.65	(1.09 - 2.5)	0.02	1.49	(0.97 - 2.29)	0.07
Hypertension and MDD	2.39	(1.74 - 3.27)	7.34×10^{-8}	1.87	(1.35 - 2.6)	1.55×10^{-4}	1.20	(0.83 - 1.74)	0.33

**Adjusted for sociodemographic factors (age, sex, Townsend score, age of leaving full time education and ethnicity. †Additionally adjusted for history of diabetes, history of hypercholesterolemia, BMI, smoking history, alcohol use, systolic blood pressure, sedentary hours per day, physical activity and psychotropic medication use. MDD = Major depressive disorder, HR = Hazard ratio, aHR = Adjusted hazard ratio, C.I.= Confidence interval*

For peer review only - http://bmjopen.bmj.com/site/about/guidelines.xhtml

Supplementary Table 8: Risk of stroke event by clinical group, in females only.

	Model one (unadjusted)			Model two (partially adjusted)*			Model three (fully adjusted) †		
**Group**	HR	95% C.I.	p-value	aHR	95% C.I.	p-value	aHR	95% C.I.	p-value
No Hypertension - No MDD	1(ref)			1(ref)			1(ref)		
Hypertension only	2.38	(1.84 - 3.09)	6.50×10^{-11}	1.51	(1.14 - 1.99)	3.63×10^{-3}	1.25	(0.88 - 1.79)	0.21
MDD only	1.09	(0.73 - 1.62)	0.67	1.15	(0.76 - 1.75)	0.51	0.99	(0.64 - 1.53)	0.98
Hypertension and MDD	3.05	(2.22 - 4.21)	8.71×10^{-12}	2.22	(1.59 - 3.08)	2.27×10^{-6}	1.62	(1.08 - 2.42)	0.02

**Adjusted for sociodemographic factors (age, sex, Townsend score, age of leaving full time education and ethnicity. †Additionally adjusted for history of diabetes, history of hypercholesterolemia, BMI, smoking history, alcohol use, systolic blood pressure, sedentary hours per day, physical activity and psychotropic medication use. MDD = Major depressive disorder, HR = Hazard ratio, aHR = Adjusted hazard ratio, C.I.= Confidence interval*

For peer review only - http://bmjopen.bmj.com/site/about/guidelines.xhtml

Supplementary Table 9: Time stratified analysis by moment of proportional hazards failure for adverse cardiovascular outcomes (stratified at 22.5 months)

	Fully adjusted* model pre-22.5 months			Fully adjusted* model post-22.5 months		
**Group**	aHR	95% C.I.	p-value	aHR	95% C.I.	p-value
No Hypertension - No MDD	1(ref)			1(ref)		
Hypertension only	1.36	(1.12 - 1.66)	0.002	1.36	(1.19 -1.55)	$5.06x10^{-6}$
MDD only	0.82	(0.60 - 1.13)	0.22	1.27	(1.06 -1.52)	0.01
Hypertension and MDD	1.75	(1.39 - 2.21)	$2.62x10^{-6}$	1.62	(1.38 - 1.90)	$5.72x10^{-9}$

**Adjusted for sociodemographic factors (age, sex, Townsend score, age of leaving full time education and ethnicity, history of diabetes, history of hypercholesterolemia, BMI, smoking history, alcohol use, systolic blood pressure, sedentary hours per day, physical activity and psychotropic medication use.*

MDD = Major depressive disorder, aHR =adjusted hazard ratio, C.I.= Confidence interval

For peer review only - http://bmjopen.bmj.com/site/about/guidelines.xhtml

Supplementary Table 10: Time stratified analysis by moment of proportional hazards failure for adverse cardiovascular outcomes (females only - stratified at 29 months)

	Fully adjusted* model pre-29 months			Fully adjusted* model post-29 months		
**Group**	HR	95% C.I.	p-value	HR	95% C.I.	p-value
No Hypertension - No MDD	1(ref)			1(ref)		
Hypertension only	1.49	(1.06 - 2.08)	0.02	1.75	(1.33 - 2.30)	$5.56x10^{-5}$
MDD only	0.73	(0.48 - 1.10)	0.13	1.58	(1.19 - 2.09)	0.002
Hypertension and MDD	1.80	(1.24 - 2.62)	0.002	2.47	(1.83 - 3.33)	$2.89x10^{-9}$

**Adjusted for sociodemographic factors (age, sex, Townsend score, age of leaving full time education and ethnicity, history of diabetes, history of hypercholesterolemia, BMI, smoking history, alcohol use, systolic blood pressure, sedentary hours per day, physical activity and psychotropic medication use.*

MDD = Major depressive disorder, aHR =adjusted hazard ratio, C.I.= Confidence interval

For peer review only - http://bmjopen.bmj.com/site/about/guidelines.xhtml

Supplementary Table 11: Relative excess risk due to interaction results on fully adjusted* models

Analysis	RERI	95% C.I.	LR test chi	p-value
Adverse cardiovascular outcome before 22.5 months	**0.563**	**(0.189 - 0.938)**	**6.38**	**0.0116**
Adverse cardiovascular outcome after 22.5 months	-0.009	(-0.293 - 0.275)	0.33	0.563
Adverse cardiovascular outcome (males only)	0.058	(-0.240 - 0.357)	0.02	0.899
Adverse cardiovascular outcome (females only)before 29 months	**0.588**	**(0.074 - 1.103)**	**4.65**	**0.031**
Adverse cardiovascular outcome (females only)after 29 months	0.142	(-0.447 - 0.732)	0.42	0.5173
Stroke outcome	-0.047	(-0.485 - 0.391)	0.12	0.7271
Stroke outcome (males only)	-0.480	(-1.195 - 0.234)	2.2	0.1376
Stroke outcome (females only)	0.372	(-0.216 - 0.959)	1.01	0.314

**Adjusted for sociodemographic factors (age, sex, Townsend score, age of leaving full time education and ethnicity, history of diabetes, history of hypercholesterolemia, BMI, smoking history, alcohol use, systolic blood pressure, sedentary hours per day, physical activity and psychotropic medication use.*

RERI = Relative excess risk due to interaction, C.I.= Confidence interval

For peer review only - http://bmjopen.bmj.com/site/about/guidelines.xhtml

Supplementary Table 12: Comparison of additional hypertension factors (medication and diagnosis duration) across groups

	No Hypertension – No MDD		Hypertension only		MDD only		Hypertension and MDD	
**Antihypertensive medication prescription, N (%)**	1,265	(2.49)	19,045	(33.99)	476	(3.04)	5,037	(37.34)
**Number of antihypertensive medications, N (range)***	1	(1-1)	1	(1-2)	1	(1-1)	1	(1- 2)
**Reported a duration of hypertension, N (%)**	*1,376*	(2.71)	*16,709*	(29.82)	678	(4.32)	4,525	(33.55)
**Duration of hypertension in years, median (range)***	6	(2-14)	8	(4-13)	6	(3-14)	8	(4 - 14)

**Median quantity of antihpertensive medications and median duration of hypertensive diagnosis presented for those on antihypertensive medications and supplied an age of hypertension diagnosis, respectively. MDD = Major Depressive disorder*

For peer review only - http://bmjopen.bmj.com/site/about/guidelines.xhtml

Reporting checklist for cohort study.

Based on the STROBE cohort guidelines.

Instructions to authors

Complete this checklist by entering the page numbers from your manuscript where readers will find each of the items listed below.

Your article may not currently address all the items on the checklist. Please modify your text to include the missing information. If you are certain that an item does not apply, please write "n/a" and provide a short explanation.

Upload your completed checklist as an extra file when you submit to a journal.

In your methods section, say that you used the STROBE cohort reporting guidelines, and cite them as:

von Elm E, Altman DG, Egger M, Pocock SJ, Gotzsche PC, Vandenbroucke JP. The Strengthening the Reporting of Observational Studies in Epidemiology (STROBE) Statement: guidelines for reporting observational studies.

For peer review only

		Reporting Item	Page Number
Title	#1a	Indicate the study's design with a commonly used term in the title or the abstract	1
Abstract	#1b	Provide in the abstract an informative and balanced summary of what was done and what was found	3
Background / rationale	#2	Explain the scientific background and rationale for the investigation being reported	5
Objectives	#3	State specific objectives, including any prespecified hypotheses	5
Study design	#4	Present key elements of study design early in the paper	5
Setting	#5	Describe the setting, locations, and relevant dates, including periods of recruitment, exposure, follow-up, and data collection	6
Eligibility criteria	#6a	Give the eligibility criteria, and the sources and methods of selection of participants. Describe methods of follow-up.	6-7

	#6b	For matched studies, give matching criteria and number of exposed and unexposed	n/a
Variables	#7	Clearly define all outcomes, exposures, predictors, potential confounders, and effect modifiers. Give diagnostic criteria, if applicable	7-8
Data sources / measurement	#8	For each variable of interest give sources of data and details of methods of assessment (measurement). Describe comparability of assessment methods if there is more than one group. Give information separately for for exposed and unexposed groups if applicable.	6-8
Bias	#9	Describe any efforts to address potential sources of bias	6
Study size	#10	Explain how the study size was arrived at	6
Quantitative variables	#11	Explain how quantitative variables were handled in the analyses. If applicable, describe which groupings were chosen, and why	See note 1
Statistical methods	#12a	Describe all statistical methods, including those used to control for confounding	8-9
	#12b	Describe any methods used to examine subgroups and interactions	See note 2
	#12c	Explain how missing data were addressed	6-7
	#12d	If applicable, explain how loss to follow-up was addressed	1
	#12e	Describe any sensitivity analyses	9
Participants	#13a	Report numbers of individuals at each stage of study—eg numbers potentially eligible, examined for eligibility, confirmed eligible, included in the study, completing follow-up, and analysed. Give information separately for for exposed and unexposed groups if applicable.	10
	#13b	Give reasons for non-participation at each stage	6,7
	#13c	Consider use of a flow diagram	n/a
Descriptive data	#14a	Give characteristics of study participants (eg demographic, clinical, social) and information on exposures and potential	10

For peer review only

		confounders. Give information separately for exposed and unexposed groups if applicable.	
	#14b	Indicate number of participants with missing data for each variable of interest	See note 3
	#14c	Summarise follow-up time (eg, average and total amount)	10
Outcome data	#15	Report numbers of outcome events or summary measures over time. Give information separately for exposed and unexposed groups if applicable.	6
Main results	#16a	Give unadjusted estimates and, if applicable, confounder-adjusted estimates and their precision (eg, 95% confidence interval). Make clear which confounders were adjusted for and why they were included	11-12
	#16b	Report category boundaries when continuous variables were categorized	n/a
	#16c	If relevant, consider translating estimates of relative risk into absolute risk for a meaningful time period	n/a
Other analyses	#17	Report other analyses done—e.g., analyses of subgroups and interactions, and sensitivity analyses	See note 4
Key results	#18	Summarise key results with reference to study objectives	12
Limitations	#19	Discuss limitations of the study, taking into account sources of potential bias or imprecision. Discuss both direction and magnitude of any potential bias.	14
Interpretation	#20	Give a cautious overall interpretation considering objectives, limitations, multiplicity of analyses, results from similar studies, and other relevant evidence.	13-17
Generalisability	#21	Discuss the generalisability (external validity) of the study results	15
Funding	#22	Give the source of funding and the role of the funders for the present study and, if applicable, for the original study on which the present article is based	18

Author notes

1. 6,7,8,9, supplementary

For peer review only

2. 8-9, supplementary

3. n/a (supplementary)

4. 11-12, supplemental

The STROBE checklist is distributed under the terms of the Creative Commons Attribution License CC-BY. This checklist was completed on 25. May 2018 using http://www.goodreports.org/, a tool made by the EQUATOR Network in collaboration with Penelope.ai

For peer review only

BMJ Open

Impact of major depression on cardiovascular outcomes for individuals with hypertension: prospective survival analysis in UK Biobank.

Journal:	*BMJ Open*
Manuscript ID	bmjopen-2018-024433.R3
Article Type:	Original research
Date Submitted by the Author:	22-Aug-2019
Complete List of Authors:	Graham, Nicholas; University of Glasgow Institute of Health and Wellbeing, Gartnavel Royal Hopsital 1055 Great Western Road Glasgow, UK G12 0XH<br>Ward, Joey; University of Glasgow Institute of Health and Wellbeing<br>Mackay, Daniel; University of Glasgow Institute of Health and Wellbeing<br>Pell, J. P.; University of Glasgow Institute of Health and Wellbeing<br>Cavanagh, Jonathan; University of Glasgow Institute of Health and Wellbeing<br>Padmanabhan, Sandosh; University of Glasgow, Institute of Cardiovascular and Medical Sciences, British Heart Foundation Glasgow Cardiovascular Research Centre<br>Smith, Daniel; University of Glasgow, Institute of Health and Wellbeing
**Primary Subject Heading**:	Epidemiology
Secondary Subject Heading:	Mental health, Cardiovascular medicine
Keywords:	EPIDEMIOLOGY, mortality, cardiovascular disease, morbidity, depression, Hypertension < CARDIOLOGY

SCHOLARONE™
Manuscripts

Impact of major depression on cardiovascular outcomes for individuals with hypertension: prospective survival analysis in UK Biobank.

Short title: Outcomes of Hypertension plus Depression

Nicholas A GRAHAM*[a], Clinical Research Fellow

Joey WARD[a], Research Fellow

Daniel MACKAY[b], Reader in Public Health

Jill PELL[b], Professor of Public Health

Jonathan CAVANAGH[c], Professor of Psychiatry

Sandosh PADMANABHAN[d], Professor of Cardiovascular Genomics and Therapeutics

Daniel J. SMITH[a], Professor of Psychiatry.

Number of Supplementary files: 1

Word count of Manuscript: 3,969 (exc. Tables, references, abstract, summary and Author contribution statements)

Word count of Supplementary file: 1,455 (exc. tables)

Number of tables and figures: 18 tables (including 12 in supplementary digital content) and 3 figures

[a]Institute of Health and Wellbeing, University of Glasgow, Gartnavel Royal Hospital, 1055 Great Western Road, Glasgow G12 OXH. [b]Institute of Health and Wellbeing, University of Glasgow, Public Health, 1 Lilybank Gardens, Glasgow G12 8RZ. [c]Institute of Health and Wellbeing, Centre for Immunobiology, Sir Graeme Davies Building College of Medical, Veterinary and Life Sciences University of Glasgow. [d]Institute of Cardiovascular and Medical Sciences, British Heart Foundation Glasgow Cardiovascular Research centre, University of Glasgow, Glasgow G12 8TA.

For peer review only

For peer review only - http://bmjopen.bmj.com/site/about/guidelines.xhtml

*corresponding author: nicholas.graham@glasgow.ac.uk, phone: +44 0141 211 3918,

CONFLICTS OF INTEREST: None.

For peer review only

For peer review only - http://bmjopen.bmj.com/site/about/guidelines.xhtml

ABSTRACT

Objectives: To assess whether a history of major depressive disorder (MDD) in middle-aged individuals with hypertension influences first-onset cardiovascular disease outcomes.

Design: Prospective cohort survival analysis using Cox proportional hazards regression with a median follow-up of 63 months (702,902 person-years). Four mutually exclusive groups were compared: hypertension only (n=56,035), MDD only (n=15,098), comorbid hypertension plus MDD (n=12,929), and an unaffected (no hypertension, no MDD) comparison group (n=50,798).

Setting: UK Biobank

Participants: UK Biobank participants without cardiovascular disease aged 39–70 who completed psychiatric questions relating ICD-10 diagnostic criteria on a touchscreen questionnaire at baseline interview in 2006–2010 (n=134,860).

Primary and Secondary outcome measures: First-onset adverse cardiovascular outcomes leading to hospital admission or death (ICD-10 codes I20-I259, I60-69 and G45- G46), adjusted in a stepwise manner for sociodemographic, health and lifestyle features. Secondary analyses were performed looking specifically at stroke outcomes (ICD-10 codes I60-69 and G45- G46) and in gender-separated models.

Results: Relative to controls, adjusted hazard ratios (HRs) for adverse cardiovascular outcomes were increased for the hypertension only group (HR=1.36, 95%CI 1.22-1.52) and were higher still for the comorbid hypertension plus MDD group (HR=1.66, 95%CI 1.45-1.9). HRs for the comorbid hypertension plus MDD group were significantly raised compared to hypertension alone (HR=1.22, 95%CI 1.1-1.35). Interaction measured using relative excess risk due to interaction (RERI) and likelihood ratios (LR) were identified at baseline (RERI=0.563, 95%CI 0.189 - 0.938; LR chi 6.38, p= 0.0116) but not maintained during follow-up.

Limitations: Possible selection bias in UK Biobank and inability to assess for levels of medication adherence.

For peer review only - http://bmjopen.bmj.com/site/about/guidelines.xhtml

Conclusions: Comorbid hypertension and MDD conferred greater hazard than hypertension alone for adverse cardiovascular outcomes, although evidence of interaction between hypertension and MDD was inconsistent over time. Future cardiovascular risk prediction tools may benefit from the inclusion of questions about prior history of depressive disorders.

Word count of Abstract: 299

Key words: epidemiology, mortality, morbidity, depression; hypertension, cardiovascular disease

Article Summary

STRENGTHS AND LIMITATIONS

- Methodological advantages over previous studies, including a very large sample size, adjustment for a more comprehensive range of confounders, and the inclusion of non-fatal adverse cardiovascular events from hospital admission data and death registry data.
- Definition of prior MDD history was based on ICD-10 diagnostic criteria (rather than a score on a symptoms questionnaire) and our composite definition of hypertension incorporated past history, current medication and objective blood pressure measurements.
- Although analyses were adjusted for a broad range of baseline factors (such as smoking status, BMI, psychotropic medication use and diabetes), we were unable to account for how these factors may have changed over the course of follow-up, or assess adherence to cardiovascular medications.
- Trained nurses interviewed UK Biobank participants, but the self-report nature of some of these data may represent a limitation.
- UK Biobank may have issues with respect to selection biases. For example, individuals with more severe MDD may have been less likely to volunteer.

For peer review only

INTRODUCTION

By 2030 major depressive disorder (MDD) and cardiovascular disease (CVD) will be the two leading causes of disability worldwide[1]. MDD is associated with CVD and worse long-term outcomes[2]. To date, survival analysis in comorbid hypertension and MDD have focussed on all-cause death[3-5] cardiovascular death[5] or incorporated individuals with previous CVD[3-6], and have suggested a possible additive interaction between hypertension and MDD on survival[5 6]. MDD is well known to worsen post-cardiovascular event survival[6 7]. The contribution on survival to first-onset CVD is less clear when MDD is stratified by hypertension and no prior study has assessed comorbid MDD and hypertension on first episode CVD. Within this study we look specifically at first-onset events, irrespective of whether they lead to death or not.

Hypertension is extremely common (affecting 1 billion people worldwide)[8] and is responsible for 50% of all CVD[9]. It is commonly comorbid with MDD[10 11], with recent meta-analysis showing 27% of individuals with hypertension having MDD[12] and population-based studies showing a hypertension prevalence of 21% in those with MDD[11]. A biological link has been found by genome-wide association studies, showing calcium-channel genes, important in blood pressure (BP) control and hypertension[13], also act to increase risk for MDD[14 15] and bipolar disorder (BD)[16 17]. The sympathetic nervous system (SNS), Renin-angiotensin system, the immune system and the cortisol stress response system are all also implicated in both conditions[18]. Medication management of both conditions are also thought to impact one another with side effects of psychotropic medications including raised BP and vice versa[19-21], although there is contrary evidence suggesting either medication or MDD may in actual fact be protective of hypertension[20 22].

Here we make use of prospective data from the UK Biobank cohort[23] to test the hypothesis that a lifetime history of MDD in individuals with hypertension impacts adversely on first-episode cardiovascular events. We also assess whether MDD exacerbates the effects of hypertension as a risk factor for CVD.

For peer review only - http://bmjopen.bmj.com/site/about/guidelines.xhtml

METHODS

Study design

This was a population cohort study using data from UK Biobank. Four mutually exclusive groups (hypertension only, MDD only, hypertension plus MDD, and a comparison group) were compared for adverse CVD and stroke outcomes.

Sample description

UK Biobank is a large cohort of 502,655 participants recruited between April 2007 and July 2010 from 21 assessment centres located across Great Britain[23]. Participants aged 39-70 were invited to take participate if registered with the NHS and lived within a reasonable distance of an assessment centre. At baseline assessment participants completed a series of detailed assessments relating to lifestyle and medical history on touchscreen questionnaire and have a range of physical health measurements, including body mass index (BMI) and BP taken by a nurse. UK Biobank was approved by the North West NHS Multi-Centre Research Ethics Committee and all participants provided written informed consent to participate. This analysis is part of UK Biobank approved application number 7155.

During the last two years of recruitment, questions relating to mood disorder features were added to the baseline assessment schedule questionnaire. From the 172,729 participants asked these questions, 134,860 provided sufficient responses to be included in our analysis. Participants were excluded based on the following *a priori* criteria: a history of BD (n=1,831) or schizophrenia (n=262); where there were insufficient data provided by participants to clearly rule out MDD (n= 25,520) or hypertension (n=1,080); and where there were coding errors for date and/or time of death (n=4). These exclusions were based on self-report (individuals who listed schizophrenia or BD from a list of pre-existing medical conditions), or criteria for BD as per Smith et al,[24] or where they responded “don’t know” or “prefer not to answer” to questions or data was missing that would limit our ability

For peer review only

to exclude the presence of hypertension or MDD. Participants were further excluded from the adverse CVD outcome if they had a record of CVD prior to recruitment (self-reported angina, myocardial infarction (MI) or stroke based on specific questions, or previous hospital admission for angina, MI or stroke) (n= 9,172). For the stroke outcome this exclusion was limited to a record of stroke prior to baseline assessment (self-report or previous hospital admission for stroke) (n=2,280).

Classification of hypertension and MDD

Participants were defined as having hypertension if either: *a)* mean BP at baseline was greater than clinically-defined criteria over two measurements (systolic BP greater than or equal to 140 mmHg or diastolic BP greater than or equal to 90 mmHg. Where only one reading was available this was used (n=1,571)); or *b)* self-reported 'hypertension diagnosed by a doctor' plus self-report of currently taking antihypertensive medication. This composite classification was used to ensure that undiagnosed hypertensive participants were not misclassified and is in line with similar epidemiological studies[5 25 26]. The requirement for antihypertensive use in the context of a history of hypertension was incorporated to limit those on beta-blockers for anxiety. According to these criteria, n=68,964 participants (51.1% of the sample) had hypertension for the adverse cardiovascular outcomes analysis and n=73,671 participants (52% of the sample) had hypertension in the stroke outcome analysis.

A history of lifetime MDD was defined according to the criteria for mood disorders developed by Smith et al[24 27] and has been used in further papers[27-31] (n=28,027 adverse cardiovascular outcomes; n=29,528 stroke outcomes). Participants were classified as having a history of MDD if they reported at least one episode, which comprised of depression and/or irritability, with a duration of at least two weeks, plus had consulted with either a general practitioner or psychiatrist for mental ill-health. This classification followed the structured diagnostic approach within the International Classification of Diseases[24] and is described in more detail within the supplementary content.

For peer review only - http://bmjopen.bmj.com/site/about/guidelines.xhtml

For the adverse cardiovascular outcomes, the remainder of the sample, with no history of hypertension or MDD (n=50,798) were classified as a comparator group. The three mutually exclusive diagnostic groups for this study were therefore: hypertension only (n=56,035); MDD only (n=15,098) and hypertension plus MDD (n= 12,929). For the stroke outcomes, the mutually exclusive groups were hypertension only (n=59,724); MDD only (n=15,581) and hypertension plus MDD (n= 13,947) and no hypertension – no MDD (n=52,502).

Outcomes

The primary outcome was defined as a first-episode cardiovascular event leading to hospital admission or death, specifically angina, MI, or chronic ischaemic heart disease (ICD-10 codes I20-I259), and transient ischaemic attack (TIA) or stroke (ICD-10 codes I60-69 and G45- G46). A secondary outcome was defined as stroke leading to hospital admission or death (ICD-10 codes I60-69 and G45- G46)[32] due to the strength of relationship hypertension has with this outcome in particular[9]. Admission data were obtained from Hospital Episode Statistics in England, Patient Episode Database for Wales and Scottish Morbidity Records in Scotland. Mortality outcomes were obtained from the National Health Service (NHS) Information Centre for England and Wales and from the NHS Central Register for Scotland. Individuals who died from a non-cardiovascular cause/stroke were censored at the time of death but not recorded as having an event. Admission data were available for Scottish, English and Welsh participants until 31 August 2014, 31 March 2015 and 28 February 2015 respectively. End of follow-up was classified as these dates unless preceded by date of death or the date of first cardiovascular admission.

For peer review only

Confounding variables

Information on potential confounding factors was available for age, sex, socioeconomic status (Townsend score)[33], self-reported ethnicity, age of leaving full-time education, diabetes, body mass index (BMI), systolic BP, hypercholesterolemia, alcohol use, smoking history, sedentary behaviour

For peer review only - http://bmjopen.bmj.com/site/about/guidelines.xhtml

(number of hours each day spent sitting at a computer, television or driving), physical activity levels[34] and psychotropic medication use. Specific details on these variables are provided in supplementary content.

Analyses

Baseline characteristics were compared between groups using Chi-squared tests for categorical variables and Kruskal Wallis for continuous variables. Confounding variables were assessed for differences in adverse cardiovascular outcomes using log rank sums. For the four groups of interest we assessed associations with adverse cardiovascular outcomes using Cox proportional hazard regression and the Efron method for ties[35]. Models were applied in a staged process in line with previous studies[3-5] and reported as unadjusted (model one), partially adjusted (model two) and fully adjusted (model three). Model two adjusted for sociodemographic factors (age, sex, Townsend score, age of leaving full time education and ethnicity) and model three additionally adjusted for health and lifestyle factors (diabetes, hypercholesterolemia, BMI, smoking history, alcohol use, systolic BP, sedentary hours per day, physical activity and psychotropic medication use). The proportionality of hazard assumption was assessed using Schoenfeld residuals[36]. We compared our fully adjusted models with results from competing risk analyses using the Fine and Grey approach[37], incorporating non-cardiovascular deaths as a competing event for cardiovascular events, and non-stroke deaths for stroke events. The relative excess risk due to interaction (RERI)[38] was calculated to assess for additivity in the risk. All analyses were performed with Stata statistical software, version 12[39] with the exception of RERI which was calculated using the Microsoft Excel method of Andersson and colleagues, which allows for comparison of adjusted outcomes[40]. Presence of multiplicative interaction was calculated using the likelihood ratio test.[41]

Psychotropic medication use was included as a confounding variable because of reports that they may increase risk of mortality[42] but we also conducted a sensitivity analysis which excluded participants who were taking psychotropic medication. Sub-group analyses looking separately at

For peer review only

For peer review only - http://bmjopen.bmj.com/site/about/guidelines.xhtml

196 hazard ratios (HR) in male and female groups only was also carried out to assess for any gender
197 specific differences in light of differing rates of depression and adverse cardiovascular events in each
198 gender[43 24].

199 ***Time-varying coefficients.***

200 In the context of Schoenfeld residuals showing non-proportionality, models with time varying
201 coefficients were used. In addition, log(-log) plots were carried out to find the time point at which
202 the proportionality assumption failed. Following this, the data was stratified by time at this time
203 point, effectively creating two separate survival analyses pre and post the failure time point.

204 **Patient involvement**

205 Although patients were not directly involved with the design of the specific research questions in
206 this study, the hypotheses tested were developed in the context of clinical experience that
207 depression and hypertension may interact to impact on CVD. UK Biobank has an active and ongoing
208 programme of participant involvement: www.ukbiobank.ac.uk/participants/. The outcome
209 measures used were those provided by the UK Biobank data collection protocol, the design of which
210 had input from participants. UK Biobank also has a website and social media streams to disseminate
211 research findings and hosts an annual scientific meeting, which includes cohort participants.

212 **RESULTS**

213 The final sample for adverse cardiovascular outcome included 134,860 participants followed for a
214 median duration of 63 months (702,901.6 person-years follow-up, mean 62.5 months). In total
215 3,685 (2.73%) participants had a first-episode cardiovascular event during the follow-up period (total
216 number of all deaths plus non-fatal cardiovascular events = 5,788) and 910 (0.64%) participants had
217 a first-episode stroke event (total number of all deaths plus non-fatal stroke events = 7,317).

For peer review only - http://bmjopen.bmj.com/site/about/guidelines.xhtml

For peer review only

Table 1 describes the baseline characteristics of the four groups. In general, the hypertension only and comorbid hypertension plus MDD groups were older, had higher BMI and were more likely to have diabetes and hypercholesterolemia. The MDD only and comorbid hypertension plus MDD groups had a higher proportion of women and were more likely to be current smokers (table 1). Gender-separated descriptive tables are shown in the supplementary content (Supplementary tables 1 and 2).

The sample for stroke-specific outcomes included 141,754 participants followed for a median duration of 63 months (735247.7 person-years follow-up, mean 62.2 months). Table 2 describes the baseline characteristics of the four groups which display similar characteristics to the adverse CVD outcome groups. Gender-separated descriptive tables are shown in the supplementary content (Supplementary tables 3 and 4).

Adverse cardiovascular outcomes

Within the main analysis and the female only subgroup analysis, MDD failed the proportional hazards assumption. Table 3 presents unadjusted and multivariate-adjusted Hazard ratios (aHR) for adverse cardiovascular outcomes. In the fully adjusted model, relative to the comparator group, the aHR for adverse cardiovascular outcomes was significantly raised for hypertension only (aHR=1. 36, 95%CI 1.22-1.52) and higher still for comorbid hypertension plus MDD (aHR=1.66, 95%CI 1.46-1.9) but reduced for MDD only (aHR=0.55, 95%CI 0.46-0.76). Although the MDD only HR was noted to increase over time as a time-varying coefficient. With the exception of MDD, these findings were robust to sensitivity-analysis excluding those on psychotropic medication (sensitivity analysis aHR=1.43, 95%CI 1.27-1.62; aHR=1.72, 95%CI 1.49-1.999, aHR=0.74, 95%CI 0.52-1.06 respectively). Table 4 presents HRs and aHRs for adverse cardiovascular outcomes using the hypertension only group as comparator. In the fully adjusted model, relative to hypertension, the aHR for adverse cardiovascular outcomes was significantly raised for comorbid hypertension plus MDD (aHR=1.22,

For peer review only

For peer review only - http://bmjopen.bmj.com/site/about/guidelines.xhtml

95%CI 1.1-1.35, sensitivity-analysis aHR= 1.20, 95%CI 1.08-1.34). An adjusted survival plot is shown in figure 1.

Within the sub-analysis, the male-only model showed a significant increase in HR for hypertension (male aHR 1.29, 95% CI 1.13-1.47) (supplementary table 5) and comorbid MDD and hypertension (male aHR 1.47, 95%CI 1.24-1.74). However, the difference between comorbid disease and hypertension only was not statistically significant (aHR 1.14, 95%CI 0.995-1.3). The female only sub-analysis showed an increase in HR for hypertension (aHR 1.64, 95%CI 1.33-2.02) and a greater increase in comorbid MDD and hypertension (aHR 2.18, 95%CI 1.82-2.92) (table 6 of the supplementary content). The difference between comorbid disease and hypertension only was also statistically significant (aHR 1.33, 95%CI 1.14- 1.56). Sensitivity analysis supported these findings.

Stroke Outcomes

None of the independent variables for stroke outcome failed the proportionality assumption. Table 5 presents HRs and aHRs for stroke outcomes. In the fully adjusted model, the aHR for stroke was insignificantly raised for hypertension only (aHR=1.21, 95%CI 0.97-1.51) and depression only (aHR=1.20, 95%CI 0.89-1.63) but significantly raised for comorbid hypertension plus MDD (aHR=1.37, 95%CI 1.04-1.79). In the hypertension comparator group, no group was significantly different from hypertension only (table 6). Similar trends were shown in the gender subset analysis but not reaching significance (supplementary Tables 7-8). An adjusted survival plot is shown in figure 2. Again, all results were supported by sensitivity analysis excluding those on psychotropic medication.

Interaction, time stratified analysis and competing risk analysis

Survival analysis stratified by time is described and included within the supplementary content (supplementary tables 9, 10 and figure 3). There was evidence of both additive and multiplicative interaction between hypertension and MDD at baseline for the overall cardiovascular outcome analysis before the 22.5 month time point (additive: RERI=0.563, 95%CI 0.189 - 0.938. Multiplicative:

For peer review only - http://bmjopen.bmj.com/site/about/guidelines.xhtml

Likelihood ratio p-value 0.0116) and the female only cardiovascular endpoint analysis before the 29 month time point (additive: RERI=0.588, 95%CI 0.074 - 1.103. Multiplicative: Likelihood ratio p-value 0.031). However, after these time points there was no evidence of interaction on either the additive or multiplicative scale. Supplementary table 11 shows the full results for this analysis. Competing risk analysis showed no significant difference from the main analyses for cardiovascular outcomes or stroke outcomes (tables 7-8)

DISCUSSION

In this large population cohort of middle-aged adults without CVD (adjusted for a broad range of confounders), individuals with co-morbid hypertension and MDD were at increased risk of CVD when compared to those with hypertension alone, MDD alone and neither condition. There was some evidence of additive and multiplicative interaction between hypertension and MDD at baseline, but not throughout follow-up and only within the female subgroup. Such a finding may suggest a causal interaction between MDD and hypertension in females only, but suggests that this may be limited over time leading to a suspected further interaction with a gender specific unmeasured confounder. Differences between co-morbid disease and either disease alone or no disease were more marked in females. For stroke outcomes, comorbid depression and hypertension was the only group that showed significantly increased HRs.

Previous research

Our findings expand upon previous research from UK Biobank looking at CVD in those with BD and MDD[27]. It was found that there were significantly increased odds of having 'any CVD' (fully adjusted OR 1.15 CI 1.12–1.19) or hypertension (fully adjusted OR 1.15 CI 1.13–1.18) if depressed, with even higher odds for stroke (fully adjusted OR 1.26 CI 1.13–1.40). There are distinct differences between our current paper and the previous publication. Follow-up data within UK-Biobank has been released

For peer review only - http://bmjopen.bmj.com/site/about/guidelines.xhtml

to allow meaningful prospective studies be conducted. Thus, the current paper has the benefits of using hospital records and death certification for outcomes, rather than self-reported data. We are also able to make inferences about the direction of effect regarding MDD and CVD and assess the influence of hypertension and MDD over time, both in isolation and when comorbid, and assess for statistical interaction to inform on whether there may be a biological interaction.

Other survival analyses in hypertension/MDD comorbidity have focussed primarily on mortality outcomes. In the National Health and Nutrition Epidemiologic Follow-up Study in the United States[31] and the Taiwanese Survey of Health and Living Status[32], individuals with self-reported hypertension plus depressive symptoms (compared to a reference group with neither) had increased all-cause mortality (aHR=1.39, 95%CI 1.14-1.69, aHR=1.54, 95%CI 1.29-1.83, respectively) [3 4] with the former also showing increased CVD specific mortality (aHR=1.59, 95%CI 1.08-2.34)[4]. Similarly, Hamer and colleagues[5] reported a prospective analysis of common mental disorder on mortality outcomes in individuals with hypertension versus those without hypertension in participants from the Health Survey for England and the Scottish Health Survey (1994–2004), finding that risk of CVD death was highest in the group with comorbid disease.

Strengths

These observations are broadly consistent with our results but our study has a number of methodological advantages, including a very large sample size, adjustment of analyses for a more comprehensive range of confounders, and a focus on first-episode non-fatal and fatal adverse cardiovascular events. We also used a definition of prior MDD history which was based on diagnostic criteria within ICD-10 (rather than a threshold score on a depressive symptoms or general wellbeing scale) and our composite definition of hypertension incorporated past history, baseline medication and BP measurements. Lifetime MDD is thought to be under-reported in the literature. However, using current symptom scores may reduce power and precision because a smaller number of respondents would be identified as having an episode of MDD.[44] Given that we are assessing

For peer review only - http://bmjopen.bmj.com/site/about/guidelines.xhtml

outcomes for which risk accumulates over a lifetime, we felt that a primary focus on lifetime episodes was appropriate. We believe our lifetime definition to be better suited as it offers a view depression and depressive symptoms over the course of a lifespan as opposed the past week. Also, within our current study we were able to exclude those with previous self-declared or hospital admission CVD, as previous studies show depression may result from CVD[45 46] and worsen prognosis[46]

Limitations

However, some limitations are acknowledged. Recruitment criteria for UK Biobank may lead to selection bias. Specifically, age-restrictions may lead to underrepresentation of early-onset hypertension and those with more severe MDD may be less inclined to attend for assessment. We also acknowledge limitations with our classifications of MDD and hypertension, which were primarily self-report rather than formal diagnostic assessments. Although we have excluded prior cardiovascular events where possible, the MDD plus hypertension sub-type may capture older individuals with a degree of vascular depression, which has an established association with raised BP[47]. In addition, although we adjust for a host of risk factors at baseline such as smoking status, BMI and psychotropic medication, we are limited by the lack of follow-up data, which could show change and modification of said risk factors over time. Similarly, we were unable to assess for medication adherence and transitions from one investigatory group to another. Participants who are aware of or had sought treatment for MDD may also have complicated our findings, however, our sensitivity analysis excluded those using pharmaceutical treatments and was in keeping with our main findings. Such modifications could explain the non-proportional nature of the depression group, which may in itself be a predictor of poor medication adherence[48]. Although adherence to medication was not formally assessed, the number and duration of antihypertensive medications used in the hypertension plus MDD group was the same as for the hypertension only group (supplementary content, table 12). As such, worse outcomes in the MDD plus hypertension group are not explained

For peer review only - http://bmjopen.bmj.com/site/about/guidelines.xhtml

by less intensive antihypertensive treatment at baseline. The end-points used for stroke and cardiovascular events also require to be further validated, however are in line with previous epidemiological studies[5] and have been suggested in previous papers in UK Biobank[32]. Cardiovascular endpoints have not, to our knowledge, been validated within UKbiobank, however we do not feel that this will bias the results towards any particular group. The amelioration of the aHR suggests other covariates contribute considerably to the risk. This is important in the context of increased rates of diabetes, hypercholesterolemia and obesity along with lower socio-economic status in the hypertension only and comorbid groups and as such we may be seeing the summation of CV risk factors. Finally, the overall recruitment rate to UK Biobank was low (at around 6%); however, the large final cohort size, the depth and diversity of phenotype data collected at baseline, and the wide sociodemographic representation of participants all make our findings highly relevant to UK primary care settings. While UK Biobank participants cannot be used to provide representative disease prevalence and incidence rates, valid assessment of exposure-disease relationships are nonetheless widely generalizable and do not require participants to be representative of the UK population at large[49], although findings will not be generalizable to other countries.

Possible mechanisms

Our finding that a history of MDD, in the context of a current diagnosis of hypertension increased the risk of first-episode CVD is complicated by the time varying risk that MDD conveys to CVD. Sub-sample analysis show this time-varying aspect is gender-specific to females. Within our sample, the MDD group has a slightly reduced BP compared to comparators. Previously, reduced BP has been put forth as being causative of MDD and therefore reducing CVD risk[20], but findings from longitudinal studies are inconsistent with regards to direction of effect[50 51]. Potential menopausal effects are tempting explanations. Common factors for BP and mood such as neuropeptide Y[52 53] may also influence cardiovascular outcomes. Neuropeptide Y has a complex relationship with oestrogen[54]

For peer review only - http://bmjopen.bmj.com/site/about/guidelines.xhtml

365 and both have dampening effect on the SNS[55]. Neuropeptide Y and oestrogen may represent a
366 biologically plausible interaction between MDD and hypertension, however, this would require
367 investigation.

368 Personality factors may also play a role. MDD correlates highly with neuroticism which, although
369 inconsistent, may be protective of CVD[56]. Conscientiousness traits may lead to better outcomes[57]
370 and it is possible that this trait has been selected for within UK Biobank. Despite this early reduced
371 risk, due to the time varying nature of MDD, MDD has increased risk in the latter aspects of the time-
372 stratified analyses for the full and female only analyses (supplementary table 9 and 10). The findings
373 from our study in this context suggest MDDs role as a risk factor for CVD and its relationship with BP
374 may be much more complex than initially thought, in particular within female populations however
375 further investigation is clearly needed.

376 We can see in the hypertension only baseline models that comorbid hypertension and depression
377 convey a significantly greater risk than hypertension alone. Individuals with either hypertension or
378 depression may have increased sympathetic stimulation that is increased further in comorbid states
379 leading to worse outcomes[58].

380

381 **CONCLUSIONS**

382 Overall, our findings may have important implications for routine clinical practice, particularly within
383 primary care settings and further demonstrate the complex relationship between depression and
384 hypertension. Although evidence of an interaction is inconsistent, we found that comorbid
385 hypertension and depression conferred greater hazard than hypertension alone for adverse
386 cardiovascular outcomes. This significant finding remained after adjustment for factors such as BMI,
387 smoking status and diabetes and was robust to sensitivity analysis excluding those on psychotropic
388 medication. One implication is that clinicians should be more aware of the negative long-term
389 impact on CVD outcomes caused by a history of MDD in the context of hypertension, particularly

For peer review only - http://bmjopen.bmj.com/site/about/guidelines.xhtml

390 within females. Although this work awaits replication and testing in other cohorts and settings,
391 further work in this field may suggest that future iterations of CVD risk prediction tools, such as
392 ASSIGN[59], would benefit from the addition of a question on whether individuals have a past history
393 of MDD, to facilitate more intensive support to prevent CVD[60].

For peer review only

For peer review only - http://bmjopen.bmj.com/site/about/guidelines.xhtml

ACKNOWLEDGEMENTS

We are grateful to all participants of the UK Biobank cohort. UK Biobank was established by the Wellcome Trust, the Medical Research Council, Department of Health, Scottish Government and the Northwest Regional Development Agency. It has also had funding from the Welsh Assembly Government and the British Heart Foundation. UK Biobank is hosted by the University of Manchester and supported by the National Health Service (NHS). NG is supported by the Aitchison Family Clinical Research Fellowship at the University of Glasgow and DJS is supported by a Lister Institute Prize Fellowship. JC is supported by the Sackler Trust and the Wellcome Trust. Part-funded by the Medical Research Council Mental Health Data Pathfinder Award (grant reference MC_PC_17217)"

Footnotes

Authors Statement: Contributors NG, JW, JP, JC, DS, SP and DM, contributed to study design and writing of the manuscript. JP and DM contributed to data acquisition. NG conducted data processing and statistical analyses.

Funding: Authors declare no support from any organisation for the submitted work;

Competing interests: All authors have completed the ICMJE uniform disclosure form at http://www.icmje.org/coi_disclosure.pdf and no financial relationships with any organisations that might have an interest in the submitted work in the previous three years; no other relationships or activities that could appear to have influenced the submitted work.

Ethics approval: This study has been conducted using UK Biobank data. UK Biobank has received ethics approval from the UK Biobank Research Ethics Committee (ref. 11/NW/0382).

Data sharing statement: The data used in this study are available via a direct application to UK Biobank.

For peer review only

For peer review only - http://bmjopen.bmj.com/site/about/guidelines.xhtml

Transparency statement: The lead author affirms that this manuscript is an honest, accurate and transparent account of the study being reported; that no important aspects of the study have been omitted; and that any discrepancies from the study as planned (and, if relevant, registered) have been explained.

COMPETING INTERESTS STATEMENTS

All authors have completed the ICMJE uniform disclosure form at http://www.icmje.org/coi_disclosure.pdf and no financial relationships with any organisations that might have an interest in the submitted work in the previous three years; no other relationships or activities that could appear to have influenced the submitted work.

For peer review only

For peer review only - http://bmjopen.bmj.com/site/about/guidelines.xhtml

References

1. Organization. WH. The global burden of disease: 2004 update. Geneva, Switzerland.: WHO press. 2008.
2. Hare DL, Toukhsati SR, Johansson P, et al. Depression and cardiovascular disease: a clinical review. *Eur Heart J* 2014;35(21):1365-72. doi: 10.1093/eurheartj/eht462
3. Kuo PL, Pu C. The contribution of depression to mortality among elderly with self-reported hypertension: analysis using a national representative longitudinal survey. *J Hypertens* 2011;29(11):2084-90. doi: 10.1097/HJH.0b013e32834b59ad [published Online First: 2011/09/22]
4. Axon RN, Zhao Y, Egede LE. Association of depressive symptoms with all-cause and ischemic heart disease mortality in adults with self-reported hypertension. *Am J Hypertens* 2010;23(1):30-7. doi: 10.1038/ajh.2009.199
5. Hamer M, Batty GD, Stamatakis E, et al. The combined influence of hypertension and common mental disorder on all-cause and cardiovascular disease mortality. *J Hypertens* 2010;28(12):2401-6. doi: 10.1097/HJH.0b013e32833e9d7c
6. Jani BD, Cavanagh J, Barry SJ, et al. Relationship Between Blood Pressure Values, Depressive Symptoms, and Cardiovascular Outcomes in Patients With Cardiometabolic Disease. *Journal of clinical hypertension (Greenwich, Conn)* 2016;18(10):1027-35. doi: 10.1111/jch.12813 [published Online First: 2016/04/05]
7. Taylor CB, Youngblood ME, Catellier D, et al. Effects of antidepressant medication on morbidity and mortality in depressed patients after myocardial infarction. *Arch Gen Psychiatry* 2005;62(7):792-8. doi: 10.1001/archpsyc.62.7.792 [published Online First: 2005/07/06]
8. Kearney PM, Whelton M, Reynolds K, et al. Global burden of hypertension: analysis of worldwide data. *Lancet* 2005;365(9455):217-23. doi: 10.1016/S0140-6736(05)17741-1
9. Lawes CM, Vander Hoorn S, Rodgers A, et al. Global burden of blood-pressure-related disease, 2001. *Lancet* 2008;371(9623):1513-8. doi: 10.1016/S0140-6736(08)60655-8
10. Meng L, Chen D, Yang Y, et al. Depression increases the risk of hypertension incidence: a meta-analysis of prospective cohort studies. *J Hypertens* 2012;30:842 - 51.
11. Wu EL, Chien IC, Lin CH, et al. Increased risk of hypertension in patients with major depressive disorder: a population-based study. *J Psychosom Res* 2012;73(3):169-74. doi: 10.1016/j.jpsychores.2012.07.002
12. Li Z, Li Y, Chen L, et al. Prevalence of Depression in Patients With Hypertension: A Systematic Review and Meta-Analysis. *Medicine (Baltimore)* 2015;94(31):e1317. doi: 10.1097/md.0000000000001317 [published Online First: 2015/08/08]
13. Johnson AD, Newton-Cheh C, Chasman DI, et al. Association of hypertension drug target genes with blood pressure and hypertension in 86,588 individuals. *Hypertension* 2011;57(5):903-10. doi: 10.1161/HYPERTENSIONAHA.110.158667
14. Casamassima F HJ, Fava M, Sachs GS, Smoller JW, Cassano GB, Lattanzi L, Fagerness J, Stange JP, Perlis RH. Phenotypic effects of a bipolar liability gene among individuals with major depressive disorder. . *Am J Med Genet B Neuropsychiatr Genet* 2010;153B:303-09.
15. Green EK, Grozeva D, Jones I, et al. The bipolar disorder risk allele at CACNA1C also confers risk of recurrent major depression and of schizophrenia. *Molecular Psychiatry* 2010;15(10):1016-22. doi: 10.1038/mp.2009.49
16. Ferreira MA, O'Donovan MC, Meng YA, et al. Collaborative genome-wide association analysis supports a role for ANK3 and CACNA1C in bipolar disorder. *Nat Genet* 2008;40(9):1056-8. doi: 10.1038/ng.209
17. Consortium WTCC. Identification of risk loci with shared effects on five major psychiatric disorders: a genome-wide analysis. *The Lancet* 2013;381(9875):1371-79.

For peer review only - http://bmjopen.bmj.com/site/about/guidelines.xhtml

18. Scalco AZ, Scalco MZ, Azul JB, et al. Hypertension and depression. *Clinics (Sao Paulo)* 2005;60(3):241-50. doi: /S1807-59322005000300010
19. Boal AH, Smith DJ, McCallum L, et al. Monotherapy With Major Antihypertensive Drug Classes and Risk of Hospital Admissions for Mood Disorders. *Hypertension* 2016;68(5):1132-38. doi: 10.1161/hypertensionaha.116.08188 [published Online First: 2016/10/14]
20. Licht CM, de Geus EJ, Seldenrijk A, et al. Depression is associated with decreased blood pressure, but antidepressant use increases the risk for hypertension. *Hypertension* 2009;53(4):631-8. doi: 10.1161/HYPERTENSIONAHA.108.126698
21. Crookes DM, Demmer RT, Keyes KM, et al. Depressive Symptoms, Antidepressant Use, and Hypertension in Young Adulthood. *Epidemiology (Cambridge, Mass)* 2018;29(4):547-55. doi: 10.1097/ede.0000000000000840 [published Online First: 2018/04/10]
22. Diminic-Lisica I, Popovic B, Rebic J, et al. Outcome of treatment with antidepressants in patients with hypertension and undetected depression. *Int J Psychiatry Med* 2014;47(2):115-29. doi: 10.2190/PM.47.2.c [published Online First: 2014/08/03]
23. Sudlow C, Gallacher J, Allen N, et al. UK biobank: an open access resource for identifying the causes of a wide range of complex diseases of middle and old age. *PLoS Med* 2015;12(3):e1001779. doi: 10.1371/journal.pmed.1001779
24. Smith DJ, Nicholl BI, Cullen B, et al. Prevalence and characteristics of probable major depression and bipolar disorder within UK biobank: cross-sectional study of 172,751 participants. *PLoS One* 2013;8(11):e75362. doi: 10.1371/journal.pone.0075362
25. Ong KL, Cheung BM, Man YB, et al. Prevalence, awareness, treatment, and control of hypertension among United States adults 1999-2004. *Hypertension* 2007;49(1):69-75. doi: 10.1161/01.hyp.0000252676.46043.18 [published Online First: 2006/12/13]
26. Egan BM, Zhao Y, Axon RN. US trends in prevalence, awareness, treatment, and control of hypertension, 1988-2008. *Jama* 2010;303(20):2043-50. doi: 10.1001/jama.2010.650 [published Online First: 2010/05/27]
27. Martin DJ, Ul-Haq Z, Nicholl BI, et al. Cardiometabolic disease and features of depression and bipolar disorder: population-based, cross-sectional study. *Br J Psychiatry* 2016;208(4):343-51. doi: 10.1192/bjp.bp.114.157784
28. Ul-Haq Z, Smith DJ, Nicholl BI, et al. Gender differences in the association between adiposity and probable major depression: a cross-sectional study of 140,564 UK Biobank participants. *BMC psychiatry* 2014;14:153-53. doi: 10.1186/1471-244X-14-153
29. Sarkar C, Webster C, Gallacher J. Residential greenness and prevalence of major depressive disorders: a cross-sectional, observational, associational study of 94 879 adult UK Biobank participants. *The Lancet Planetary Health* 2018;2(4):e162-e73. doi: https://doi.org/10.1016/S2542-5196(18)30051-2
30. Hall LS, Adams MJ, Arnau-Soler A, et al. Genome-wide meta-analyses of stratified depression in Generation Scotland and UK Biobank. *Translational psychiatry* 2018;8(1):9-9. doi: 10.1038/s41398-017-0034-1
31. Howard DM, Adams MJ, Shirali M, et al. Genome-wide association study of depression phenotypes in UK Biobank identifies variants in excitatory synaptic pathways. *Nature Communications* 2018;9(1):1470. doi: 10.1038/s41467-018-03819-3
32. Woodfield R, Grant I, Group UKBSO, et al. Accuracy of Electronic Health Record Data for Identifying Stroke Cases in Large-Scale Epidemiological Studies: A Systematic Review from the UK Biobank Stroke Outcomes Group. *PLOS ONE* 2015;10(10):e0140533. doi: 10.1371/journal.pone.0140533
33. Townsend P. Deprivation. *Journal of Social Policy* 2009;16(02):125. doi: 10.1017/s0047279400020341
34. Guo W, Bradbury KE, Reeves GK, et al. Physical activity in relation to body size and composition in women in UK Biobank. *Annals of Epidemiology* 2015;25(6):406-13.e6. doi: http://dx.doi.org/10.1016/j.annepidem.2015.01.015

For peer review only - http://bmjopen.bmj.com/site/about/guidelines.xhtml

35. Efron B. The Efficiency of Cox's Likelihood Function for Censored Data. *Journal of the American Statistical Association,* 1977;72(359):557-65.

36. Schoenfeld D. Partial Residuals for the Proportional Hazards Regression-Model. *Biometrika* 1982;69(1):239-41. doi: Doi 10.2307/2335876

37. Fine JP, Gray RJ. A Proportional Hazards Model for the Subdistribution of a Competing Risk. *Journal of the American Statistical Association* 1999;94(446):496-509. doi: 10.2307/2670170

38. Hosmer DW, Lemeshow S. Confidence interval estimation of interaction. *Epidemiology (Cambridge, Mass)* 1992;3(5):452-6. [published Online First: 1992/09/01]

39. Stata Statistical Software, version 12 [program]. College station, Texas.

40. Andersson T, Alfredsson L, Kallberg H, et al. Calculating measures of biological interaction. *European journal of epidemiology* 2005;20(7):575-9. [published Online First: 2005/08/27]

41. Marshall SW. Power for tests of interaction: effect of raising the Type I error rate. *Epidemiologic Perspectives & Innovations* 2007;4(1):4. doi: 10.1186/1742-5573-4-4

42. Weich S, Pearce HL, Croft P, et al. Effect of anxiolytic and hypnotic drug prescriptions on mortality hazards: retrospective cohort study. *BMJ* 2014;348:g1996. doi: 10.1136/bmj.g1996

43. Nichols M, Townsend N, Scarborough P, et al. Cardiovascular disease in Europe 2014: epidemiological update. *European Heart Journal* 2014;35(42):2950-59. doi: 10.1093/eurheartj/ehu299

44. Patten SB. Accumulation of major depressive episodes over time in a prospective study indicates that retrospectively assessed lifetime prevalence estimates are too low. *BMC psychiatry* 2009;9:19-19. doi: 10.1186/1471-244X-9-19

45. Kang HJ, Stewart R, Bae KY, et al. Predictive value of homocysteine for depression after acute coronary syndrome. *Oncotarget* 2016;7(42):69032-40. doi: 10.18632/oncotarget.11966

46. Lichtman JH, Froelicher ES, Blumenthal JA, et al. Depression as a risk factor for poor prognosis among patients with acute coronary syndrome: systematic review and recommendations: a scientific statement from the American Heart Association. *Circulation* 2014;129(12):1350-69. doi: 10.1161/CIR.0000000000000019

47. Aizenstein HJ, Baskys A, Boldrini M, et al. Vascular depression consensus report – a critical update. *BMC Medicine* 2016;14(1):161. doi: 10.1186/s12916-016-0720-5

48. Goldstein CM, Gathright EC, Garcia S. Relationship between depression and medication adherence in cardiovascular disease: the perfect challenge for the integrated care team. *Patient preference and adherence* 2017;11:547-59. doi: 10.2147/PPA.S127277

49. Fry A, Littlejohns TJ, Sudlow C, et al. Comparison of Sociodemographic and Health-Related Characteristics of UK Biobank Participants With Those of the General Population. *American journal of epidemiology* 2017;186(9):1026-34. doi: 10.1093/aje/kwx246 [published Online First: 2017/06/24]

50. Hildrum B, Mykletun A, Holmen J, et al. Effect of anxiety and depression on blood pressure: 11-year longitudinal population study. *British Journal of Psychiatry* 2018;193(2):108-13. doi: 10.1192/bjp.bp.107.045013 [published Online First: 01/02]

51. Paterniti S. Low blood pressure and risk of depression in the elderly: A prospective community-based study. *The British Journal of Psychiatry* 2000;176(5):464-67. doi: 10.1192/bjp.176.5.464

52. Zhang P, Qi Y-X, Yao Q-P, et al. Neuropeptide Y Stimulates Proliferation and Migration of Vascular Smooth Muscle Cells from Pregnancy Hypertensive Rats via Y1 and Y5 Receptors. *PLOS ONE* 2015;10(7):e0131124. doi: 10.1371/journal.pone.0131124

53. Morales-Medina JC, Dumont Y, Quirion R. A possible role of neuropeptide Y in depression and stress. *Brain research* 2010;1314:194-205. doi: https://doi.org/10.1016/j.brainres.2009.09.077

54. Pelletier G, Li S, Luu-The V, et al. Oestrogenic Regulation of Pro-Opiomelanocortin, Neuropeptide Y and Corticotrophin-Releasing Hormone mRNAs in Mouse Hypothalamus. *Journal of Neuroendocrinology* 2007;19(6):426-31. doi: 10.1111/j.1365-2826.2007.01548.x

For peer review only - http://bmjopen.bmj.com/site/about/guidelines.xhtml

55. Rosano GM, Panina G. Oestrogens and the heart. *Therapie* 1999;54(3):381-5. [published Online First: 1999/09/29]
56. Gale CR, Čukić I, Batty GD, et al. When Is Higher Neuroticism Protective Against Death? Findings From UK Biobank. *Psychological Science* 2017;28(9):1345-57. doi: 10.1177/0956797617709813
57. Cheng H, Montgomery S, Treglown L, et al. Emotional stability, conscientiousness, and self-reported hypertension in adulthood. *Personality and Individual Differences* 2017;115:159-63. doi: https://doi.org/10.1016/j.paid.2016.02.034
58. Barton DA, Dawood T, Lambert EA, et al. Sympathetic activity in major depressive disorder: identifying those at increased cardiac risk? *J Hypertens* 2007;25(10):2117-24. doi: 10.1097/HJH.0b013e32829baae7 [published Online First: 2007/09/22]
59. Woodward M, Brindle P, Tunstall-Pedoe H. Adding social deprivation and family history to cardiovascular risk assessment: the ASSIGN score from the Scottish Heart Health Extended Cohort (SHHEC). *Heart* 2007;93(2):172-76. doi: 10.1136/hrt.2006.108167
60. Hippisley-Cox J, Coupland C, Vinogradova Y, et al. Predicting cardiovascular risk in England and Wales: prospective derivation and validation of QRISK2. *BMJ* 2008;336(7659):1475-82. doi: 10.1136/bmj.39609.449676.25

For peer review only

For peer review only - http://bmjopen.bmj.com/site/about/guidelines.xhtml

Table 1. Baseline characteristics for adverse cardiovascular outcomes

	Comparator group	Hypertension only	MDD only	Hypertension plus MDD
	N = 50798	N = 56035	N = 15098	N = 12929
**Median age (range)***	54 (47 - 61)	61 (55 - 65)	53 (46 - 60)	60 (53 - 64)
**Females, N (%)**	29228 (57.54%)	25893 (46.21%)	10929 (72.39%)	7676 (59.37%)
**Ethnicity, N (%)**				
White	46147 (90.84%)	51249 (91.46%)	14247 (94.36%)	12272 (94.92%)
Asian/Asian British	1771 (3.49%)	1696 (3.03%)	261 (1.73%)	179 (1.38%)
Black/ Black British	1323 (2.6%)	1769 (3.16%)	219 (1.45%)	222 (1.72%)
**Median Townsend score (range)***	-1.89 (-3.45 - 0.54)	-2.07 (-3.51 - 0.39)	-1.64 (-3.3 - 0.93)	-1.84 (-3.42 - 0.76)
**Age at leaving full-time education, N (%)**				
<16	5916 (11.65%)	12085 (21.57%)	1725 (11.43%)	2607 (20.16%)
16	10265 (20.21%)	11827 (21.11%)	3178 (21.05%)	2732 (21.13%)
>16	34090 (67.11%)	31480 (56.18%)	10090 (66.83%)	7503 (58.03%)
**Total physical activity in metabolic**	3.97 (1.68 - 8.03)	3.79 (1.51 - 8.03)	3.89 (1.66 - 8)	3.68 (1.49 - 7.95)
**Sedentary time in hours, median (range)***	4 (3 - 6)	4.5 (3.5 - 6)	4.5 (3 - 6)	5 (3.5 - 6)

For peer review only - http://bmjopen.bmj.com/site/about/guidelines.xhtml

**Diabetes, N (%)**	1268	(2.5%)	3777	(6.74%)	380	(2.52%)	929	(7.19%)
**Hypercholesterolaemia, N (%)**	3011	(5.93%)	9210	(16.44%)	893	(5.91%)	2211	(17.1%)
**Systolic BP in mmHg, median (range)***	125.5	(118 - 132)	149.5	(142 - 159.5)	124	(116 - 131)	147.5	(140.5 - 157.)
**Body Mass Index, N (%)**								
<18.5	389	(0.77%)	142	(0.25%)	103	(0.68%)	34	(0.26%)
18.5 – 25	22549	(44.39%)	13678	(24.41%)	6251	(41.4%)	2874	(22.23%)
25-30	20410	(40.18%)	25216	(45 %)	5936	(39.32%)	5389	(41.68%)
>30	7450	(14.67%)	16999	(30.34%)	2808	(18.6%)	4632	(35.83%)
**Smoking status, N (%)**								
Never smoked	30626	(60.29%)	31503	(56.22%)	7864	(52.09%)	6454	(49.92%)
Previously smoked	15056	(29.64%)	20140	(35.94%)	5118	(33.9%)	5065	(39.18%)
Current smoker	4970	(9.78%)	4199	(7.49%)	2093	(13.86%)	1381	(10.68%)
**Alcohol frequency, N (%)**								
Daily or almost daily	9450	(18.6%)	12970	(23.15%)	2736	(18.12%)	2881	(22.28%)
Three or four times a week	12175	(23.97%)	13033	(23.26%)	3253	(21.55%)	2837	(21.94%)
Once or twice a week	13644	(26.86%)	13889	(24.79%)	3880	(25.7%)	2916	(22.55%)

For peer review only - http://bmjopen.bmj.com/site/about/guidelines.xhtml

One to three times a month	6052	(11.91%)	5588	(9.97%)	2058	(13.63%)	1512	(11.69%)
Special occasions only	5534	(10.89%)	6330	(11.3%)	1904	(12.61%)	1729	(13.37%)
Never	3924	(7.72%)	4199	(7.49%)	1262	(8.36%)	1048	(8.11%)
**Psychotropic medication, N (%)**	1341	(2.64%)	1795	(3.2%)	2844	(18.84%)	2522	(19.51%)

*All data presented as N (%) and has chi-squared p-value of <0.001 except * which are median values (interquartile range) and have a Kruskal-Wallis p-value of 0.0001. Data presented as MET-hrs (hours spent doing exercise adjusted for multiples of basal metabolic rate in accordance with IPAQ).Townsend score is an area based measure based on census statistics. It is a calculation based on the number of: households without a car, overcrowded households, households not owner-occupied and unemployment.*

For peer review only - http://bmjopen.bmj.com/site/about/guidelines.xhtml

Table 2 Baseline characteristics for stroke outcomes

	Comparator group	Hypertension only	MDD only	Hypertension plus MDD
	N = 52502	N = 59724	N = 15581	N = 13947
**Median age (range)***	54 (47 - 61)	61 (55 - 65)	54 (47 - 61)	60 (53 - 64)
**Females, N (%)**	29684 (56.54%)	26937 (45.1%)	11143 (71.52%)	8090 (58.01%)
**Ethnicity, N (%)**				
White	47697 (90.85%)	54578 (91.38%)	14697 (94.33%)	13212 (94.73%)
Asian/Asian British	1857 (3.54%)	1889 (3.16%)	280 (1.8%)	209 (1.5%)
Black/ Black British	1355 (2.58%)	1854 (3.1%)	223 (1.43%)	246 (1.76%)
**Median Townsend score (range)***	-1.89 (-3.45 - 0.55)	-2.04 (-3.49 - 0.44)	-1.56 (-3.28 - 1.15)	-1.74 (-3.4 - 0.93)
**Age at leaving full-time education, N (%)**				
<16	6446 (12.28%)	13396 (22.43%)	1884 (12.09%)	2945 (21.12%)
16	10590 (20.17%)	12507 (20.94%)	3270 (20.99%)	2953 (21.17%)
>16	34914 (66.5%)	33114 (55.45%)	10317 (66.22%)	7947 (56.98%)
**Total physical activity in metabolic**	3.96 (1.67 - 8.02)	3.75 (1.5 - 8)	4.13 (1.67 - 8.36)	3.66 (1.45 - 7.83)
**Sedentary time in hours, median (range)***	4 (3 - 6)	5 (3.5 - 6)	5 (3.5 - 6.5)	5 (4 - 7)

For peer review only - http://bmjopen.bmj.com/site/about/guidelines.xhtml

**Diabetes, N (%)**	1454	(2.77%)	4502	(7.54%)	449	(2.88%)	1163	(8.34%)
**Hypercholesterolaemia, N (%)**	3592	(6.84%)	10768	(18.03%)	1049	(6.73%)	2620	(18.79%)
**Systolic BP in mmHg, median (range)***	125.5	(118 - 132)	149.5	(142 - 159.5)	127	(120.5 - 133)	147.5	(140.5 - 156.5)
**Body Mass Index, N (%)**								
<18.5	395	(0.75%)	151	(0.25%)	104	(0.67%)	38	(0.27%)
18.5 – 25	22967	(43.75%)	14242	(23.85%)	6374	(40.91%)	3017	(21.63%)
25-30	21185	(40.35%)	26817	(44.9%)	6149	(39.46%)	5769	(41.36%)
>30	7953	(15.15%)	18514	(31.%)	2954	(18.96%)	5123	(36.73%)
**Smoking status, N (%)**								
Never smoked	31318	(59.65%)	32982	(55.22%)	8052	(51.68%)	6834	(49%)
Previously smoked	15851	(30.19%)	22019	(36.87%)	5340	(34.27%)	5560	(39.87%)
Current smoker	5170	(9.85%)	4501	(7.54%)	2163	(13.88%)	1519	(10.89%)
**Alcohol frequency, N (%)**								
Daily or almost daily	9760	(18.59%)	13751	(23.02%)	2817	(18.08%)	3085	(22.12%)
Three or four times a week	12563	(23.93%)	13827	(23.15%)	3335	(21.4%)	3020	(21.65%)
Once or twice a week	14089	(26.84%)	14719	(24.65%)	3993	(25.63%)	3125	(22.41%)

For peer review only - http://bmjopen.bmj.com/site/about/guidelines.xhtml

One to three times a month	6220	(11.85%)	5971	(10%)	2122	(13.62%)	1627	(11.67%)
Special occasions only	5744	(10.94%)	6794	(11.38%)	1978	(12.69%)	1885	(13.52%)
Never	4102	(7.81%)	4630	(7.75%)	1330	(8.54%)	1199	(8.6%)
**Psychotropic medication, N (%)**	1408	(2.68%)	1996	(3.34%)	2976	(19.1%)	2778	(19.92%)

*All data presented as N (%) and has chi-squared p-value of <0.001 except * which are median values (interquartile range) and have a Kruskal-Wallis p-value of 0.0001. Data presented as MET-hrs (hours spent doing exercise adjusted for multiples of basal metabolic rate in accordance with IPAQ).Townsend score is an area based measure based on census statistics. It is a calculation based on the number of: households without a car, overcrowded households, households not owner-occupied and unemployment.*

For peer review only

For peer review only - http://bmjopen.bmj.com/site/about/guidelines.xhtml

Table 3: Risk of adverse cardiovascular event by clinical group: unadjusted, partially adjusted and fully adjusted models.

	Unadjusted			Model 1 - Sociodemographic			Model 2 - Model 1 + Health/ Lifestyle		
**Group**	HR	95% C.I.	p-value	aHR	95% C.I.	p-value	aHR	95% C.I.	p-value
No Hypertension - No MDD	1(ref)			1(ref)			1(ref)		
Hypertension only	2.60	(2.39 - 2.82)	3.31×10^{-113}	1.72	(1.57 - 1.88)	1.99×10^{-33}	1.36	(1.22 - 1.52)	2.92×10^{-8}
MDD only	0.69	(0.51 - 0.94)	0.02	0.82	(0.6 - 1.13)	0.23	0.75	(0.54 - 1.04)	0.08
Hypertension and MDD	2.84	(2.55 - 3.17)	6.31×10^{-77}	2.27	(2.02 - 2.55)	2.75×10^{-44}	1.66	(1.45 - 1.9)	7.48×10^{-14}
**Time varying Variables**									
MDD only	1.01	(1.004 - 1.02)	2.38×10^{-3}	1.01	(1.004 - 1.02)	3.19×10^{-3}	1.01	(1.004 - 1.02)	3.03×10^{-3}

**Adjusted for sociodemographic factors (age, sex, Townsend score, age of leaving full time education and ethnicity. †Additionally adjusted for history of diabetes, history of hypercholesterolemia, BMI, smoking history, alcohol use, systolic blood pressure, sedentary hours per day, physical activity and psychotropic medication use. MDD = Major depressive disorder, HR = Hazard ratio, aHR = Adjusted hazard ratio, C.I. = Confidence interval.*

For peer review only

Table 4: Risk of adverse cardiovascular event by clinical group: unadjusted, partially adjusted and fully adjusted models with hypertension as the comparator

	Unadjusted			Model 1 - Sociodemographic			Model 2 - Model 1 + Health/ Lifestyle		
**Group**	HR	95% C.I.	p-value	aHR	95% C.I.	p-value	aHR	95% C.I.	p-value
Hypertension only	1(ref)			1(ref)			1(ref)		
No Hypertension - No MDD	0.38	(0.35 - 0.42)	$3.31x10^{-113}$	0.58	(0.53 - 0.63)	$1.99x10^{-33}$	0.73	(0.66 - 0.82)	$2.92x10^{-8}$
MDD only	0.27	(0.2 - 0.36)	$1.14x10^{-17}$	0.48	(0.35 - 0.66)	$4.91x10^{-6}$	0.55	(0.4 - 0.76)	$3.23x10^{-4}$
Hypertension and MDD	1.09	(0.996 - 1.2)	0.06	1.32	(1.2 - 1.46)	$3.07x10^{-8}$	1.22	(1.1 - 1.35)	$1.30x10^{-4}$
**Time varying Variables**									
MDD only	1.01	(1.004 - 1.02)	0.002	1.01	(1.004 - 1.02)	$3.19x10^{-3}$	1.01	(1.004 - 1.02)	$3.03x10^{-3}$

**Adjusted for sociodemographic factors (age, sex, Townsend score, age of leaving full time education and ethnicity. †Additionally adjusted for history of diabetes, history of hypercholesterolemia, BMI, smoking history, alcohol use, systolic blood pressure, sedentary hours per day, physical activity and psychotropic medication use. MDD = Major depressive disorder, HR = Hazard ratio, aHR = Adjusted hazard ratio, C.I. = Confidence interval.*

For peer review only - http://bmjopen.bmj.com/site/about/guidelines.xhtml

Table 5: Risk of stroke event by clinical group: unadjusted, partially adjusted and fully adjusted models.

	Unadjusted			Model 1 - Sociodemographic			Model 2 - Model 1 + Health/ Lifestyle		
**Group**	HR	95% C.I.	p-value	aHR	95% C.I.	p-value	aHR	95% C.I.	p-value
No Hypertension- No MDD	1(ref)			1(ref)			1(ref)		
Hypertension only	2.55	(2.16 - 3.02)	$3.84x10^{-28}$	1.64	(1.38 - 1.96)	$3.35x10^{-8}$	1.21	(0.97 - 1.51)	0.09
MDD only	1.14	(0.86 - 1.52)	0.37	1.37	(1.02 - 1.84)	0.037	1.20	(0.89 - 1.63)	0.24
Hypertension and MDD	2.67	(2.13 - 3.34)	$9.79x10^{-18}$	2.05	(1.63 - 2.58)	$1.08x10^{-9}$	1.37	(1.04 - 1.79)	0.02

**Adjusted for sociodemographic factors (age, sex, Townsend score, age of leaving full time education and ethnicity. †Additionally adjusted for history of diabetes, history of hypercholesterolemia, BMI, smoking history, alcohol use, systolic blood pressure, sedentary hours per day, physical activity and psychotropic medication use. MDD = Major depressive disorder, HR = Hazard ratio, aHR = Adjusted hazard ratio, C.I. = Confidence interval.*

For peer review only

Table 6: Risk of stroke event by clinical group: unadjusted, partially adjusted and fully adjusted models with hypertension as the comparator

	Unadjusted			Model 1 - Sociodemographic			Model 2 - Model 1 + Health/ Lifestyle		
**Group**	HR	95% C.I.	p-value	aHR	95% C.I.	p-value	aHR	95% C.I.	p-value
Hypertension only	1(ref)			1(ref)			1(ref)		
No Hypertension - No MDD	0.39	(0.33 - 0.46)	$3.84x10^{-28}$	0.61	(0.51 - 0.73)	$3.35x10^{-8}$	0.82	(0.66 - 1.03)	0.09
MDD only	0.45	(0.34 - 0.58)	$1.43x10^{-9}$	0.83	(0.63 - 1.1)	0.19	0.99	(0.73 - 1.35)	0.95
Hypertension and MDD	1.05	(0.86 - 1.27)	0.64	1.25	(1.03 - 1.52)	0.03	1.13	(0.92 - 1.39)	0.26

**Adjusted for sociodemographic factors (age, sex, Townsend score, age of leaving full time education and ethnicity. †Additionally adjusted for history of diabetes, history of hypercholesterolemia, BMI, smoking history, alcohol use, systolic blood pressure, sedentary hours per day, physical activity and psychotropic medication use. MDD = Major depressive disorder, HR = Hazard ratio, aHR = Adjusted hazard ratio, C.I. = Confidence interval.*

For peer review only - http://bmjopen.bmj.com/site/about/guidelines.xhtml

632 Table 7: Fully adjusted HR compared with results from competing risks analysis for cardiovascular endpoints

Group	Fully adjusted non-competing risks analysis			Fully adjusted competing risks model		
	aHR	95% C.I.	p-value	aHR	95% C.I.	p-value
No Hypertension - No MDD	1(ref)			1(ref)		
Hypertension only	1.36	(1.22- 1.52)	2.92×10^{-8}	1.37	(1.22-1.53)	4×10^{-8}
MDD only	0.75	(0.54- 1.04)	0.08	0.76	(0.55-1.03)	0.08
Hypertension and MDD	1.66	(1.45- 1.9)	7.48×10^{-14}	1.67	(1.45-1.91)	2.2×10^{-13}
tvc						
MDD only	1.01	(1.004- 1.02)	3.03×10^{-3}	1.01	(1.004-1.02)	0.003

633 *Adjusted for age, sex, Townsend score, age of leaving full time education and ethnicity, history of diabetes, history of hypercholesterolemia, BMI, smoking*
634 *history, alcohol use, systolic blood pressure, sedentary hours per day, physical activity and psychotropic medication use. MDD = Major depressive disorder,*
635 *aHR = Adjusted hazard ratio, C.I. = Confidence interval.*

For peer review only - http://bmjopen.bmj.com/site/about/guidelines.xhtml

636 Table 8: Fully adjusted HR compared with results from competing risks analysis for stroke endpoints

	**Fully adjusted non-competing risks analysis**			**Fully adjusted competing risks model**		
**Group**	aHR	95% C.I.	p-value	aHR	95% C.I.	p-value
No Hypertension - No MDD	1(ref)			1(ref)		
Hypertension only	1.21	(0.97- 1.51)	0.09	1.21	(0.96- 1.52)	0.1
MDD only	1.20	(0.89- 1.63)	0.24	1.20	(0.88- 1.64)	0.25
Hypertension and MDD	1.37	(1.04- 1.79)	0.02	1.36	(1.03- 1.8)	0.031

637 *Adjusted for age, sex, Townsend score, age of leaving full time education and ethnicity, history of diabetes, history of hypercholesterolemia, BMI, smoking*
638 *history, alcohol use, systolic blood pressure, sedentary hours per day, physical activity and psychotropic medication use. MDD = Major depressive disorder,*
639 *aHR = Adjusted hazard ratio, C.I. = Confidence interval.*

640

641 Figure 1: Adjusted survival analysis graph for adverse cardiovascular outcome showing greatest hazard for the comorbid group. MDD appears protective compared to the
642 comparator group initially, however, shows increased hazard after 41 months. Analysis adjusted for age, sex, Townsend score, age of leaving full time education and
643 ethnicity, history of diabetes, history of hypercholesterolemia, BMI, smoking history, alcohol use, systolic blood pressure, sedentary hours per day, physical activity and
644 psychotropic medication use (MDD = Major Depressive disorder)
645
646 Figure 2: Adjusted survival analysis graph for stroke outcomes showing significantly increased hazard for comorbid Hypertension and MDD, with similar insignificant
647 increased hazard trends for hypertension only and MDD only. Analysis adjusted for age, sex, Townsend score, age of leaving full time education and ethnicity, history of
648 diabetes, history of hypercholesterolemia, BMI, smoking history, alcohol use, systolic blood pressure, sedentary hours per day, physical activity and psychotropic
649 medication use. (MDD = Major Depressive disorder)

For peer review only - http://bmjopen.bmj.com/site/about/guidelines.xhtml

Figure 3: Log (-log) plot showing non-proportionality of MDD only survival over time. Paths between the comparator group and the MDD group cross at the 22.5 month mark. Analysis adjusted for age, sex, Townsend score, age of leaving full time education and ethnicity, history of diabetes, history of hypercholesterolemia, BMI, smoking history, alcohol use, systolic blood pressure, sedentary hours per day, physical activity and psychotropic medication use. (MDD = Major Depressive disorder)

For peer review only

For peer review only - http://bmjopen.bmj.com/site/about/guidelines.xhtml

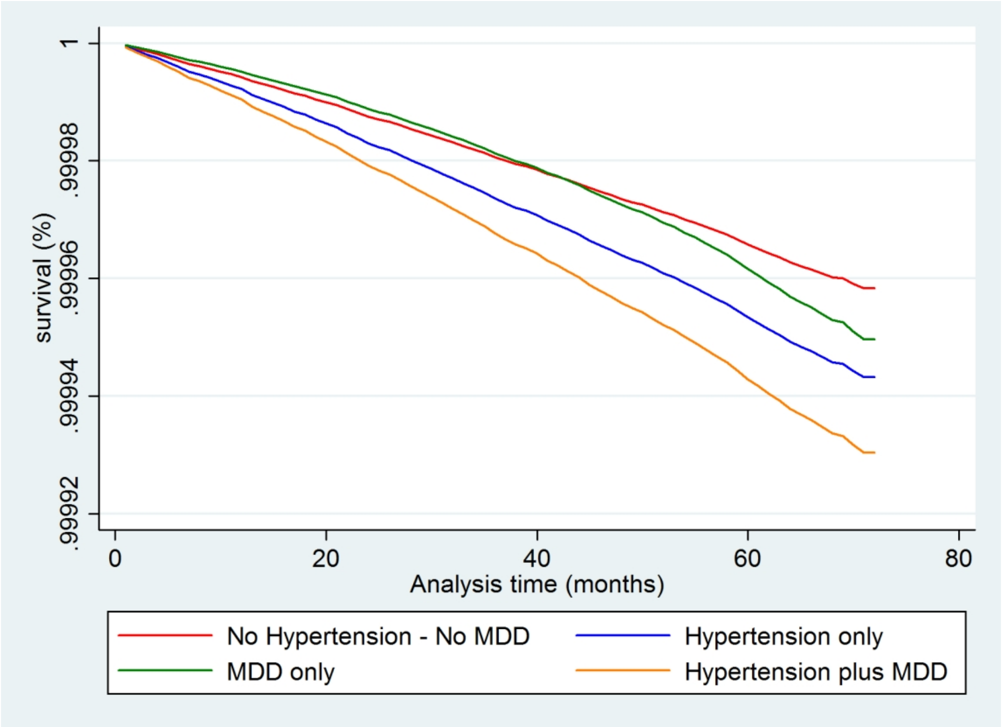

Adjusted survival analysis graph for adverse cardiovascular outcome showing greatest hazard for the comorbid group. MDD appears protective compared to the comparator group initially, however, shows increased hazard after 41 months. Analysis adjusted for age, sex, Townsend score, age of leaving full time education and ethnicity, history of diabetes, history of hypercholesterolemia, BMI, smoking history, alcohol use, systolic blood pressure, sedentary hours per day, physical activity and psychotropic medication use (MDD = Major Depressive disorder)

152x110mm (300 x 300 DPI)

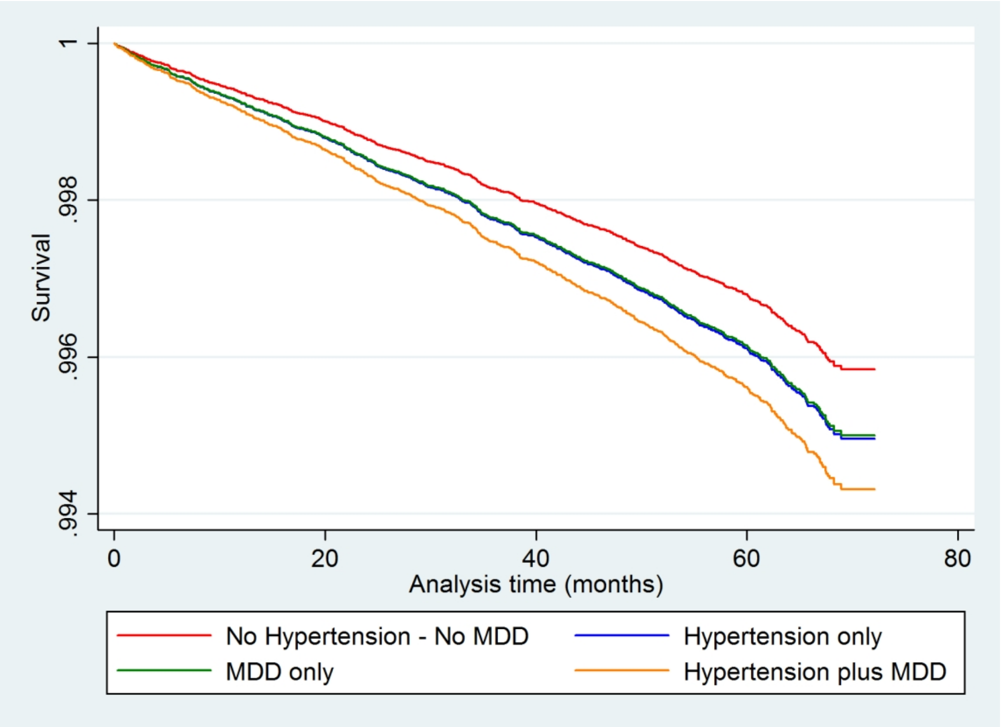

Adjusted survival analysis graph for stroke outcomes showing significantly increased hazard for comorbid Hypertension and MDD, with similar insignificant increased hazard trends for hypertension only and MDD only. Analysis adjusted for age, sex, Townsend score, age of leaving full time education and ethnicity, history of diabetes, history of hypercholesterolemia, BMI, smoking history, alcohol use, systolic blood pressure, sedentary hours per day, physical activity and psychotropic medication use. (MDD = Major Depressive disorder)

152x110mm (300 x 300 DPI)

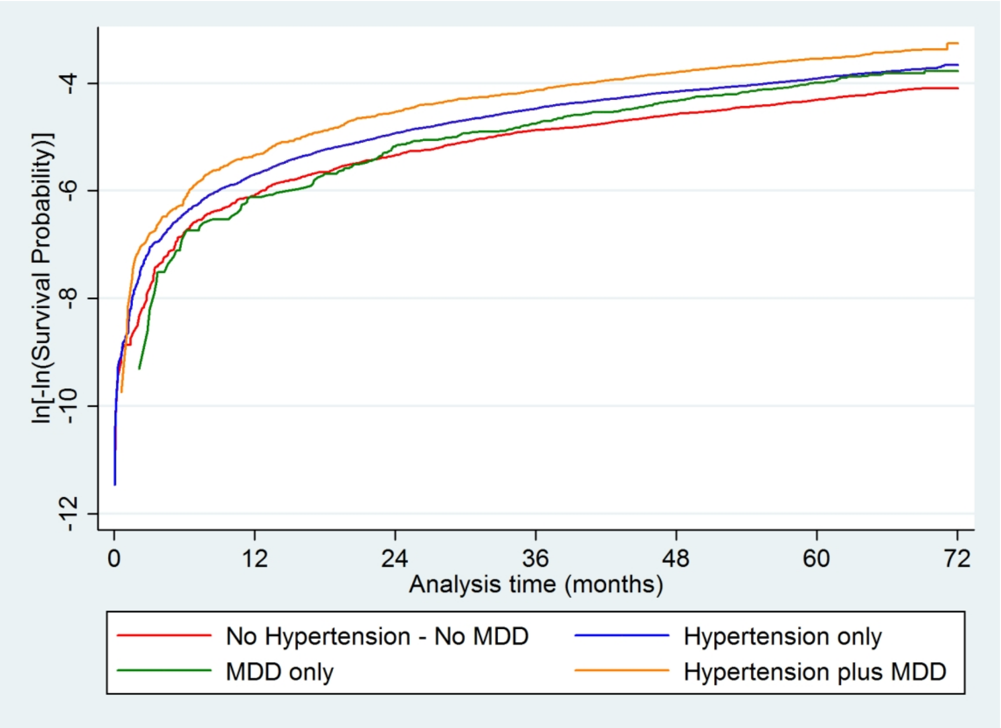

Log (-log) plot showing non-proportionality of MDD only survival over time. Paths between the comparator group and the MDD group cross at the 22.5 month mark. Analysis adjusted for age, sex, Townsend score, age of leaving full time education and ethnicity, history of diabetes, history of hypercholesterolemia, BMI, smoking history, alcohol use, systolic blood pressure, sedentary hours per day, physical activity and psychotropic medication use. (MDD = Major Depressive disorder)

152x110mm (300 x 300 DPI)

Supplementary information for Impact of major depression on cardiovascular outcomes for individuals with hypertension: prospective study in UK Biobank. Graham et al

METHODS

New-onset cardiovascular outcomes

Date and cause of death were obtained from death certificates held by the National Health Service (NHS) Information Centre for participants from England and Wales and the NHS Central Register Scotland for participants from Scotland. Date and cause of hospital admissions were identified via record linkage to Health Episode Statistics (HES) records for England, the Patient Episode Database for Wales (PEDW) and to the Scottish Morbidity Records (SMR) for Scotland. Detailed information about the record linkage procedure is available online [1 2]. At the time of analysis, mortality data were available up to 31st January 2016 for England and Wales and 11th November 2015 for Scotland. Hospital admission data were available for the Scottish, English and Welsh participants until the 31st August 2014, 31st March 2015, and 28th February 2015 respectively. Therefore, for new cardiovascular events, end of follow up was classified as the hospital admission dates unless preceded by the date of death or the date of first cardiovascular event. New onset cardiovascular events were defined as an ICD 10 code of G45, G46, I20- I25, or I6 recorded on a death certificate or hospital admission. Deaths that predated the assessment date were excluded from analysis as presumed errors as were those in which data had only recorded a death date but no cause of death or a cause of death but no death date. Participants that had hospital admissions prior to the assessment date due to the aforementioned ICD10 codes were excluded as were not first episode. In addition, ICD-9 codes 430-438, 410-414, 429 and 429.2 were also excluded.   hospital records are not available for the entire lifetime of study individuals, potentially missing some early cardiovascular events, as such those with self-declared prior cardiovascular disease at baseline were also excluded.

Blood Pressure

For peer review only - http://bmjopen.bmj.com/site/about/guidelines.xhtml

Blood pressure was measured in a sitting position partway through the interview and at the end of the interview using a digital blood pressure monitor (Omron HEM-7015IT.). Full protocol is available online https://biobank.ctsu.ox.ac.uk/crystal/docs/Bloodpressure.pdf

Depression definition

The criteria for lifetime MDD were created via the the following questions via touchscreen questionnaire were: *"Looking back over your life, have you ever had a time when you were feeling depressed or down for at least a whole week?"* (depression); *"Have you ever had a period of time lasting at least two days when you were so irritable that you found yourself shouting at people or starting fights or arguments?"* (irritability); *"How many weeks was the longest period when you were feeling depressed or down?"* (duration); *"Have you ever seen a general practitioner (GP) for nerves, anxiety, tension or depression?"* (consulted GP); *"Have you ever seen a psychiatrist for nerves, anxiety, tension or depression?"* (consulted psychiatrist). Participants were classified as having a history of MDD if they reported at least one episode which comprised of depression and/or irritability, with a duration of at least two weeks, plus had consulted with either a general practitioner or psychiatrist for mental ill-health.

Physical activity

Physical activity was based on self-report, utilising the short form International Physical Activity Questionnaire (IPAQ). Participants reported the frequency and duration of moderate and vigorous activity along with walking undertaken in a typical week [3]. Data were analysed in accordance with the IPAQ scoring protocol [4] and total physical activity was computed as the sum of walking, moderate and vigorous activity, measured as metabolic equivalents (MET-hours/week). Physical activity was used in analyses as a continuous variable. Participants who reported greater than 24 hours a day doing all activity were classified as missing.

For peer review only - http://bmjopen.bmj.com/site/about/guidelines.xhtml

Sedentary behaviour

Sedentary behaviour duration was derived from the sum of self-reported time spent driving, using computer and watching television. Those stating that they had performed “less than an hour” of sedentary activities were coded as 0.5hrs to allow use of a continuous variable. Participants who reported greater than 24 hours a day doing all activity were classified as missing.

Socio-demographic and other covariates

Self-report on taking antihypertensive medication was taken from a question specific to cardiovascular medications, where antihypertensive medication was an option to respond. Area-based socioeconomic status was derived from postcode of residence, utilising the census-derived Townsend deprivation index scored on housing, employment, social class and car availability where a negative score represents greater affluence [5 6]. Age was calculated from dates of birth and baseline assessment date. Smoking status was categorised into never, former and current smoking based on self-report, those who wished not to answer were coded as missing. Drink frequency was categorised into daily, three or four times a week, once or twice a week, one to three times a month, special occasions only, and never based on self-report. Those who wished not to answer were coded as missing. Medical history of diabetes and high cholesterol was collected from the self-completed, baseline assessment questionnaire of medical conditions. Ethnicity was categorised as Caucasian, black/mixed and Asian/mixed based on self-report. Other ethnicities coded as missing due to small numbers. Age at completing full-time education was categorised as (<16, 16, >16). Height and body weight were measured by trained nurses during the initial assessment centre visit. Body mass index (BMI) was calculated as (weight/height^2) and the WHO criteria[7] to classify BMI into: underweight <18.5, normal weight 18.5-24.9, overweight 25.0-29.9 and obese ≥30.0 kg.m^{-2}. Psychotropic medication use was defined by the presence of pharmaceuticals from British National Formulary (BNF) chapters 4.1.1 to 4.3.4 [8] on self-report medication lists at baseline. Duration of hypertension was calculated utilising age and age of hypertension diagnosis. Antihypertensive medication count

For peer review only - http://bmjopen.bmj.com/site/about/guidelines.xhtml

was calculated as the absolute number of ACE inhibitors, angiotensin II receptor antagonists, calcium channel blockers, beta-blockers and thiazide diuretics prescribed to an individual. Generic medication names were sought and cross-referenced with the BNF chapters 2.2.1, 2.4, 2.5.5 and 2.6.2 [8].

Statistical analysis:

A best-fit multivariable regression spline model (stata command "mvrs") was used to find the best model to adjust for non-linear covariates. For the adverse cardiovascular outcomes, A single knot was fitted for age at age 50 and two knots were fitted for total physical activity at 1.65 and 8.062 metabolic equivalent hours. In the male subgroup analysis two knots were fitted for total physical activity at 1.7 and 8.507 metabolic equivalent hours, in the female subgroup two knots were fitted for total physical activity at 1.57 and 3.75 and two knots were fitted at systolic blood pressure 121.5 and 147.5. No bends were noted in any models for the stroke outcomes.

Model selection and covariate adjustment

All variables were tested against outcome measures (cardiovascular outcomes and stroke outcomes) using univariate analysis to assess appropriateness for inclusion in the final model. All covariates were significantly associated with the outcomes. and were Two continuous variables, age and total physical activity, expressed non-linearity within the main analysis and male subgroup analysis for cardiovascular outcomes and as such regression splines were used with two and three knots respectively. Two knots were included within the female subgroup analysis for physical activity. For stroke outcome there were no bends in the main or sex-specific models.

Within the main analysis for cardiovascular outcomes, the groups of depression only, Asian/Asian British ethnicity and BMI<18.5 covariates failed the proportionality assumption and as such, were incorporated into the model as a time varying coefficients. Within the sex specific models depression

For peer review only - http://bmjopen.bmj.com/site/about/guidelines.xhtml

only failed the PH test within the female only analysis and ethnicity and BMI failed within the male only analysis. For the stroke outcomes gender and BMI class failed the proportionality assumption within the main analysis, with no failures within the sex-specific analysis. Analysis was repeated with the hypertension only as the comparator group to assess for any significant difference between the co-morbid group and the hypertension only group.

Time varying covariates

Due to the finding of MDD failing the proportionality assumption in the cardiovascular outcome in the primary analysis a series of further analyses have been performed to find when the assumption was not met. A log (-log) plot (fig 3) showed the proportionality assumption was broken at 22.5 months in the fully adjusted model in the primary analysis. As such, separate models were performed prior to and after these points. Prior to 22.5months the HR for MDD shows a trend that is reduced but insignificant (HR 0.82, 95%CI 0.6 - 1.13), becoming significantly increased after the 22.5 time point. (HR 1.27, 95%CI 1.06 - 1.52) (Table 9 supplementary digital content). Both stratified models passed the proportionality assumption using Schoenfeld residuals. Similar to the major analysis, the female model showed the MDD only group failing the proportionality assumption, although this was at the 29 month time point. (tables 6 and 10 of the supplementary digital content).

For peer review only - http://bmjopen.bmj.com/site/about/guidelines.xhtml

References

1. Palmer LJ. UK Biobank: bank on it. *Lancet* 2007;369(9578):1980-2. doi: 10.1016/S0140-6736(07)60924-6
2. Sudlow C, Gallacher J, Allen N, et al. UK Biobank: An Open Access Resource for Identifying the Causes of a Wide Range of Complex Diseases of Middle and Old Age. *Plos Medicine* 2015;12(3) doi: 10.1371/journal.pmed.1001779
3. Guo W, Bradbury KE, Reeves GK, et al. Physical activity in relation to body size and composition in women in UK Biobank. *Annals of Epidemiology* 2015;25(6):406-13.e6. doi: http://dx.doi.org/10.1016/j.annepidem.2015.01.015
4. Craig CL, Marshall AL, Sjostrom M, et al. International physical activity questionnaire: 12-country reliability and validity. *Medicine and science in sports and exercise* 2003;35(8):1381-95. doi: 10.1249/01.mss.0000078924.61453.fb [published Online First: 2003/08/06]
5. Townsend P. Deprivation. *Journal of Social Policy* 2009;16(02):125. doi: 10.1017/s0047279400020341
6. Townsend P, Phillimore M, Beattie A. Health and Deprivation: Inequality and the North. London: Croom Helm Ltd 1988.
7. WHO. Obesity: preventing and managing the global epidemic. Report of a WHO consultation. *World Health Organ Tech Rep Ser* 2000;894:i-xii, 1-253.
8. COMMITTEE. JF. British National Formulary. 67 ed. London: BMJ Group and Pharmaceutical Press 2014.

For peer review only - http://bmjopen.bmj.com/site/about/guidelines.xhtml

For peer review only

For peer review only - http://bmjopen.bmj.com/site/about/guidelines.xhtml

Supplementary Tables and figures

Supplementary Table1: Descriptive analysis for adverse cardiovascular outcome – males only

	Comparator group		Hypertension only		MDD only		Hypertension plus MDD	
	N= 21570		N = 30142		N = 4169		N = 5253	
**Median age (range)***	54	(47 - 61)	61	(54 - 65)	53	(46 - 60)	59	(52 - 64)
**Ethnicity, N (%)**								
White	19562	(90.69%)	27808	(92.26%)	3923	(94.1%)	5001	(95.2%)
Asian/Asian British	863	(4.%)	969	(3.21%)	87	(2.09%)	86	(1.64%)
Black/ Black British	559	(2.59%)	780	(2.59%)	52	(1.25%)	54	(1.03%)
**Median Townsend score (range)***	-1.87	(-3.47 - 0.59)	-2.08	(-3.53 - 0.41)	-1.58	(-3.3 - 1.07)	-1.81	(-3.44 - 0.78)
**Age at leaving full-time education, N (%)**								
<16	2517	(11.67%)	6328	(20.99%)	464	(11.13%)	1005	(19.13%)
16	4473	(20.74%)	6235	(20.69%)	859	(20.6%)	1096	(20.86%)
>16	14344	(66.5%)	17257	(57.25%)	2807	(67.33%)	3118	(59.36%)
**Total physical activity in metabolic**	4.15	(1.75 - 8.51)	3.99	(1.65 - 8.51)	4.15	(1.7 - 8.36)	3.76	(1.54 - 7.97)

For peer review only - http://bmjopen.bmj.com/site/about/guidelines.xhtml

**Sedentary time in hours, median (range)***	4.5	(3.5 - 6)	5	(3.5 - 6.5)	5	(3.5 - 6.5)	5	(4 - 7)
**Diabetes, N (%)**	721	(3.34%)	2401	(7.97%)	159	(3.81%)	477	(9.08%)
**Hypercholesterolaemia, N (%)**	1614	(7.48%)	5585	(18.53%)	363	(8.71%)	1056	(20.1%)
**Systolic BP in mmHg, median (range)***	128	(121.5 - 133.5)	149.5	(142 - 159)	127.5	(120.5 - 133)	148	(141 - 157)
**Body Mass Index, N (%)**								
<18.5	74	(0.34%)	35	(0.12%)	22	(0.53%)	12	(0.23%)
18.5 – 25	7607	(35.27%)	5842	(19.38%)	1394	(33.44%)	890	(16.94%)
25-30	10594	(49.11%)	15114	(50.14%)	2019	(48.43%)	2532	(48.2%)
>30	3295	(15.28%)	9151	(30.36%)	734	(17.61%)	1819	(34.63%)
**Smoking status, N (%)**								
Never smoked	12038	(55.81%)	15145	(50.25%)	1999	(47.95%)	2268	(43.18%)
Previously smoked	6777	(31.42%)	12125	(40.23%)	1447	(34.71%)	2295	(43.69%)
Current smoker	2688	(12.46%)	2776	(9.21%)	716	(17.17%)	686	(13.06%)
**Alcohol frequency, N (%)**								
Daily or almost daily	4822	(22.36%)	8653	(28.71%)	969	(23.24%)	1503	(28.61%)
Three or four times a week	5718	(26.51%)	7913	(26.25%)	1022	(24.51%)	1323	(25.19%)

For peer review only - http://bmjopen.bmj.com/site/about/guidelines.xhtml

Once or twice a week	5932	(27.5%)	7546	(25.03%)	1063	(25.5%)	1178	(22.43%)
One to three times a month	2193	(10.17%)	2392	(7.94%)	440	(10.55%)	479	(9.12%)
Special occasions only	1554	(7.2%)	2154	(7.15%)	328	(7.87%)	423	(8.05%)
Never	1343	(6.23%)	1473	(4.89%)	345	(8.28%)	345	(6.57%)
**Psychotropic medication, N (%)**	398	(1.85%)	670	(2.22%)	678	(16.26%)	879	(16.73%)

*All data presented as N (%) and has chi-squared p-value of <0.001 except * which are median values (interquartile range) and have a Kruskal-Wallis p-value of 0.0001. Data presented as MET-hrs (hours spent doing exercise adjusted for multiples of basal metabolic rate in accordance with IPAQ).Townsend score is an area based measure based on census statistics. It is a calculation based on the number of: households without a car, overcrowded households, households not owner-occupied and unemployment.*

For peer review only - http://bmjopen.bmj.com/site/about/guidelines.xhtml

For peer review only

Supplementary Table 2: Descriptive analysis for adverse cardiovascular outcome – females only

	Comparator group	Hypertension only	MDD only	Hypertension plus MDD
	N= 29228	N = 25893	N = 10929	N = 7676
**Median age (range)***	54 (47 - 61)	61 (55 - 65)	53 (47 - 60)	60 (53 - 64)
**Ethnicity, N (%)**				
White	26585 (90.96%)	23441 (90.53%)	10324 (94.46%)	7271 (94.72%)
Asian/Asian British	908 (3.11%)	727 (2.81%)	174 (1.59%)	93 (1.21%)
Black/ Black British	764 (2.61%)	989 (3.82%)	167 (1.53%)	168 (2.19%)
**Median Townsend score (range)***	-1.90 (-3.44 - 0.51)	-2.06 (-3.5 - 0.38)	-1.66 (-3.3 - 0.84)	-1.87 (-3.4 - 0.74)
**Age at leaving full-time education, N (%)**				
<16	3399 (11.63%)	5757 (22.23%)	1261 (11.54%)	1602 (20.87%)
16	5792 (19.82%)	5592 (21.6%)	2319 (21.22%)	1636 (21.31%)
>16	19746 (67.56%)	14223 (54.93%)	7283 (66.64%)	4385 (57.13%)
**Total physical activity in metabolic**	3.87 (1.65 - 7.71)	3.51 (1.37 - 7.59)	3.79 (1.65 - 7.91)	3.65 (1.45 - 7.93)
**Sedentary time in hours, median (range)***	4 (3 - 5)	4 (3 - 5.5)	4 (3 - 5.5)	4.5 (3 - 6)

For peer review only - http://bmjopen.bmj.com/site/about/guidelines.xhtml

**Diabetes, N (%)**	547	(1.87%)	1376	(5.31%)	221	(2.02%)	452	(5.89%)
**Hypercholesterolaemia, N (%)**	1397	(4.78%)	3625	(14.%)	530	(4.85%)	1155	(15.05%)
**Systolic BP in mmHg, median (range)***	123.5	(115.5 - 130.5)	149.5	(142 - 160)	122.5	(114.5 - 130)	147.5	(140.5 - 157)
**Body Mass Index, N (%)**								
<18.5	315	(1.08%)	107	(0.41%)	81	(0.74%)	22	(0.29%)
18.5 – 25	14942	(51.12%)	7836	(30.26%)	4857	(44.44%)	1984	(25.85%)
25-30	9816	(33.58%)	10102	(39.01%)	3917	(35.84%)	2857	(37.22%)
>30	4155	(14.22%)	7848	(30.31%)	2074	(18.98%)	2813	(36.65%)
**Smoking status, N (%)**								
Never smoked	18588	(63.6%)	16358	(63.18%)	5865	(53.66%)	4186	(54.53%)
Previously smoked	8279	(28.33%)	8015	(30.95%)	3671	(33.59%)	2770	(36.09%)
Current smoker	2282	(7.81%)	1423	(5.5%)	1377	(12.6%)	695	(9.05%)
**Alcohol frequency, N (%)**								
Daily or almost daily	4628	(15.83%)	4317	(16.67%)	1767	(16.17%)	1378	(17.95%)
Three or four times a week	6457	(22.09%)	5120	(19.77%)	2231	(20.41%)	1514	(19.72%)
Once or twice a week	7712	(26.39%)	6343	(24.5%)	2817	(25.78%)	1738	(22.64%)

For peer review only - http://bmjopen.bmj.com/site/about/guidelines.xhtml

One to three times a month	3859	(13.2%)	3196	(12.34%)	1618	(14.8%)	1033	(13.46%)
Special occasions only	3980	(13.62%)	4176	(16.13%)	1576	(14.42%)	1306	(17.01%)
Never	2581	(8.83%)	2726	(10.53%)	917	(8.39%)	703	(9.16%)
**Psychotropic medication, N (%)**	943	(3.23%)	1125	(4.34%)	2166	(19.82%)	1643	(21.4%)

*All data presented as N (%) and has chi-squared p-value of <0.001 except * which are median values (interquartile range) and have a Kruskal-Wallis p-value of 0.0001. Data presented as MET-hrs (hours spent doing exercise adjusted for multiples of basal metabolic rate in accordance with IPAQ).Townsend score is an area based measure based on census statistics. It is a calculation based on the number of: households without a car, overcrowded households, households not owner-occupied and unemployment.*

For peer review only

Supplementary Table 3: Descriptive analysis for stroke outcome – males only

	Comparator group		Hypertension only		MDD only		Hypertension plus MDD	
	N = 22816		N = 32787		N = 4438		N = 5857	
**Median age (range)***	55	(47 - 62.)	61	(54 - 65)	54	(47 - 61)	60	(53 - 64)
**Ethnicity, N (%)**								
White	20699	(90.72%)	30219	(92.17%)	4173	(94.03%)	5569	(95.08%)
Asian/Asian British	932	(4.08%)	1116	(3.4%)	102	(2.3%)	105	(1.79%)
Black/ Black British	576	(2.52%)	820	(2.5%)	53	(1.19%)	59	(1.01%)
**Median Townsend score (range)***	-1.88	(-3.47 - 0.59)	-2.05	(-3.5 - 0.46)	-1.56	(-3.28 - 1.15)	-1.74	(-3.4 - 0.93)
**Age at leaving full-time education, N (%)**								
<16	2900	(12.71%)	7256	(22.13%)	558	(12.57%)	1193	(20.37%)
16	4702	(20.61%)	6704	(20.45%)	909	(20.48%)	1222	(20.86%)
>16	14960	(65.57%)	18471	(56.34%)	2930	(66.02%)	3397	(58.%)
**Total physical activity in metabolic**	4.12	(1.74 - 8.48)	3.96	(1.65 - 8.44)	4.13	(1.67 - 8.36)	3.66	(1.45 - 7.83)
**Sedentary time in hours, median (range)***	5	(3.5 - 6)	5	(4 - 7)	5	(3.5 - 6.5)	5	(4 - 7)

For peer review only - http://bmjopen.bmj.com/site/about/guidelines.xhtml

**Diabetes, N (%)**	873	(3.83%)	2951	(9.%)	208	(4.69%)	635	(10.84%)
**Hypercholesterolaemia, N (%)**	2045	(8.96%)	6736	(20.54%)	457	(10.3%)	1293	(22.08%)
**Systolic BP in mmHg, median (range)***	128	(121.5 - 133.5)	149	(142 - 159)	127	(120.5 - 133)	147.5	(140.5 - 156.5)
**Body Mass Index, N (%)**								
<18.5	79	(0.35%)	39	(0.12%)	22	(0.5%)	12	(0.2%)
18.5 – 25	7867	(34.48%)	6215	(18.96%)	1452	(32.72%)	960	(16.39%)
25-30	11203	(49.1%)	16341	(49.84%)	2142	(48.26%)	2780	(47.46%)
>30	3667	(16.07%)	10192	(31.09%)	822	(18.52%)	2105	(35.94%)
**Smoking status, N (%)**								
Never smoked	12502	(54.79%)	16054	(48.96%)	2094	(47.18%)	2469	(42.15%)
Previously smoked	7399	(32.43%)	13603	(41.49%)	1582	(35.65%)	2610	(44.56%)
Current smoker	2836	(12.43%)	3013	(9.19%)	754	(16.99%)	770	(13.15%)
**Alcohol frequency, N (%)**								
Daily or almost daily	5085	(22.29%)	9309	(28.39%)	1021	(23.01%)	1645	(28.09%)
Three or four times a week	6039	(26.47%)	8556	(26.1%)	1077	(24.27%)	1450	(24.76%)
Once or twice a week	6264	(27.45%)	8161	(24.89%)	1121	(25.26%)	1305	(22.28%)

For peer review only - http://bmjopen.bmj.com/site/about/guidelines.xhtml

One to three times a month	2307	(10.11%)	2642	(8.06%)	478	(10.77%)	538	(9.19%)
Special occasions only	1666	(7.3%)	2394	(7.3%)	355	(8.%)	503	(8.59%)
Never	1444	(6.33%)	1711	(5.22%)	383	(8.63%)	414	(7.07%)
**Psychotropic medication, N (%)**	429	(1.88%)	793	(2.42%)	735	(16.56%)	1025	(17.5%)

*All data presented as N (%) and has chi-squared p-value of <0.001 except * which are median values (interquartile range) and have a Kruskal-Wallis p-value of 0.0001. Data presented as MET-hrs (hours spent doing exercise adjusted for multiples of basal metabolic rate in accordance with IPAQ).Townsend score is an area based measure based on census statistics. It is a calculation based on the number of: households without a car, overcrowded households, households not owner-occupied and unemployment.*

For peer review only - http://bmjopen.bmj.com/site/about/guidelines.xhtml

Supplementary Table 4: Descriptive analysis for stroke outcome – females only

	Comparator group		Hypertension only		MDD only		Hypertension plus MDD	
	N = 29684		N = 26937		N = 11143		N = 8090	
**Median age (range)***	54	(47 - 61)	61	(56 - 65)	53	(47 - 60)	60	(54 - 64)
**Ethnicity, N (%)**								
White	26998	(90.95%)	24359	(90.43%)	10524	(94.44%)	7643	(94.47%)
Asian/Asian British	925	(3.12%)	773	(2.87%)	178	(1.6%)	104	(1.29%)
Black/ Black British	779	(2.62%)	1034	(3.84%)	170.00	(1.53%)	187	(2.31%)
**Median Townsend score (range)***	-1.90	(-3.44 - 0.52)	-2.03	(-3.48 - 0.43)	-1.66	(-3.29 - 0.86)	-1.83	(-3.38 - 0.85)
**Age at leaving full-time education, N (%)**								
<16	3546	(11.95%)	6140	(22.79%)	1326	(11.9%)	1752	(21.66%)
16	5888	(19.84%)	5803	(21.54%)	2361	(21.19%)	1731	(21.4%)
>16	19954	(67.22%)	14643	(54.36%)	7387	(66.29%)	4550	(56.24%)
**Total physical activity in metabolic**	3.85	(1.65 - 7.7)	3.49	(1.35 - 7.57)	3.79	(1.65 - 7.89)	3.61	(1.41 - 7.87)

For peer review only - http://bmjopen.bmj.com/site/about/guidelines.xhtml

**Sedentary time in hours, median (range)***	4.0	(3 - 5)	4.0	(3 - 5.5)	4.0	(3 - 5.5)	4.5	(3 - 6)
**Diabetes, N (%)**	581	(1.96%)	1551	(5.76%)	241	(2.16%)	528	(6.53%)
**Hypercholesterolaemia, N (%)**	1547	(5.21%)	4032	(14.97%)	592	(5.31%)	1327	(16.4%)
**Systolic BP in mmHg, median (range)***	123.5	(115.5 - 131)	149.5	(142 - 160)	122.5	(114.5 - 130)	147.0	(140.5 - 157)
**Body Mass Index, N (%)**								
<18.5	316	(1.06%)	112	(0.42%)	82	(0.74%)	26	(0.32%)
18.5 – 25	15100	(50.87%)	8027	(29.8%)	4922	(44.17%)	2057	(25.43%)
25-30	9982	(33.63%)	10476	(38.89%)	4007	(35.96%)	2989	(36.95%)
>30	4286	(14.44%)	8322	(30.89%)	2132	(19.13%)	3018	(37.31%)
**Smoking status, N (%)**								
Never smoked	18816	(63.39%)	16928	(62.84%)	5958	(53.47%)	4365	(53.96%)
Previously smoked	8452	(28.47%)	8416	(31.24%)	3758	(33.73%)	2950	(36.46%)
Current smoker	2334	(7.86%)	1488	(5.52%)	1409	(12.64%)	749	(9.26%)
**Alcohol frequency, N (%)**								

Daily or almost daily	4675 (15.75%)	4442 (16.49%)	1796 (16.12%)	1440 (17.8%)
Three or four times a week	6524 (21.98%)	5271 (19.57%)	2258 (20.26%)	1570 (19.41%)
Once or twice a week	7825 (26.36%)	6558 (24.35%)	2872 (25.77%)	1820 (22.5%)
One to three times a month	3913 (13.18%)	3329 (12.36%)	1644 (14.75%)	1089 (13.46%)
Special occasions only	4078 (13.74%)	4400 (16.33%)	1623 (14.57%)	1382 (17.08%)
Never	2658 (8.95%)	2919 (10.84%)	947 (8.5%)	785 (9.7%)
**Psychotropic medication, N (%)**	979 (3.3%)	1203 (4.47%)	2241 (20.11%)	1753 (21.67%)

*All data presented as N (%) and has chi-squared p-value of <0.001 except * which are median values (interquartile range) and have a Kruskal-Wallis p-value of 0.0001. Data presented as MET-hrs (hours spent doing exercise adjusted for multiples of basal metabolic rate in accordance with IPAQ).Townsend score is an area based measure based on census statistics. It is a calculation based on the number of: households without a car, overcrowded households, households not owner-occupied and unemployment.*

For peer review only - http://bmjopen.bmj.com/site/about/guidelines.xhtml

Supplementary Table 5: Risk of adverse cardiovascular event by clinical group, in males only.

	Model one (unadjusted)			Model two (partially adjusted)*			Model three (fully adjusted) †		
**Group**	HR	95% C.I.	p-value	aHR	95% C.I.	p-value	aHR	95% C.I.	p-value
No Hypertension- No MDD	1(ref)			1(ref)			1(ref)		
Hypertension only	2.21	(2.00-2.45)	2.28×10^{-53}	1.62	(1.46-1.83)	5.80×10^{-19}	1.29	(1.13-1.47)	1.35×10^{-4}
MDD only	1.17	(0.95-1.56)	0.12	1.18	(0.95-1.46)	0.12	1.12	(0.9-1.39)	0.3
Hypertension and MDD	2.46	(2.13-2.84)	3.12×10^{-34}	1.95	(1.68-2.27)	2.81×10^{-18}	1.47	(1.24-1.74)	8.71×10^{-6}

**Adjusted for sociodemographic factors (age, Townsend score, age of leaving full time education and ethnicity. †Additionally adjusted for history of diabetes, history of hypercholesterolemia, BMI, smoking history, alcohol use, systolic blood pressure, sedentary hours per day, physical activity and psychotropic medication use. MDD = Major depressive disorder, HR = Hazard ratio, aHR = Adjusted hazard ratio, C.I.= Confidence interval*

For peer review only - http://bmjopen.bmj.com/site/about/guidelines.xhtml

Supplementary Table 6: Risk of adverse cardiovascular event by clinical group, in females only.

	Model one (unadjusted)			Model two (partially adjusted)*			Model three (fully adjusted) †		
**Group**	HR	95% C.I.	p-value	aHR	95% C.I.	p-value	aHR	95% C.I.	p-value
No Hypertension - No MDD	1(ref)			1(ref)			1(ref)		
Hypertension only	2.75	(2.38 - 3.18)	6.16×10^{-43}	1.86	(1.6-2.17)	1.43×10^{-15}	1.64	(1.33-2.02)	4.36×10^{-6}
MDD only	0.67	(0.42-1.08)	0.10	0.72	(0.45-1.17)	0.19	0.68	(0.42-1.1)	0.12
Hypertension and MDD	3.68	(3.1-4.38)	5.62×10^{-49}	2.78	(1.58-3.29)	4.62×10^{-29}	2.18	(1.82-2.92)	4.76×10^{-11}
**Time varying Variables**									
MDD only	1.02	(1.006-1.03)	2.45×10^{-3}	1.02	(1.005-1.03)	4.00×10^{-3}	1.02	(1.004-1.03)	6.19×10^{-3}

**Adjusted for sociodemographic factors (age, Townsend score, age of leaving full time education and ethnicity. †Additionally adjusted for history of diabetes, history of hypercholesterolemia, BMI, smoking history, alcohol use, systolic blood pressure, sedentary hours per day, physical activity and psychotropic medication use. MDD = Major depressive disorder, HR = Hazard ratio, aHR = Adjusted hazard ratio, C.I.= Confidence interval*

For peer review only - http://bmjopen.bmj.com/site/about/guidelines.xhtml

Supplementary Table 7: Risk of stroke event by clinical group, in males only.

	Model one (unadjusted)			Model two (partially adjusted)*			Model three (fully adjusted) †		
**Group**	HR	95% C.I.	p-value	aHR	95% C.I.	p-value	aHR	95% C.I.	p-value
No Hypertension - No MDD	1(ref)			1(ref)			1(ref)		
Hypertension only	2.43	(1.95 - 3.03)	1.92×10^{-15}	1.74	(1.38 - 2.19)	2.58×10^{-6}	1.19	(0.9 - 1.58)	0.22
MDD only	1.45	(0.96 - 2.2)	0.07	1.65	(1.09 - 2.5)	0.02	1.49	(0.97 - 2.29)	0.07
Hypertension and MDD	2.39	(1.74 - 3.27)	7.34×10^{-8}	1.87	(1.35 - 2.6)	1.55×10^{-4}	1.20	(0.83 - 1.74)	0.33

**Adjusted for sociodemographic factors (age, sex, Townsend score, age of leaving full time education and ethnicity. †Additionally adjusted for history of diabetes, history of hypercholesterolemia, BMI, smoking history, alcohol use, systolic blood pressure, sedentary hours per day, physical activity and psychotropic medication use. MDD = Major depressive disorder, HR = Hazard ratio, aHR = Adjusted hazard ratio, C.I.= Confidence interval*

For peer review only - http://bmjopen.bmj.com/site/about/guidelines.xhtml

Supplementary Table 8: Risk of stroke event by clinical group, in females only.

	Model one (unadjusted)			Model two (partially adjusted)*			Model three (fully adjusted) †		
**Group**	HR	95% C.I.	p-value	aHR	95% C.I.	p-value	aHR	95% C.I.	p-value
No Hypertension - No MDD	1(ref)			1(ref)			1(ref)		
Hypertension only	2.38	(1.84 - 3.09)	6.50×10^{-11}	1.51	(1.14 - 1.99)	3.63×10^{-3}	1.25	(0.88 - 1.79)	0.21
MDD only	1.09	(0.73 - 1.62)	0.67	1.15	(0.76 - 1.75)	0.51	0.99	(0.64 - 1.53)	0.98
Hypertension and MDD	3.05	(2.22 - 4.21)	8.71×10^{-12}	2.22	(1.59 - 3.08)	2.27×10^{-6}	1.62	(1.08 - 2.42)	0.02

**Adjusted for sociodemographic factors (age, sex, Townsend score, age of leaving full time education and ethnicity. †Additionally adjusted for history of diabetes, history of hypercholesterolemia, BMI, smoking history, alcohol use, systolic blood pressure, sedentary hours per day, physical activity and psychotropic medication use. MDD = Major depressive disorder, HR = Hazard ratio, aHR = Adjusted hazard ratio, C.I.= Confidence interval*

For peer review only - http://bmjopen.bmj.com/site/about/guidelines.xhtml

Supplementary Table 9: Time stratified analysis by moment of proportional hazards failure for adverse cardiovascular outcomes (stratified at 22.5 months)

	Fully adjusted* model pre-22.5 months			Fully adjusted* model post-22.5 months		
**Group**	aHR	95% C.I.	p-value	aHR	95% C.I.	p-value
No Hypertension - No MDD	1(ref)			1(ref)		
Hypertension only	1.36	(1.12 - 1.66)	0.002	1.36	(1.19 -1.55)	$5.06x10^{-6}$
MDD only	0.82	(0.60 - 1.13)	0.22	1.27	(1.06 -1.52)	0.01
Hypertension and MDD	1.75	(1.39 - 2.21)	$2.62x10^{-6}$	1.62	(1.38 - 1.90)	$5.72x10^{-9}$

**Adjusted for sociodemographic factors (age, sex, Townsend score, age of leaving full time education and ethnicity, history of diabetes, history of hypercholesterolemia, BMI, smoking history, alcohol use, systolic blood pressure, sedentary hours per day, physical activity and psychotropic medication use.*

MDD = Major depressive disorder, aHR =adjusted hazard ratio, C.I.= Confidence interval

For peer review only - http://bmjopen.bmj.com/site/about/guidelines.xhtml

Supplementary Table 10: Time stratified analysis by moment of proportional hazards failure for adverse cardiovascular outcomes (females only - stratified at 29 months)

	Fully adjusted* model pre-29 months			Fully adjusted* model post-29 months		
**Group**	HR	95% C.I.	p-value	HR	95% C.I.	p-value
No Hypertension - No MDD	1(ref)			1(ref)		
Hypertension only	1.49	(1.06 - 2.08)	0.02	1.75	(1.33 - 2.30)	5.56×10^{-5}
MDD only	0.73	(0.48 - 1.10)	0.13	1.58	(1.19 - 2.09)	0.002
Hypertension and MDD	1.80	(1.24 - 2.62)	0.002	2.47	(1.83 - 3.33)	2.89×10^{-9}

**Adjusted for sociodemographic factors (age, sex, Townsend score, age of leaving full time education and ethnicity, history of diabetes, history of hypercholesterolemia, BMI, smoking history, alcohol use, systolic blood pressure, sedentary hours per day, physical activity and psychotropic medication use.*

MDD = Major depressive disorder, aHR =adjusted hazard ratio, C.I.= Confidence interval

For peer review only - http://bmjopen.bmj.com/site/about/guidelines.xhtml

Supplementary Table 11: Relative excess risk due to interaction results on fully adjusted* models

Analysis	RERI	95% C.I.	LR test p-value
Adverse cardiovascular outcome before 22.5 months	**0.563**	**(0.189 - 0.938)**	**0.0116**
Adverse cardiovascular outcome after 22.5 months	-0.009	(-0.293 - 0.275)	0.563
Adverse cardiovascular outcome (males only)	0.058	(-0.240 - 0.357)	0.899
Adverse cardiovascular outcome (females only)before 29 months	**0.588**	**(0.074 - 1.103)**	**0.031**
Adverse cardiovascular outcome (females only)after 29 months	0.142	(-0.447 - 0.732)	0.5173
Stroke outcome	-0.047	(-0.485 - 0.391)	0.7271
Stroke outcome (males only)	-0.480	(-1.195 - 0.234)	0.1376
Stroke outcome (females only)	0.372	(-0.216 - 0.959)	0.314

**Adjusted for sociodemographic factors (age, sex, Townsend score, age of leaving full time education and ethnicity, history of diabetes, history of hypercholesterolemia, BMI, smoking history, alcohol use, systolic blood pressure, sedentary hours per day, physical activity and psychotropic medication use.*

RERI = Relative excess risk due to interaction (additive interaction), C.I.= Confidence interval, LR test = likelihood ratio test (multiplicative interaction)

For peer review only - http://bmjopen.bmj.com/site/about/guidelines.xhtml

Supplementary Table 12: Comparison of additional hypertension factors (medication and diagnosis duration) across groups

	No Hypertension – No MDD		Hypertension only		MDD only		Hypertension and MDD	
**Antihypertensive medication prescription, N (%)**	1,265	(2.49)	19,045	(33.99)	476	(3.04)	5,037	(37.34)
**Number of antihypertensive medications, N (range)***	1	(1-1)	1	(1-2)	1	(1-1)	1	(1- 2)
**Reported a duration of hypertension, N (%)**	*1,376*	(2.71)	*16,709*	(29.82)	678	(4.32)	4,525	(33.55)
**Duration of hypertension in years, median (range)***	6	(2-14)	8	(4-13)	6	(3-14)	8	(4 - 14)

**Median quantity of antihpertensive medications and median duration of hypertensive diagnosis presented for those on antihypertensive medications and supplied an age of hypertension diagnosis, respectively. MDD = Major Depressive disorder*

For peer review only - http://bmjopen.bmj.com/site/about/guidelines.xhtml

Reporting checklist for cohort study.

Based on the STROBE cohort guidelines.

Instructions to authors

Complete this checklist by entering the page numbers from your manuscript where readers will find each of the items listed below.

Your article may not currently address all the items on the checklist. Please modify your text to include the missing information. If you are certain that an item does not apply, please write "n/a" and provide a short explanation.

Upload your completed checklist as an extra file when you submit to a journal.

In your methods section, say that you used the STROBE cohort reporting guidelines, and cite them as:

von Elm E, Altman DG, Egger M, Pocock SJ, Gotzsche PC, Vandenbroucke JP. The Strengthening the Reporting of Observational Studies in Epidemiology (STROBE) Statement: guidelines for reporting observational studies.

		Reporting Item	Page Number
Title	#1a	Indicate the study's design with a commonly used term in the title or the abstract	1
Abstract	#1b	Provide in the abstract an informative and balanced summary of what was done and what was found	3
Background / rationale	#2	Explain the scientific background and rationale for the investigation being reported	5
Objectives	#3	State specific objectives, including any prespecified hypotheses	5
Study design	#4	Present key elements of study design early in the paper	5
Setting	#5	Describe the setting, locations, and relevant dates, including periods of recruitment, exposure, follow-up, and data collection	6
Eligibility criteria	#6a	Give the eligibility criteria, and the sources and methods of selection of participants. Describe methods of follow-up.	6-7

For peer review only

	#6b	For matched studies, give matching criteria and number of exposed and unexposed	n/a
Variables	#7	Clearly define all outcomes, exposures, predictors, potential confounders, and effect modifiers. Give diagnostic criteria, if applicable	7-8
Data sources / measurement	#8	For each variable of interest give sources of data and details of methods of assessment (measurement). Describe comparability of assessment methods if there is more than one group. Give information separately for for exposed and unexposed groups if applicable.	6-8
Bias	#9	Describe any efforts to address potential sources of bias	6
Study size	#10	Explain how the study size was arrived at	6
Quantitative variables	#11	Explain how quantitative variables were handled in the analyses. If applicable, describe which groupings were chosen, and why	See note 1
Statistical methods	#12a	Describe all statistical methods, including those used to control for confounding	8-9
	#12b	Describe any methods used to examine subgroups and interactions	See note 2
	#12c	Explain how missing data were addressed	6-7
	#12d	If applicable, explain how loss to follow-up was addressed	1
	#12e	Describe any sensitivity analyses	9
Participants	#13a	Report numbers of individuals at each stage of study—eg numbers potentially eligible, examined for eligibility, confirmed eligible, included in the study, completing follow-up, and analysed. Give information separately for for exposed and unexposed groups if applicable.	10
	#13b	Give reasons for non-participation at each stage	6,7
	#13c	Consider use of a flow diagram	n/a
Descriptive data	#14a	Give characteristics of study participants (eg demographic, clinical, social) and information on exposures and potential	10

For peer review only

		confounders. Give information separately for exposed and unexposed groups if applicable.	
	#14b	Indicate number of participants with missing data for each variable of interest	See note 3
	#14c	Summarise follow-up time (eg, average and total amount)	10
Outcome data	#15	Report numbers of outcome events or summary measures over time. Give information separately for exposed and unexposed groups if applicable.	6
Main results	#16a	Give unadjusted estimates and, if applicable, confounder-adjusted estimates and their precision (eg, 95% confidence interval). Make clear which confounders were adjusted for and why they were included	11-12
	#16b	Report category boundaries when continuous variables were categorized	n/a
	#16c	If relevant, consider translating estimates of relative risk into absolute risk for a meaningful time period	n/a
Other analyses	#17	Report other analyses done—e.g., analyses of subgroups and interactions, and sensitivity analyses	See note 4
Key results	#18	Summarise key results with reference to study objectives	12
Limitations	#19	Discuss limitations of the study, taking into account sources of potential bias or imprecision. Discuss both direction and magnitude of any potential bias.	14
Interpretation	#20	Give a cautious overall interpretation considering objectives, limitations, multiplicity of analyses, results from similar studies, and other relevant evidence.	13-17
Generalisability	#21	Discuss the generalisability (external validity) of the study results	15
Funding	#22	Give the source of funding and the role of the funders for the present study and, if applicable, for the original study on which the present article is based	18

Author notes

1. 6,7,8,9, supplementary

For peer review only

2. 8-9, supplementary

3. n/a (supplementary)

4. 11-12, supplemental

The STROBE checklist is distributed under the terms of the Creative Commons Attribution License CC-BY. This checklist was completed on 25. May 2018 using http://www.goodreports.org/, a tool made by the EQUATOR Network in collaboration with Penelope.ai

For peer review only
